# Increasing the versatility of the biphenyl-fused-dioxacyclodecyne class of strained alkynes†

**DOI:** 10.1039/d3ob01712e

**Published:** 2023-12-13

**Authors:** Sam Forshaw, Jeremy S. Parker, William T. Scott, Richard C. Knighton, Neelam Tiwari, Samson M. Oladeji, Andrew C. Stevens, Yean Ming Chew, Jami Reber, Guy J. Clarkson, Mohan K. Balasubramanian, Martin Wills

**Affiliations:** a Department of Chemistry, The University of Warwick Coventry CV4 7AL UK M.wills@warwick.ac.uk; b Early Chemical Development, Pharmaceutical Sciences, IMED Biotech Unit, AstraZeneca Macclesfield SK10 2NA UK; c Warwick Medical School, The University of Warwick Coventry CV4 7AL UK; d School of Chemistry, University of Southampton SO17 1BJ UK

## Abstract

Biphenyl-fused-dioxacyclodecynes are a promising class of strained alkyne for use in Cu-free ‘click’ reactions. In this paper, a series of functionalised derivatives of this class of reagent, containing fluorescent groups, are described. Studies aimed at understanding and increasing the reactivity of the alkynes are also presented, together with an investigation of the bioconjugation of the reagents with an azide-labelled protein.

## Abstract 2

Biphenyl-fused-dioxacyclodecynes are a promising class of strained alkyne for use in Cu-free ‘click’ reactions.

## Graphical abstract

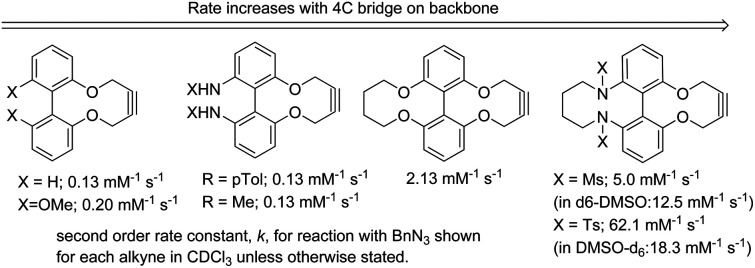

## Introduction

**Fig. 1:**
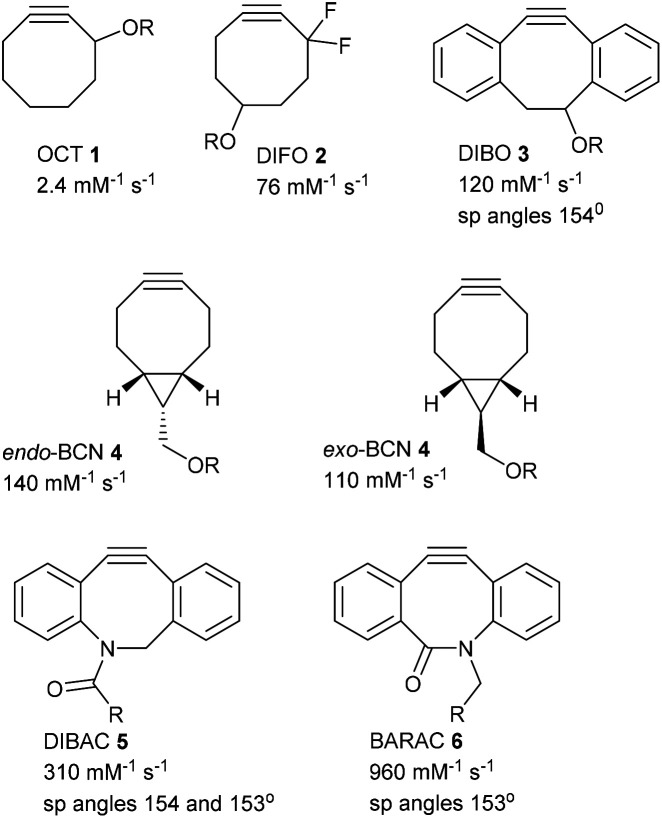
Strained alkynes and their second order rate constants for addition to benzyl azide in MeCN, MeOH or MeCN/H_2_O at rt.^2*d*^ R = functional group.

Bioconjugations^1^ using Strain-Promoted Alkyne–Azide Cycloadditions (SPAAC)^2^ are important reactions, due to their high reaction rates, the lack of a requirement for a catalyst,^3^ and their bioorthogonal reactivity. A number of strained alkynes have been widely adopted for synthetic and biolabelling applications.^4^ Early examples such as OCT 1,^4*a*^ and fluorinated derivatives such as DIFO 2^4*c*^ were followed by highly reactive, strained alkynes such as DIBO 3,^4*d*,*e*^ BCN 4,^4*f*^ DIBAC 5^4*g*^ and BARAC 6^4*h*^ (Fig. 1), which have been employed in numerous biolabelling applications. The second order rate constant for each alkyne with benzylazide provides a convenient means for comparison of their reactivity (Fig. 1). The high reactivity of the strained alkynes is reflected by the distorted sp bond angles. Derivatives of strained alkynes, loaded with a fluorescent group, also undergo cyclisations with azide-containing molecules both *in vitro* and *in vivo* without the need for a catalyst.

**Fig. 2:**
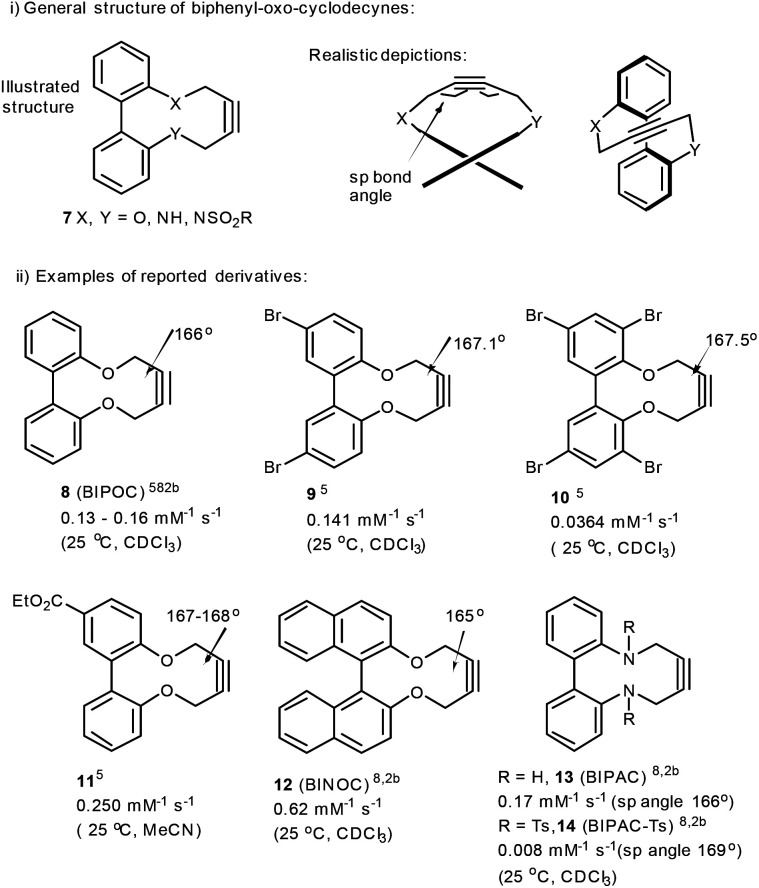
Biphenyl-fused dioxacyclodecynes and second order rate constants for addition with benzylazide under the conditions shown. Bond angles were established by X-ray crystal structure analyses or DFT calculations.

We,^5–7^ and others,^8^ recently reported the synthesis of strained alkynes of general structure 7, where X/Y = O, NH, NTs,^9^ as reagents for copper-free cycloaddition reactions with azides (Fig. 2). Specific examples of this class of alkyne are 8–14 and, although not as reactive as some of the well-established strained alkynes shown in Fig. 1, they benefit from the straightforward introduction of the alkyne through the reaction of a 2,2′-biphenol reagent with 1,4-ditosylbut-2-yne, and readily react with azides at concentrations above *ca*. 0.1 M. Alabugin *et al.*^8^ described how the ‘twisted’ structure of this class of dioxacyclodecyne is alleviated upon approach of the azide. This effect generates improved reactivity when the heteroatom (X, Y) in the structure is an oxygen or a nitrogen atom.

Altering the heteroatoms X/Y in 7 influences their reactivity; Alabugin^8^ studied biphenyl-fused-diazacyclodecyne derivative 13 and observed a similar rate constant to that of the unsubstituted biphenyl-dioxacyclodecyne 8, when reacted with benzyl azide in CDCl_3_ at rt. The *p*-toluenesulfonamide derivative 14 exhibited a lower rate of reaction, corresponding to its less distorted sp bond angle of 169° (Fig. 2).^8^ This is less distorted than in the more reactive unsubstituted alkyne 8 which has an sp angle of *ca*. 166° and significantly less than for highly reactive alkynes such as DIBAC and BARAC (Fig. 1). In this paper, we describe our studies aimed at expanding the range of biphenyl-fused-dioxacyclodecyne reagents, and at increasing their reactivity in Cu-free click reactions.

## Results and discussion

### Fluorescent enone-containing derivatives

**Fig. 3:**
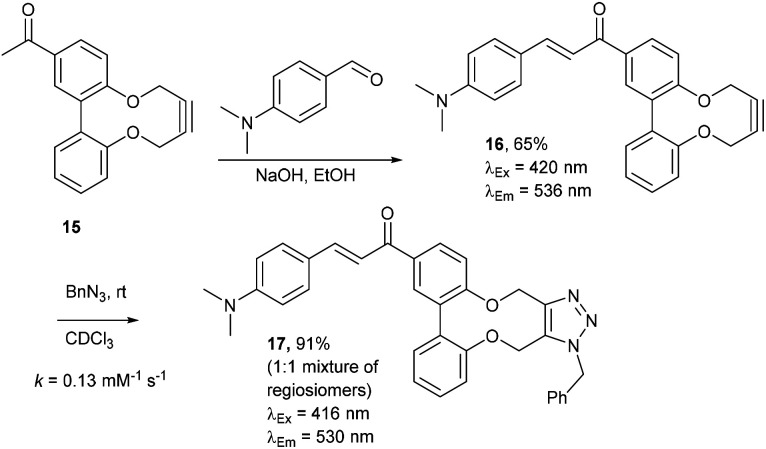
Synthesis of enone alkyne, 16, and subsequent cycloaddition of benzyl azide to form triazole 17.

Enones can exhibit fluorescent properties. A report by Xing *et al.*^10^ indicated that an effective pairing was a compound containing a methoxy group and a dimethylamino group *para*- to the ketone and aldehyde enone precursors respectively. Alkyne 15^6^ was reacted under basic conditions with 4-dimethylaminobenzaldehyde to give 16 in good yield (Fig. 3).

**Fig. 4:**
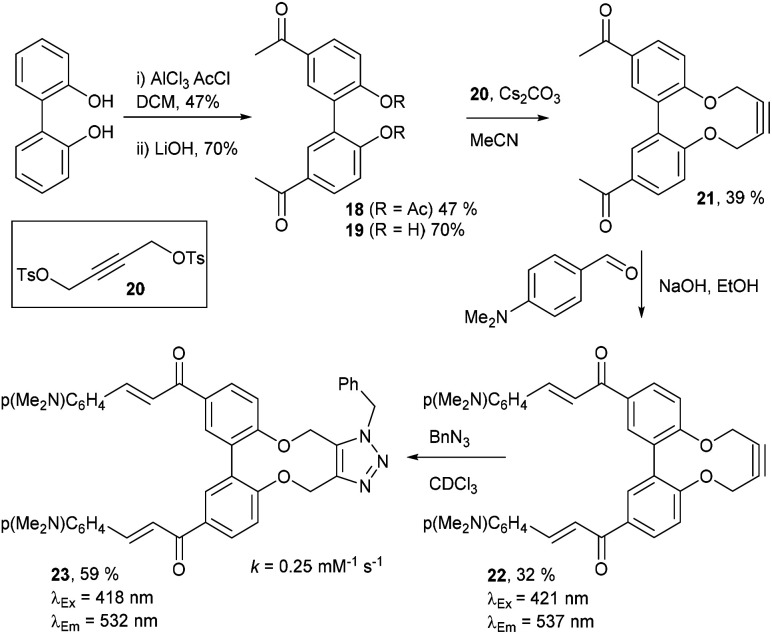
Synthesis of dienone alkyne, 22, and subsequent cycloaddition with benzyl azide to give triazole, 23.

Compound 16 exhibited strong fluorescence excitation and emission maxima at 420 nm and 536 nm respectively. The fluorescent data for the benzyl azide addition product 17 (formed as an inseparable 1 : 1 mixture of regioisomers) exhibited excitation and emission wavelengths essentially unchanged from alkyne 16. The rate constant for the cycloaddition was 0.13 mM^−1^ s^−1^, similar to that of biphenyl-fused-dioxacyclodecyne 8. The reaction of 2,2′-biphenol with an excess of AlCl_3_ and acetyl chloride gave 18 in moderate yield (47%). The ester groups were then hydrolysed using lithium hydroxide to give the diacetyl biphenol 19, and its cyclisation with ditosylate 20 gave the strained diacetyl alkyne 21 (Fig. 4). Dienone 22 was then formed using the same conditions as for the synthesis of compound 17, using two equivalents of 4-dimethylaminobenzaldehyde. Unexpectedly, the reaction rate for the reaction between dienone 22 and benzyl azide (*k* = 0.25 mM^−1^ s^−1^) to give 23 was double that for enone 16, possibly due to steric effects between the two large enone groups on the opposite side to the alkyne.

**Fig. 5:**
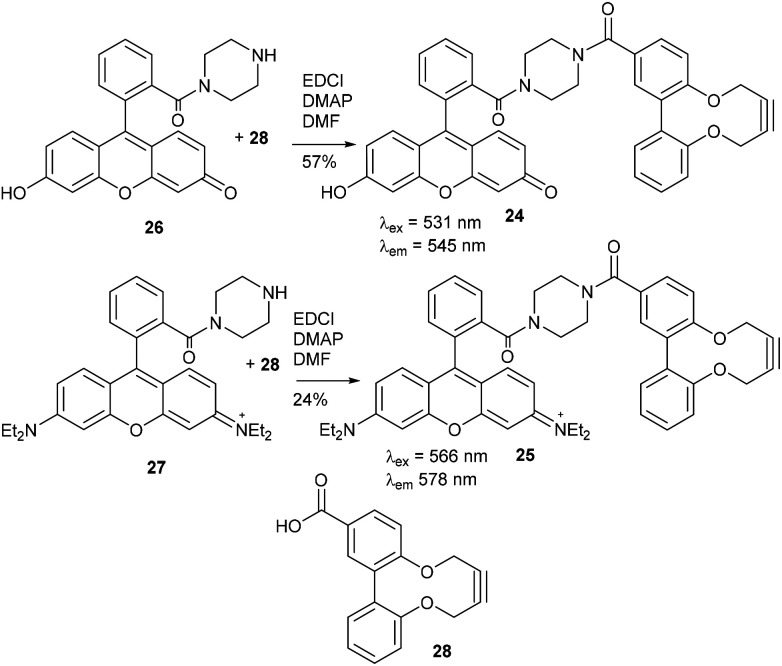
Synthesis of fluorescein and rhodamine-containing strained alkynes.

Two further derivatives, 24 and 25, containing fluorescein and rhodamine groups respectively, were prepared through DCC couplings with known fluorescent precursors 26 and 27, and the strained alkyne 28 (Fig. 5).^11,12^ These were available for subsequent testing with an azide-functionalised protein, which is described in a later section.

### Further derivatives and attempts to increase the reactivity of the alkynes

**Fig. 6:**
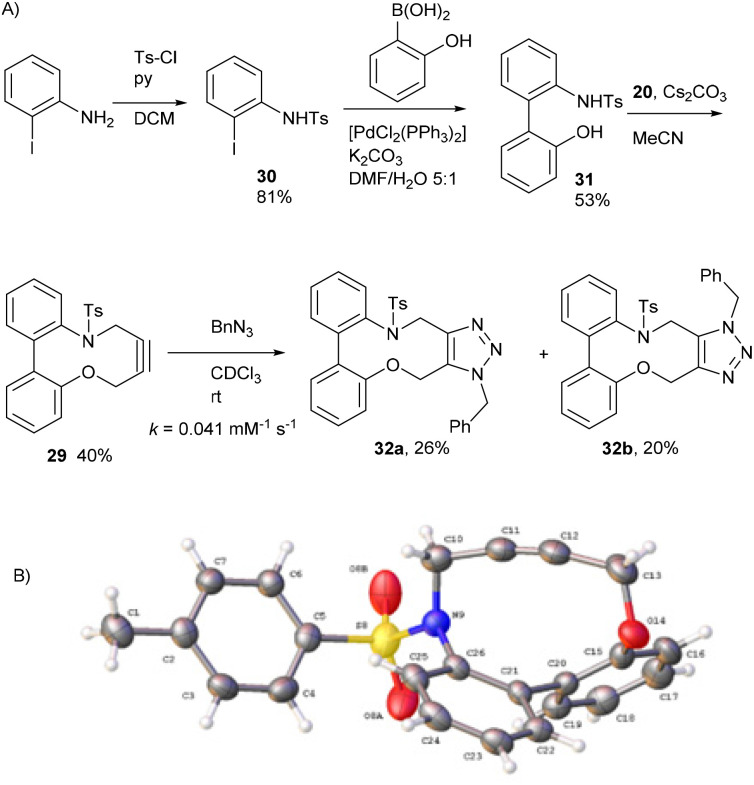
(A) The synthetic route to the heterocyclic alkyne 29 and subsequent reaction with BnN_3_. The regiochemistry of each isomer of 32 has not been confirmed. (B) X-ray crystal structure of 29.

To understand the significance of the heteroatoms on the reactivity, compound 29, containing a combination of an oxygen and toluenesulfonamide heteroatoms, was prepared. 2-Iodoaniline was converted to 30 which was coupled under Suzuki conditions with 2-hydroxyphenylboronic acid to give the biphenyl 31, and subsequently converted to 29 in moderate yield (Fig. 6a).

**Fig. 7:**
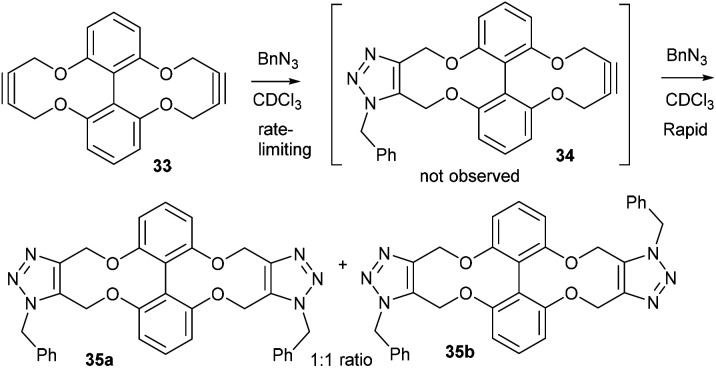
The reaction between bisalkyne, 33, and benzyl azide.

Alkyne 29 underwent cycloaddition with benzyl azide in CDCl_3_ with a second order rate constant of just 0.041 mM^−1^ s^−1^, to form 32, as a mixture of isomers. This rate is lower than the same reaction of biphenyl-fused-dioxacyclodecyne, 8, but higher than the reaction of biphenyl-NTs-alkyne, 14. The X-ray crystal structure of 29 (Fig. 6b) revealed sp bond angles of *ca*. 169.5 and 167.4° respectively. The synthesis of other cyclic alkynes was considered, including the use of sulfur as a heteroatom. However, the introduction of sulfur atoms generally diminishes the reactivity of the alkyne due to the larger bond length of the sulfur–carbon bonds.^13,14^ In an earlier result published by Wills *et al.*,^7^ bisalkyne 33 (Fig. 7) was used in ‘protein stapling’ reactions. Analysis of the reaction by NMR, which featured direct formation of 34 without the monoadduct 35, suggested that the first cycloaddition was rate limiting and that the second cycloaddition occurred much more rapidly. Molecular modelling confirmed that the transition state for the second cycloaddition had a lower energy barrier than the first. This increase in reactivity is likely caused by an increased distortion of the remaining alkyne bond.

**Fig. 8:**
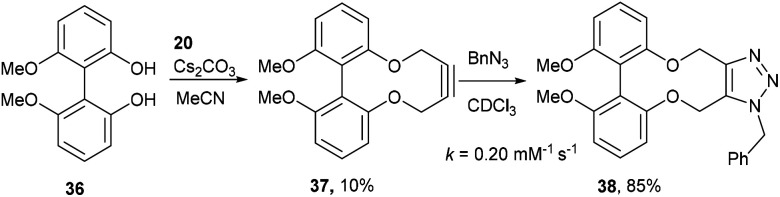
Synthetic route to 6,6′-dimethoxybiphenyl-dioxacyclodecyne 37 and subsequent click reaction with benzyl azide in CDCl_3_.

It was speculated that harnessing this effect into a strained alkyne could be beneficial. To achieve this, we studied the effects that functional groups at the 6 and 6′ positions have on the rates of cycloaddition. The known biphenol 36,^15^ was converted to 6,6′-dimethoxybiphenyl-dioxacyclodecyne 37 in low yield (Fig. 8) but sufficient material was isolated to test the addition reaction. The rate constant for the reaction of 37 with BnN_3_, forming adduct 38, was 0.20 mM^−1^ s^−1^, indicating that methoxy groups at these positions have little effect on the rate of reaction or the structure of the alkyne and the distortion of the alkyne bond.

**Fig. 9:**
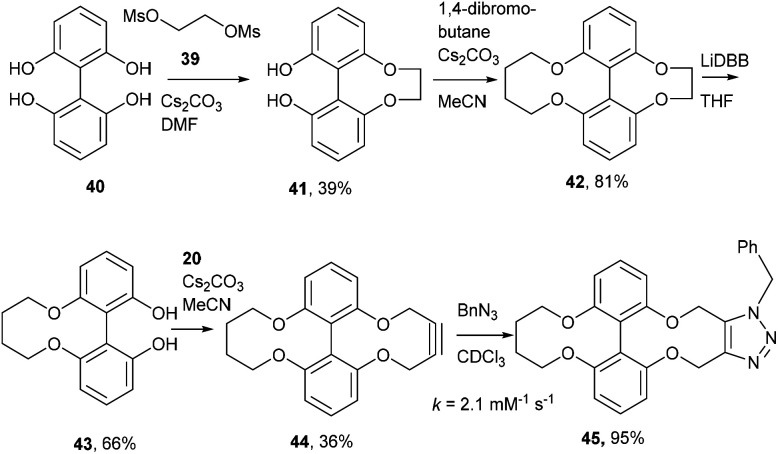
Synthetic route to 44 and subsequent cycloaddition reaction with benzyl azide to produce triazole 45.

A route to asymmetric biphenols with a bridge between the 6 and 6′ positions has been reported using a removable chiral bridging group.^16,17^ Using this approach, dimethylsulfonate, 39 was reacted with the known tetrol 40^7^ under the conditions reported by Harada *et al.*,^16^ producing the ethyl bridged biphenol 41,^18^ in moderate yield. Cyclisation with 1,4-dibromobutane formed the bicyclic compound 42. Lithium di-*tert*-butylbiphenyl (LiDBB) cleaved the more strained ethyl bridge in 42 selectively to produce 43 in good yield and this was then cyclised with alkyne 20 to give the 4C bridged strained alkyne 44 (Fig. 9).^8^

**Fig. 10:**
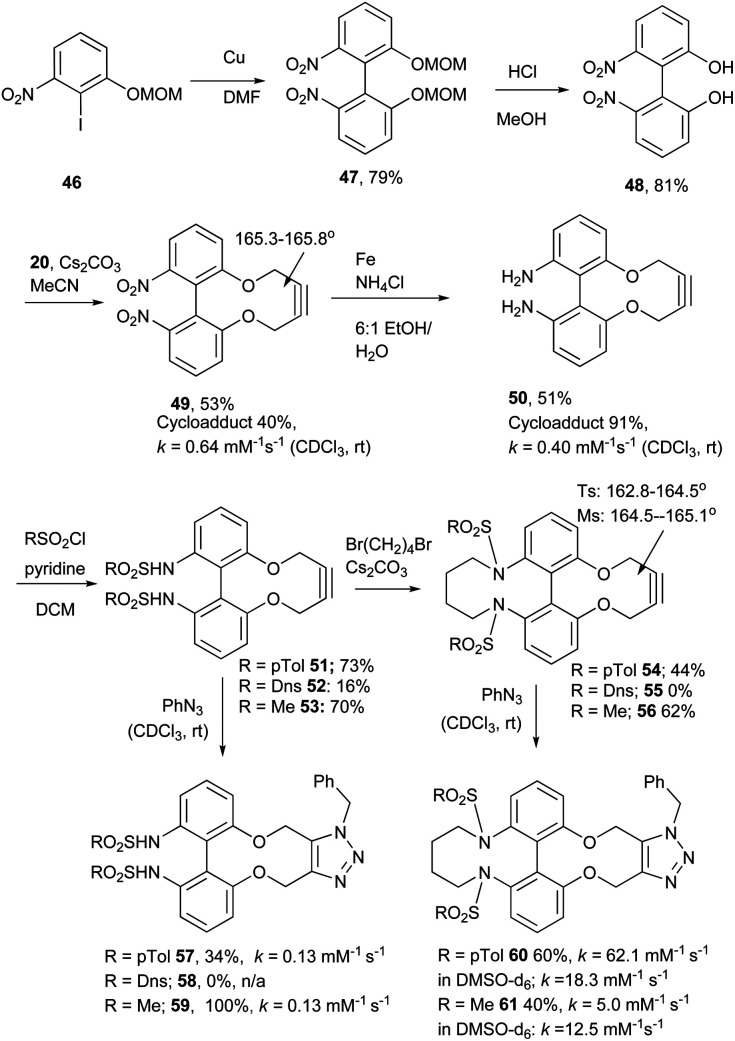
Synthetic route to the 6,6′-dinitrobiphenyl-dioxacyclodecyne 49 and derivatives, with rate constraints for subsequent cycloadditions with benzyl azide in CDCl_3_(unless otherwise indicated) Where sp bond angles are given, these were determined by X-ray crystallography (Fig. 11).

The 4C-oxo-bridged biphenyl-fused-dioxabiphenylcyclodecyne 44, reacted with benzyl azide to give adduct 45, with a rate constant of 2.1 mM^−1^ s^−1^, representing an increase compared to analogous biphenyl-fused-dioxacyclodecyne 37 (*k* = 0.17 mM^−1^ s^−1^). The increase in reactivity is likely caused by the 6,6′-4C bridge forcing the phenyl rings to lie in a more planar structure and providing more distortion to the alkyne bond angles. To increase this effect further, the synthesis of a three-carbon bridged derivative was attempted, however this was not successful. Given the promising result with 44, an N-containing C4-bridged reagent was prepared. Ullmann homo-coupling of 46^19^ with activated copper was carried out to give protected biphenol 47 in high yield. An attempt at the Ullmann coupling of the unprotected analogue of 46 was unsuccessful. Deprotection of 47 gave biphenol 48 in high yield, which was then cyclised to the strained alkyne 49 (Fig. 10).

**Fig. 11:**
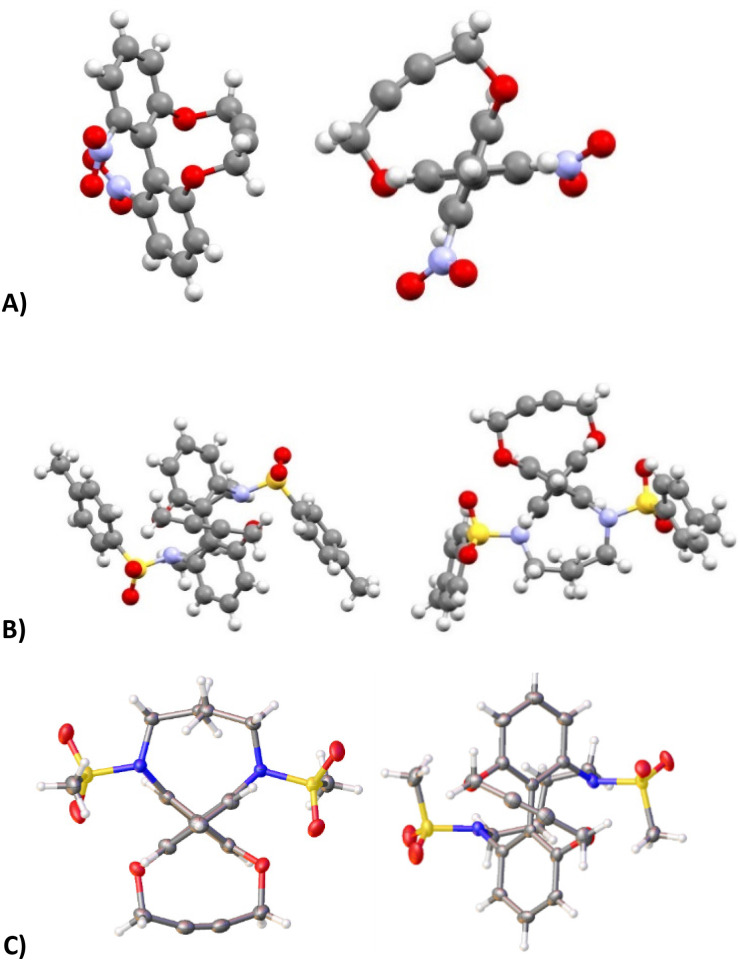
(A) Two views of the X-ray crystal structure of 6,6′-dintrobiphenyl-dioxacyclodecyne 49. (B) Two views of the X-ray crystal structure of bridged alkyne 54. (C) Two views of the X-ray crystal structure of bridged alkyne 56.

It was found that the use of iron powder and ammonium chloride selectively reduced the nitro groups to amines to give 50, leaving the alkyne intact (Fig. 10). 6,6′-Diamino-dioxacyclodecyne 50 was then reacted with toluenesulfonyl chloride, dansyl chloride and mesyl chloride under basic conditions to give 51 (Ts), 52 (Dns) and 53 (Ms) respectively. To create the anticipated more reactive derivatives, each bisulfonamide was reacted with 1,4-dibromobutane under basic conditions using a syringe pump to maintain a pseudo-dilute solution.^8^ These studies afforded the bridged product, 54, in moderate yield from the ditosylate precursor. Unfortunately, the bis-dansyl precursor 52 gave no corresponding product 55, likely due to increased steric hindrance. Tests on the cyclisation of dimesylate 53 with varying equivalents of 1,4-dibromobutane revealed that the use of three equivalents of the dibromide gave an improved yield of C4-cyclised product 56 over the use of one equivalent. This was surprising as we were concerned that an excess of the dihalide would result in dialkylation of the dimesylate prior to intramolecular cyclisation. However, the improved yield indicates that the intramolecular step must outpace the second *N*-alkylation. The cycloadditions of the new alkynes with benzyl azide in CDCl_3_ (at rt) were tested (Fig. 10). For 49, the rate constant calculated for this reaction was 0.64 mM^−1^ s^−1^; an improvement of about a factor of four compared to 37, suggesting that the electron-withdrawing nitro group increases the reactivity. The X-ray crystal structure of 49 (Fig. 11a) indicates that the alkyne bond angles average 165.6°, more distorted than the biphenyl-fused-dioxacyclodecyne, 8. For diamine 50, the rate constant was 0.40 mM^−1^ s^−1^; slightly lower than that for 49, but slightly higher than that observed for the reaction with biphenyl-fused-dioxacyclodecyne 37. Bisulfonylated compounds 51 and 53 produced the triazole products 57 and 59 respectively upon reaction with benzyl azide, however due to the low solubility of alkyne 52, a rate constant could not be accurately determined and the anticipated product 58 was not isolated. The reaction between 51 (Ts) and 53 (Ms) and benzyl azide gave rate constants of 0.13 mM^−1^ s^−1^ in each case, similar to that of biphenyl-fused-dioxacyclodecyne 8. However, the corresponding cycloaddition reactions of 54 and 56 proceeded with significantly higher rate constants of 62.1 and 5.0 mM^−1^ s^−1^ respectively, in CDCl_3_ to give products 60 and 61 respectively. The reaction of 54 in an NMR tube was substantially complete within 5 hours ([alkyne] = 0.04 mM), representing a step change in reactivity for this class of strained alkynes. X-ray structures of 54 and 46 (Fig. 11b and c), revealing the alkyne sp bond angles to be 163.7° and 162.8° in 54 and 164.0°/165.1° in 56. The difference in average alkyne bond angle between compound 54 and compound 49 is only 2.3°, which shows even small changes to the bond angle can have a great influence on the reaction rate. For 56 (diMs) the angles were intermediate, and this was reflected in its reactivity.

When comparing the rate of the reaction between alkyne 54 and benzyl azide with previously published strained alkynes it displays similar reactivity to the difluorinated cyclooctynes, which display rate constants between 42–76 mM^−1^ s^−1^.^2*d*^ The comparative rate constants of the novel compounds in this study give an insight into how electronic and structural effects can combine to produce more reactive alkynes. Although using electron withdrawing groups at the 6 and 6′ position in 49 did improve the reactivity, the largest increases in reactivity came with the addition of the 6,6′-4C bridge, *i.e.* in 44, 54 and 56. The reason for the difference in reactivity between 54 and 56 may stem from an increase strain created by the bulky tosyl groups creating increased distortion in the alkyne bond.

To establish whether the new alkynes may be compatible with biomolecules, attempts were made to react the novel alkynes with glutathione *S*-transferase (GST) containing an azidophenylalanine at position 52. In an initial series of tests, an earlier-reported BoDIPY-containing strained alkyne, fluorescein 24 and rhodamine derivative 25 were reacted and a gel indicated that conjugation had occurred in most cases. However, MS analyses of the adducts indicated that this was only the case for the fluorescein derivative 24, hence there may be non-covalent, non-specific interactions between protein and dye in the other cases (see the ESI†). In a second round of tests of non-fluorescent compounds, the dimesylated compound 56 gave an addition product when analysed by mass spectrometry, although the more reactive ditosylated 54 did not. Examination of the second order rate constant for the reaction with BnN_3_ in DMSO-d_6_ (*i.e.* reflecting more closely the conditions used in the enzyme reactions where DMSO/H_2_O was used) gave *k* values of 18.3 and 12.5 mM^−1^ s^−1^ for 54 and 56 respectively. Hence the rates of each compound were closer in DMSO-d_6_ than in CHCl_3_. Coupled to a potential lower solubility of the larger molecule in the water/DMSO mixture used with the enzyme may account for differences in the observed results. Compound 44, bearing a 4C aliphatic linking group, added to the protein, but at a low level. See the ESI† for full details of these tests.

## Conclusion

The development of novel strained alkynes for use in bioconjugation is still a focus of international research. Biphenyl-fused-dioxacyclodecynes react with azides without the need for a catalyst. The current investigation into the reactivity of this class of strained alkyne has led to the synthesis of a variety of 6,6′-functionalised biphenyl-fused-dioxacyclodecyne derivatives with rate constants in the region of 0.13–0.64 mM^−1^ s^−1^. This inspired the synthesis of a four-carbon bridged class of biphenyl-fused-dioxacyclodecynes, which are more reactive towards azides, with rate constants between 2.13–62.1 mM^−1^ s^−1^. There is potential for functionalisation of the sulfonamide groups of the C4-bridged alkynes, *e.g.* with fluorescent groups, which could provide a reactive, fluorescent strained biphenyl alkyne for use in bioimaging. Further studies of the value of the alkynes, of which 56 represents a promising candidate for further applications, are ongoing.

## Experimental section

Solvents and reagents were degassed before use and all reactions were carried out under a nitrogen atmosphere using vacuum line apparatus. Reactions were monitored by TLC using aluminium backed silica gel 60 (F254) plates, visualized using UV 254 nm and phosphomolybdic acid or potassium permanganate as appropriate. Flash column chromatography was carried out routinely on silica gel. Reagents were used as received from commercial sources unless otherwise stated. Dry solvents were purchased and used as received. ^1^H NMR spectra were recorded on a Bruker DPX (300, 400 or 500 MHz) spectrometer. Chemical shifts are reported in *δ* units, parts per million relative to the singlet at 7.26 ppm for chloroform and 0.00 ppm for TMS. Coupling constants (*J*) are measured in hertz. Mass spectra for analysis of synthetic products were recorded on a Bruker Esquire 2000 or a Bruker MicroTOF mass spectrometer. IR spectra were recorded on a PerkinElmer Spectrum One FT-IR Golden Gate. Melting points were recorded on a Stuart Scientific SMP 1 instrument and are uncorrected. X-ray crystallography was carried out on a Rigaku Oxford Diffraction SuperNova diffractometer with a duel source (Cu at zero) equipped with an AtlasS2 CCD area detector or an Xcalibur Gemini diffractometer with a Ruby CCD area detector. The procedure and full details of the kinetic ^1^H NMR runs are given in the ESI.†

### Safety and hazards

All synthetic organic chemistry has potential hazards, however azides are known to be highly reactive and required full risk assessment and care in handling throughout their preparation, use and disposal. In this study, small amounts of benzylazide (typically <10 mg) were used in NMR-scale tests of reactivity with the strained alkynes.

The following compounds were prepared following published methods; ditosyl-1,4-dihydroxybut-2-yne 20,^21^*N*-tosyl-2-iodoaniline 30,^20^ dimethoxydiphenol 36,^15^ tetrahydroxybiphenyl 40,^7^ ethanedioldimesylate 39,^22^ ethylbridged tetrahydroxybiphenyl 41^18^ and the MOM derivative of 2-iodo-3-nitrophenol 46.^19^

### 5-(3-(4-Dimethylaminophenyl)-1-oxo-prop-2-ene)-2,2′-biphenyldioxacyclodecyne (16) 


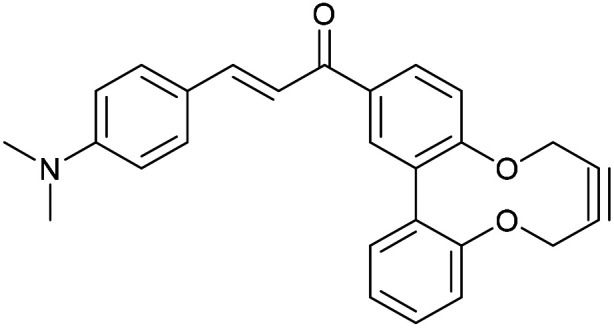
This compound is novel. A solution of 15 (100 mg, 0.360 mmol, 1.0 eq.), 4-dimethylaminobenzaldehyde (58 mg, 0.39 mmol, 1.1 eq.) and NaOH (43 mg, 1.1 mmol, 3.0 eq.) in EtOH (2 mL) was stirred at room temperature for 24 h. H_2_O (20 mL) was added and the product was extracted with EtOAc (3 × 20 mL). The combined organic extracts were dried over MgSO_4_ and concentrated. Purification by column chromatography (eluted with 50% EtOAc/hexane) gave the pure product as a yellow solid (96 mg, 0.23 mmol, 65%). *R*_f_ = 0.38 (1 : 1 EtOAc/hexane); (found (ESI)) 432.1563 C_27_H_23_NNaO_3_ requires 432.1570; *v*_max_ 2916, 2865, 1641, 1565, 1526, 1180, 1170, 966, 802 cm^−1^; *δ*_H_ (500 MHz, CDCl_3_) 8.08 (1 H, dd, *J* = 8.4, 2.1 Hz, Ar***H***) 7.89 (1 H, d, *J* = 2.1 Hz, Ar***H***) 7.80 (1 H, d, *J* = 15.4 Hz, COCHC***H***Ph) 7.53 (2 H, d, *J* = 8.9 Hz, Ar***H***) 7.42 (1 H, ddd, *J* = 8.0, 6.5, 2.7 Hz, Ar***H***) 7.32 (1 H, d, *J* = 15.4 Hz, COC***H***CHPh) 7.28 (1 H, d, *J* = 8.4 Hz, Ar***H***) 7.19–7.25 (3 H, m, Ar***H***) 6.67 (2 H, d, *J* = 8.9 Hz, Ar***H***) 4.52–4.62 (2 H, m, OC***H***_***a***_H_b_) 4.32–4.44 (2 H, m, OCH_a_***H***_***b***_) 3.03 (6 H, s, NC***H***_**3**_); *δ*_C_ (125 MHz, CDCl_3_) 189.5, 158.0, 154.5, 152.0, 145.7, 135.8, 135.3, 135.1, 132.5, 131.9, 130.5, 129.5, 129.4, 124.3, 122.9, 122.7, 122.7, 116.7, 111.8, 87.2, 86.3, 63.7, 63.5, 40.1 ppm; *m*/*z* (ESI) 410.2 [M + H]^+^, 432.2 [M + Na]^+^; Fluorescence (MeCN; *λ*_ex_ = 420 nm); *λ*_em_ = 536 nm; UV-Vis (MeCN) *λ*_max_ (*ε*/M^−1^ cm^−1^): 413 (486 976) nm.

### (*E*)-1-(1-Benzyl-4,15-dihydro-1*H*-dibenzo[7,8:9,10][1,6]dioxecino[3,4-*d*][1,2,3]triazol-8-yl)-3-(4-(dimethylamino)phenyl)prop-2-en-1-one (17)


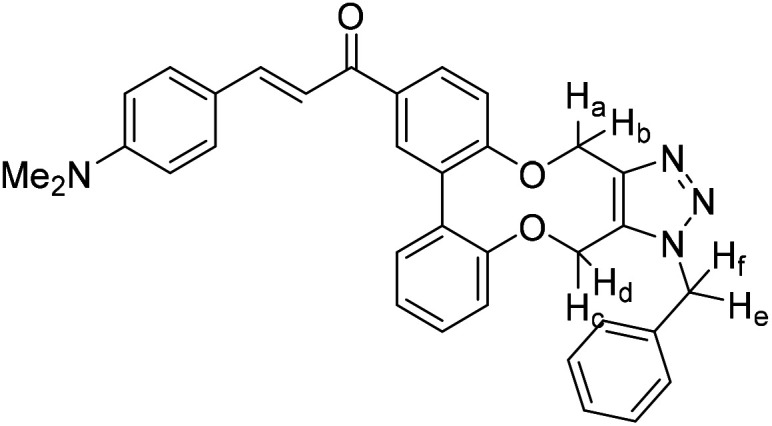
This compound is novel. A solution of 16 (10 mg, 0.024 mmol, 1 eq.) and benzyl azide (3.2 mg, 0.024 mmol, 1 eq.) in CDCl_3_ (0.5 mL) was monitored by NMR until completion of the reaction. The solvent was removed under vacuum and the crude material was purified by column chromatography (eluted with 50% EtOAc/hexane) to give the pure product as two inseparable isomers in a 1 : 1 ratio as an orange solid (11.8 mg, 0.022 mmol, 91%).

*R*_f_ = 0.16 (1 : 1 EtOAc/hexane); (found (ESI)) 543.2379 C_34_H_31_N_4_O_3_ requires 543.2391; *v*_max_ 2923, 1570, 1521, 1495, 1443, 1433, 1332, 1261, 1180, 1167, 1107, 810, 750 cm^−1^; *δ*_H_ (500 MHz, CDCl_3_) 8.00 (0.5 H, dd, *J* = 8.5, 2.1 Hz, PhC***H***CHCO) 7.92 (1 H, dd, *J* = 6.6, 2.1 Hz, Ar***H***) 7.82 (0.5 H, dd, *J* = 8.5, 2.1 Hz, PhC***H***CHCO) 7.78 (1 H, d, *J* = 15.4 Hz, PhC***H***CHCO) 7.53 (2 H, d, *J* = 8.8 Hz, Ar***H***), 7.38–7.42 (2 H, m, Ar***H***), 7.32–7.37 (3 H, m, Ar***H***), 7.26–7.05 (7 H, m, Ar***H***), 6.78 (0.5 H, d, *J* = 7.9 Hz, PhCHC***H***CO) 6.68 (2 H, d, *J* = 8.8 Hz, Ar***H***) 6.59 (0.5 H, d, *J* = 8.5 Hz, PhCHC***H***CO) 5.81 (0.5 H, d, *J* = 16.0 Hz, C***H***_***e***_H_f_) 5.77 (0.5 H, d, *J* = 16.0 Hz, C***H***_***e***_H_f_) 5.63 (0.5 H, d, *J* = 13.7 Hz, CH_c_***H***_***d***_) 5.35–5.45 (2.5 H, m, CH_e_***H***_***f***_ + C***H***_***c***_H_d_ + CH_c_***H***_***d***_) 5.22 (1.5 H, m, 2 × OC***H***_***a***_H_b_ + OCH_a_***H***_***b***_) 5.05 (0.5 H, d, *J* = 13.0 Hz, OCH_a_***H***_***b***_) 3.03 (6 H, s, NC***H***_3_); (125 MHz, CDCl_3_) 188.8, 188.8, 159.4, 158.8, 156.9, 156.0, 152.0, 151.9, 145.5, 145.2, 144.8, 144.5, 134.6, 134.4, 134.1, 133.1, 132.3, 132.1, 131.2, 131.2, 130.7, 130.5, 130.4, 130.3, 130.1, 129.7, 129.6, 129.6, 129.4, 129.3, 129.2, 128.9, 128.9, 128.7, 127.2, 127.1, 123.6, 122.8, 122.7, 122.4, 116.5, 116.4 116.0, 114.7, 114.4, 113.5, 111.8, 63.6, 62.9, 61.0, 60.4, 52.6, 52.4, 40.1 ppm; *m*/*z* (ESI) 543.2 [M + H]^+^, 565.2 [M + Na]^+^; fluorescence (MeCN; *λ*_ex_ = 416 nm); *λ*_em_ = 530 nm; UV-Vis (MeCN) *λ*_max_ (*ε*/M^−1^ cm^−1^): 410 (70 000) nm.

### 5,5′-Diacetyl-[1,1′-biphenyl]-2,2′-diyl diacetate (18)


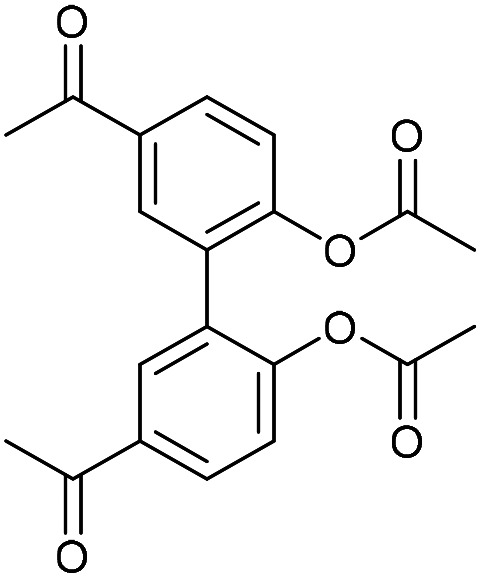
This compound is novel. A solution of AlCl_3_ (6.00 g, 45 mmol, 8.3 eq.) in DCM (4 mL) was cooled to 0 °C. Acetyl chloride (4.40 g, 56.0 mmol, 10.4 eq.) was added to the solution and the reaction mixture was stirred for 30 min. A solution of 2,2′-biphenol (1.00 g, 5.4 mmol, 1.0 eq.) in DCM (10 mL) was added to the reaction mixture at 0 °C and the mixture was stirred for a further 30 min. The reaction was then refluxed until completion, at which point H_2_O (30 mL) was added dropwise to quench. The product was extracted with EtOAc (3 × 30 mL) and the combined organic extracts were dried over MgSO_4_, which was removed by filtration, and concentrated under vacuum to give the crude product. Purification by column chromatography gave the pure product as a white solid (892 mg, 2.5 mmol, 47%).

*R*_f_ = 0.60 (1 : 1 EtOAc/Pet Ether); mp = 205–209 °C; (found (ESI)) 377.0982 C_20_H_18_NaO_6_ requires 377.0996; *v*_max_ 1740, 1683, 1600, 1355, 1191, 910, 619 cm^−1^; *δ*_H_ (500 MHz, CDCl_3_) 8.07 (2 H, dd, *J* = 8.5, 2.1 Hz, Ar***H***) 7.97 (2 H, d, *J* = 2.1 Hz, Ar***H***) 7.33 (2 H, d, *J* = 8.5 Hz, Ar***H***) 2.65 (6 H, s, COC***H***_**3**_) 2.09 (6 H, s, OCOC***H***_**3**_) ppm; *δ*_C_ (125 MHz, CDCl_3_) 196.5, 168.6, 151.7, 135.0, 131.6, 130.0, 129.6, 123.0, 26.7, 20.7 ppm; *m*/*z* (ESI) 377.1 [M + Na]^+^.

### 1,1′-(6,6′-Dihydroxy-[1,1′-biphenyl]-3,3′-diyl)bis(ethan-1-one) (19)


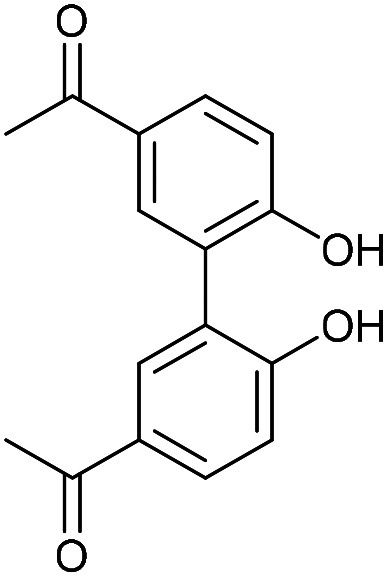
This compound is novel. A solution of compound 18 (848 mg, 2.25 mmol, 1 eq.) and LiOH (302 mg, 12.6 mmol, 5.6 eq.) in MeOH/H_2_O 1 : 1 (10 mL) was refluxed for 2 h. The mixture was then cooled to room temperature before 2 M HCl (20 mL) was added. The product was then extracted with EtOAc (3 × 20 mL), the combined organic extracts were dried over MgSO_4_ and concentrated and recrystalised in MeOH to give the pure product as a white solid (472 mg, 1.76 mmol, 70%).

*R*_f_ = 0.2 (1 : 1 EtOAc/Pet Ether); mp = 177–181 °C; (found (ESI)) 293.0782 C_16_H_14_NaO_4_ requires 293.0784; *v*_max_ 3222, 1651, 1579, 1383, 1354, 1255, 818, 583 cm^−1^; *δ*_H_ (500 MHz, DMSO-d_6_) 10.30 (2 H, s, O***H***) 7.84 (2 H, dd, *J* = 8.5, 2.3 Hz, Ar***H***) 7.78 (2 H, d, *J* = 2.3 Hz, Ar***H***) 7.00 (2 H, d, *J* = 8.5 Hz, Ar***H***) 2.50 (6 H, s, OC***H***_**3**_); *δ*_C_ (125 MHz, d^6^-DMSO) 196.2, 159.6, 132.3, 129.6, 128.3, 124.9, 115.4, 26.3 ppm; *m*/*z* (ESI) 293.1 [M + Na]^+^.

### 5,5′-Diacetyl-2,2′-biphenyldioxacyclodecyne (21) 


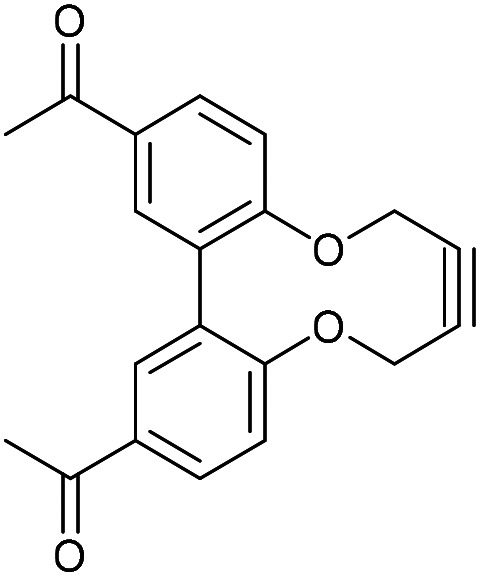
This compound is novel. To a solution of Cs_2_CO_3_ (1.33 g, 4.1 mmol, 2.2 eq.) in MeCN (43 mL), at 60 °C, was added a solution of ditosylate 20 (733 mg, 1.86 mmol, 1 eq.) and compound 19 (500 mg, 1.86 mmol, 1 eq.) in MeCN (8.7 mL) over 6 h. The mixture was stirred for a further 12 h before being cooled to room temperature and the solvent removed under vacuum. The residue was dissolved in H_2_O (30 mL) and the product was extracted with EtOAc (3 × 30 mL). The combined organic extracts were dried over MgSO_4_ and concentrated to give the crude product, which was purified by column chromatography (eluted with 50% EtOAc/hexane) to give the crude product as a white solid (233 mg, 0.73 mmol, 39%).

*R*_f_ = 0.42 (1 : 1 EtOAc/Pet. Ether); mp = 188–189 °C; (found (ESI)) 343.0935 C_20_H_16_NaO_4_ requires 343.0941; *v*_max_ 3060, 2919, 1673, 1594, 1477, 1238, 1191, 956, 676 cm^−1^; *δ*_H_ (500 MHz, CDCl_3_) 8.05 (2 H, dd, *J* = 8.5, 2.3 Hz, Ar***H***), 7.82 (2 H, d, *J* = 2.3 Hz, Ar***H***) 7.28 (2 H, d, *J* = 8.5 Hz, Ar***H***) 4.55–4.64 (2 H, m, OC***H***_***a***_H_b_) 4.36–4.44 (2 H, m, OCH_a_***H***_***b***_) 2.60 (6 H, s, COC***H***_3_); *δ*_C_ (125 MHz, CDCl_3_) 197.1, 158.7, 135.2, 133.4, 132.6, 129.7, 123.1, 86.7, 63.7, 26.7 ppm; *m*/*z* (ESI) 343.1 [M + Na]^+^.

### 5,5′-Bis(3-(4-dimethylaminophenyl)-1-oxo-prop-2-ene)-2,2′-biphenyldioxacyclodecyne (22) 


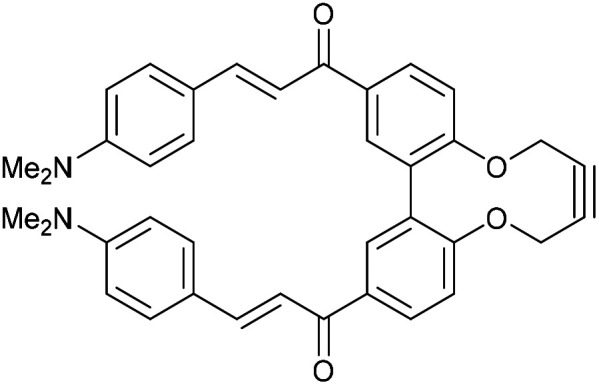
This compound is novel. A solution of compound 21 (99.2 mg, 0.310 mmol, 1.0 eq.), 4-dimethylamino benzaldehyde (101 mg, 0.680 mmol, 2.2 eq.) and NaOH (74 mg, 1.9 mmol, 6 eq.) in EtOH (2 mL) was stirred at room temperature for 12 h. H_2_O (20 mL) was added, and the product was extracted with EtOAc (3 × 20 mL). The combined organic extracts were then dried over MgSO_4_ and concentrated. Purification by column chromatography (eluted with 50% EtOAc/hexane) gave the pure product as an orange solid (59 mg, 0.10 mmol, 32%).

*R*_f_ = 0.26 (1 : 1 EtOAc/hexane); (found (ESI)) 605.2396 C_38_H_34_N_2_NaO_4_ requires 605.2411; *v*_max_ 2906, 2854, 1647, 1569, 1518, 1331, 1163, 1109, 810, 747 cm^−1^; *δ*_H_ (500 MHz, CDCl_3_) 8.12 (2 H, dd, *J* = 8.4, 2.1 Hz, Ar***H***), 7.91 (2 H, d, *J* = 2.1 Hz, Ar***H***), 7.81 (2 H, d, *J* = 15.4 Hz, COCHC***H***Ph), 7.55 (4 H, d, *J* = 8.9 Hz, Ar***H***), 7.34 (2 H, d, *J* = 15.4 Hz, COC***H***CHPh), 7.29–7.34 (2 H, m, Ar***H***), 6.69 (4 H, d, *J* = 8.9 Hz, Ar***H***), 4.52–4.68 (2 H, m, OC***H***_***a***_H_b_), 4.33–4.49 (2 H, m, OCH_a_***H***_***b***_), 3.09 (12 H, s, NC***H***_**3**_) ppm; *δ*_C_ (125 MHz, CDCl_3_) 189.4, 158.0, 152.0, 145.8, 135.2, 132.4, 130.5, 129.8, 123.0, 122.7, 116.7, 111.8, 86.8, 63.7, 40.1 ppm; *m*/*z* (ESI) 583.3 [M + Na]^+^, 605.2 [M + Na]^+^; fluorescence (MeCN; *λ*_ex_ = 420 nm); *λ*_em_ = 536 nm; UV-Vis (MeCN) *λ*_max_ (*ε*/M^−1^ cm^−1^): 416 (148 000) nm.

### (2*E*,2′*E*)-1,1′-(1-Benzyl-4,15-dihydro-1*H*-dibenzo[7,8:9,10][1,6]dioxecino[3,4-*d*][1,2,3]triazole-8,11-diyl)bis(3-(4-(dimethylamino)phenyl)prop-2-en-1-one) (23)


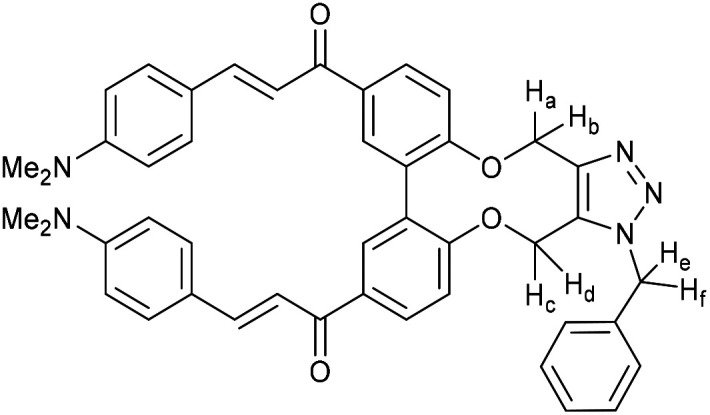
This compound is novel. A solution of compound 22 (10 mg, 0.017 mmol, 1 eq.) and benzyl azide (2.3 mg, 0.017 mg, 1 eq.) in CDCl_3_ (0.5 mL) was monitored by NMR until completion. The solvent was removed under vacuum and the crude material was purified by column chromatography (eluted with EtOAc) to give the pure product as a red solid (7.0 mg, 0.010 mmol, 58%).

*R*_f_ = 0.20 (1 : 1 EtOAc/hexane); (found (ESI)) 716.3214 C_45_H_42_N_5_O_4_ requires 716.3231; *v*_max_ 1575, 1521, 1334, 1167, 1117, 1026, 979, 809 cm^−1^; *δ*_H_ (500 MHz, CDCl_3_) 8.06 (1 H, dd, *J* = 8.5, 2.1 Hz, Ar***H***), 8.02 (1 H, d, *J* = 2.1 Hz, Ar***H***) 8.00 (1 H, d, *J* = 2.1 Hz, Ar***H***), 7.94–7.99 (1 H, m, Ar***H***) 7.90–7.95 (1 H, m, Ar***H***) 7.78–7.84 (2 H, m, PhC***H***CHCO) 7.55 (4 H, m, *J* = 8.7, 3.5 Hz, Ar***H***) 7.40 (2 H, m, PhCHC***H***CO) 7.33–7.38 (3 H, m, Ar***H***) 7.20 (3 H, m, *J* = 6.1, 2.9 Hz, Ar***H***) 6.68 (4 H, m, *J* = 8.9 Hz, Ar***H***) 5.82 (1 H, d, *J* = 15.7 Hz, C***H***_***e***_H_f_) 5.58 (1 H, d, *J* = 13.4 Hz, OC***H***_***c***_H_d_) 5.39–5.47 (2 H, m, OCH_c_***H***_***d***_ + CH_e_***H***_***f***_) 5.29 (1 H, d, *J* = 13.4 Hz, OC***H***_***a***_H_b_) 5.15 (1 H, d, *J* = 13.4 Hz, OCH_a_***H***_***b***_) 3.03 (12 H, s, NC***H***_**3**_); *δ*_C_ (125 MHz, CDCl_3_) 188.7, 159.7, 159.1, 152.0, 152.0, 145.7, 145.4, 144.5, 134.4, 134.4, 133.4, 132.0, 131.1, 131.1, 130.5, 130.4, 130.1, 130.0, 129.8, 129.4, 129.0, 128.9, 127.2, 122.7, 122.7, 116.4, 116.3, 115.6, 114.0, 111.8, 63.5, 60.8, 52.7, 40.1 ppm; *m*/*z* (ESI) 716.3 [M + H]^+^, 738.3 [M + Na]^+^; fluorescence (MeCN; *λ*_ex_ = 418 nm); *λ*_em_ = 532 nm; UV-Vis (MeCN) *λ*_max_ (*ε*/M^−1^ cm^−1^): 409 (199 900) nm.

### Fluorescein amide 2,2′-biphenyldioxacyclodecyne (24)


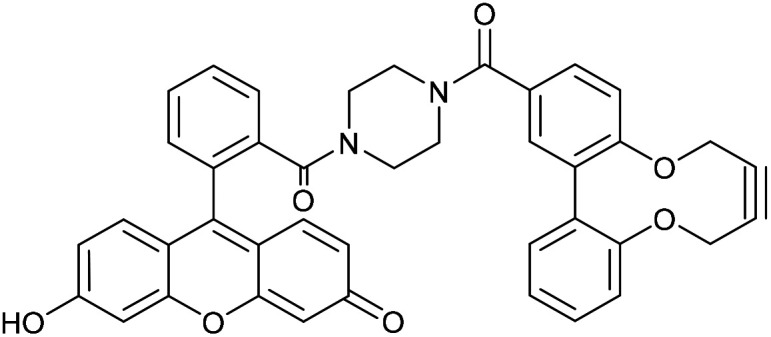
This compound is novel. 6-Hydroxy-9-(2-(piperazine-1-carbonyl)phenyl)-3*H*-xanthen-3-one 26, synthesised as previously described,^11^ (78.0 mg, 0.195 mmol) and acid alkyne 28 (54.6 mg, 0.195 mmol) were dissolved in anhydrous DMF (2 mL). DMAP (59.5 mg, 0.287 mmol) and EDCI (74.9 mg, 0.390 mmol) were added and the reaction stirred under N_2_ at room temperature for 18 hours. H_2_O (10 mL) was added, extracted with CH_2_Cl_2_/IPA (4 : 1) (3 × 10 mL) and the combined organic layers dried over MgSO_4_. The crude mixture was purified by column chromatography (SiO_2_; CH_2_Cl_2_/MeOH; 100 : 0 → 90 : 10) to afford the compound as an orange solid (74 mg, 0.111 mmol, 57%). *R*_f_ = 0.60 (4 : 1 DCM/MeOH); mp 187–198 (dec) °C; (found (ESI) [M + H]^+^, 663.2125. C_41_H_31_N_2_O_7_ requires [M + H]^+^, 663.2126); *ν*_max_ 1591, 1417, 1379, 1195, 1001, 964 and 847 cm^−1^; *δ*_H_ (500 MHz, CD_3_OD) 7.90 (2 H, s, Ar***H***), 7.80–7.61 (3 H, m, Ar***H***), 7.53–7.46 (1 H, m, Ar***H***), 7.45–7.35 (2 H, m, Ar***H***), 7.29 (1 H, d, *J* = 8.3, Ar***H***), 7.20–7.12 (4 H, m, Ar***H***), 6.76–6.68 (3 H, m, Ar***H***), 4.57–4.43 (2 H, m, OC***H***_***a***_H_b_), 4.43–4.30 (2 H, m, OCH_a_***H***_***b***_), 3.45 (8 H, br. s, NCH_2_); *δ*_C_ (126 MHz, CD_3_OD) 172.2, 169.8, 157.9, 156.0, 153.7, 137.7, 136.5, 136.4, 132.9, 132.8, 132.6, 132.2, 131.8, 131.6, 131.2, 131.1, 130.6, 129.4, 128.9, 125.1, 124.5, 123.8, 104.4, 88.0, 87.3, 64.4, 64.3, 64.3, 64.1; *m*/*z* (ESI) 663 (M^+^ + H, 30%) and 685 (M^+^ + Na, 30); UV-Vis (MeCN) lmax (*ε*/M^−1^ cm^−1^): 487 (13 200), 457 (18 800), 430 (16 700), 353 (9200), 227 (59 000) nm; fluorescence (MeCN; *λ*_ex_ = 531 nm); *λ*_em_ 545 nm.

### Rhodamine amide 2,2′-biphenyldioxacyclodecyne (25)


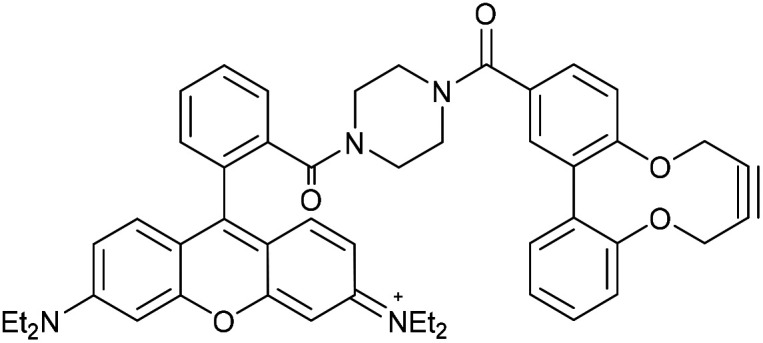
This compound is novel. *N*-(6-(Diethylamino)-9-(2-(piperazine-1-carbonyl)phenyl)-3*H*-xanthen-3-ylidene)-*N*-ethylethanaminium 27, synthesised as previously described,^12^ (100 mg, 0.195 mmol) and acid alkyne 28 (54.6 mg, 0.195 mmol) were dissolved in anhydrous DMF (2 mL). DMAP (59.5 mg, 0.287 mmol) and EDCI (74.9 mg, 0.390 mmol) were added and the reaction stirred under N_2_ at room temperature for 18 hours. H_2_O (10 mL) was added, extracted with CH_2_Cl_2_/IPA (4 : 1) (3 × 10 mL) and the combined organic layers dried over MgSO_4_. The crude mixture was purified by column chromatography (SiO_2_; CH_2_Cl_2_/MeOH; 100 : 0 → 90 : 10) to afford the compound as a dark purple solid (36 mg, 0.049 mmol, 24%).

*R*_f_ = 0.70 (4 : 1 DCM/MeOH); mp 169–170 (dec) °C; (found (ESI) [M + H]^+^, 773.3692. C_49_H_49_N_4_O_5_ requires [M + H]^+^, 773.3697); *ν*_max_ 1586, 1334, 1244, 1178, 1122, 1070, 1002 and 759 cm^−1^; *δ*_H_ (500 MHz, CD_3_OD) 7.90 (2 H, s, Ar***H***), 7.81–7.74 (2 H, m, Ar***H***), 7.70 (1 H, d, *J* = 6.8, Ar***H***), 7.54–7.49 (1 H, m, Ar***H***), 7.46–7.39 (2 H, m, Ar***H***), 7.32–7.25 (3 H, m, Ar***H***), 7.23–7.12 (5 H, m, Ar***H***), 7.05 (2 H, d, *J* = 9.7, Ar***H***), 6.95 (2 H, t, *J* = 3.1, Ar***H***), 4.55–4.43 (2 H, m, OC***H***_***a***_H_b_), 4.40–4.33 (2 H, m, OCH_a_***H***_***b***_), 3.66 (8 H, app. pent., *J* = 7.3, NC***H***_2_CH_3_), 3.58–3.37 (8 H, m, NC***H***_2_), 1.29 (12 H, app. q, *J* = 7.1, NCH_2_C***H***_3_); *δ*_C_ (126 MHz, CD_3_OD) 172.2, 169.6, 159.3, 157.9, 157.2, 157.1, 156.0, 137.7, 136.5, 136.4, 133.2, 132.8, 132.4, 132.2, 131.8, 131.6, 131.2, 130.7, 129.4, 128.9, 125.2, 124.6, 123.8, 114.9, 97.4, 88.0, 87.4, 64.4, 64.3, 46.9, 12.8. *m*/*z* (ESI) 773 (M^+^ + H, 100%); UV-Vis (MeCN) lmax (*ε*/M^−1^ cm^−1^): 558 (57 400), 522 (35 100), 352 (24 700), 522 (36 200), 250 (59 800) nm; fluorescence (MeCN; *λ*_ex_ = 566 nm); *λ*_em_ 578 nm.

### *N*-(2′-Hydroxy-[1,1′-biphenyl]-2-yl)-4-methylbenzenesulfonamide (31)


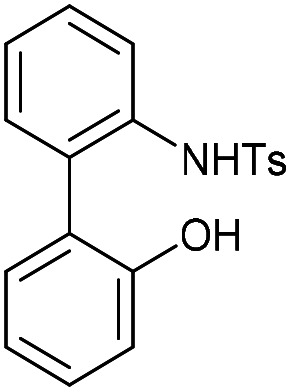
This compound is novel. A solution of 2-hydroxyphenyl boronic acid (175 mg, 1.27 mmol, 1.3 eq.), compound 30 (365 mg, 0.981 mmol, 1.0 eq.), K_2_CO_3_ (270 mg, 1.96 mmol, 2.0 eq.) and [PdCl_2_(PPh_3_)_2_] (68 mg, 0.098 mmol, 0.1 eq.) in 5 : 1 DMF-H_2_O (9 mL) was stirred at 80 °C for 12 h. The reaction was cooled to room temperature and then diluted with H_2_O (20 mL). The product was then extracted with EtOAc (3 × 20 mL) and the combined organic extracts were dried over MgSO_4_ before being concentrated. The crude product was then subjected to column chromatography (graduated eluent: 9 : 1 Hex/EtOAc–7 : 3 Hex/EtOAc) to give the pure product as a white solid (177 mg, 0.523 mmol, 53%).

*R*_f_ = 0.63 (2 : 3 EtOAc/DCM); mp = 142–146 °C; (found (ESI) [M + Na]^+^ 362.0821 C_16_H_17_NNaO_3_S requires 362.0821); *v*_max_ 3422, 3321, 3231, 1596, 1484, 1163, 700 and 527 cm^−1^; *δ*_H_ (CDCl_3_, 500 MHz) 7.74 (1 H, d, *J* = 8.0 Hz, Ar***H***), 7.42 (1 H, t, *J* = 8.1 Hz, Ar***H***), 7.36 (2 H, d, *J* = 8.1 Hz, 2 × Ar***H***), 7.22–7.28 (1 H, m, Ar***H***), 7.15 (1 H, d, *J* = 7.6 Hz, Ar***H***), 7.09 (2 H, d, *J* = 8.1 Hz, 2 × Ar***H***), 6.94 (2 H, d, *J* = 8.0 Hz, Ar***H***), 6.85 (1 H, t, *J* = 7.5 Hz, Ar***H***) 6.57 (1 H, d, *J* = 7.5 Hz, Ar***H***) 5.07–5.12 (1 H, br. s, N***H***) 2.39 (3 H, s, ArC***H***_3_) ppm; *δ*_C_ (CDCl_3_, 125 MHz) 151.8, 143.6, 135.9, 134.6, 131.0, 130.0, 130.0, 129.5, 129.3, 126.9, 126.0, 123.8, 121.3, 116.0, 21.5 ppm; *m*/*z* (ESI) 362.2 [M + Na]^+^.

### Compound 32


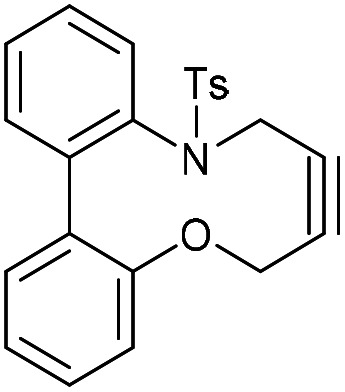
This compound is novel. A solution of 20 (116 mg, 0.294 mmol, 1.0 eq.), 30 (99.3 mg, 0.294 mmol, 1.0 eq.) and Cs_2_CO_3_ (383 mg, 1.18 mmol, 4.0 eq.) in CH_3_CN (15 mL) was stirred at room temperature for 2 weeks. The solvent was then removed under vacuum and the residue taken up in water (20 mL) the product was extracted with EtOAc (3 × 20 mL). The combined organic extracts were dried over MgSO_4_ and concentrated to give the crude product. This was then subjected to column chromatography (eluent: 1 : 1 Hex/EtOAc) to give the pure product as a white solid (46.6 mg, 0.12 mmol, 40%).

*R*_f_ = 0.82 (1 : 1 EtOAc/hexane); mp = 122–128 °C; (found (ESI) [M + Na]^+^ 412.0981 C_23_H_19_NNaO_3_S requires 412.0978); *v*_max_ 3059, 2922, 1596, 1501, 1452, 1106, 1056, 966, 688 and 575 cm^−1^; *δ*_H_ (CDCl_3_, 500 MHz) 7.72 (1 H, d, *J* = 7.5 Hz, Ar***H***), 7.65 (2 H, d, *J* = 8.2 Hz, 2 × Ar***H***), 7.28–7.37 (2 H, m, 2 × Ar***H***), 7.23–7.28 (3 H, m, 3 × Ar***H***), 7.17–7.23 (2 H, m, 2 × Ar***H***), 7.08 (1 H, d, *J* = 8.1 Hz, Ar***H***), 6.88 (1 H, d, *J* = 8.0 Hz, Ar***H***), 4.35 (1 H, d, *J* = 15.0 Hz, CH_a_***H***_b_) 4.18–4.25 (2 H, m, C***H***_2_) 3.50 (1H, d, *J* = 15.0 Hz, C***H***_a_H_b_), 2.39 (3 H, s, Ar–C***H***_3_) ppm; *δ*_C_ (CDCl_3_, 125 MHz) 154.3, 144.1, 142.1, 136.9, 135.8, 135.6, 133.1, 132.4, 129.8, 129.5, 128.4, 128.2, 128.2, 127.6, 124.3, 122.3, 84.9, 84.3, 63.0, 43.5, 21.6 ppm; *m*/*z* (ESI) 362.2 [M + Na]^+^.

### (3-Benzyl-14-tosyl-3,4,14,15-tetrahydrodibenzo[*b*,*d*][1,2,3]triazolo[4,5-*h*][1,6]oxazecine) and 32B (1-benzyl-14-tosyl-1,4,14,15-tetrahydrodibenzo-[*b*,*d*][1,2,3]triazolo[4,5-*h*][1,6]oxazecine) (32A)


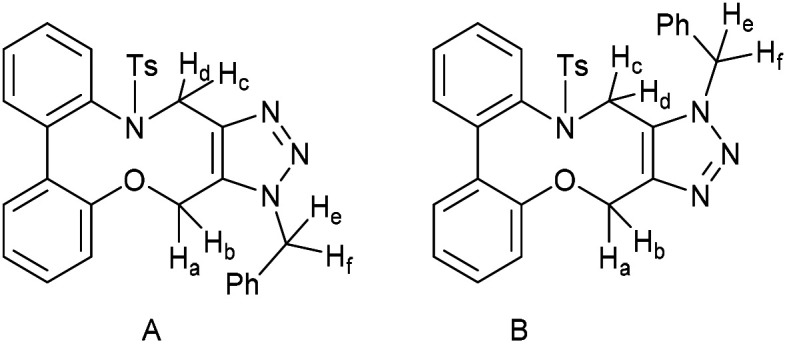
These compounds are novel. To a solution of compound 31 (20 mg, 0.051 mmol, 1.0 eq.) in CDCl_3_ (0.5 mL) was added benzyl azide (6.8 mg, 6.4 μL, 0.051 mmol, 1 eq.). The mixture was left undisturbed at room temperature and monitored by proton NMR until full conversion was observed. The chloroform was removed by evaporation and the residue purified by column chromatography (eluted with DCM) to provide the product as two isolable isomers A (white solid) (6.8 mg, 0.013 mmol, 26%) and B (white solid) (5.4 mg, 0.010 mmol, 20%). The stereochemical assignments are arbitrary.

A; *R*_f_ = 0.28 (DCM); (found (ESI) [M + Na]^+^ 545.1615 C_30_H_26_N_4_NaO_3_S requires 545.1618); *v*_max_ 3028, 2925, 2854, 1596, 1478, 1439, 1353, 1331, 1159 and 738 cm^−1^; *δ*_H_ (CDCl_3_, 500 MHz) 7.28–7.44 (7 H, m, Ar***H***), 7.13–7.20 (3 H, m, Ar***H***), 7.03 (2 H, d, *J* = 8.1 Hz, Ar***H***), 6.98–7.02 (1 H, m, Ar***H***), 6.91 (2 H, d, *J* = 8.1 Hz, Ar***H***), 6.88–6.90 (2 H, m, Ar***H***), 5.76 (1 H, d, *J* = 15.7 Hz, C***H***_***e***_H_f_), 5.64 (1 H, d, *J* = 15.7 Hz, CH_e_***H***_f_), 5.54 (1 H, d, *J* = 14.5 Hz, C***H***_*c*_CH_d_), 5.14 (1H, d, *J* = 14.5 Hz, CH_c_***H***_***d***_), 4.91 (1 H, d, *J* = 15.7 Hz C***H***_a_H_b_), 4.47 (1 H, d, *J* = 15.7 Hz, CH_a_***H***_b_), 2.39 (3 H, s, Ar–C***H***_3_) ppm; *δ*_C_ (CDCl_3_, 125 MHz) 154.4, 144.6, 143.9, 140.2, 140.0, 135.5, 134.8, 131.3, 131.2, 131.0, 130.1, 129.3, 129.2, 129.1, 128.7, 128.6, 128.5, 128.4, 127.8, 127.1, 121.8, 112.7, 62.0, 52.4, 44.4, 21.5 ppm; *m*/*z* (ESI) 523.3 [M + H]^+^ 545.2 [M + Na]^+^.

B; *R*_f_ = 0.1 (DCM); (found (ESI) [M + Na]^+^ 545.1615 C_30_H_26_N_4_NaO_3_S requires 545.1618); *v*_max_ 3028, 2925, 2854, 1596, 1478, 1439, 1353, 1331, 1159 and 738 cm^−1^; *δ*_H_ (CDCl_3_, 500 MHz) 7.33–7.40 (5 H, m, Ar***H***), 7.28–7.31 (2 H, m, Ar***H***),7.19–7.21 (2H, m, Ar***H***), 7.13–7.18 (4 H, m, Ar***H***), 7.03–7.08 (3 H, m, Ar***H***), 6.45 (1 H, m, Ar***H***), 5.65 (1 H, d, *J* = 15.7 Hz, C***H***_***c***_H_d_), 5.31 (1 H, d, *J* = 15.7 Hz, CH_c_***H***_d_) 5.05 (1 H, d, *J* = 13.7 Hz, C***H***_e_H_f_) 5.04 (1H, d, *J* = 14.8 Hz, C***H***_***a***_H_b_), 4.96 (1 H, d, *J* = 13.7 Hz CH_e_***H***_***f***_) 4.59 (1 H, d, *J* = 14.8 Hz, CH_a_***H***_b_) 2.43 (3 H, s, Ar–C***H***_3_) ppm; *δ*_C_ (CDCl_3_, 125 MHz) 155.4, 143.7, 142.4, 140.6, 139.4, 134.4, 134.0, 133.0, 132.0, 131.7, 131.1, 129.2, 129.2, 128.8, 128.7, 128.6, 128.4, 128.3, 127.2, 126.7, 123.0, 114.8, 60.0, 52.4, 47.2, 21.6 ppm; *m*/*z* (ESI) 523.3 [M + H]^+^ 545.2 [M + Na]^+^.

### 6,6-Dimethoxy-2,2′-biphenyldioxacyclodecyne (37) 


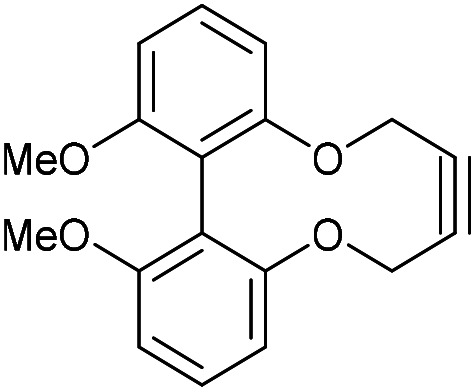
This compound is novel. To a stirring solution of compound 20 (145 mg, 0.368 mmol, 1 eq.) and compound 36^15^ (90 mg, 0.37 mmol, 1 eq.) in MeCN (20 mL) was added Cs_2_CO_3_ (475 mg, 1.46 mmol, 4 eq.). The mixture was stirred for 10 days at 50 °C and monitored by TLC. Solvents were removed under vacuum and residue was dissolved in H_2_O (30 mL). The aqueous layer was then extracted with EtOAc (3 × 30 mL) and the combined organic extracts were dried over MgSO_4_, which was removed by filtration. The solvents were removed under vacuum to give the crude product. The crude product was then purified by column chromatography (eluted with 0–25% EtOAc/heptane) to give the pure product as a white solid (10 mg, 0.034 mmol, 10%).

Mp = 134–136 °C; (found (ESI)) 297.1180 C_18_H_17_O_4_ requires 297.1127; *δ*_H_ (500 MHz, CDCl_3_) 7.37 (2 H, t, *J* = 8.2 Hz, Ar***H***) 6.78–6.87 (4 H, m, Ar***H***) 4.48–4.58 (2 H, m, C***H***_***a***_H_b_) 4.35–4.44 (2 H, m, CH_a_C***H***_***b***_) 3.76 (6 H, s, OC***H***_**3**_); *δ*_C_ (125 MHz, CDCl_3_) 158.1, 156.6, 129.3, 120.4, 114.0, 107.7, 87.2, 63.2, 56.3 ppm; *m*/*z* (ESI) 297.2 [M + Na]^+^.

### 1-Benzyl-9,10-dimethoxy-4,15-dihydro-1*H*-dibenzo[7,8:9,10][1,6]dioxecino[3,4-*d*][1,2,3]triazole (38)


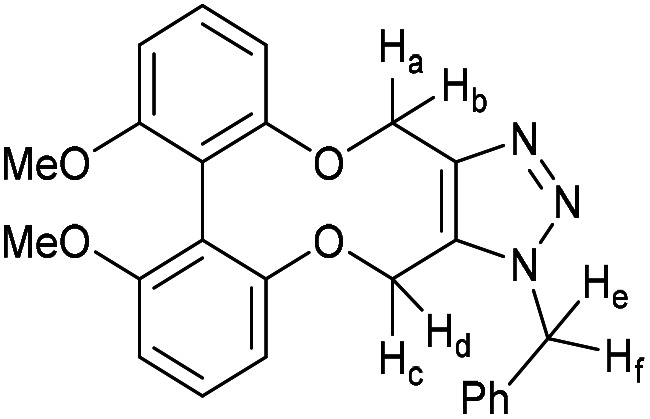
This compound is novel. To a solution of compound 37 (7.0 mg, 0.023 mmol, 1.0 eq.) in CDCl_3_ (0.5 mL) was added benzyl azide (3.1 mg, 0.023 mmol, 1.0 eq.). The reaction was monitored by proton NMR. Once the reaction was complete the solvent was evaporated to give the crude product which was purified by column chromatography (eluted 0–25% EtOAc : heptane) to give the pure product as a white solid (7.5 mg, 0.017 mmol, 85%).

(Found (ESI)) 452.1580 C_25_H_23_N_3_NaO_4_ requires 452.1581; *v*_max_ 2902, 2839, 1588, 1577, 1466, 1435, 1075 and 726 cm^−1^; *δ*_H_ (500 MHz, CDCl_3_) 7.33–7.36 (3 H, m, Ar***H***) 7.23 (1 H, t, *J* = 8.3 Hz, Ar***H***) 7.18 (1 H, t, *J* = 8.4 Hz, Ar***H***) 7.12–7.16 (2 H, m, Ar***H***) 6.83 (1 H, d, *J* = 8.3 Hz, Ar***H***) 6.71 (1 H, d, *J* = 8.3 Hz, Ar***H***) 6.63 (1 H, d, *J* = 8.4 Hz, Ar***H***) 6.46 (1 H, d, *J* = 8.3 Hz, Ar***H***) 5.73 (1 H, d, *J* = 15.9 Hz, C***H***_***e***_H_f_) 5.48 (1 H, d, *J* = 13.7 Hz, C***H***_***c***_H_d_) 5.41 (1 H, d, *J* = 15.9 Hz, CH_e_C***H***_***f***_) 5.29 (1 H, d, *J* = 13.7 Hz, CH_c_***H***_***d***_) 5.15 (1 H, d, *J* = 13.2 Hz, C***H***_***a***_H_b_) 4.96 (1 H, d, *J* = 13.2 Hz, CH_a_***H***_***b***_) 3.75 (3 H, s, OC***H***_**3**_) 3.72 (3 H, s, OC***H***_**3**_); *δ*_C_ (125 MHz, CDCl_3_) 158.5, 158.3, 158.0, 156.2, 144.7, 134.8, 132.1, 129.0, 128.9, 128.5, 127.0, 115.4, 114.0, 113.8, 109.1, 107.7, 106.4, 105.2, 63.2, 61.3, 56.0, 55.9, 52.3 ppm; *m*/*z* (ESI) 452.2 [M + Na]^+^.

### 6,7,8,9-Tetrahydro-1,14-(epoxyethanooxy)dibenzo[*b*,*d*][1,6]dioxecine (42)


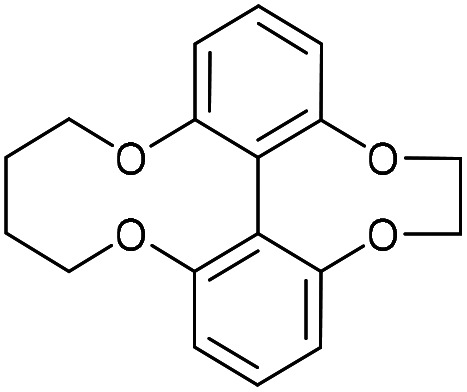
This compound is novel. A solution of compound 41 (357 mg, 1.47 mol, 1 eq.) and 1,4-dibromobutane (317 mg, 1.47 mmol, 1 eq.) in MeCN (15 mL) was added to a solution of C_2_CO_3_ (1.2 g, 3.7 mmol, 2.5 eq.) in MeCN (50 mL) at 60 °C over 5 h. The resulting solution was stirred for a further 12 h before the solvent was removed under vacuum. The residue was dissolved in H_2_O (30 mL) and the product was extracted with EtOAc (3 × 30 mL). The combined organic extracts were dried over MgSO_4_, which was removed by filtration, and then concentrated to give the crude product. Purification by column chromatography (eluted 25–100% EtOAc/hexane) gave the pure product as a white solid (352 mg, 1.18 mmol, 81%).

*R*_f_ = 0.53 (1 : 1 EtOAc/hexane); mp = 157–160 °C; (found (ESI)) 321.1094 C_18_H_18_NaO_4_ requires 321.1097; *v*_max_ 2928, 1590, 1563, 1257, 1221, 1065, 1023, 785 cm^−1^; *δ*_H_ (500 MHz CDCl_3_) 7.31 (2 H, t, *J* = 8.2 Hz, Ar***H***), 6.89 (2 H, d, *J* = 8.2 Hz, Ar***H***), 6.86 (2 H, d, *J* = 7.9 Hz, Ar***H***), 4.40 (2 H, d, *J* = 8.5 Hz, OC***H***_**2**_), 4.38–4.45 (2 H, m, OC***H***_**2**_), 4.25 (2 H, m, OC***H***_**2**_), 4.12 (2 H, d, *J* = 8.5 Hz, OC***H***_**2**_), 1.91–2.01 (2 H, m, C***H***_**2**_), 1.79–1.88 (2 H, m, C***H***_**2**_); *δ*_C_ (125 MHz, CDCl_3_) 160.3, 157.9, 129.3, 118.9, 115.6, 111.6, 74.0, 70.7, 26.7; *m*/*z* (ESI) 321.0 [M + Na]^+^.

### 6,7,8,9-Tetrahydrodibenzo[*b*,*d*][1,6]dioxecine-1,14-diol (43)


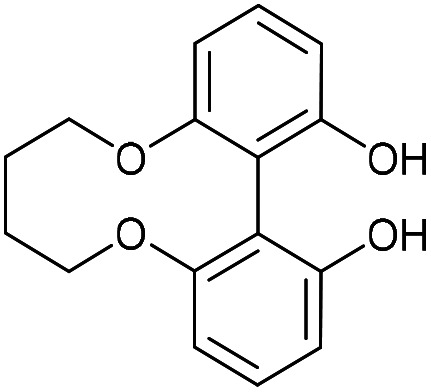
This compound is known and fully characterised.^23^ A solution of di-*tert*-butyl biphenyl (574 mg, 2.16 mmol, 8 eq.) in THF (11 mL) was cooled to 0 °C. To the solution, Li (13 mg, 1.89 mmol, 7 eq.) was added and the solution was stirred until dark blue in colour. This solution was then added to compound 42 (80 mg, 0.27 mmol, 1 eq.) and the mixture was stirred for 1 h. The mixture was then quenched with 1 M HCl (7 mL) and the product was extracted with EtOAc (3 × 10 mL). The combined organic extracts were then dried over MgSO_4_, which was removed by filtration, and the solution concentrated to give the crude product. Purification by column chromatography (eluted with 25–50% EtOAc : hexane) gave the pure product as a white solid (48.4 mg, 0.17 mmol, 66%).

*R*_f_ = 0.46 (1 : 1 EtOAc/hexane); *v*_max_ 3240, 2929, 1601, 1572, 1441, 1227, 1040, 775 cm^−1^; *δ*_H_ (500 MHz, CDCl_3_) 7.28 (2 H, t, *J* = 8.3 Hz, Ar***H***), 6.73 (2 H, d, *J* = 8.2 Hz, Ar***H***), 6.72 (2 H, d, *J* = 8.2 Hz, Ar***H***), 5.15 (2 H, s, O***H***), 4.27–4.36 (2 H, m, OC***H***_**2**_), 4.18–4.27 (2 H, m, OC***H***_**2**_), 1.83–1.96 (2 H, m, C***H***_**2**_), 1.70–1.83 (2 H, m, C***H***_**2**_); *δ*_C_ (125 MHz, CDCl_3_) 154.1, 130.1, 109.7, 108.6, 70.5, 26.0; *m*/*z* (ESI) 271.1 [M − H]^−^, 295.1 [M + H]^+^.

### 6,6′-Oxybutyloxy-linked-2,2′-biphenyldioxacyclodecyne (44)


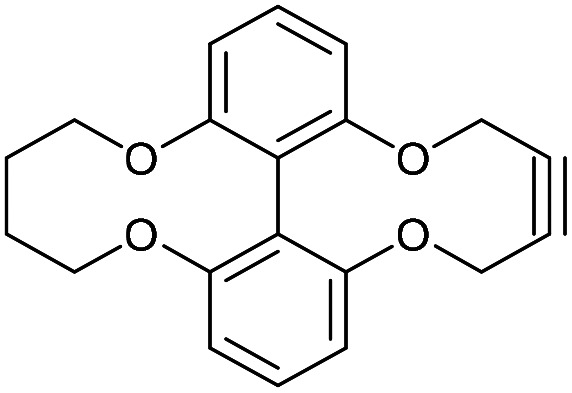
This compound is novel. A solution of compound 43 (127 mg, 0.467 mmol, 1.0 eq.) and compound 20 (184 mg, 0.467, 1.0 eq.) in MeCN (2.2 mL) was added to a solution of Cs_2_CO_3_ (336 mg, 1.03 mmol, 2.2 eq.) in MeCN (11 mL) at 60 °C over 5 h. The mixture was stirred for a further 12 h before the solvent was removed. The remaining residue was dissolved in H_2_O (20 mL) and extracted with EtOAc (3 × 20 mL). The combined organic extracts were dried over MgSO_4_, which was removed by filtration, and concentrated. The crude product was then purified by column chromatography (eluted with 25% EtOAc/hexane) to give the pure product as a white solid (56 mg, 0.17 mmol, 36%).

*R*_f_ = 0.20 (1 : 3 EtOAc/hexane); mp = 166–168 °C; (found (ESI)) 345.1088 C_20_H_18_NaO_4_ requires 345.1097; *v*_max_ 2953, 2922, 2852, 1569, 1457, 1251, 1218, 1031, 739 cm^−1^; *δ*_H_ (500 MHz, CDCl_3_) 7.37 (2 H, t, *J* = 8.2 Hz, Ar***H***), 7.00 (2 H, d, *J* = 8.2 Hz, Ar***H***), 6.94 (2 H, d, *J* = 8.2 Hz, Ar***H***), 4.54 (4 H, s, 2 × OC***H***_**2**_), 4.27 (2 H, d, *J* = 11.9 Hz, OC***H***_***a***_H_b_), 3.80 (2 H, t, *J* = 11.9 Hz, CH_a_***H***_***b***_), 1.34–1.53 (4 H, m, 2 × C***H***_**2**_); *δ*_C_ (125 MHz, CDCl_3_) 156.7, 156.4, 129.2, 125.8, 115.8, 115.2, 88.4, 73.0, 62.4, 23.9; *m*/*z* (ESI) 345.1 [M + Na]^+^.

### 15-Benzyl-5,6,7,8,15,18-hexahydro-14*H*-4,9,13,19-tetraoxa-15,16,17-triazadibenzo[hi,qr]cyclopenta[*d*]decalene (45)


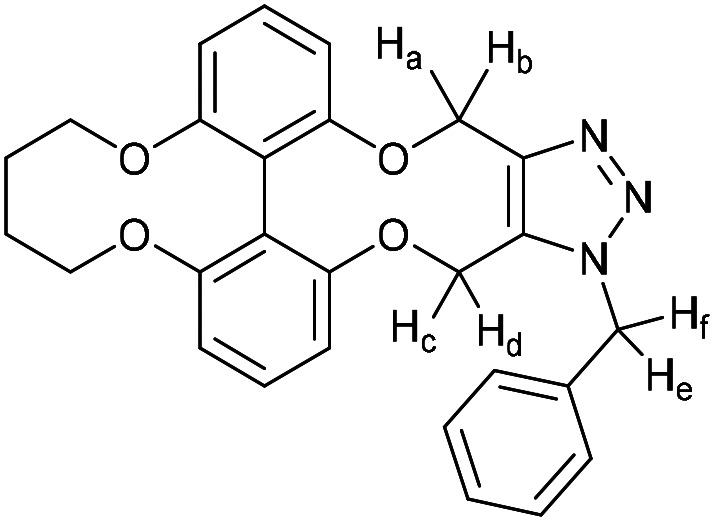
This compound is novel. To a solution of compound 44 (10 mg, 0.031 mmol, 1 eq.) in CDCl_3_ (0.6 mL) was added benzyl azide (4.1 mg, 0.031 mmol, 1 eq.). The reaction was monitored by NMR until completion after which the solvent was removed and the residue was purified by column chromatography (eluted with 10% EtOAc/hexane) to give the pure product as a white solid (13 mg, 0.028 mmol, 95%).

*R*_f_ = 0.34; (1 : 1 EtOAc/hexane); (found (ESI)) 478.1735 C_27_H_25_N_3_NaO_4_ requires 478.1737; *v*_max_ 2939, 1589, 1573, 1448, 1222, 1058, 716 cm^−1^; *δ*_H_ (500 MHz, CDCl_3_) 7.33–7.39 (3 H, m, Ar***H***), 7.21 (1 H, t, *J* = 8.2 Hz, Ar***H***), 7.15–7.19 (2 H, m, Ar***H***), 7.12 (1 H, t, *J* = 8.2 Hz, Ar***H***), 6.82 (1 H, d, *J* = 8.2 Hz, Ar***H***), 6.78 (1 H, d, *J* = 8.2 Hz, Ar***H***), 6.72 (1 H, d, *J* = 8.2 Hz, Ar***H***), 6.39 (1 H, d, *J* = 8.2 Hz, Ar***H***), 5.73 (1 H, d, *J* = 15.7 Hz, CH_f_***H***_***e***_), 5.47 (1 H, d, *J* = 13.6 Hz, C***H***_***c***_H_d_), 5.38 (1 H, d, *J* = 15.7 Hz, C***H***_***f***_H_e_), 5.30 (1 H, d, *J* = 13.6 Hz, CH_c_***H***_***d***_), 5.17 (1 H, d, *J* = 13.3 Hz, C***H***_***a***_H_b_), 5.03 (1 H, d, *J* = 13.3 Hz, CH_a_***H***_***b***_), 4.16–4.35 (4 H, m, 2 × OC***H***_**2**_), 1.70–1.94 (4 H, m, 2 × C***H***_**2**_); *δ*_C_ (125 MHz, CDCl_3_) 158.0, 157.8, 156.6, 144.9, 134.7, 132.3, 129.1, 128.7, 128.6, 128.5, 127.1, 116.9, 115.4, 110.9, 109.9, 108.9, 107.7, 70.8, 70.7, 62.8, 60.8, 52.3, 26.5; *m*/*z* (ESI) 478.2 [M + Na]^+^.

### 2,2′-Bis(methoxymethoxy)-6,6′-dinitro-1,1′-biphenyl (47)


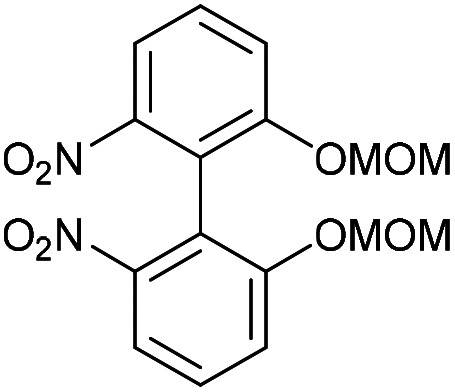
This compound is novel. Precursor 46 was prepared by the published method.^19^ It was purified by chromatography on silica gel using DCM as eluant. A solution of compound 46 (3.4 g, 11 mmol, 1 eq.)^19^ in dry DMF (100 mL) was added to Cu powder (2.8 g, 44 mmol, 4 eq.) and the mixture heated to 100 °C overnight. The mixture was cooled to room temperature and filtered to remove solid residues. The solids in the filter paper were washed through and EtOAc (2 × 50 mL). To the combined filtrates, water (100 mL) was added and the product was extracted with EtOAc (3 × 100 mL). The combined organic extracts were washed with H_2_O (3 × 100 mL) and brine (100 mL) before being dried over MgSO_4_, which was removed by filtration, and concentrated. The crude product was then purified by column chromatography (eluted with 0–25% EtOAc/pet. Ether 40 : 60) to give the pure product as a yellow solid (1.56 g, 4.34 mmol, 79%).

*R*_f_ = 0.54 (1 : 1 EtOAc/hexane); mp = 104–106 °C; (found (ESI)) 387.0797 C_16_H_16_N_2_NaO_8_ requires 387.0799; *v*_max_ 3075, 2959, 2831, 1580, 1531, 1456, 1253, 1205, 1083, 1001, 733 cm^−1^; *δ*_H_ (500 MHz, CDCl_3_) 7.79–7.85 (2 H, m, Ar***H***), 7.45–7.52 (4 H, m, Ar***H***), 5.01–5.06 (4 H, m, OC***H***_**2**_), 3.30 (6 H, s, OC***H***_**3**_); *δ*_C_ (125 MHz, CDCl_3_) 154.6, 149.1, 129.4, 119.5, 119.2, 117.7, 94.9, 56.1; *m*/*z* (ESI) 387.1 [M + Na]^+^. In another run a product was obtained in 92% yield without the need for purification by column chromatography.

### 6,6′-Dinitro-[1,1′-biphenyl]-2,2′-diol (48)


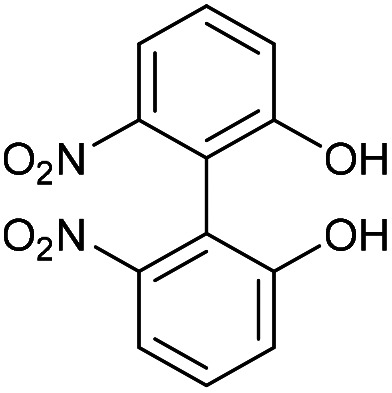
This compound is novel. To a solution of compound 47 (1.22 g, 3.35 mmol, 1 eq.) in MeOH (10 mL) was added conc. HCl (10 mL) dropwise. The mixture was stirred for 24 h and then H_2_O (30 mL) was added. The product was extracted with EtOAc (3 × 30 mL) and the combined organic extracts were dried over MgSO_4_, which was removed by filtration, and concentrated to give the crude product. Purification by column chromatography (eluted 50% EtOAc/pet.ether 40 : 60) gave the pure product (856 mg, 2.86 mmol, 81%).

*R*_f_ = 0.61 (1 : 1 EtOAc/Pet. Ether); mp = >200 °C (decomposition); (found (ESI)) 299.0269 C_12_H_8_N_2_NaO_6_ requires 299.0275; *v*_maz_ 3311, 1510, 1331, 1288, 1160, 1002, 733 cm^−1^; *δ*_H_ (500 MHz, CD_3_CN) 7.64 (2 H, dd, *J* = 8.2, 0.8 Hz, Ar***H***), 7.62 (2 H, br. s, O***H***), 7.45 (2 H, t, *J* = 8.2 Hz, Ar***H***), 7.23 (2 H, dd, *J* = 8.2, 0.8 Hz, Ar***H***); *δ*_C_ (125 MHz, CD_3_CN) 156.3, 151.1, 131.1, 121.8, 117.6, 117.3; *m*/*z* (ESI) 299.0 [M + Na]^+^ In an alternative workup the final product was purified by recrystallisation from MeOH in 88% yield.

### 6,6′-Dinitro-2,2′-biphenyldioxacyclodecyne (49)


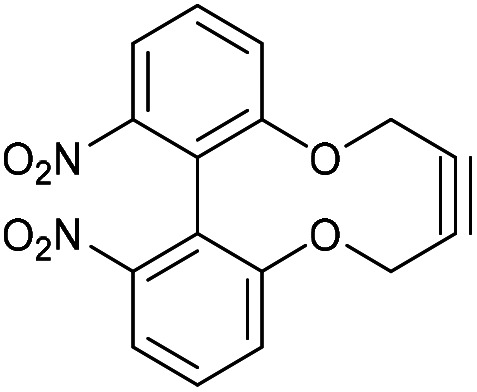
This compound is novel. A solution of compound 48 (856 mg, 3.10 mmol, 1.0 eq.) and compound 20 (1.22 g, 3.10 mmol, 1.0 eq.) in DMF (14 mL) was added to a solution of Cs_2_CO_3_ (2.50 g, 7.75 mmol, 2.5 eq.) in DMF (70 mL) at 60 °C over 5 h (using a syringe pump) and the resulting mixture was stirred for a further 12 h. The reaction mixture was then cooled to room temperature. Water (100 mL) was added and the product was extracted with EtOAc (3 × 100 mL). The combined organic extracts were then washed with H_2_O (2 × 50 mL) and brine (50 mL) and dried over MgSO_4_, which was removed by filtration, and concentrated. The water washings are required to remove DMF. The crude product was then purified by column chromatography (eluted with 50% EtOAc/pet. Ether 40 : 60) to give the pure product as a yellow solid (530 mg, 1.63 mmol, 53%).

*R*_f_ = 0.55 (1 : 1 EtOAc/hexane); mp 220–230 °C (dec); (found (ESI)) 349.0427 C_16_H_10_N_2_NaO_6_ requires 349.0431; *v*_max_ 3088, 2926, 2852, 1519, 1349, 1337, 990, 1238, 1175, 990, 735 cm^−1^; *δ*_H_ (500 MHz, CDCl_3_) 8.01 (2 H, d, *J* = 8.2 Hz, Ar***H***), 7.60 (2 H, t, *J* = 8.2 Hz, Ar***H***), 7.43 (2 H, d, *J* = 8.2 Hz, Ar***H***), 4.47–4.55 (2 H, m, OC***H***_***a***_H_b_), 4.37–4.47 (2 H, m, OCH_a_***H***_***b***_), ppm; *δ*_C_ (125 MHz, CDCl_3_) 155.2, 149.2, 129.9, 126.9, 126.5, 121.2, 87.4, 63.6 ppm; *m*/*z* (ESI) 349 [M + Na]^+^. In another procedure, chromatography on silica gel using DCM as eluant instead, and this gave a product in 39% yield.

### Benzyl azide cycloadduct of compound 49. 1-Benzyl-9,10-dinitro-4,15-dihydro-1*H*-dibenzo[7,8:9,10][1,6]dioxecino[3,4-*d*][1,2,3]triazole


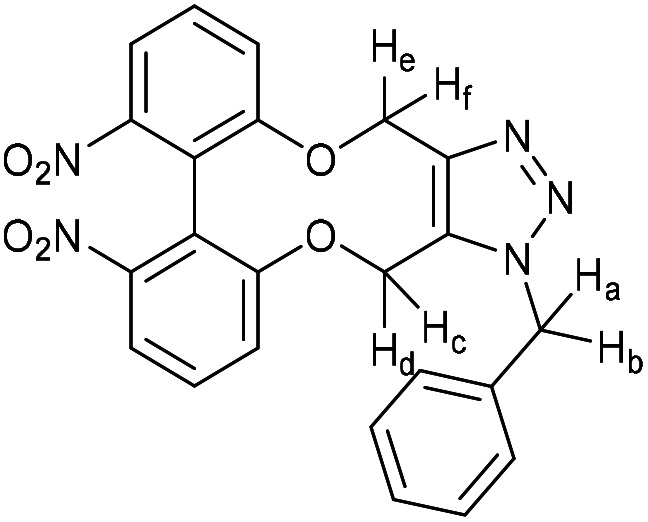
This compound is novel. To a solution of compound 49 (9.0 mg, 0.027 mmol, 1 eq.) in CDCl_3_ (0.5 mL) was added benzyl azide (3.6 mg, 0.027 mmol, 1 eq.). The reaction was monitored by proton NMR until completion and the solvent was removed under vacuum. The residue was purified by column chromatography (eluted with 50% EtOAc/hexane) to give the pure product as an oil (5.0 mg, 0.011 mmol, 40%).

*R*_f_ = 0.10 (1 : 1 EtOAc/hexane); (found (ESI)) 482.1067 C_23_H_17_N_5_NaO_6_ requires 482.1071; *v*_max_ 2923, 2853, 1521, 1346, 1266, 1182, 1076, 902, 722 cm^−1^; *δ*_H_ (500 MHz, CDCl_3_) 7.85–7.92 (1 H, m, Ar***H***), 7.76 (1 H, dd, *J* = 7.7, 1.6 Hz, Ar***H***), 7.39–7.46 (3 H, m, Ar***H***), 7.29–7.35 (3 H, m, Ar***H***), 7.09 (1 H, d, *J* = 8.1 Hz, Ar***H***), 7.01–7.07 (2 H, m, Ar***H***), 5.72 (1 H, d, *J* = 15.9 Hz, C***H***_a_H_b_), 5.58 (1 H, d, *J* = 13.9 Hz, OC***H***_***c***_H_d_), 5.44 (1 H, d, *J* = 15.9 Hz, CH_a_***H***_***b***_), 5.32 (1 H, d, *J* = 13.9 Hz, OCH_c_***H***_***d***_), 5.30 (1 H, d, *J* = 13.2 Hz, OC***H***_***e***_H_f_), 4.89 (1 H, d, *J* = 13.2 Hz, OCH_e_***H***_***f***_), ppm; (125 MHz, CDCl_3_) 157.9, 155.2, 148.8, 148.6, 143.5, 134.3, 130.8, 129.8, 129.4, 129.2, 128.8, 126.8, 122.2, 122.1, 120.7, 120.5, 119.6, 118.0, 63.9, 62.9, 52.5 ppm; *m*/*z* (ESI) 482.1 [M + Na]^+^.

### 6,6′-Diamino-2,2′-biphenyldioxacyclodecyne (50)


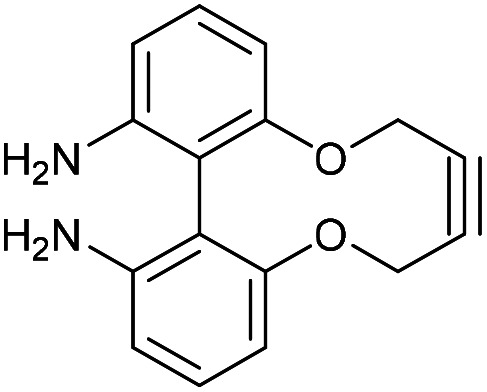
This compound is novel. To a solution of compound 49 (238 mg, 0.730 mmol, 1 eq.) in 6 : 1 EtOH/H_2_O (3.0 mL) was added NH_4_Cl (39 mg, 0.73 mmol, 1 eq.) and Fe powder (204 mg, 3.65 mmol, 5 eq.). The mixture was heated to 70 °C and stirred for 1 h. The solution was allowed to cool to rt then the crude reaction was filtered through filter paper using MeOH (4 × 30 mL). Solvent was removed, then the residue was filtered through cotton wool using DCM (4 × 30 mL). Removal of the solvent gave 50 as an amorphous solid (179 mg, 0.673 mmol, 92%) without the need for column chromatography.

*R*_f_ = 0.38 (1 : 1 EtOAc/hexane); (found (ESI)) 289.0946 C_16_H_14_N_2_NaO_2_ requires 289.0947; *v*_max_ 3465, 3360, 2960, 2914, 2864, 1611, 1565, 1461, 1302, 1248, 1116, 1020, 920, 729 cm^−1^; *δ*_H_ (500 MHz, CDCl_3_) 7.21 (2 H, t, *J* = 8.1 Hz, Ar***H***), 6.65 (2 H, d, *J* = 8.1 Hz, Ar***H***), 6.61 (2 H, d, *J* = 8.1 Hz, Ar***H***), 4.54–4.61 (2 H, m, C***H***_***a***_H_b_), 4.40–4.47 (2 H, m, CH_a_***H***_***b***_), 3.63 (4 H, br. s., N***H***_**2**_) ppm; *δ*_C_ (125 MHz, CDCl_3_) 156.2, 146.2, 129.9, 116.5, 112.3, 111.8, 87.2, 63.3 ppm; *m*/*z* (ESI) 267.1 [M + H]^+^, 289.1 [M + Na]^+^.

### Benzyl azide cycloadduct of compound 50. 1-Benzyl-4,15-dihydro-1*H*-dibenzo[7,8:9,10][1,6]dioxecino[3,4-*d*][1,2,3]triazole-9,10-diamine


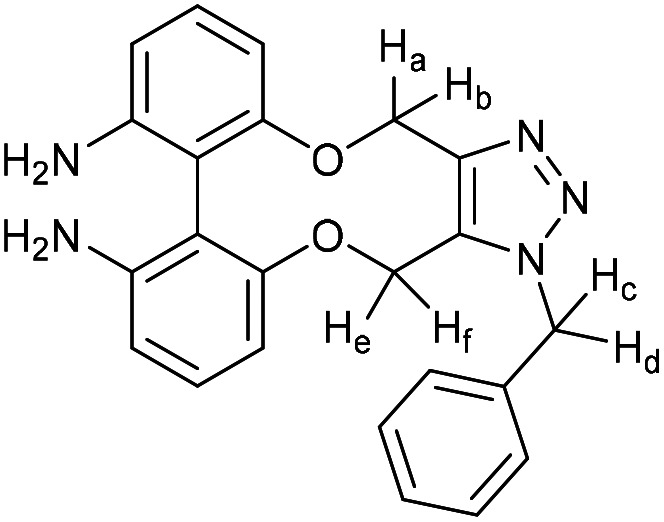
This compound is novel. To a solution of compound 50 (3.0 mg, 0.011 mmol, 1 eq.) in CDCl_3_ (0.5 mL) was added benzyl azide (1.5 mg, 0.011 mmol, 1 eq.). The reaction was monitored by proton NMR until completion, at which point the solvent was removed under vacuum. The crude material was purified by column chromatography (eluted with 25%–100% EtOAc/pet. Ether 40 : 60) to give the pure product as a white waxy solid (4 mg, 0.01 mmol, 91%).

*R*_f_ = 0.30 (1 : 1 EtOAc/hexane); (found (ESI)) 422.1590 C_23_H_21_N_5_NaO_2_ requires 422.1587; *v*_max_ 3450, 3356, 2926, 2854, 1615, 1574, 1456, 1231, 1072, 909, 724 cm^−1^; *δ*_H_ (500 MHz, CDCl_3_) 7.32–7.38 (3 H, m. Ar***H***), 7.11–7.16 (2 H, m, Ar***H***), 7.08 (1 H, t, *J* = 8.0 Hz, Ar***H***), 7.01 (1 H, t, *J* = 8.0 Hz, Ar***H***), 6.60 (1 H, d, *J* = 8.1 Hz, Ar***H***), 6.50 (1 H, d, *J* = 8.0 Hz, Ar***H***), 6.42 (1 H, d, *J* = 8.1 Hz, Ar***H***), 6.22 (1 H, d, *J* = 8.0 Hz, Ar***H***), 5.73 (1 H, d, *J* = 15.8 Hz, C***H***_***c***_H_d_), 5.43 (1 H, d, *J* = 13.6 Hz, C***H***_***a***_H_b_), 5.42 (1 H, d, *J* = 15.8 Hz, CH_c_***H***_***d***_), 5.28 (1 H, d, *J* = 13.6 Hz, CH_a_***H***_b_), 5.12 (1 H, d, *J* = 13.3 Hz, C***H***_***e***_H_f_), 4.94 (1 H, d, *J* = 13.3 Hz, CH_e_***H***_***f***_), 3.74 (4 H, br. s., N***H***_**2**_); *δ*_C_ (125 MHz, CDCl_3_) 158.7, 157.0, 145.6, 145.4, 144.8, 134.8, 132.1, 129.5, 129.1, 128.5, 127.0, 111.7, 110.6, 110.3, 109.6, 106.5, 105.4, 63.3, 61.2, 52.3 ppm; *m*/*z* (ESI) 398.1 [M − H]^−^.

### 6,6′-Bis(tosylamido)-2,2′-biphenyldioxacyclodecyne (51)


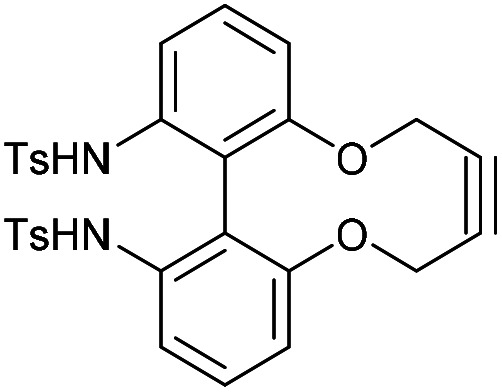
This compound is novel. To a solution of compound 50 (61 mg, 0.23 mmol, 1 eq.) and toluenesulfonyl chloride (109 mg, 0.58 mmol, 2.5 eq.) in DCM (1.7 mL) was added pyridine (45 mg, 0.58 mmol, 2.5 eq.). The reaction was stirred for 4 h at room temperature. H_2_O was added (10 mL) and the product was extracted with EtOAc (3 × 10 mL) and the combined organic extracts were dried over MgSO_4_, which was removed by filtration, and concentrated. The product was a white solid (96 mg, 0.16 mmol, 73%) which was taken forward without further purification.

*R*_f_ = 0.51 (1 : 1 EtOAc/Pet. Ether); (found (ESI)) 597.1114 C_30_H_26_N_2_NaO_6_S_2_ requires 597.1124; *v*_max_ 3365, 2917, 1596, 1576, 1454, 1378, 1321, 1212, 1157, 1029, 1004, 935, 658, 536 cm^−1^; *δ*_H_ (500 MHz, CDCl_3_) 7.51 (4 H, d, *J* = 8.1 Hz, Ar***H***), 7.51 (2 H, d, *J* = 7.9 Hz, Ar***H***), 7.38 (2 H, t, *J* = 8.1 Hz, Ar***H***), 7.19 (4 H, d, *J* = 8.1 Hz, Ar***H***), 6.90 (2 H, d, *J* = 7.9 Hz, Ar***H***), 5.95 (2 H, br. s, N***H***), 4.18–4.26 (2 H, mC***H***_***a***_H_b_), 4.08–4.18 (2 H, m, CH_a_***H***_***b***_); *δ*_C_ (125 MHz, CDCl_3_) 154.4, 144.3, 136.4, 135.8, 131.0, 129.6, 127.3, 120.7, 118.6, 117.6, 86.7, 63.3, 21.6 ppm; *m*/*z* (ESI) 597.1 [M + Na]^+^.

### 6,6′-Bis(dansylamido)-2,2′-biphenyldioxacyclodecyne (52)


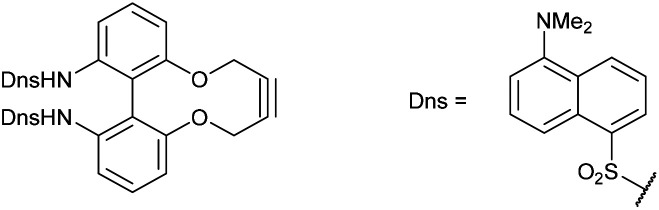
This compound is novel. To a solution of compound 50 (50 mg, 0.19 mmol, 1 eq.) and dansyl chloride (128 mg, 0.48 mmol, 2.5 eq.) in DCM (1.4 mL) was added pyridine (37.5 mg, 0.48 mmol, 2.5 eq.). The reaction was stirred for 4 h before H_2_O (10 mL) was added and the product was extracted with EtOAc (3 × 10 mL). The combined organic extracts were then dried over MgSO_4_ and concentrated to give the crude product. Purification by column chromatography (eluted with 25–50% EtOAc/pet.ether 40 : 60) gave the pure product as a yellow solid (22.6 mg, 0.0310 mmol, 16%).

*R*_f_ = 0.37 (1 : 1 EtOAc/hexane); (found (ESI)) 733.2153 C_40_H_37_N_4_O_6_S_2_ requires 733.2149; *v*_max_ 3356, 2939, 1572, 1453, 1321, 1144, 1032, 786, 622, 566 cm^−1^; *δ*_H_ (400 MHz, CDCl_3_) 8.47 (2 H, d, *J* = 8.5 Hz, Ar***H***), 8.03–8.10 (2 H, m, Ar***H***), 7.96 (2 H, d, *J* = 8.7 Hz, Ar***H***), 7.35–7.43 (4 H, m, Ar***H***), 7.20–7.24 (2 H, m, Ar***H***), 7.16 (4 H, t, *J* = 7.9 Hz, Ar***H***), 6.64 (2 H, dd, *J* = 8.0, 1.0 Hz, Ar***H***), 6.20 (2 H, br. s, N***H***), 3.98–4.09 (4 H, m, OC***H***_2_), 2.85–2.92 (12 H, s, NC***H***_**3**_); *δ*_C_ (125 MHz, CDCl_3_) 154.4, 151.7, 136.5, 135.2, 130.6, 130.4, 129.8, 129.5, 128.9, 128.21, 123.1, 121.7, 118.9, 118.7, 118.7, 115.1, 86.7, 63.1, 45.4 ppm; *m*/*z* (ESI) 733.2 [M + H]^+^, 755.2 [M + Na]^+^.

### 6,6-Bis(mesylamido)-2,2′-biphenyldioxacyclodecyne (53)


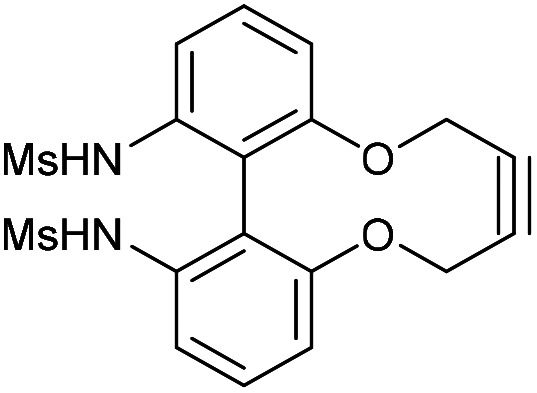
This compound is novel. To a stirred solution of compound 50 (69 mg, 0.259 mmol, 1.0 eq.) in anhydrous DCM (1.9 mL) was added mesyl chloride (0.1 mL, 148 mg, 1.29 mmol, >5 eq.) and pyridine (0.1 mL, 98 mg, 1.83 mmol, >5 eq.). The solution was degassed, then stirred at room temperature for six hours. Water (10 mL) was added, then the product was extracted with ethyl acetate (3 × 10 mL). The combined organic extracts were dried over MgSO_4_, filtered and concentrated under reduced pressure. The crude product was purified by column chromatography (eluent: DCM – 5 : 1 DCM/MeOH) to yield the pure product as a white solid (73 mg, 0.173 mmol, 70%). *R*_f_ 0.55 (99 : 1 DCM/MeOH); m.p. > 200 °C; found (ESI-TOF) 445.0494, [M + Na]^+^ calcd for C_18_H_18_N_2_O_6_S_2_Na 445.0498; *ν*_max_ 3271, 2355, 2330, 1578, 1454, 1356, 1323, 1216, 1154, 1014, 965, 745, 524 cm^−1^; *δ*_H_ (500 MHz, CDCl_3_) 7.61 (2H, d, *J* = 8.0, Ar***H***), 7.52 (2H, t, *J* = 8.0, Ar***H***), 7.08 (2H, d, *J* = 8.0, Ar***H***), 6.14 (2H, br. s, –N***H***–), 4.55–4.42 (4H, m, –OC***H***_2_–), 2.89 (6H, s, –SO_2_C***H***_3_); *δ*_C_ (125 MHz, CDCl_3_) 154.9, 136.8, 131.7, 121.8, 119.5, 118.4, 86.9, 63.8, 39.7; *m*/*z* (ESI) 445.0 [M + Na]^+^.

### 6,6′-Bis(tosylamide)-2,2′-biphenyldioxacyclodecyne butyl bridged derivative (54)


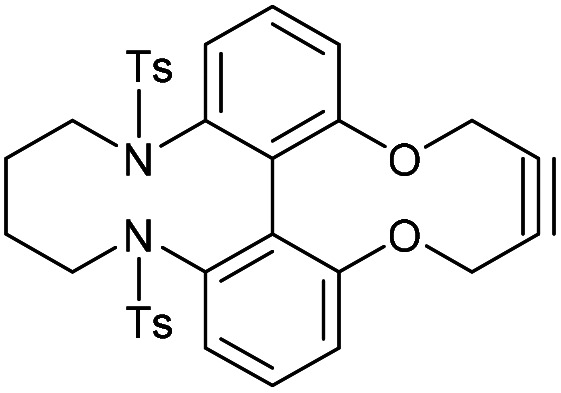
This compound is novel. A solution of 1,4-dibromobutane (34.5 mg, 0.160 mmol, 1 eq.) and compound 51 (96 mg, 0.160 mmol, 1 eq.) in MeCN (1.6 mL) was added to a mixture of Ca_2_CO_3_ (130 mg, 0.400 mmol, 2.5 eq.) in MeCN (6 mL) at 75 °C over 5 h. The reaction was stirred for a further 12 h before the solvent was removed under vacuum and H_2_O (20 mL) was added. The product was extracted with EtOAc (3 × 20 mL) and the combined organic extracts were dried over MgSO_4_, which was removed by filtration, and concentrated. The crude material was then purified by column chromatography (eluted with 50% EtOAc/pet. ether 40 : 60) to give the pure product as an oil (43 mg, 0.068 mmol, 44%).

*R*_f_ = 0.48 (1 : 1 EtOAc/hexane); (found (ESI)) 651.1586 C_34_H_32_N_2_NaO_6_S_2_ requires 651.1594; *v*_max_ 3026, 2924, 2872, 1592, 1453, 1371, 1125, 1041, 813, 684, 571, 555 cm^−1^; *δ*_H_ (500 MHz, CDCl_3_) 7.62 (4 H, d, *J* = 8.2 Hz, Ar***H***), 7.49 (2 H, t, *J* = 7.6 Hz, Ar***H***), 7.31 (2 H, d, *J* = 7.6 Hz, Ar***H***), 7.21 (2 H, d, *J* = 7.6 Hz, Ar***H***), 7.16 (4 H, d, *J* = 8.2 Hz, Ar***H***), 4.58–4.69 (4 H, m, 2 × OC***H***_**2**_), 3.06–3.13 (2 H, m, NC***H***_**2**_), 2.95–3.02 (2 H, m, NC***H***_**2**_), 2.38 (6 H, s, 2 × C***H***_3_), 1.00–1.12 (2 H, m, C***H***_**2**_), 0.84–0.98 (2 H, m, C***H***_**2**_); *δ*_C_ (125 MHz, CDCl_3_) 158.0, 143.5, 141.1, 135.9, 132.8, 123.0, 129.2, 128.5, 124.5, 121.1, 89.8, 62.1, 52.8, 22.1, 21.5 ppm; *m*/*z* (ESI) 629.2 [M + H]^+^, 651.2 [M + Na]^+^.

### 6,6′-Bis(mesylamide)-2,2′-biphenyldioxacyclodecyne butyl bridged derivative (56)


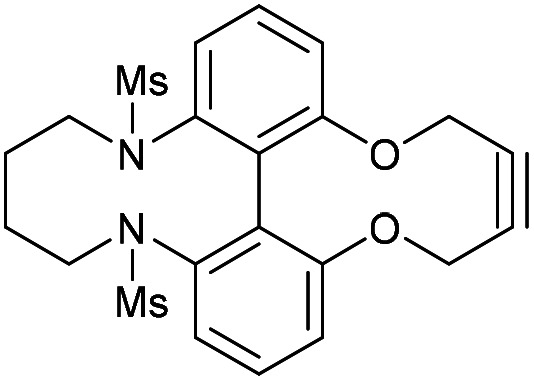
This compound is novel. To a solution of caesium carbonate (147 mg, 0.45 mmol, 2.5 eq.) in dry acetonitrile (6.8 mL), stirred and heated at 75 °C was added dropwise a solution of compound 53 (76 mg, 0.18 mmol, 1.0 eq.) and 1,4-dibromobutane (65 μL, 0.54 mmol, 3.0 eq.) in acetonitrile (20 mL) over 24 hours. The solution was then stirred for a further 24 hours, before being cooled to room temperature. Solvents were removed *in vacuo*. Water (20 mL) was added, and the product was extracted with ethyl acetate (3 × 20 mL). The combined organic layers were dried over MgSO_4_, filtered and concentrated under reduced pressure. The resulting crude product was purified by column chromatography (eluent: DCM/MeOH gradient) to afford the pure product as a brown solid (53 mg, 0.11 mmol, 62%). *R*_f_: 0.28 (29 : 1 DCM/MeOH); m.p. 131–132 °C (dec.); found 499.0959 (ESI-TOF) *m*/*z*: [M + Na]^+^ calcd for C_22_H_24_N_2_O_6_S_2_Na 499.0968; *ν*_max_ 3034, 2932, 2871, 2331, 1723, 1573, 1449, 1322, 1150, 1047, 969, 935, 894, 748, 664, 529; *δ*_H_ (500 MHz, CDCl_3_) 7.49 (2H, t, *J* = 8.0, Ar***H***), 7.29–7.27 (2H, m, Ar***H***) 7.19 (2H, d, *J* = 8.0, Ar***H***), 4.57–4.45 (4H, m, –OC***H***_2_), 3.33–3.26 (4H, m, MsNC***H***_2_–), 2.89 (6H, s, –SO_2_C***H***_3_) 1.33–1.09 (4H, m, MsNCH_2_C***H***_2_–); *δ*_C_ (125 MHz, CDCl_3_) 156.4, 141.0, 133.0, 130.4, 126.9, 121.5, 88.3, 62.5, 52.3, 40.3, 23.7; *m*/*z* (ESI) [M + Na]^+^ 499.0.

### *N*,*N*’-(1-Benzyl-4,15-dihydro-1*H*-dibenzo[7,8:9,10][1,6]dioxecino[3,4-*d*][1,2,3]triazole-9,10-diyl)bis(4-methylbenzenesulfonamide) (57)


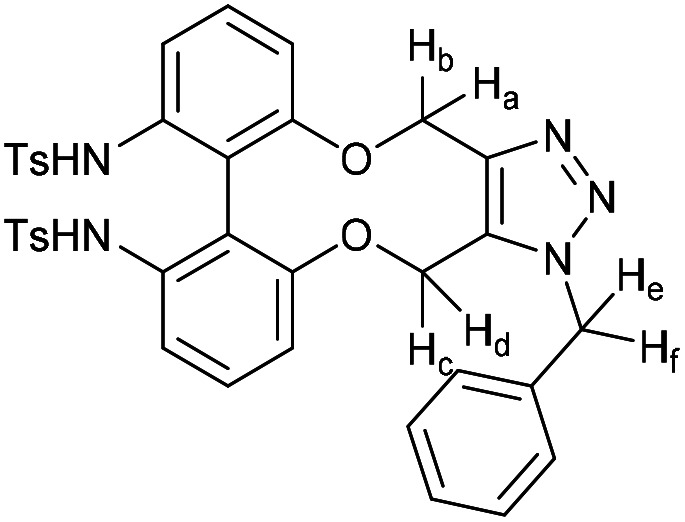
This compound is novel. To a solution of compound 51 (28.8 mg, 0.050 mmol, 1 eq.) in CDCl_3_ (0.5 mL) was added benzyl azide (6.7 mg, 0.050 mmol, 1 eq.). The reaction was monitored by proton NMR until completion. The solvent was removed under vacuum and the crude material was purified by column chromatography (eluted with 50–100% EtOAc/hexane) to give the pure product (12.1 mg, 0.017 mmol, 34%).

*R*_f_ = 0.27 (1 : 1 EtOAc/hexane); (found (ESI)) 730.1770 C_37_H_33_N_5_NaO_6_S_2_ requires 730.1764; *v*_max_ 3337, 3059, 2925, 2854, 1596, 1455, 1288, 1043, 728, 553 cm^−1^; *δ*_H_ (500 MHz, CDCl_3_) 7.70 (2 H, d, *J* = 8.1 Hz, Ar***H***), 7.54 (2 H, d, *J* = 8.1 Hz, Ar***H***), 7.31–7.35 (3 H, m, Ar***H***), 7.23–7.29 (4 H, m, Ar***H***), 7.14–7.22 (3 H, m, Ar***H***), 7.10 (1 H, d, *J* = 8.2 Hz, Ar***H***), 7.03 (2 H, m, *J* = 5.0 Hz, Ar***H***), 6.77 (1 H, d, *J* = 8.2 Hz, Ar***H***), 6.50 (1 H, d, *J* = 8.1 Hz, Ar***H***), 6.18 (2 H, br. s, N***H***), 5.62 (1 H, d, *J* = 15.8 Hz, C***H***_***e***_H_f_), 5.41 (1 H, d, *J* = 15.8 Hz, CH_e_***H***_***f***_), 5.17 (1 H, d, *J* = 13.6 Hz, C***H***_***a***_H_b_), 5.01 (1 H, d, *J* = 13.6 Hz, CH_a_***H***_***b***_), 4.93 (1 H, d, *J* = 13.4 Hz, C***H***_***c***_H_d_), 4.68 (1 H, d, *J* = 13.4 Hz, CH_c_***H***_***d***_); *δ*_C_ (125 MHz, CDCl_3_) 158.1, 156.7, 144.3, 144.1, 144.0, 136.2, 136.1, 136.1, 136.0, 134.4, 131.3, 130.8, 130.8, 129.9, 129.7, 129.2, 128.7, 127.5, 127.4, 126.9, 115.6, 114.9, 113.7, 113.7, 112.6, 112.1, 63.9, 61.5, 52.3, 21.7, 21.6 ppm; *m*/*z* (ESI)706.2 [M − H]^−^.

### *N*,*N*’-(1-Benzyl-4,15-dihydro-1*H*-dibenzo[7,8:9,10][1,6]dioxecino[3,4-*d*][1,2,3]triazole-9,10-diyl)dimethanesulfonamide (59)


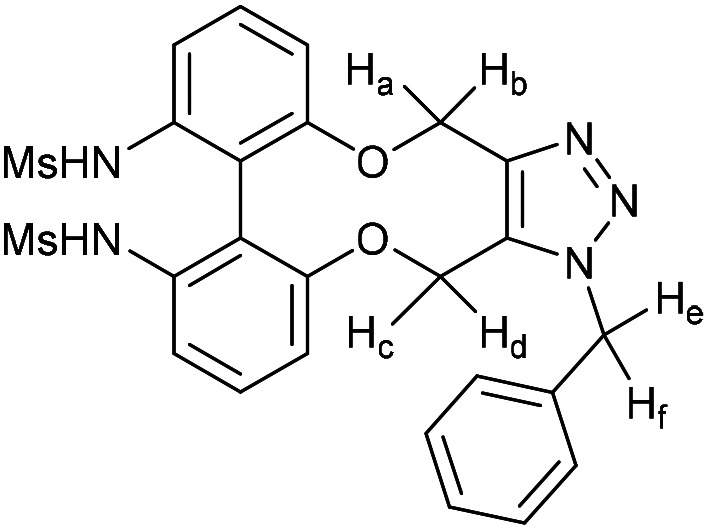
This compound is novel. Compound 53 (10 mg, 23.7 μmol, 1.0 eq.) was dissolved in CDCl_3_ (0.6 mL) to which was added benzyl azide (3.1 μL, 24.8 μmol, 1.0 eq.). After 9 days, the reaction was deemed complete; solvents were removed *in vacuo* and the residue was purified by column chromatography (eluent: DCM/MeOH gradient) to yield the pure product as a yellow-brown solid (20 mg, since the yield is >100%, an NMR-silent impurity is likely present). *R*_f_: 0.20 (99 : 1 DCM/MeOH); found 578.1136 (ESI-TOF) *m*/*z*: [M + Na]^+^ calcd for C_25_H_25_N_5_O_6_S_2_Na 578.1138; *δ*_H_ (500 MHz, CDCl_3_) 7.42–7.30 (9H, m, Ar***H***), 7.11–7.09 (2H, m, Ar***H***), 6.15 (1H, s, –N***H***–), 6.04 (1H, s, –N***H***–), 5.71 (1H, d, *J* = 15.5, PhC***H***_e_H_f_–), 5.49 (1H, d, *J* = 16.0, PhH_e_***H***_f_–), 5.46 (1H, d, *J* = 14.0, –OC***H***_a_H_b_–), 5.32 (1H, d, *J* = 14.0, –OCH_a_***H***_b_–), 5.18 (1H, d, *J* = 13.5, –OC***H***_c_H_d_–), 4.94 (1H, d, *J* = 13.0, –OCH_c_***H***_d_–); *δ*_C_ (125 MHz, CDCl_3_); 158.4, 157.0, 144.0, 136.4, 136.1, 134.4, 131.7, 131.4, 129.5, 129.3, 128.8, 119.5, 115.7, 114.7, 114.4, 113.8, 113.7, 112.7, 64.2, 61.8, 52.5, 40.3, 40.2; *m*/*z* (ESI) [M + Na]^+^ 578.1.

### 15-Benzyl-4,9-ditosyl-4,5,6,7,8,9,15,18-octahydro-14*H*-13,19-dioxa-4,9,15,16,17-pentaazadibenzo[hi,qr]cyclopenta-[d]decalene (60)


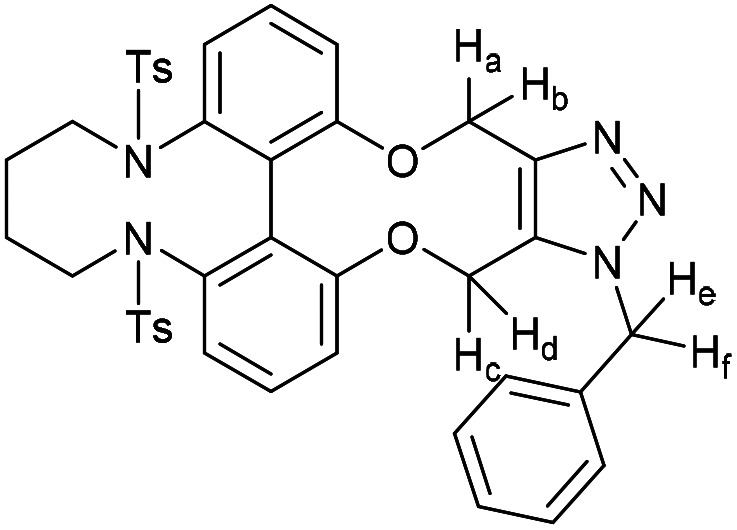
This compound is novel. To a solution of compound 54 (12.5 mg, 0.020 mmol, 1 eq.) in CDCl_3_ (0.5 mL) was added benzyl azide (2.7 mg, 0.020 mmol, 1 eq.). The reaction was monitored by proton NMR until completion. The solvents were removed under vacuum and the crude material was purified by column chromatography (eluted with 50% EtOAc/pet. ether 40 : 60) to give the pure product as a colourless oil (9.2 mg, 0.12 mmol, 60%).

*R*_f_ = 0.15 (1 : 19 MeOH/DCM); (found (ESI)) 784.2222 C_41_H_39_N_5_NaO_6_S_2_ requires 784.2234; *v*_max_ 2950, 1452, 1348, 1163, 1068, 728, 694 cm^−1^; *δ*_H_ (500 MHz, CDCl_3_) 7.30–7.40 (5 H, m, Ar***H***), 7.22–7.30 (5 H, m, Ar***H***), 7.10–7.20 (6 H, m, Ar***H***), 6.88 (1 H, d, *J* = 7.9 Hz, Ar***H***), 6.50 (1 H, d, *J* = 8.1 Hz, Ar***H***), 6.20 (1 H, d, *J* = 7.8 Hz, Ar***H***), 5.76 (1 H, d, *J* = 15.4 Hz, C***H***_***e***_H_f_), 5.65 (1 H, d, *J* = 13.4 Hz, OC***H***_***c***_H_d_), 5.52 (1 H, d, *J* = 15.4 Hz, CH_***e***_***H***_***f***_), 5.42 (1 H, d, *J* = 13.4 Hz, OCH_c_***H***_***d***_), 5.37 (1 H, d, *J* = 12.5 Hz, OC***H***_***a***_H_b_), 5.12 (1 H, d, *J* = 12.5 Hz, OCH_a_***H***_*b*_), 3.24–3.56 (4 H, m, 2 × NC***H***_**2**_), 2.41 (6 H, s, 2 × C***H***_**3**_), 1.77–1.97 (2 H, m, C***H***_2_), 1.55–1.77 (2 H, m, C***H***_2_); *δ*_C_ (125 MHz, CDCl_3_) 159.7, 157.4, 145.0, 143.5, 143.5, 140.4, 140.1, 134.8, 133.9, 133.3, 132.3, 130.1, 129.3, 129.1, 129.1, 128.7, 128.6, 128.6, 128.5, 128.3, 127.2, 120.5, 118.6, 115.9, 113.6, 62.8, 61.6, 51.1, 51.0, 24.7, 24.3, 21.6 ppm; *m*/*z* (ESI) 784.2 [M + Na]^+^.

### 15-Benzyl-4,9-bis(methylsulfonyl)-4,5,6,7,8,9,15,18-octahydro-14*H*-13,19-dioxa-4,9,15,16,17-pentaazadibenzo-[hi,qr]cyclopenta[*d*]decalene (61) 


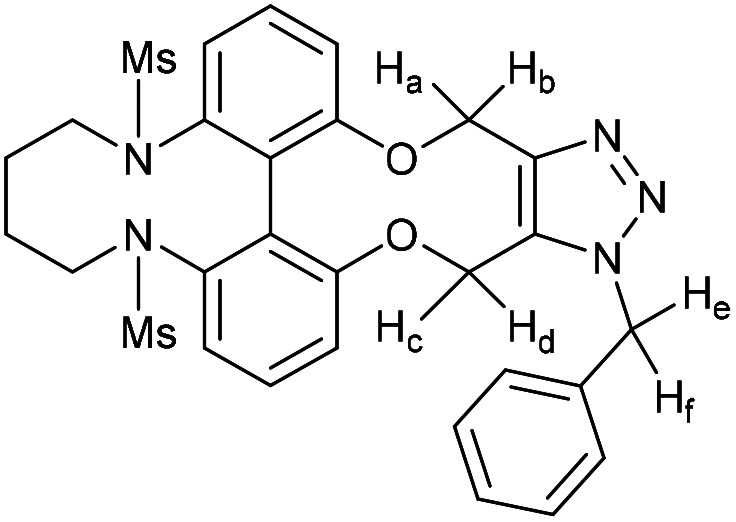
This compound is novel. Compound 56 (10.4 mg, 21.8 μmol, 1.0 eq.) was dissolved in CDCl_3_ (0.6 mL), to which was added benzyl azide (2.72 μL, 21.8 μmol, 1.0 eq.). After 28 hours, the reaction was deemed complete, and solvents were removed *in vacuo*. The reaction was performed again with Compound 11 (5.2 mg, 10.9 μmol, 1.0 eq.) and benzyl azide (1.35 μL, 10.8 μmol, 1.0 eq.). After 26 hours, the reaction was deemed complete, and solvents were removed *in vacuo*. The residues from each attempt at this reaction were purified together by column chromatography (eluent: DCM/MeOH gradient) to afford the pure product as a yellow-brown solid (8 mg, 13.1 μmol, 40%). *R*_f_: 0.19 (99 : 1 DCM/MeOH); HRMS (ESI-TOF) *m*/*z*: found 632.1603 [M + Na]^+^ calcd for C_25_H_31_N_5_O_6_S_2_Na 632.1608; *ν*_max_ 2928, 2253, 1714, 1573, 1452, 1336, 1149, 1066, 971, 904, 723, 647, 513 cm^−1^; *δ*_H_ (500 MHz, CDCl_3_) 7.36–7.32 (4H, m, Ar***H***), 7.23 (1H, t, *J* = 8.0, Ar***H***), 7.17–7.15 (3H, m, Ar***H***), 7.08 (1H, d, *J* = 8.0, Ar***H***), 6.99 (1H, d, *J* = 8.0, Ar***H***), 6.68 (1H, d, *J* = 7.5, Ar***H***), 5.73 (1H, d, *J* = 16.0, PhC***H***_e_H_f_–), 5.52 (1H, d, *J* = 14.0, –OCH_a_***H***_b_–), 5.41 (1H, d, *J* = 16.0, PhC***H***_e_H_f_–), 5.37 (1H, d, *J* = 14.0, –OCH_a_***H***_b_–), 5.25 (1H, d, *J* = 13.0, –OC***H***_c_H_d_–), 5.05 (1H, d, *J* = 13.0, –OCH_c_***H***_d_–), 4.17–3.64 (4H, m, 2× –NC***H***_2_–), 2.63 (3H, s, –SO_2_CH_3_), 2.57 (3H, s, –SO_2_CH_3_), 2.10–1.69 (4H, m, –NCH_2_C***H***_2_–); *δ*_C_ (125 MHz, CDCl_3_) 159.0, 157.5, 144.5, 140.6, 140.0, 134.6, 132.0, 129.4, 129.3, 129.0, 128.7, 128.3 128.1, 127.1, 121.1, 118.9, 115.5, 114.2, 63.4, 61.2, 52.4, 51.3, 51.2, 36.5, 35.3, 25.4, 24.1; *m*/*z* (ESI) 632.1 [M + Na]^+^

## Data availability

The research data (and/or materials) supporting this publication can be accessed at https://wrap.warwick.ac.uk/.

## Author contributions

Sam Forshaw, Richard C. Knighton, Neelam Tiwari, Samson M. Oladeji and Andrew C. Stevens carried out synthetic chemistry, contributed to the design of the project and contributed to the writing up. Jeremy S. Parker contributed to the project design. William T. Scott carried out synthetic chemistry and molecular biology, contributed to the design of the project and contributed to the writing up. Yean Ming Chew and Jami Reber carried out molecular biology, contributed to the design of the project and contributed to the writing up. Guy J. Clarkson completed the X-ray crystal structure analyses and provided data for the project. Mohan K. Balasubramanian and Martin Wills conceived and directed the project.

## Conflicts of interest

There are no conflicts to declare.

## Supplementary Material

OB-022-D3OB01712E-s001

OB-022-D3OB01712E-s002
