# Supplementary material for: Increasing the versatility of the biphenyl-fused-dioxacyclodecyne class of strained alkynes
Source: Org Biomol Chem. 2023 Dec 13;22(3):590–605. doi: 10.1039/d3ob01712e (PMC10792613; doi:10.1039/d3ob01712e)
Supplement: OB-022-D3OB01712E-s001 [file OB-022-D3OB01712E-s001.pdf]

Electronic Supplementary Material (ESI) for Organic & Biomolecular Chemistry.
This journal is © The Royal Society of Chemistry 2023

Supporting information

Increasing the versatility of the biphenyl-fused-dioxacyclodecyne class of strained alkynes.

.

Sam Forshaw,[a] Jeremy S. Parker,[b] William T. Scott,[a,c] Richard C. Knighton,[a,d] Neelam Tiwari,[a] Samson M. Oladeji,[a] Andrew C. Stevens,[a] Yean Ming Chew,[c] Jami Reber,[a] Guy J. Clarkson,[a] Mohan K. Balasubramanian[c] and Martin Wills[a]*

a. Department of Chemistry, The University of Warwick, Coventry, CV4 7AL, UK.

b. Early Chemical Development, Pharmaceutical Sciences, IMED Biotech Unit, AstraZeneca, Macclesfield, SK10 2NA, UK.

c. Warwick Medical School, The University of Warwick, Coventry, CV4 7AL, UK.

d. School of Chemistry, University of Southampton, SO17 1BJ, UK.

Summary of second order rate constants with benzylazide	**S2**
Procedure for kinetic measurements	**S3**
Cycloaddition rate graphs	**S4**
NMR Spectra	**S20**
X-ray crystal data	**S95**
Studies of binding to azide-functionalised proteins	**S106**
Excel spreadsheets for kinetic runs	**S126**

Summary of second order rate constants with benzylazide.

Cycloadditions were carried out in $CDCl_3$ unless otherwise stated.

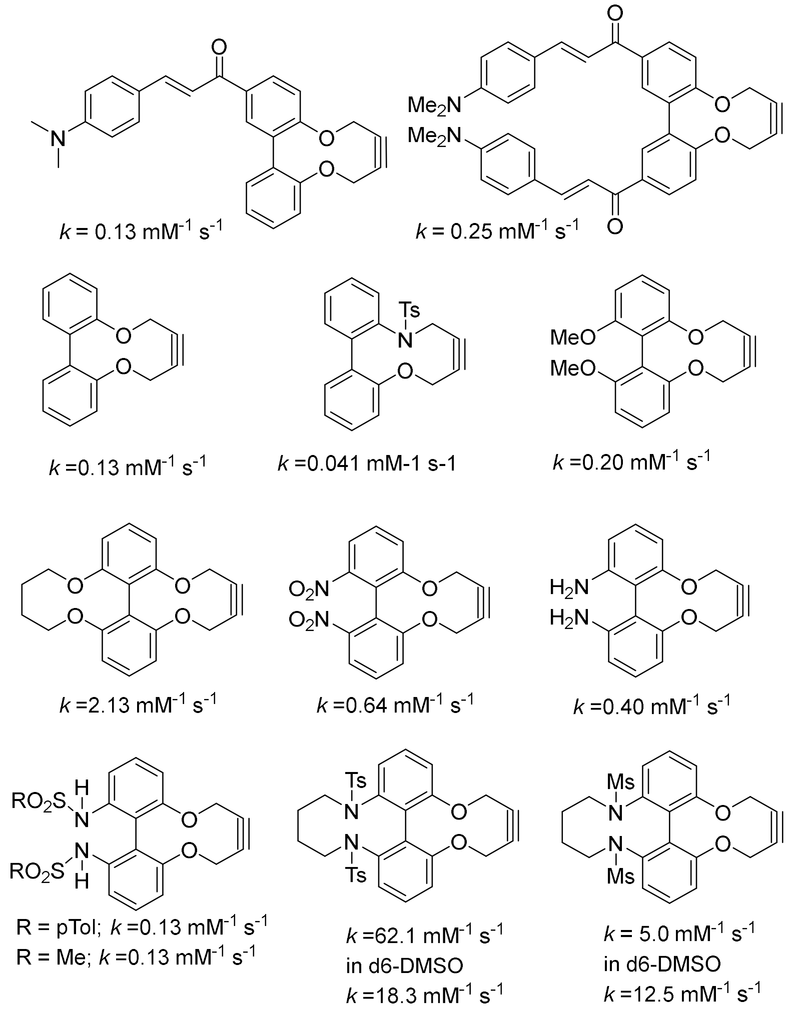

Figure S1. Summary of second order rate constants (*k*) with benzylazide at rt, in $CDCl_3$ (unless otherwise stated), for compounds reported in this paper. Full details of kinetic runs following conversion over time and derivation of second order rate constants are given in the accompanying excel spreadsheets at the end of this Supporting Information.

Procedure for kinetic measurements.

A sample of the alkyne (typically 5-15 mg) was dissolved in $CDCl_3$ (0.5 mL) to give the initial solution of known concentration which was calculated for each run. The ^1H NMR spectrum was recorded at room temperature (ca. 20 ºC). Benzylazide (1.0 equivalent) was then added using a microsyringe and ^1H NMR spectra were recorded at intervals until no further conversion was noted. The conversion was calculated by integrating well-separated peaks in the starting material and product and correcting for the number of protons giving rise to each peak. The data are summarised in the pdfs of the excel pages used to calculate the second order rate constants in each case. These are located at the end of this Supporting Information. Reactions of compounds **54** and **56** were also recorded in d6-DMSO following the procedure above. The use of ^1H NMR allowed the ratio of BnN_3 to the alkene at the start and throughout to be confirmed and this was within ca 10% of a 1:1 ratio in each case. An error of ca. 10% can be associated with the measurements. Reactions of compounds **54** and **56** with PhN_3 (using a 0.5 M solution in tBuOMe) were also recorded in $CDCl_3$ following the procedure above, however the ratio of reagents could not be confirmed and a rate constant could not be generated for these reactions.

Kinetic analysis of cycloadditions (see the excel files for full details):

Monochalcone **16** (SF688), conversion vs time:

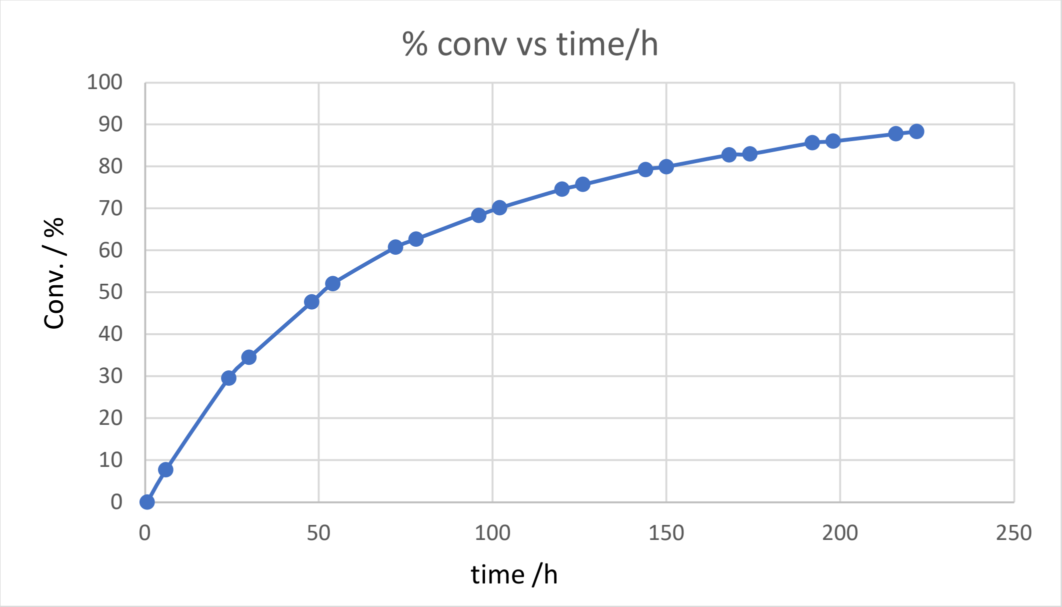

Second order rate graph:

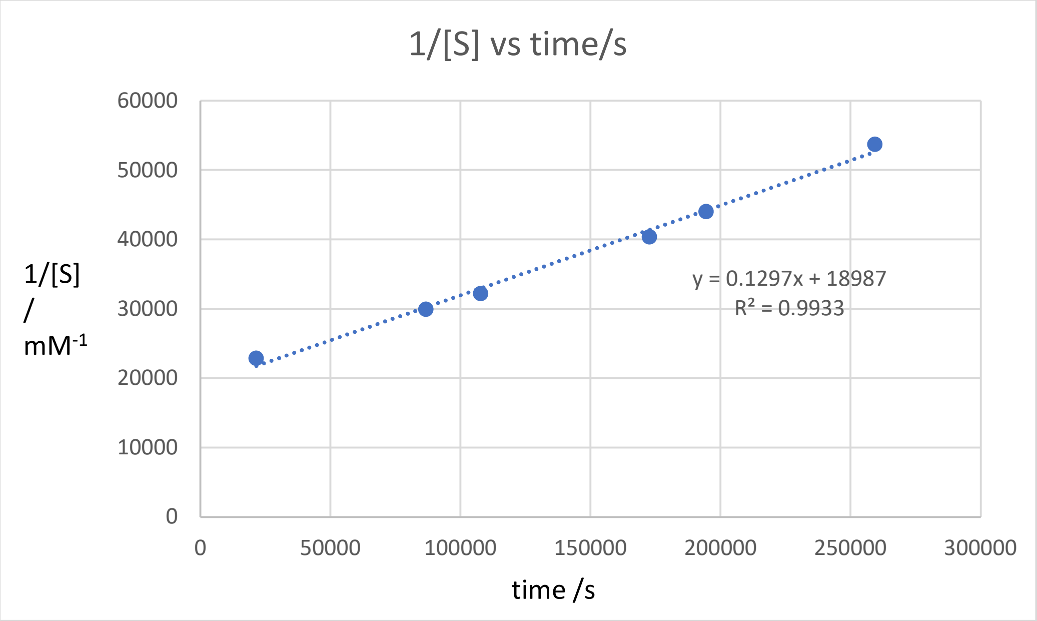

[alkyne] initial = 47 mM, $CDCl_3$.

$k = 0.13$ mM^{-1} s^{-1}.

Dichalcone **22** (SF689), conversion vs time:

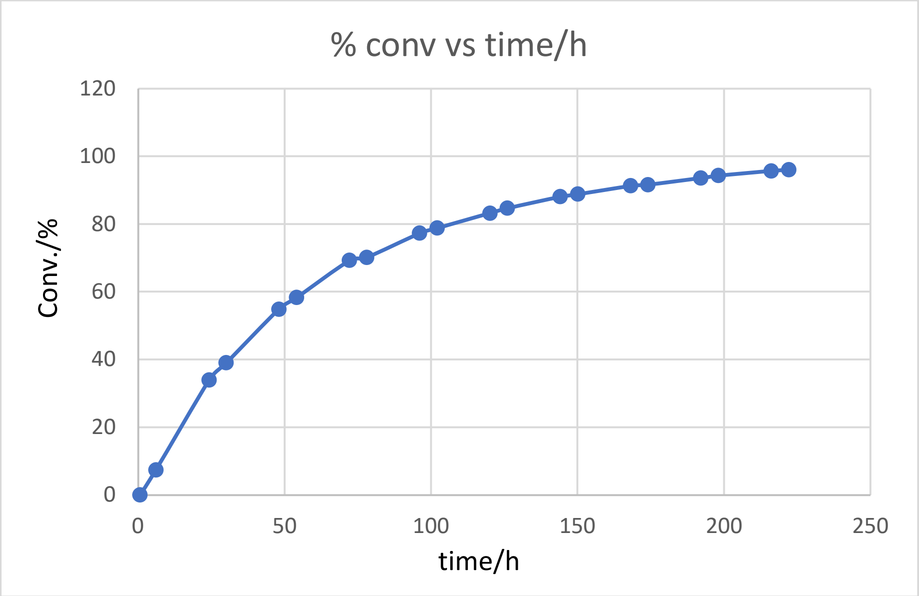

Second order rate graph:

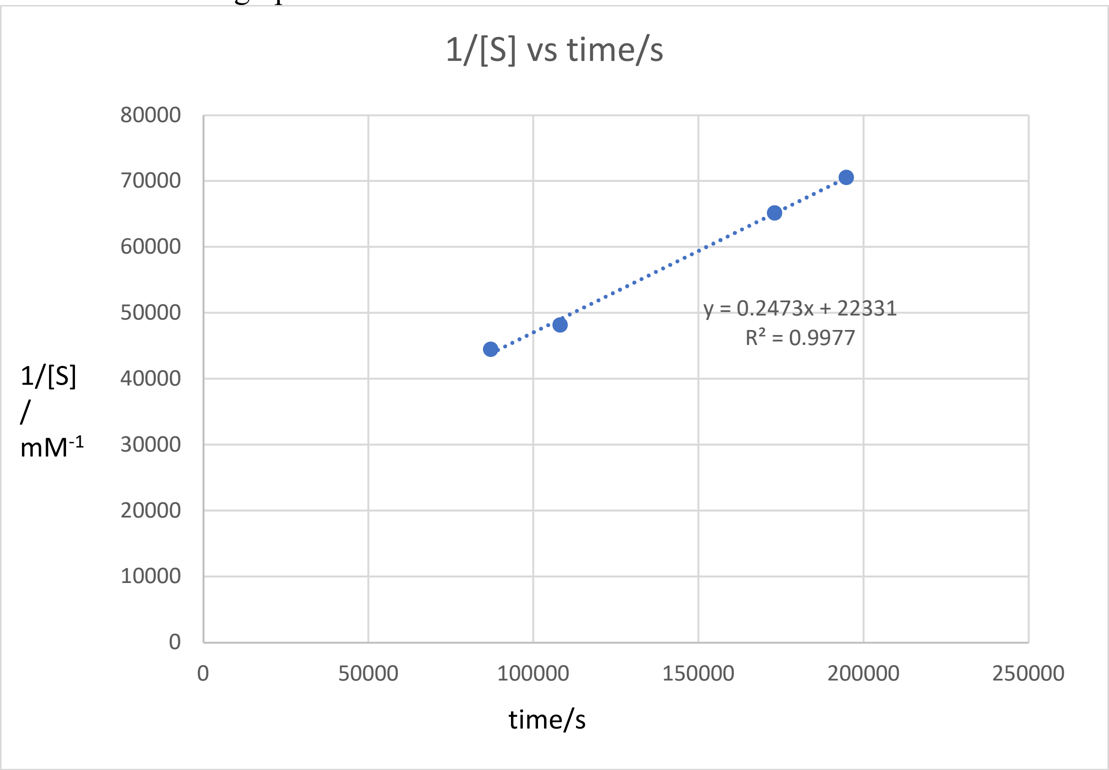

[alkyne] initial = 34 mM, $CDCl_3$.

k = 0.25 mM^{-1} s^{-1}.

NTs/O alkyne **29** (SF379), conversion vs time:

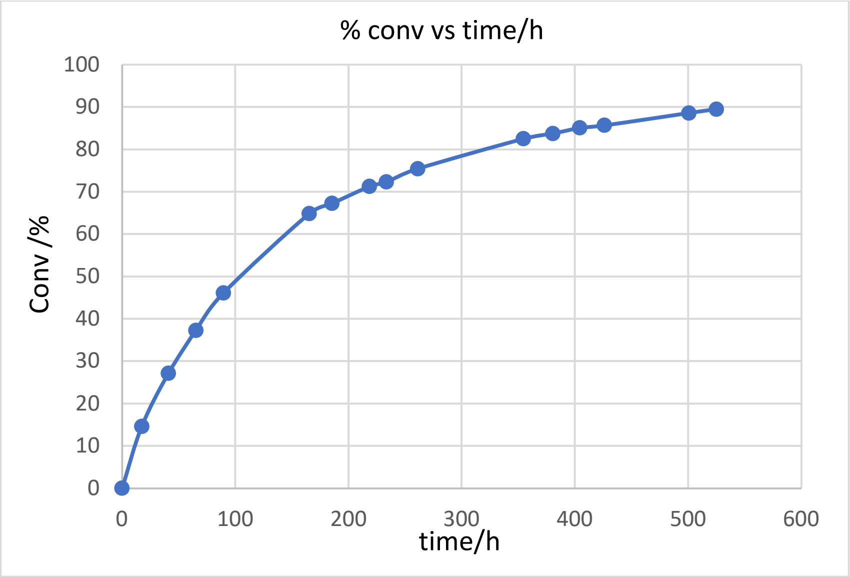

Second order rate graph:

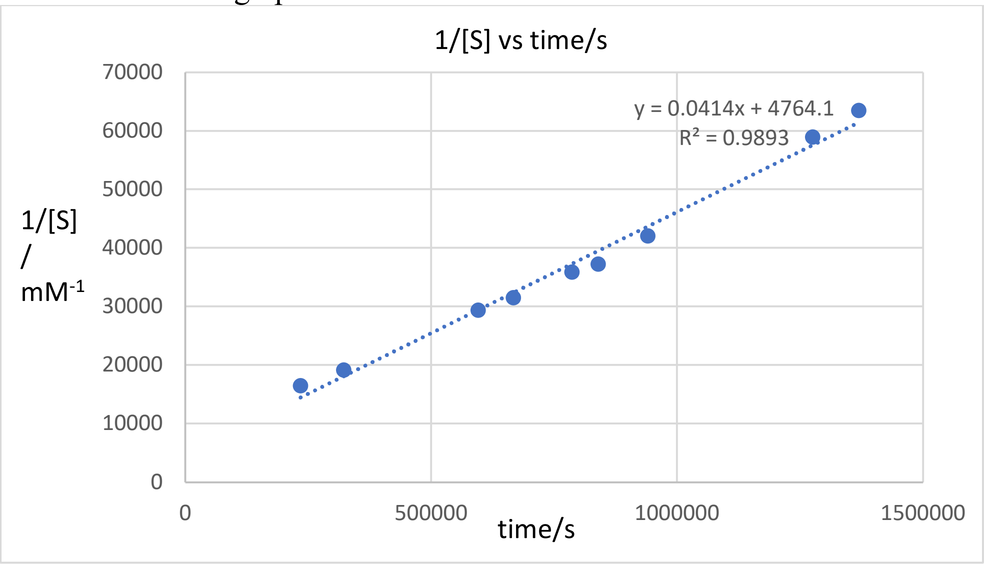

[alkyne] initial = 97 mM, $CDCl_3$.

k = 0.041 mM^{-1} s^{-1}.

Dimethoxy **37** (SFAZ46), conversion vs time:

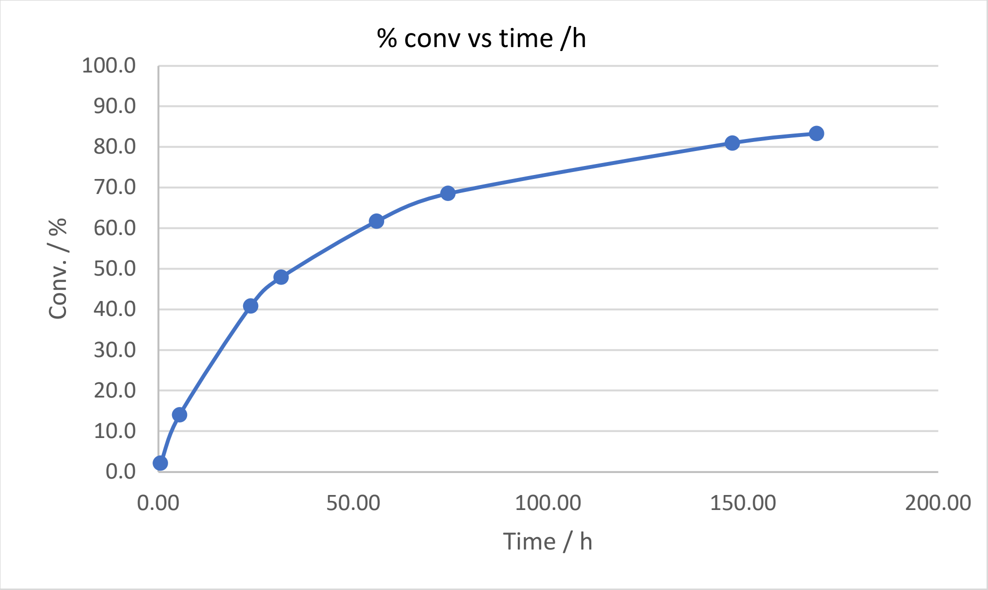

Second order rate graph:

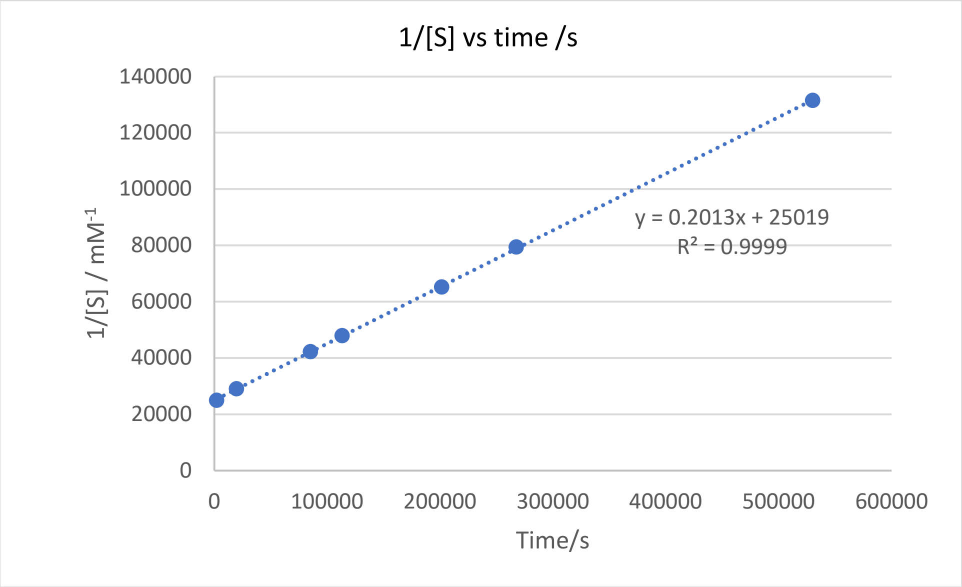

[alkyne] initial = 40 mM, $CDCl_3$.

$k = 0.20\ mM^{-1}\ s^{-1}$.

C4 diether bridged **44** (SF675), conversion vs time:
Compared to **37** (SFAZ46, shown on previous page) to illustrate the effect of the 4C bridge on the reactivity, compared to two methoxy groups.

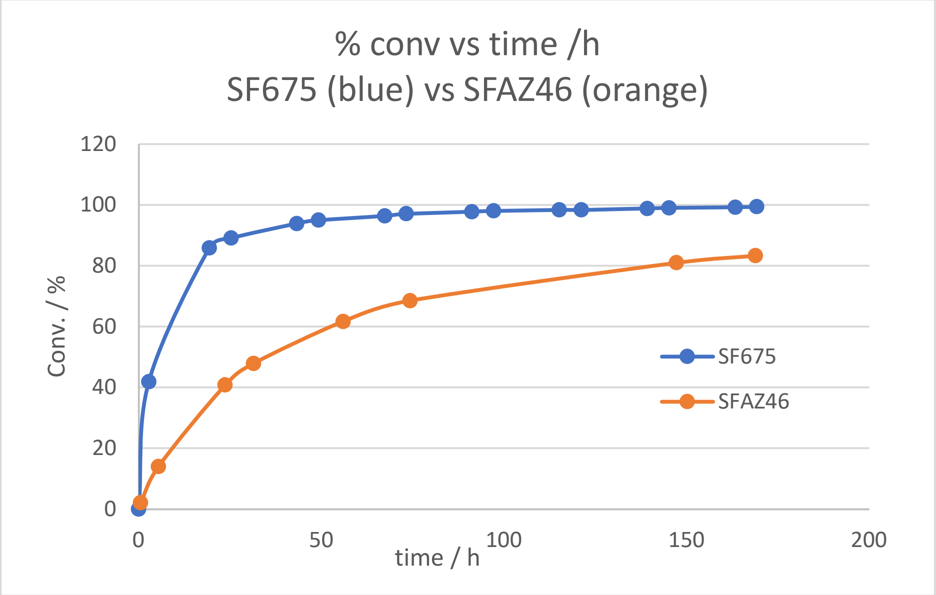

Second order rate graph (vs SFA46):

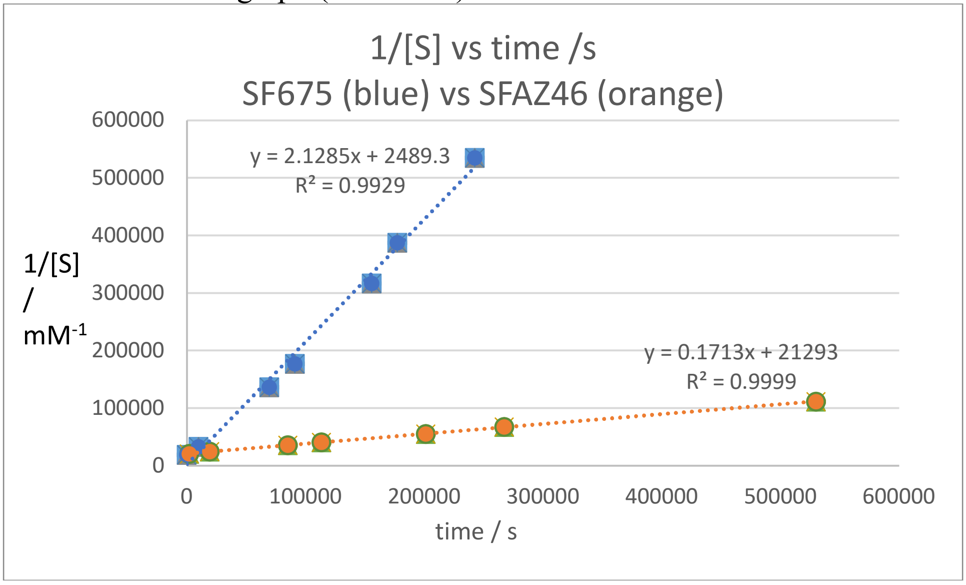

[alkyne] initial = 52 mM, $CDCl_3$.

k = 2.13 mM^{-1} s^{-1}.

Di NO2 **49** (SF720), conversion vs time:

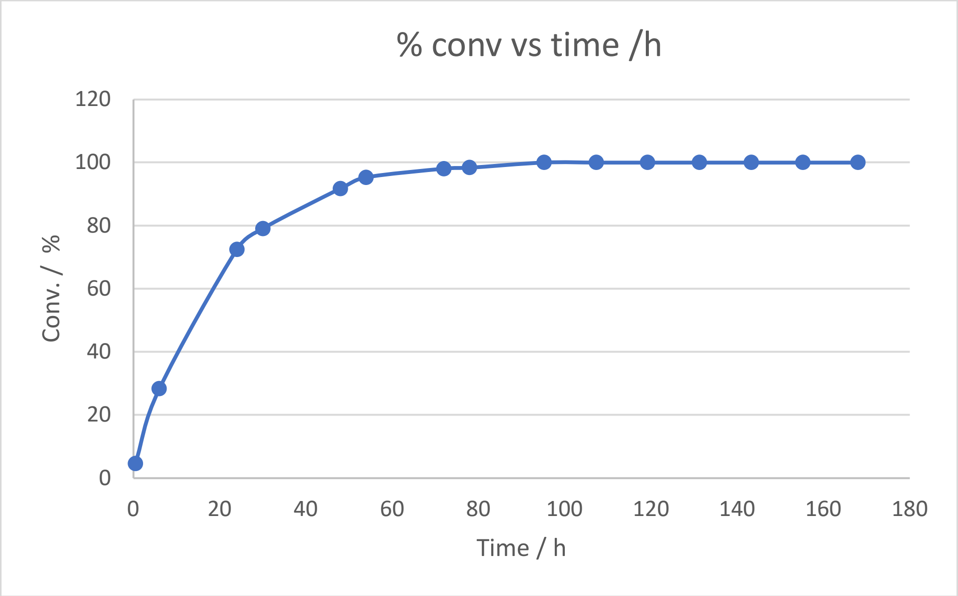

Second order rate graph:

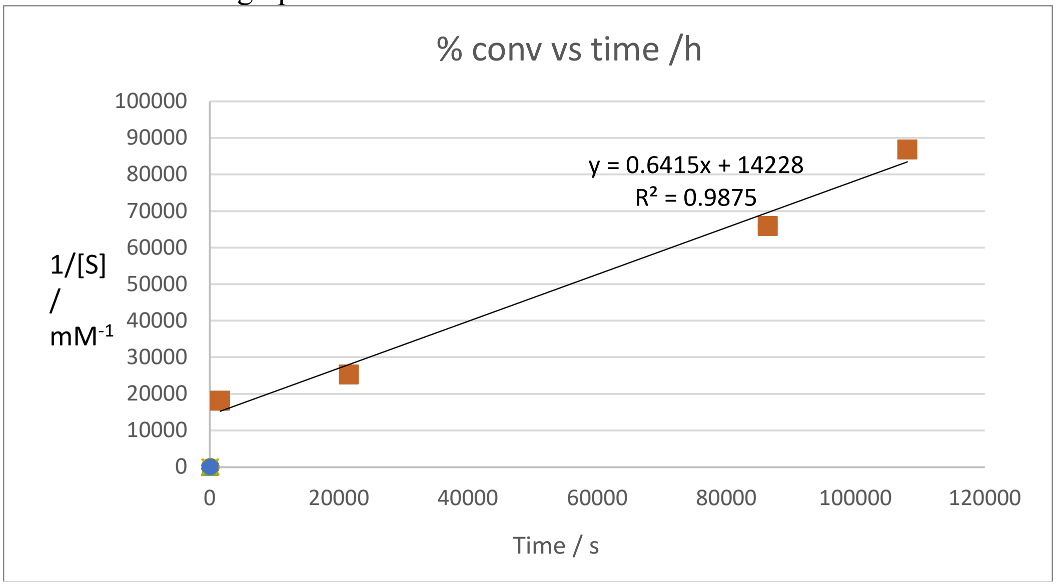

[alkyne] initial = 55 mM, $CDCl_3$.

$k = 0.64\ mM^{-1}\ s^{-1}$.

Di NH2 **50** (SF 752), conversion vs time:

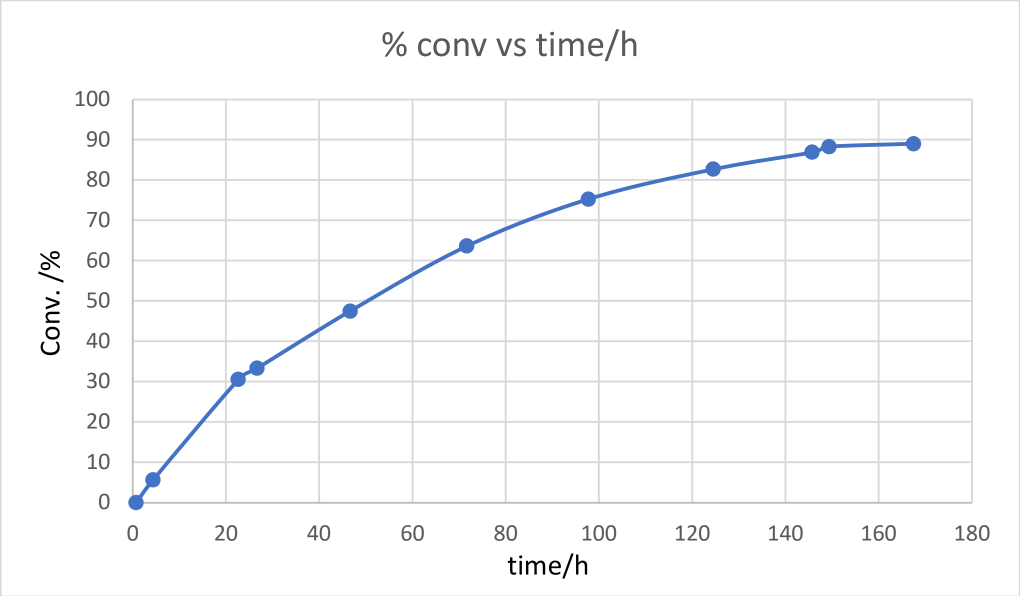

Second order rate graph:

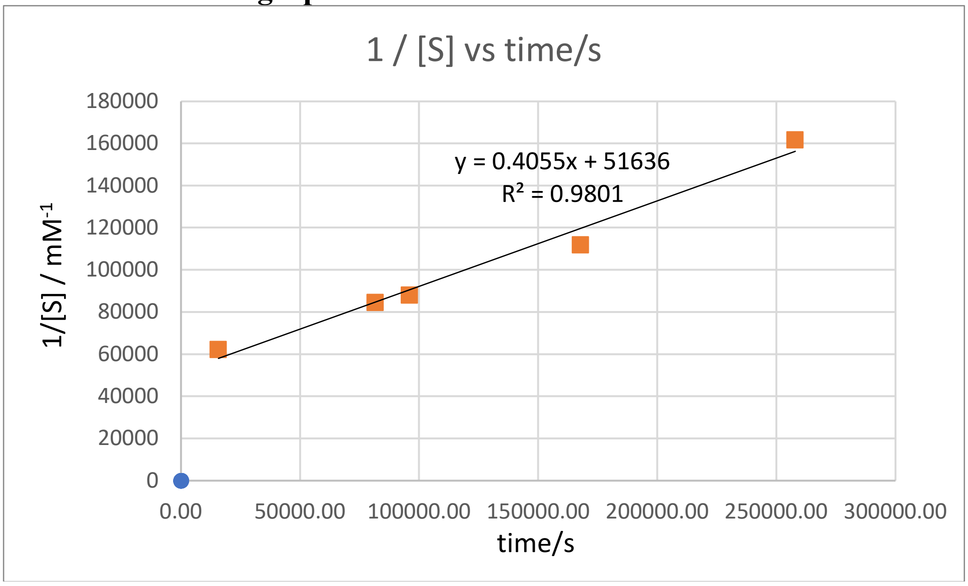

[alkyne] initial = 17 mM, $CDCl_3$.

$k = 0.40$ mM^{-1} s^{-1}.

Di NTs non C4 **51** (SF756), conversion vs time:

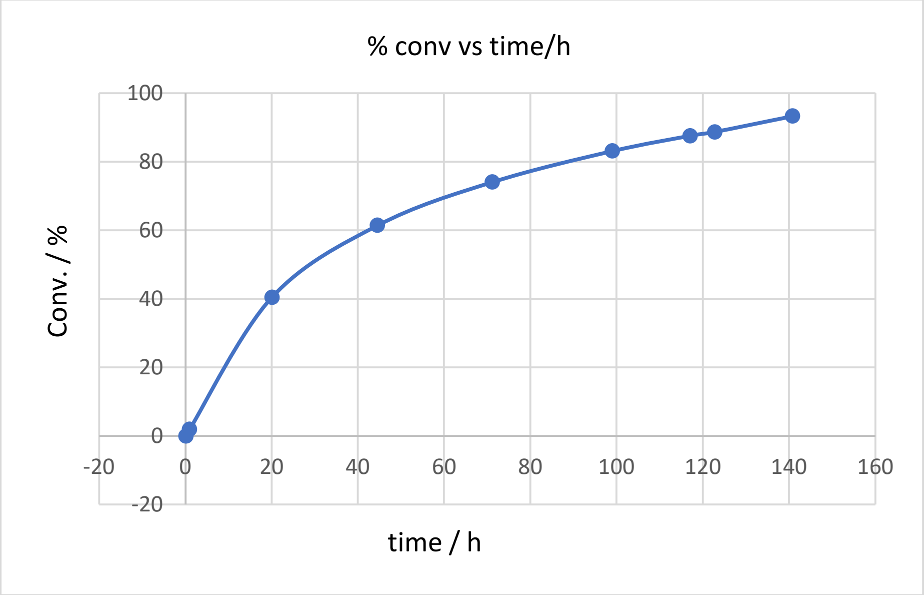

Second order rate graph:

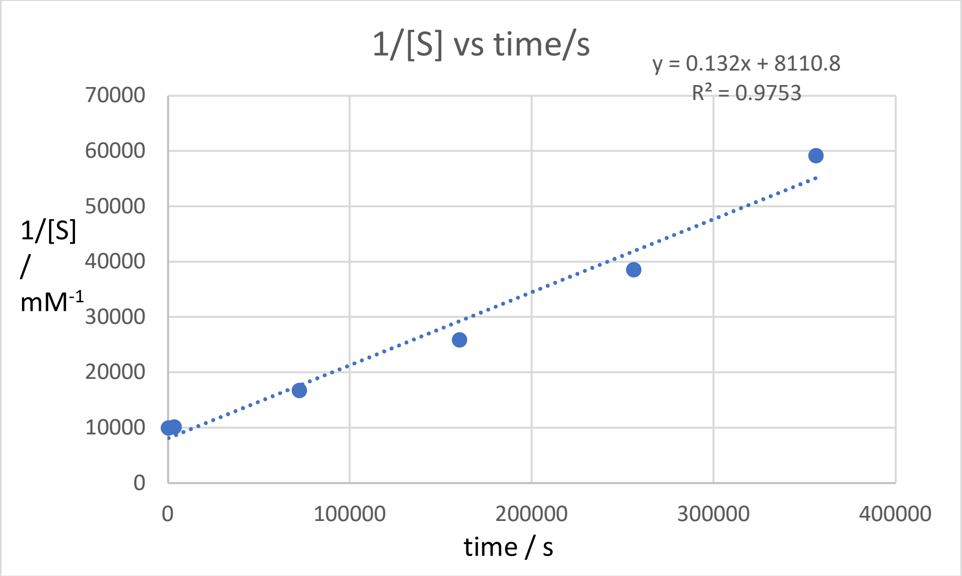

[alkyne] initial = 100 mM, $CDCl_3$.

k = 0.13 mM^{-1} s^{-1}.

Di Ms non C4 **53** (AS12), conversion vs time:

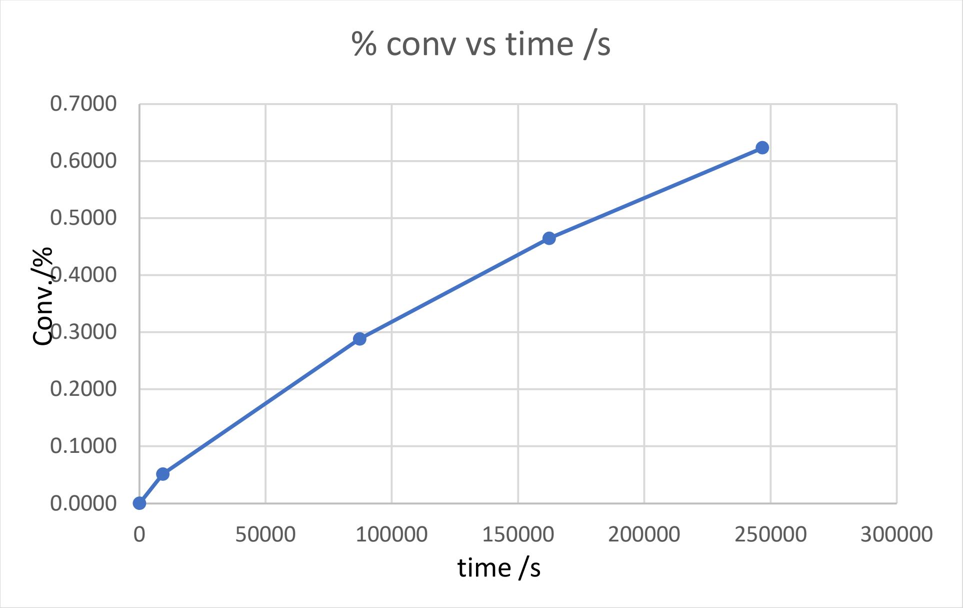

Second order rate graph:

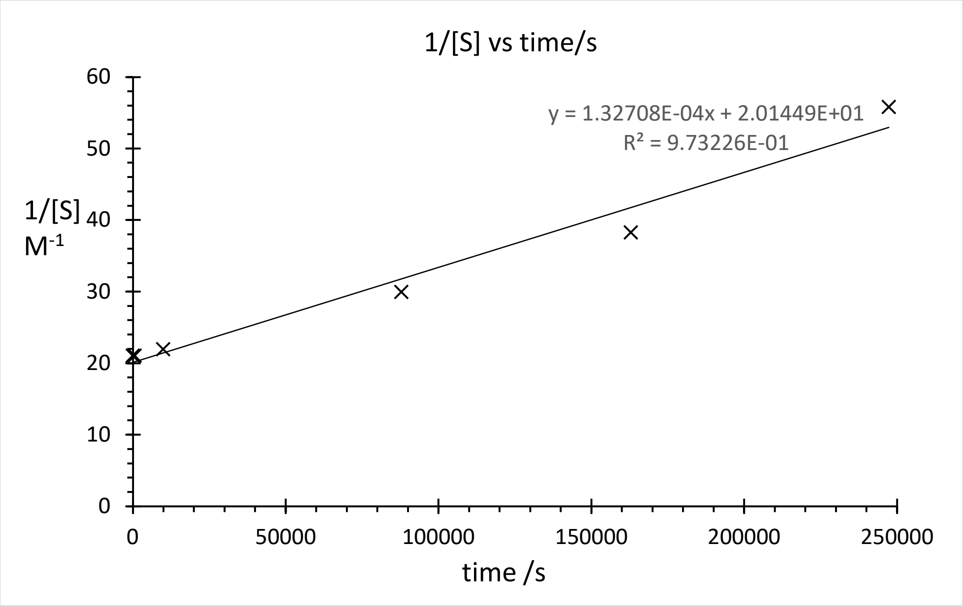

[alkyne] initial = 47 mM, $CDCl_3$.

k = 0.13 mM^{-1} s^{-1}.

DiNTs C4 **54** (SF761), conversion vs time:

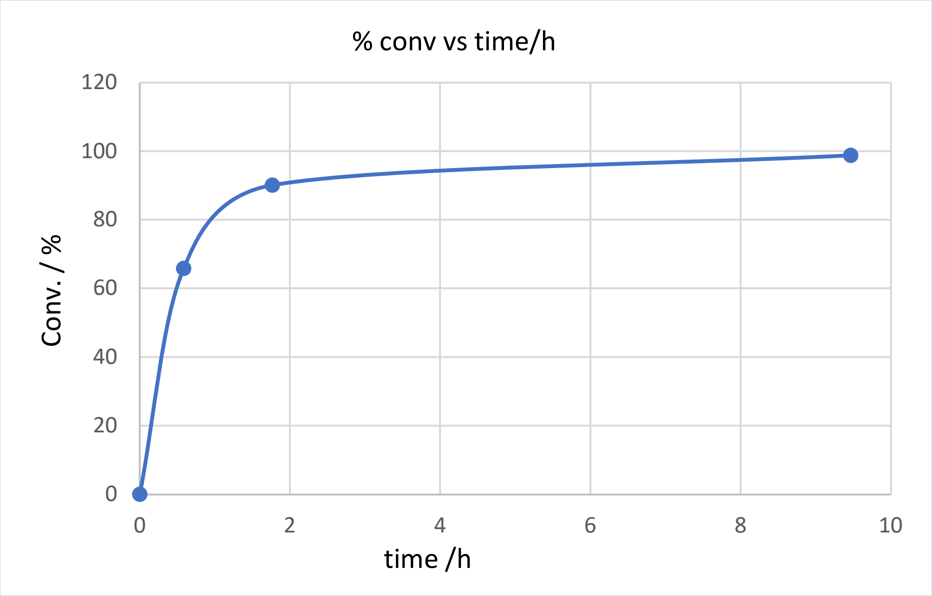

Second order rate graph:

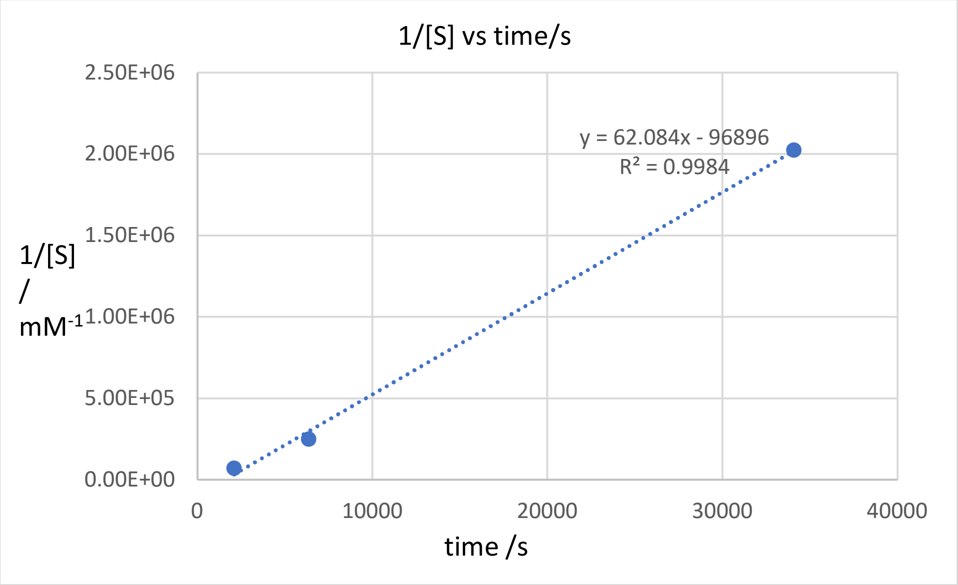

[alkyne] initial = 40 mM, $CDCl_3$.

k = 62.1 mM^{-1} s^{-1}.

DiNTs C4 (SF761), Repeat on a 1.2 mg scale available (SF763), conversion vs time:

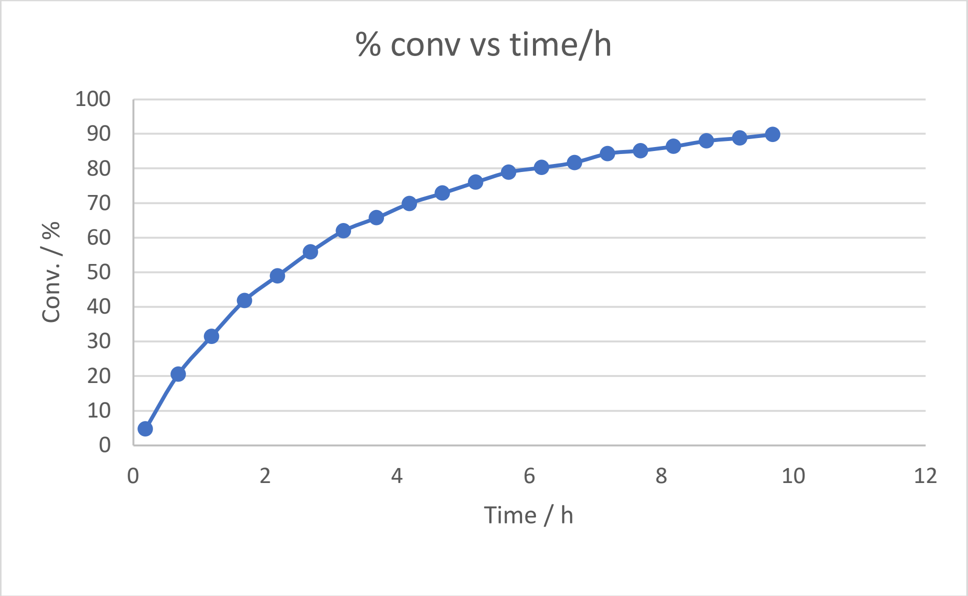

Second order rate graph:

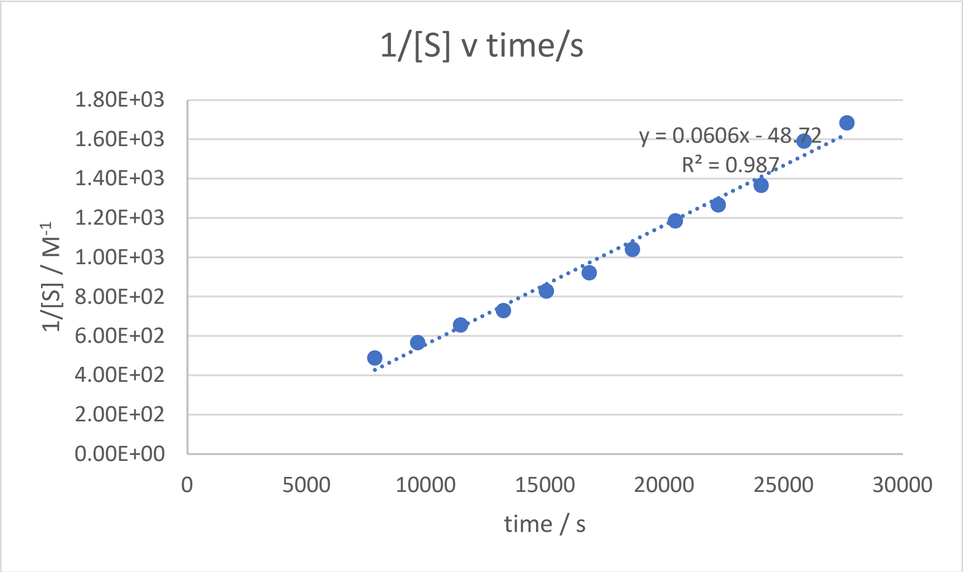

[alkyne] initial = 4 mM, $CDCl_3$.

k = 60 mM^{-1} s^{-1}.

diMs, C4 **56** (AS36). Conversion vs time:

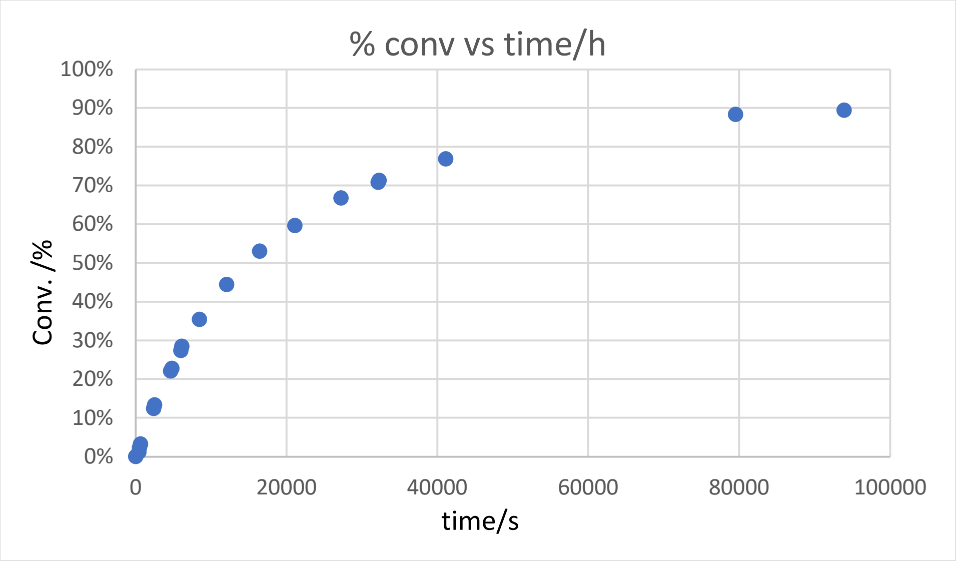

Second order rate graph:

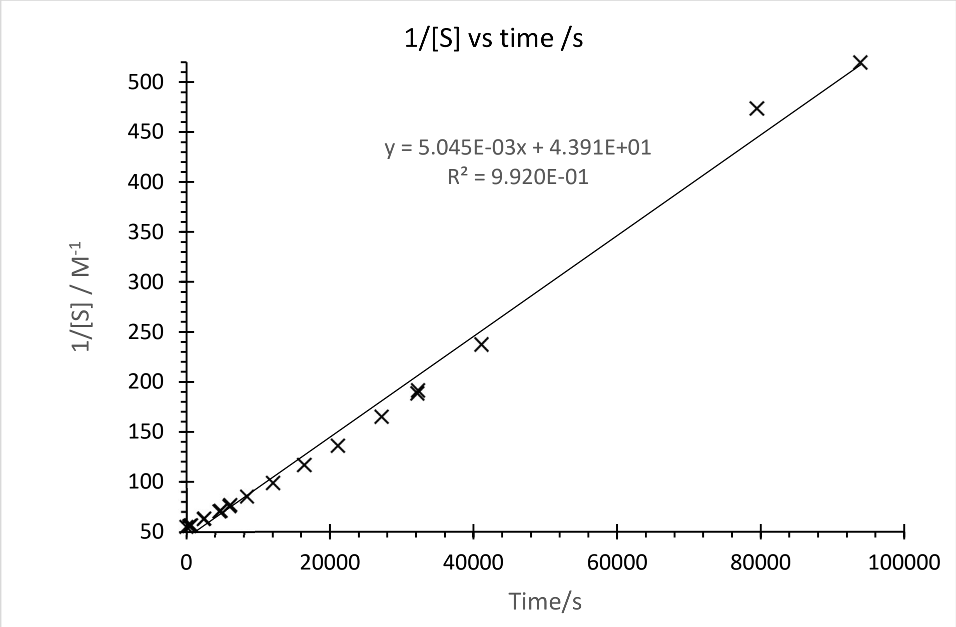

[alkyne] initial = 22 mM, $CDCl_3$.

$k = 5.0$ mM^{-1} s^{-1}.

In this instance, the NMR spectra for the time course of the experiment have been included, to illustrate the change in peaks observed during the conversion:

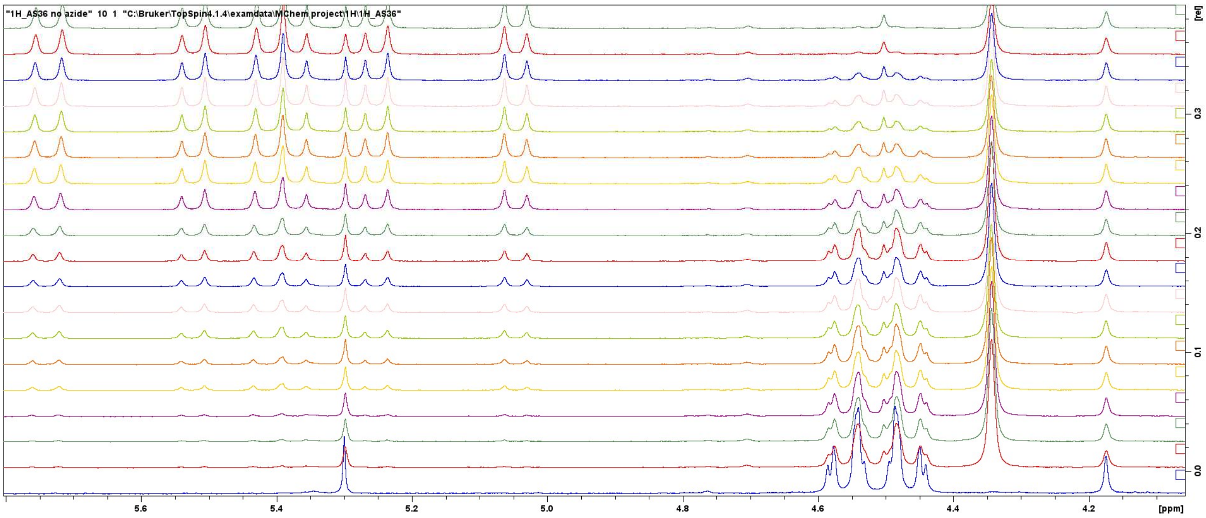

From bottom to top : t = 7 minutes, t = 9 minutes, t = 11 minutes, t = 40 minutes, t = 42 minutes, t = 77 minutes, t = 80 minutes, t = 100 minutes, t = 102 minutes, t = 141 minutes, 201 minutes, t = 274 minutes, t = 352 minutes, t = 454 minutes, t = 536 minutes, t = 538 minutes, t= 685 minutes, t = 22 hours, t = 26 hours.

diTs, C4 **54** in D6-DMSO. Conversion vs time:

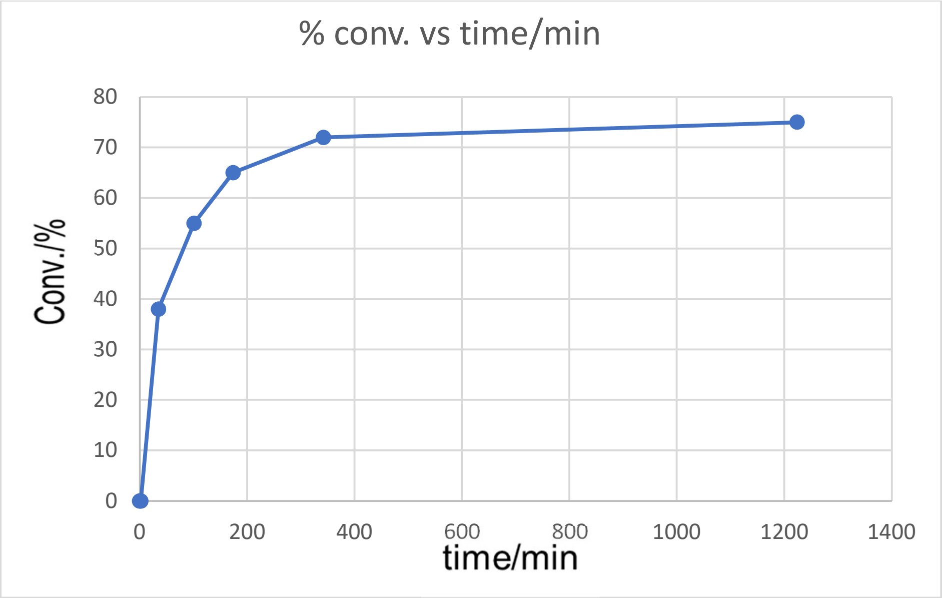

Second order rate graph:

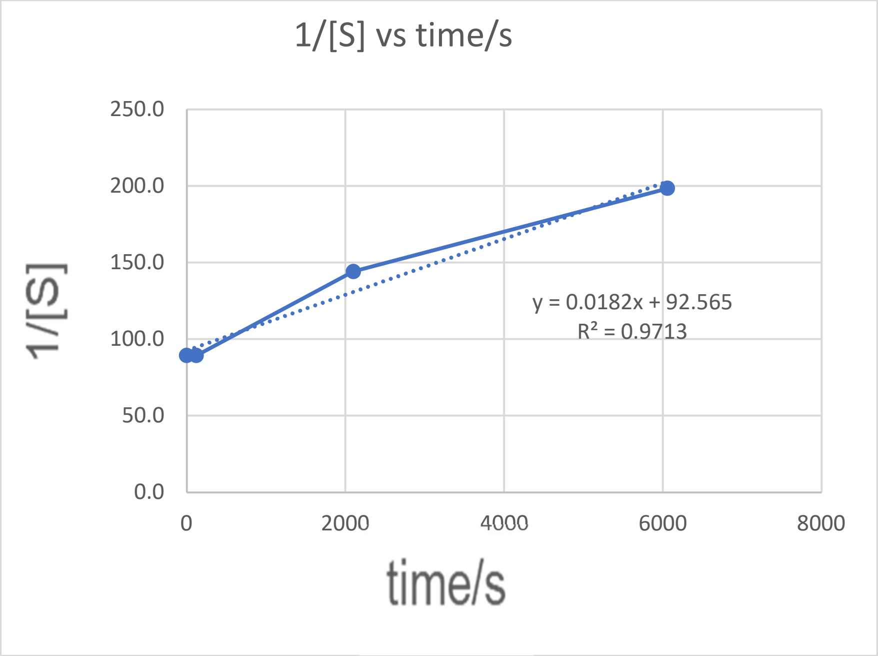

[alkyne] initial = 11 mM, d6-DMSO.

k = 18.3 mM^{-1} s^{-1}.

diMs, C4 **56** in D6-DMSO. Conversion vs time:

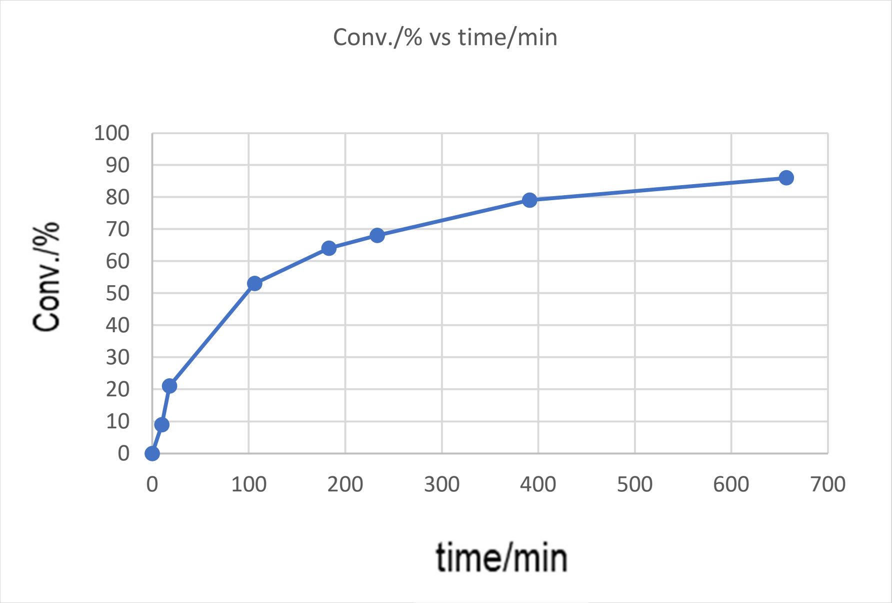

Second order rate graph:

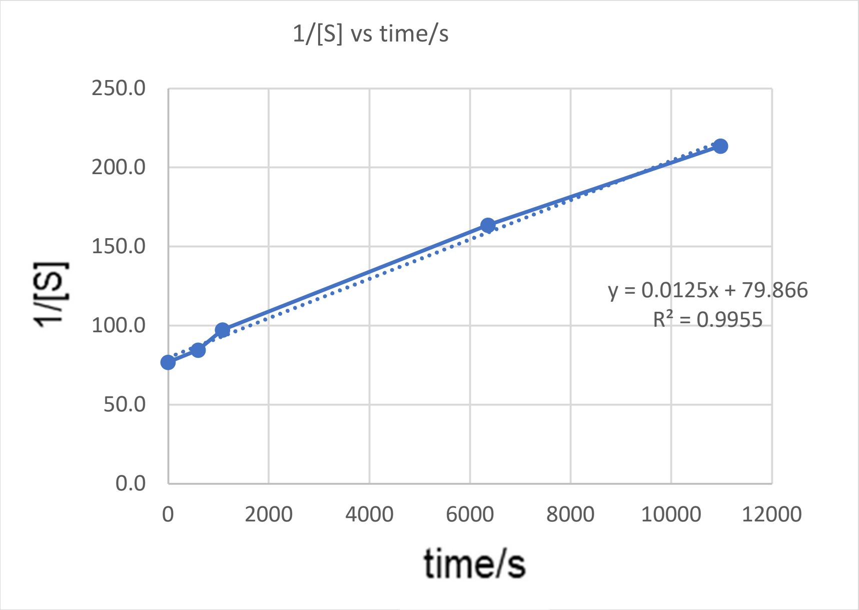

[alkyne] initial = 13 mM, d6-DMSO.

k = 12.5 mM^{-1} s^{-1}.

Reactions of **54** and **56** with PhN_3.

Reaction of **54** with PhN_3 solution in tBuOMe

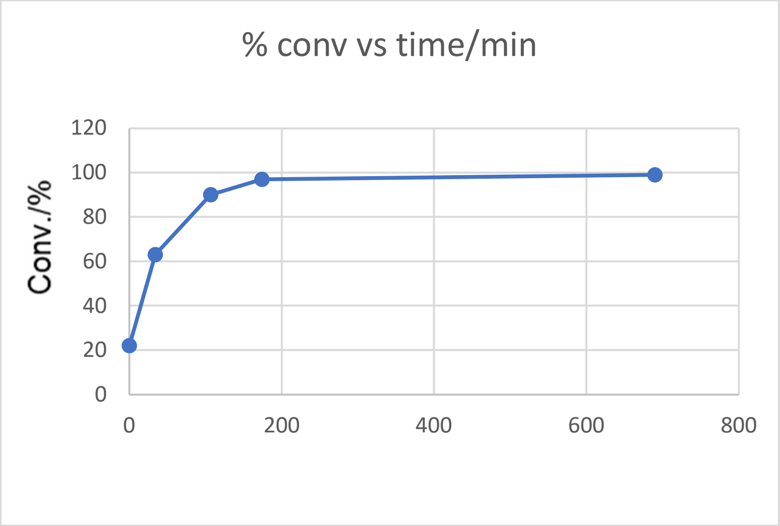

[alkyne] initial = 11.2 mM, $CDCl_3$.

k value cannot be accurately generated because the [PhN_3] cannot be confirmed to be 1 eq.. The product was not purified or fully characterised.

Reaction of **56** with PhN_3 solution in tBuOMe.

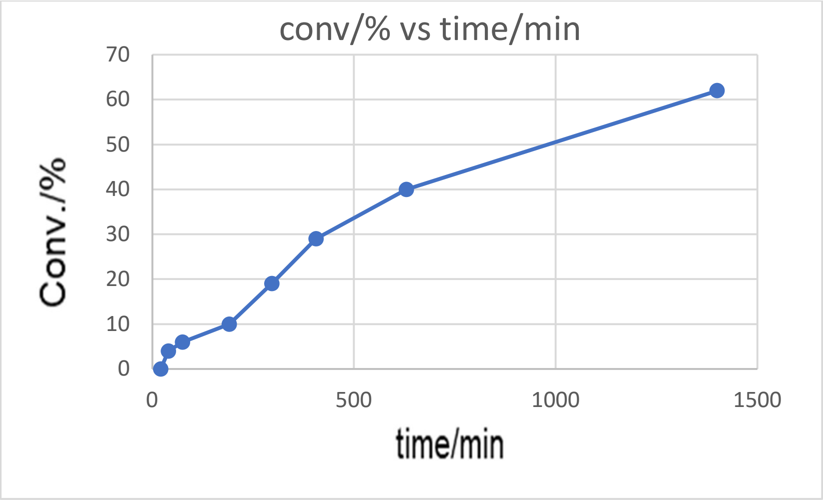

[alkyne] initial = 16.8 mM, $CDCl_3$.

k value cannot be accurately generated because the [PhN_3] cannot be confirmed to be 1 eq. The product was not purified or fully characterised.

NMR spectra:

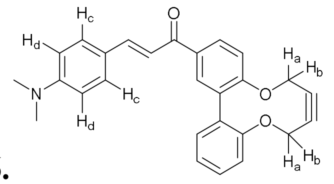

Monochalcone 16.

δ_H (500 MHz, $CDCl_3$):

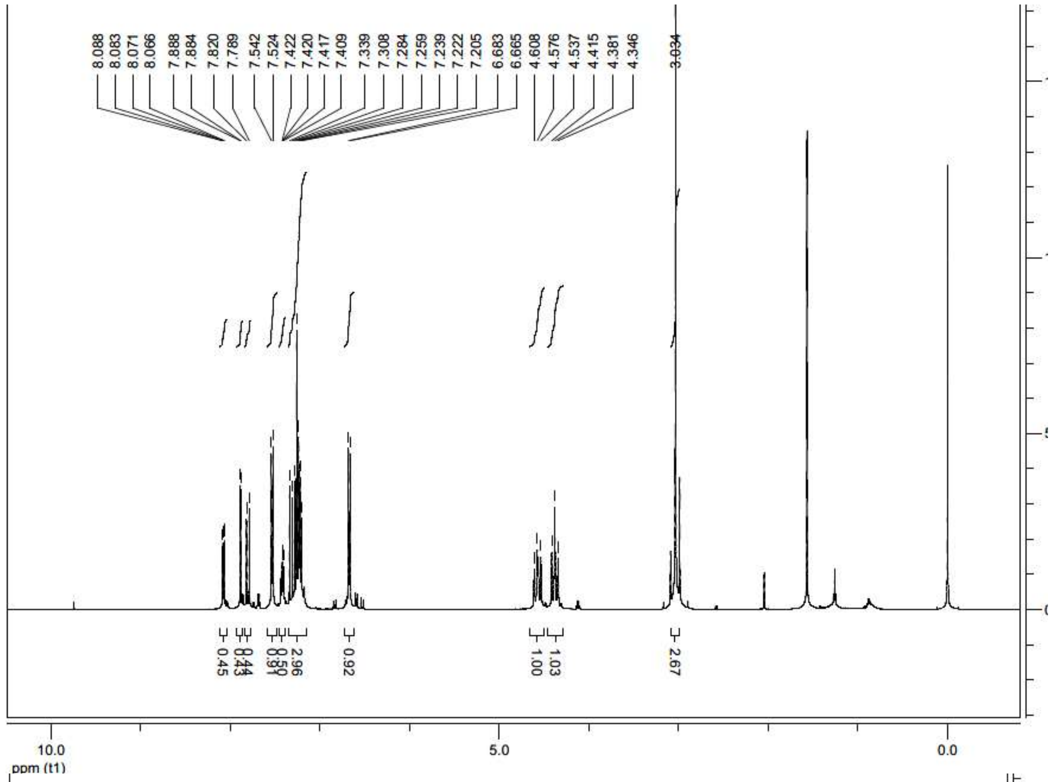

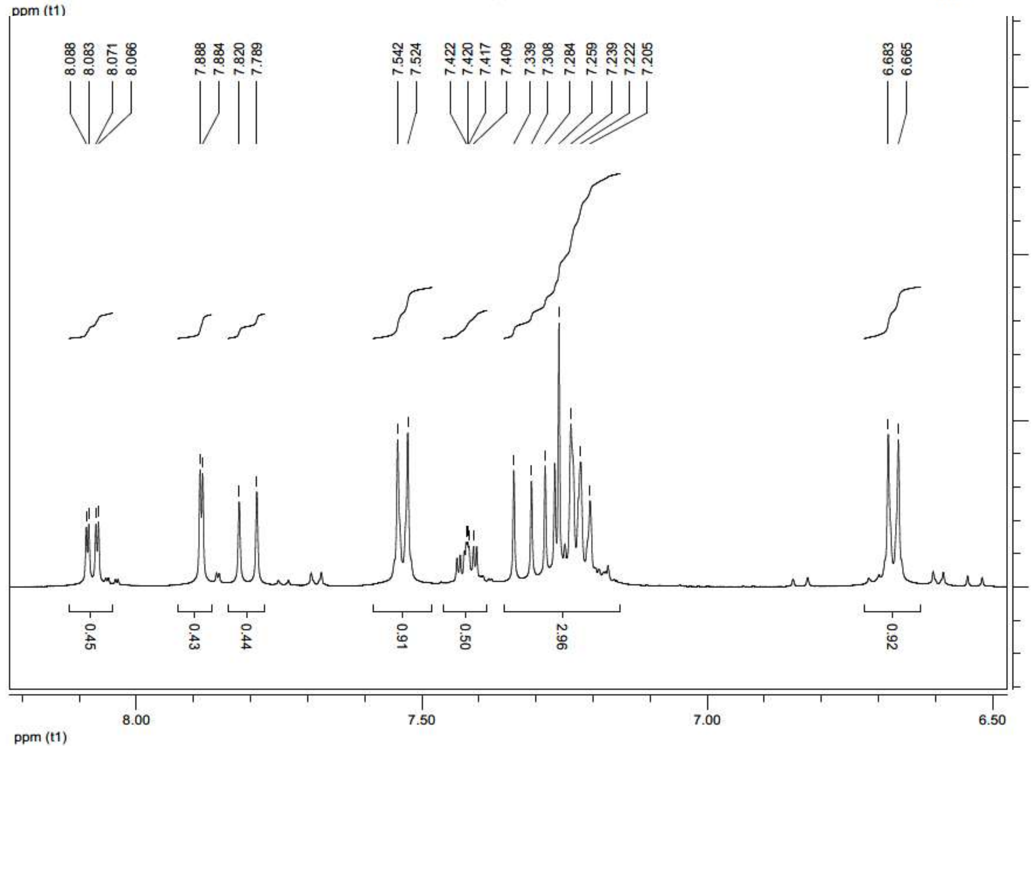

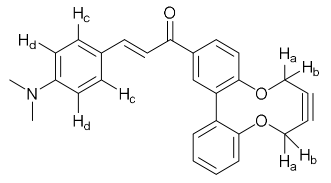

δC (126 MHz, $CDCl_3$)

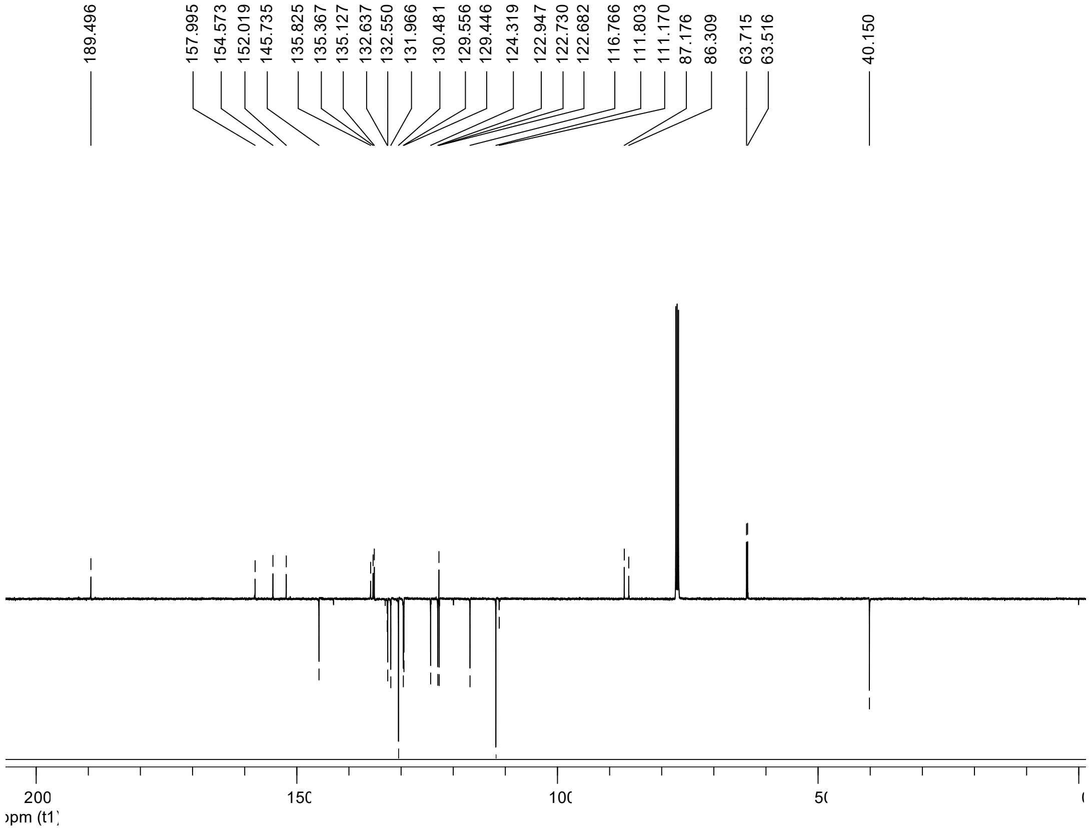

Monochalcone azide adduct 17.

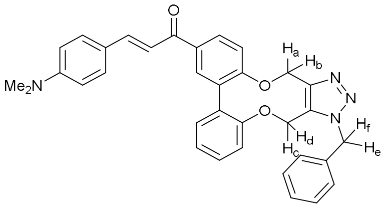

δ_H (500 MHz, $CDCl_3$)

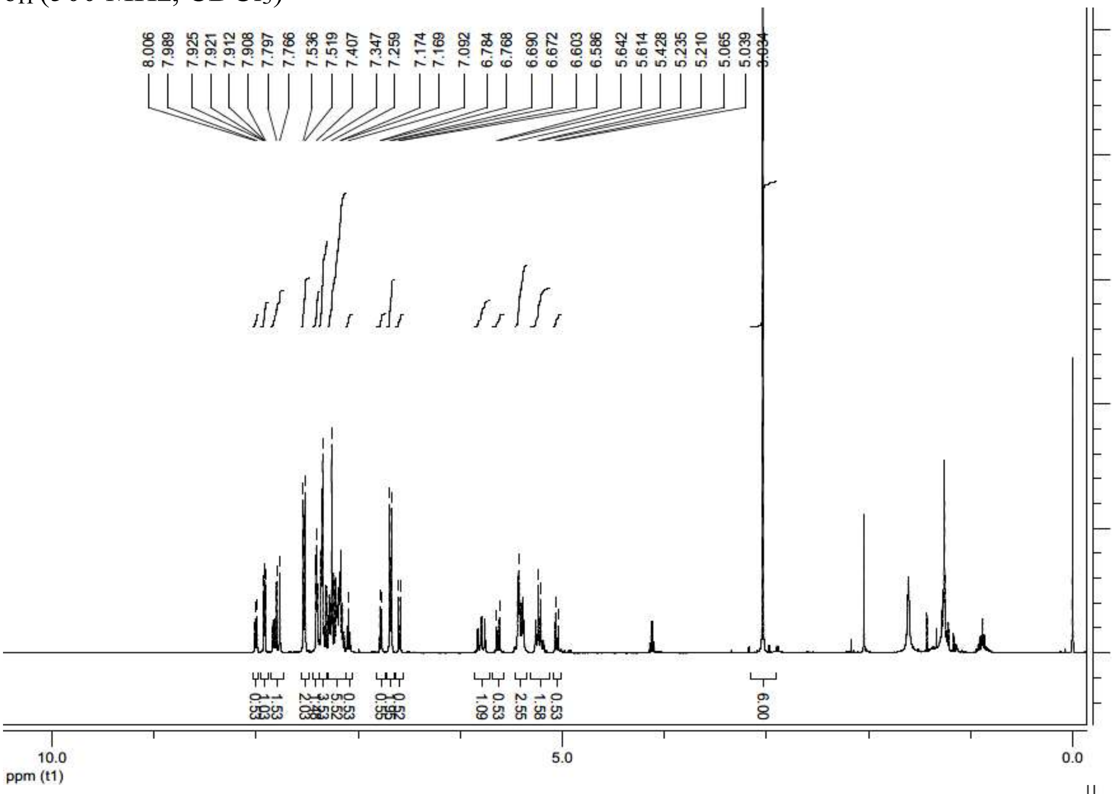

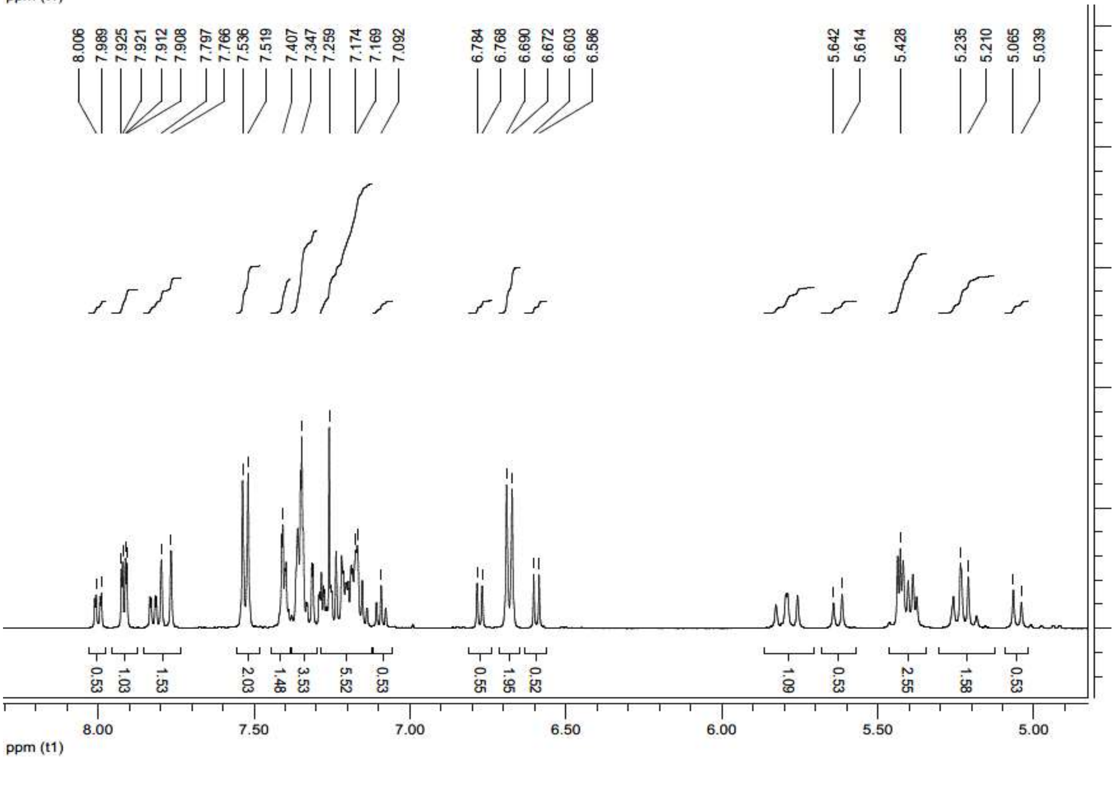

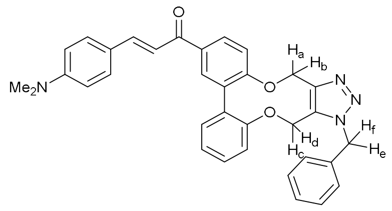

δC (126 MHz, CDCl3)

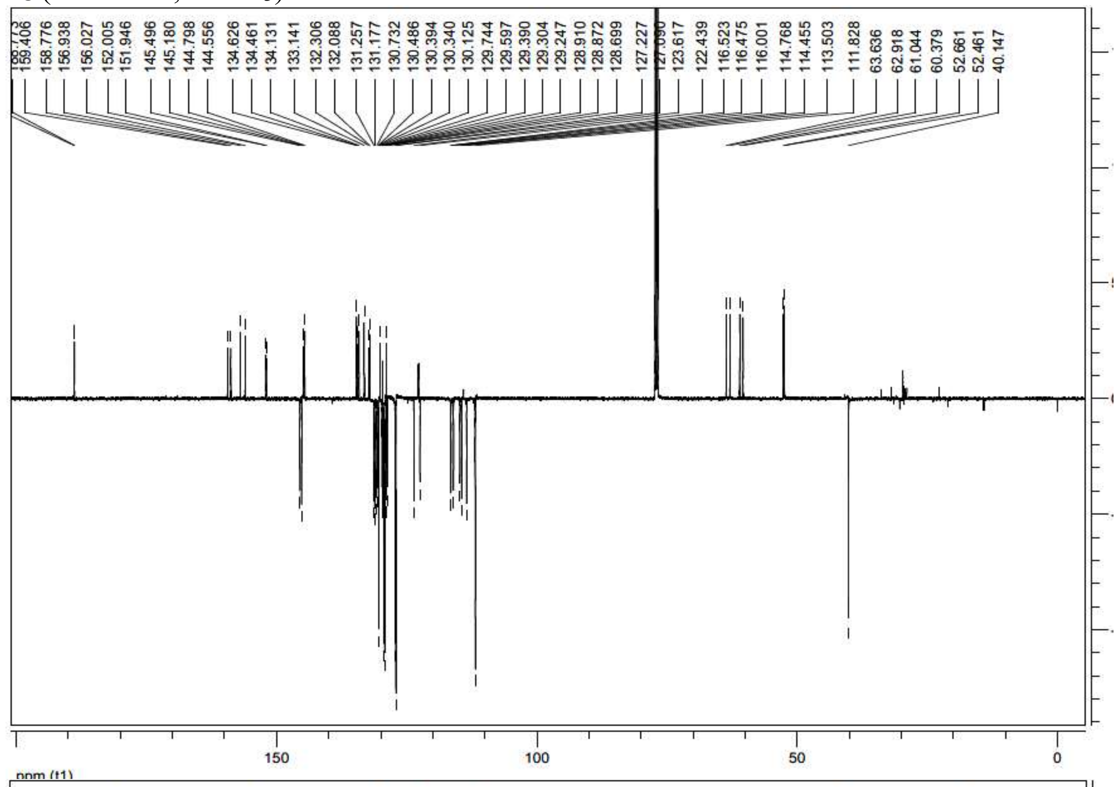

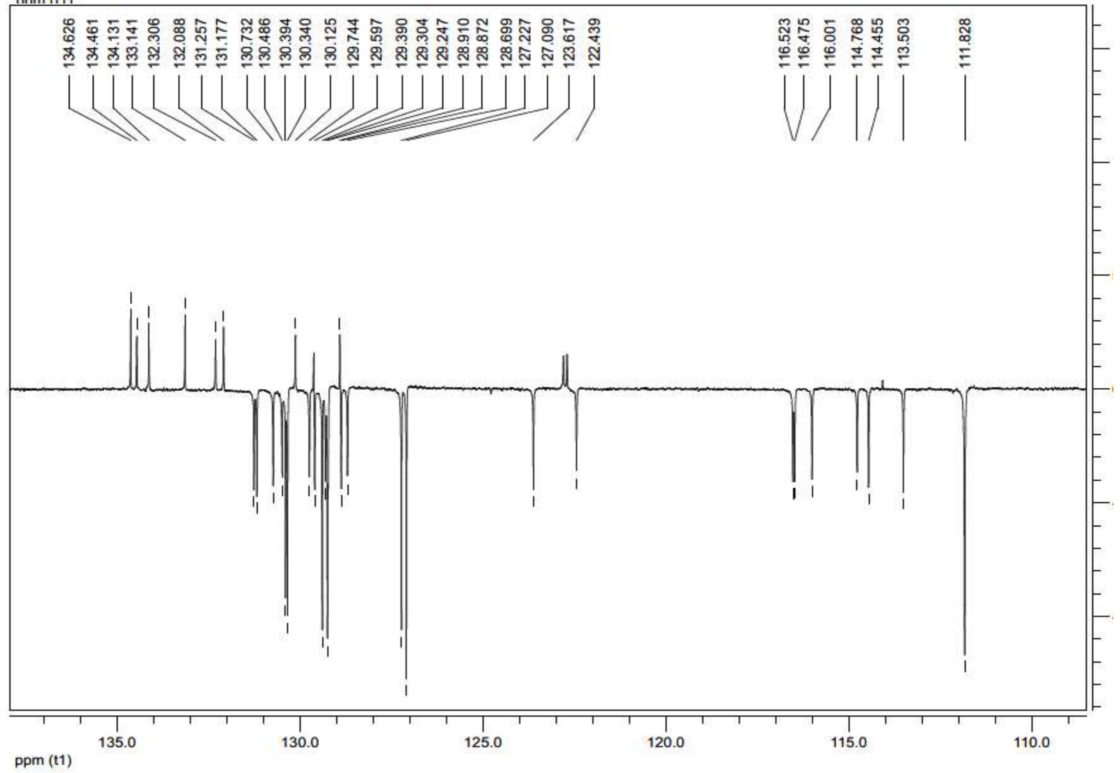

Compound 18.

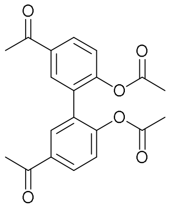

δ_H (500 MHz, $CDCl_3$)

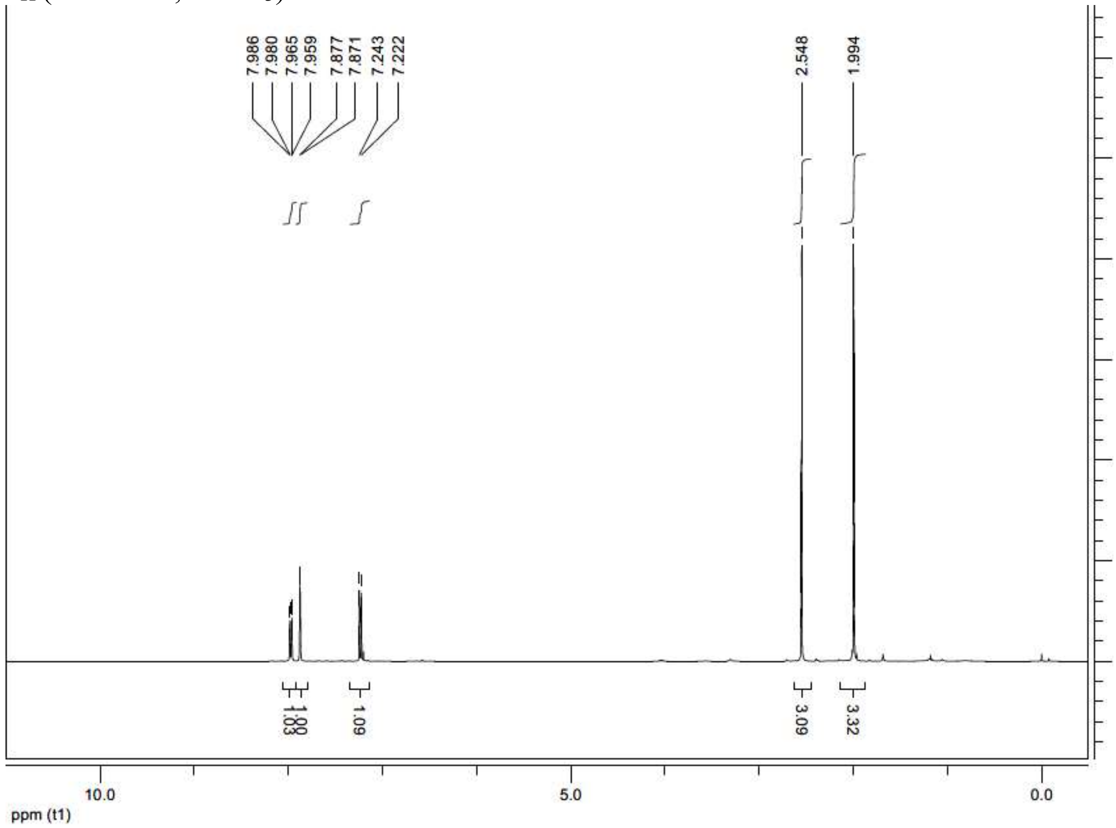

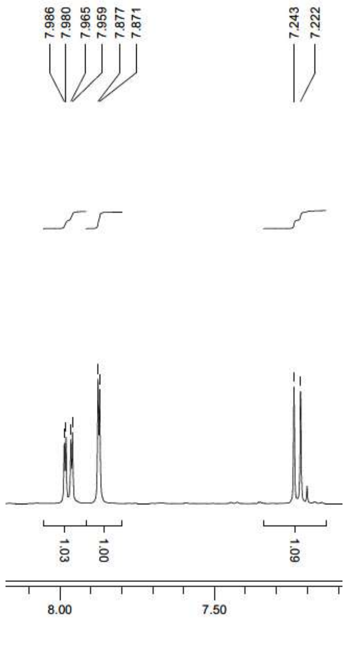

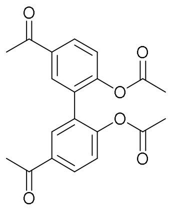

δ_C (126 MHz, $CDCl_3$)t

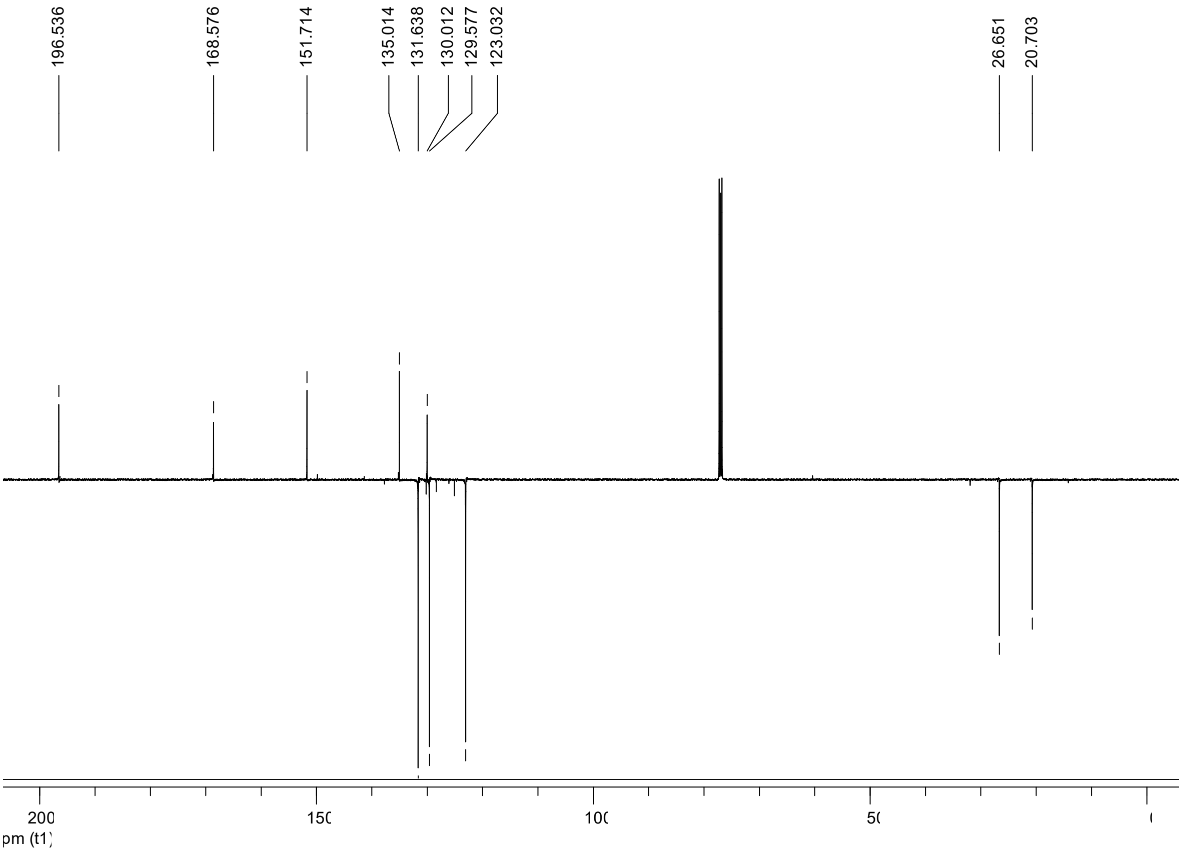

Compound 19.

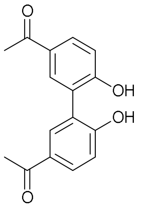

δ_H (500 MHz, d[6]-DMSO)

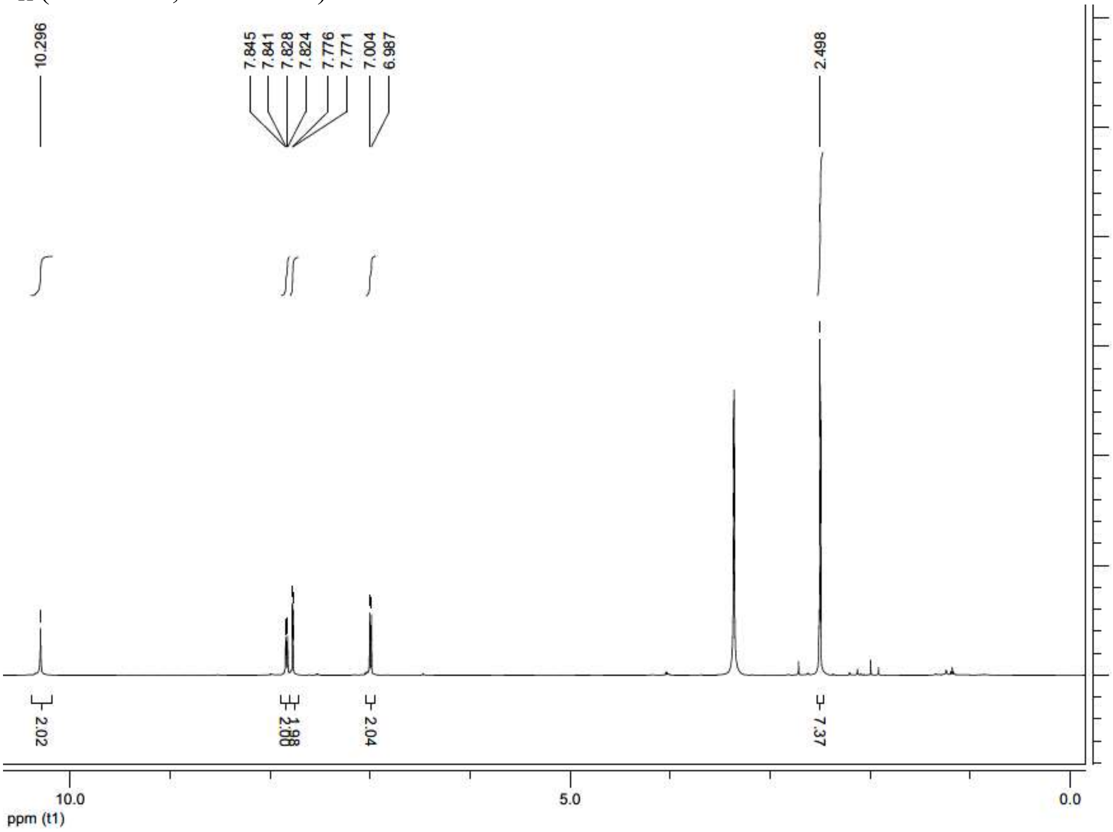

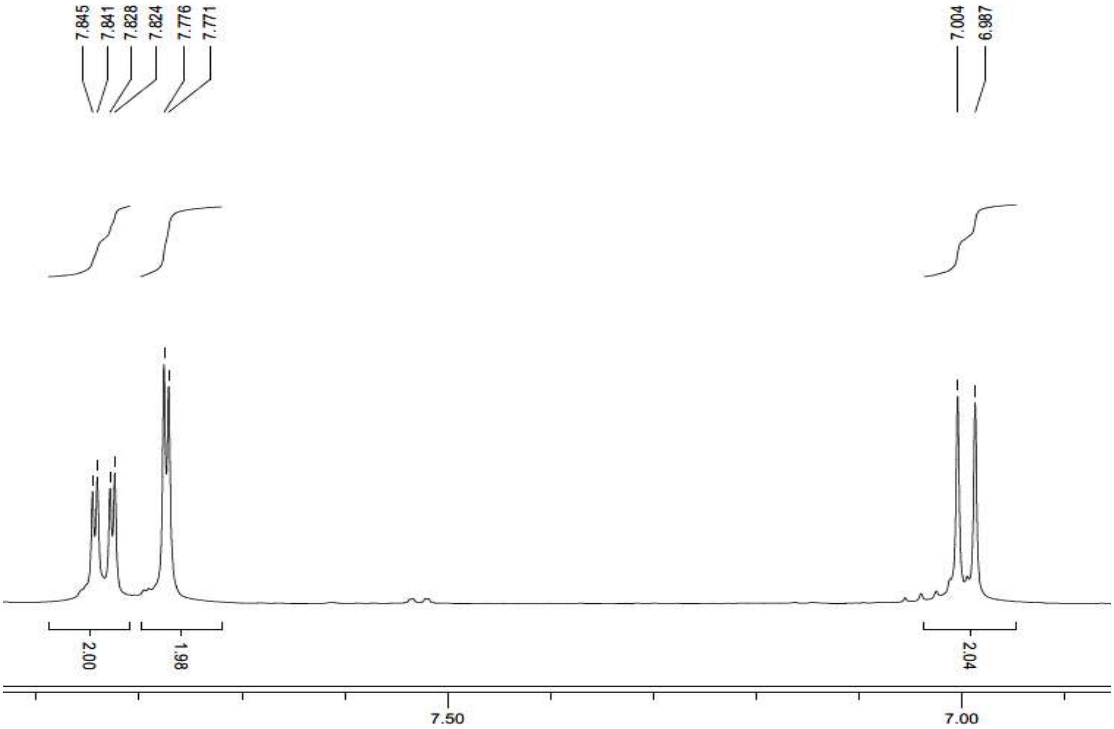

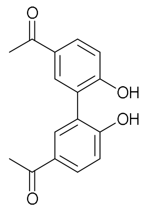

δ$_C$ (126 MHz, d^6-DMSO)

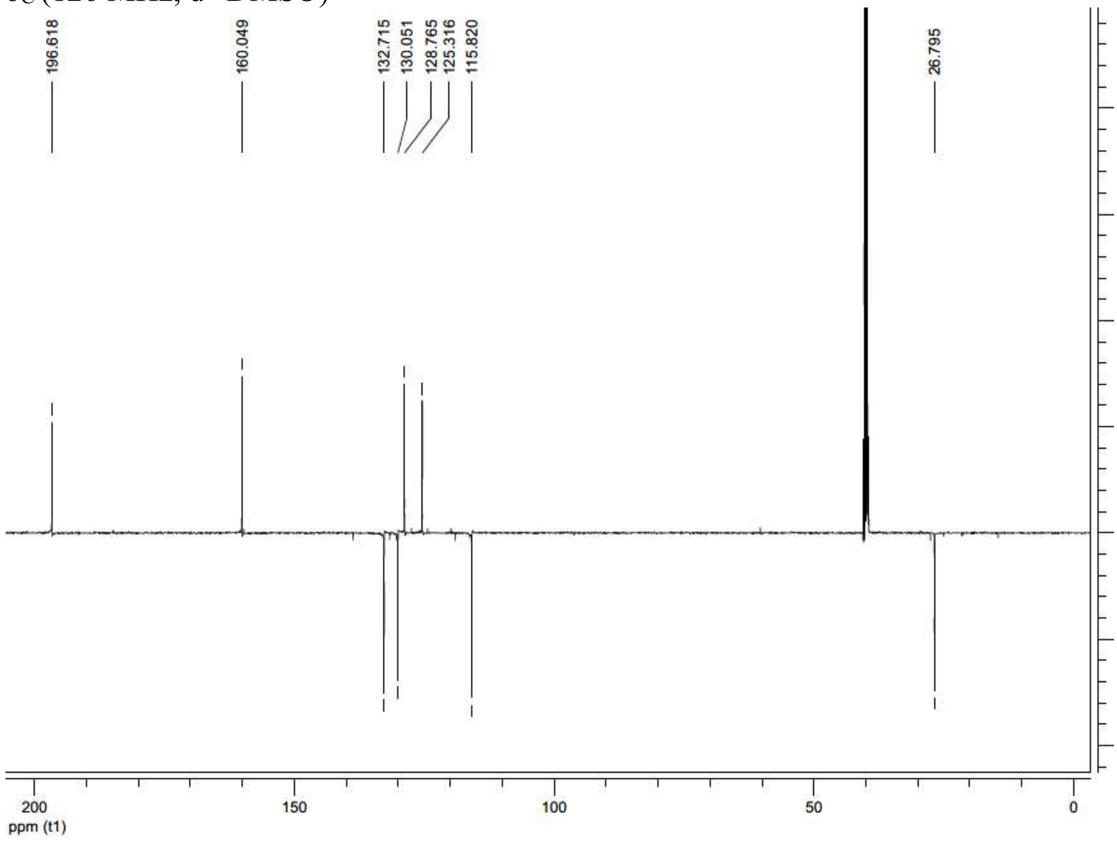

Compound 21.

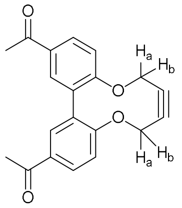

δ_H (500 MHz, $CDCl_3$)

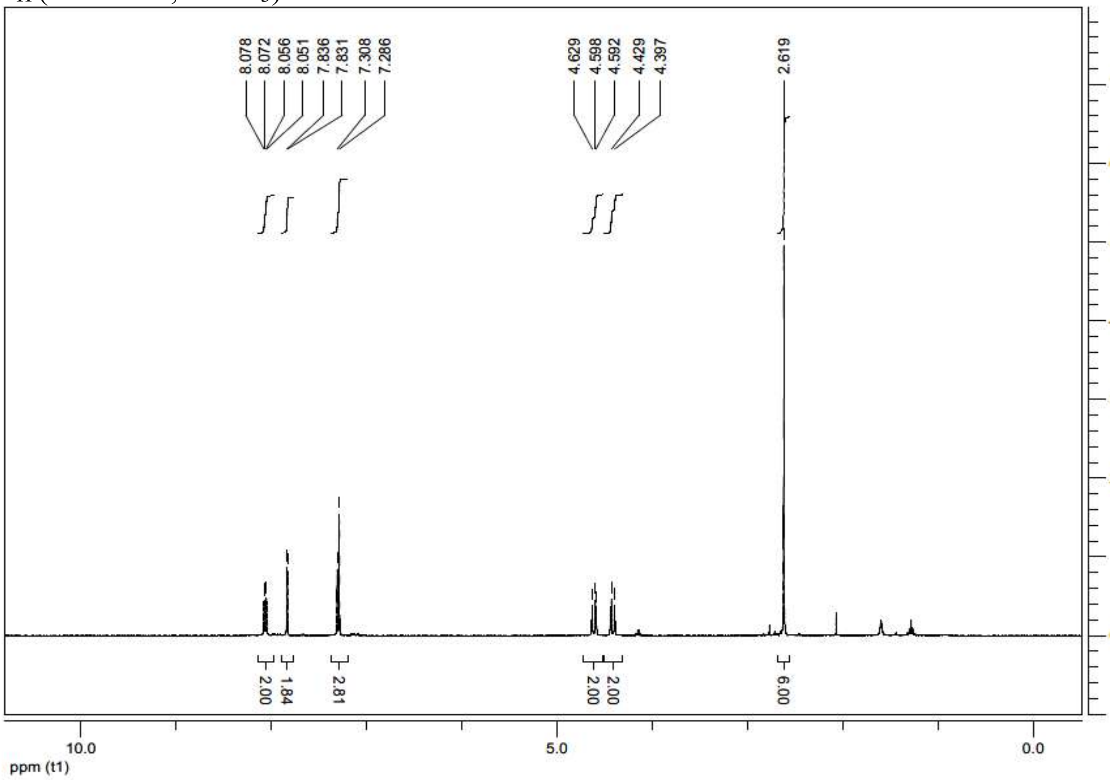

There is a small amount of EtOAc in the spectrum.

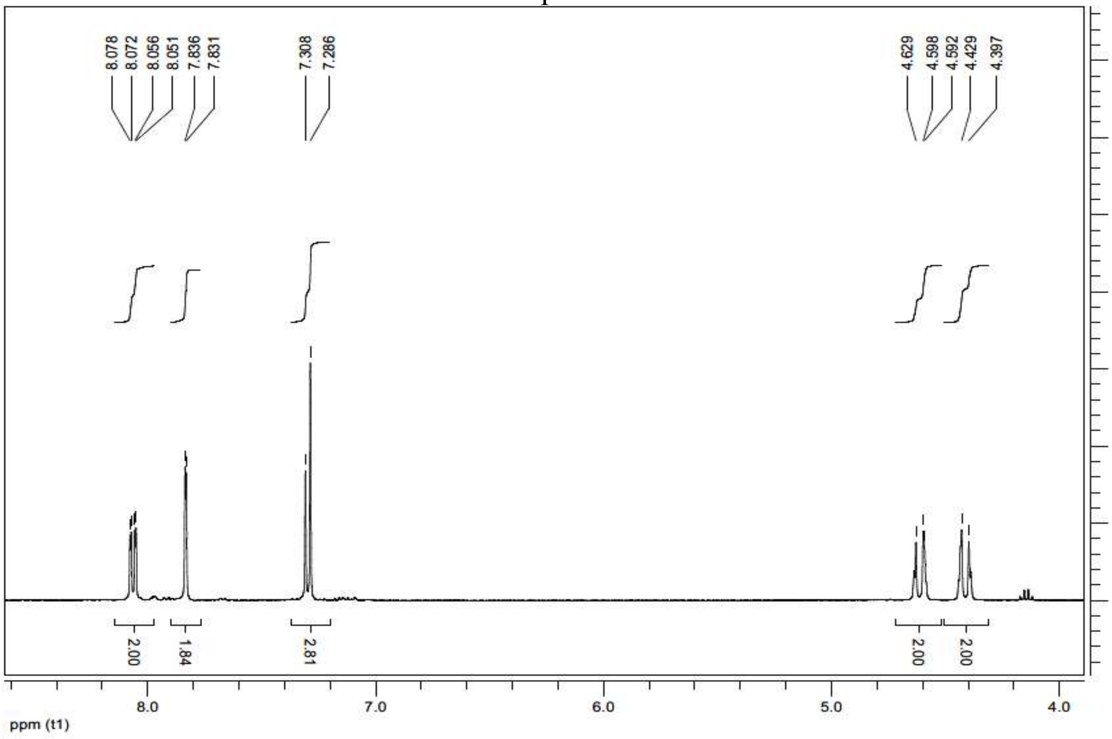

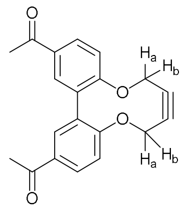

δC (126 MHz, $CDCl_3$)

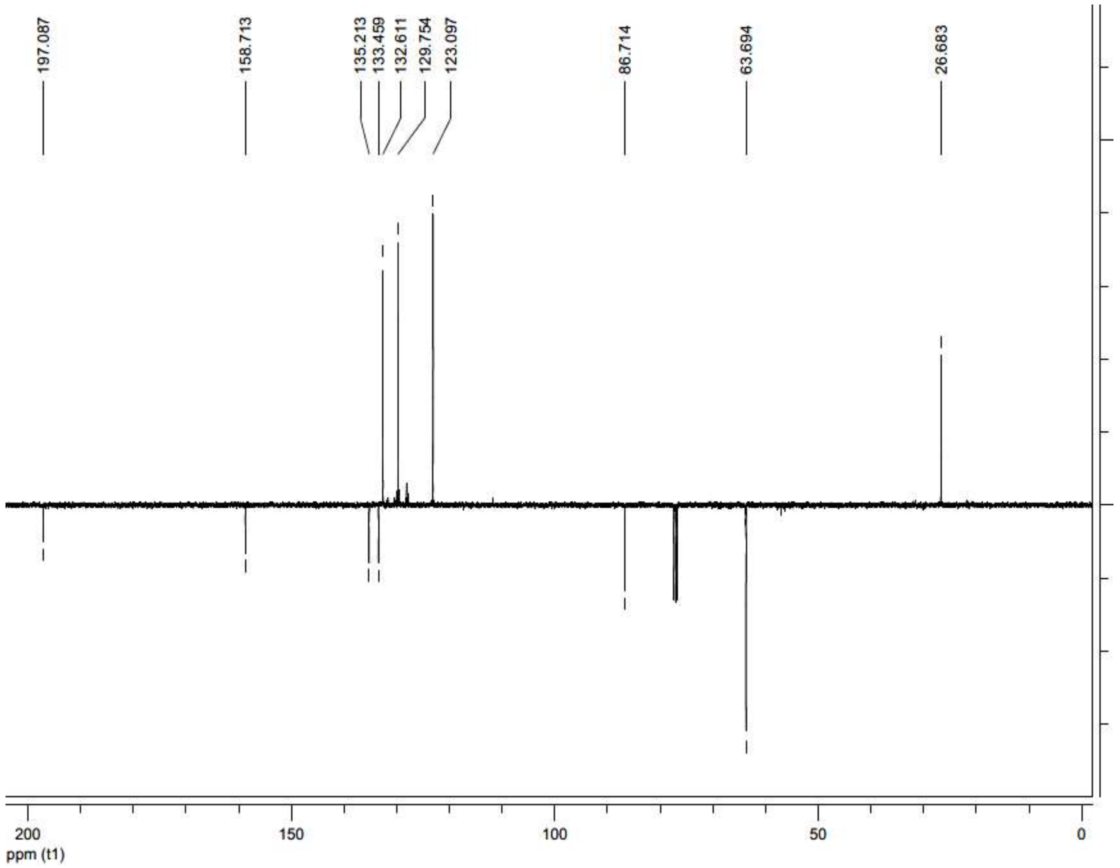

Dichalcone 22.

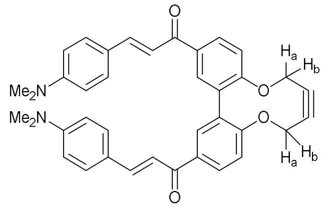

δ_H (500 MHz, $CDCl_3$)

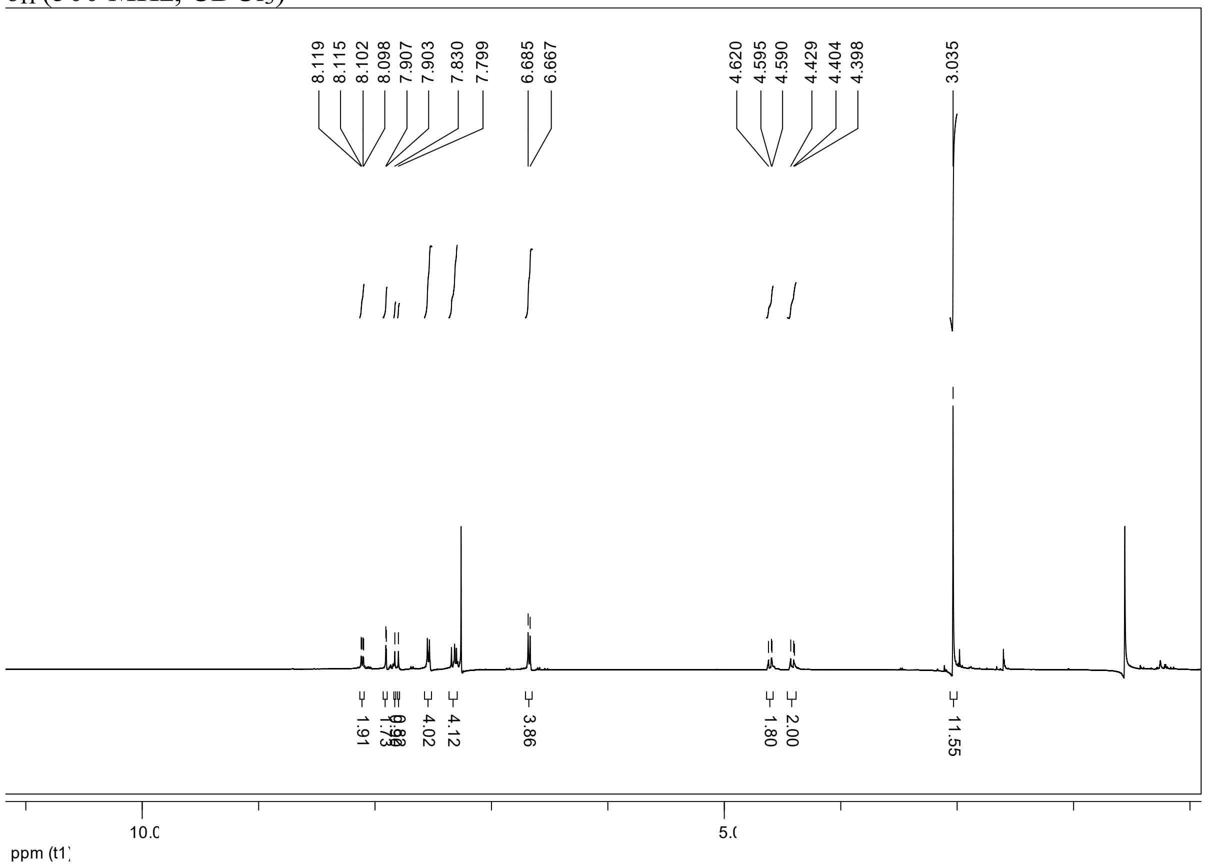

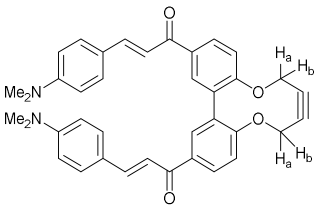

δC (126 MHz, $CDCl_3$)

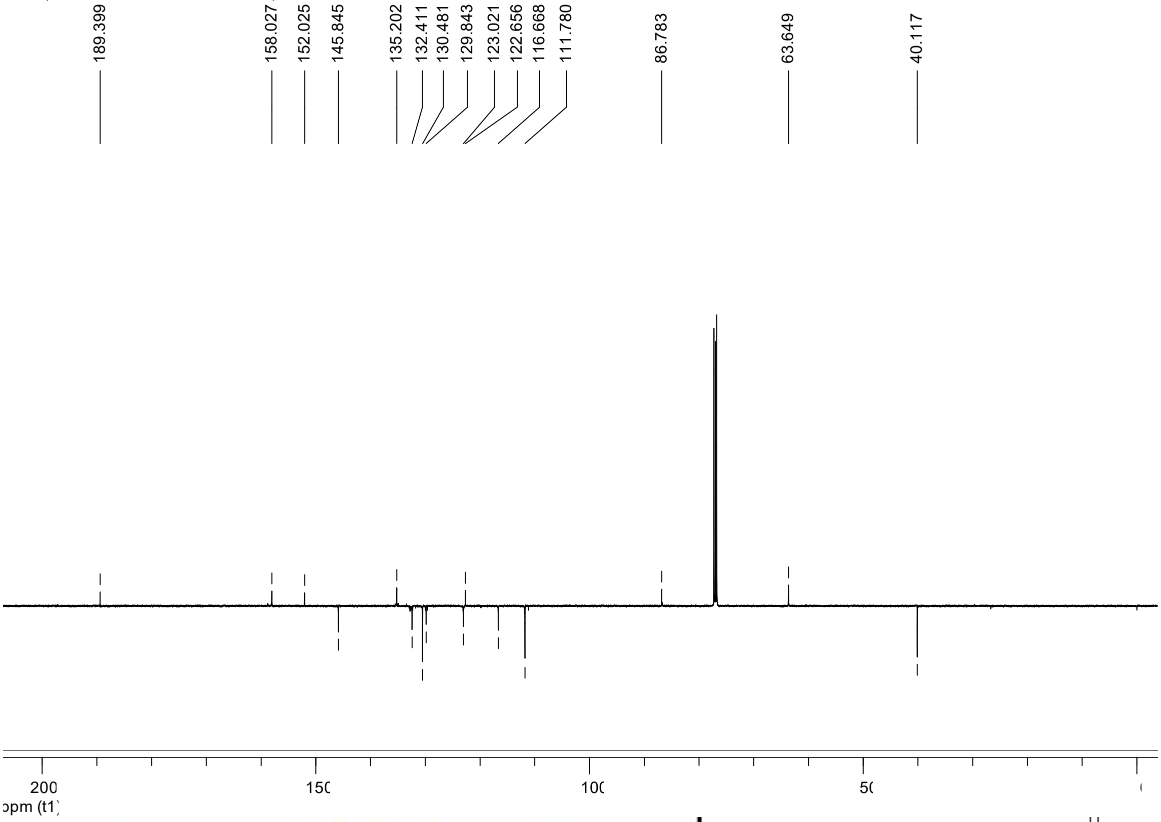

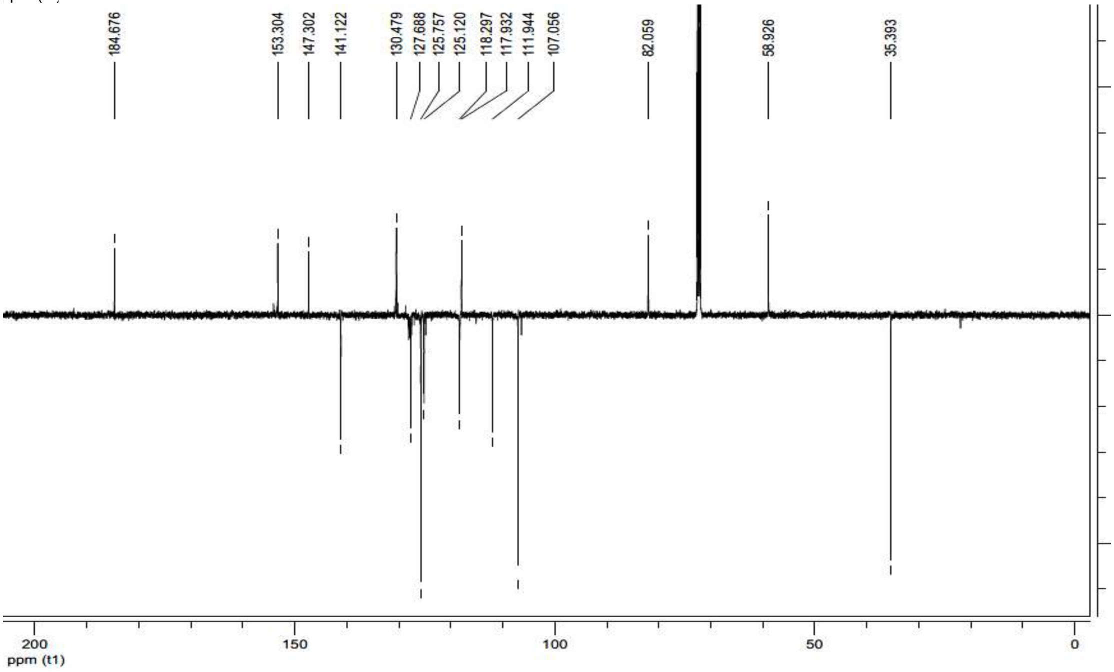

Dichalcone azide adduct 23.

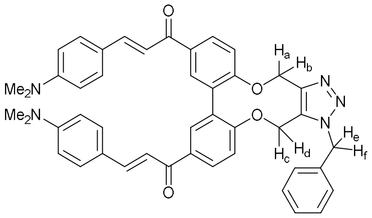

δH (500 MHz, $CDCl_3$)

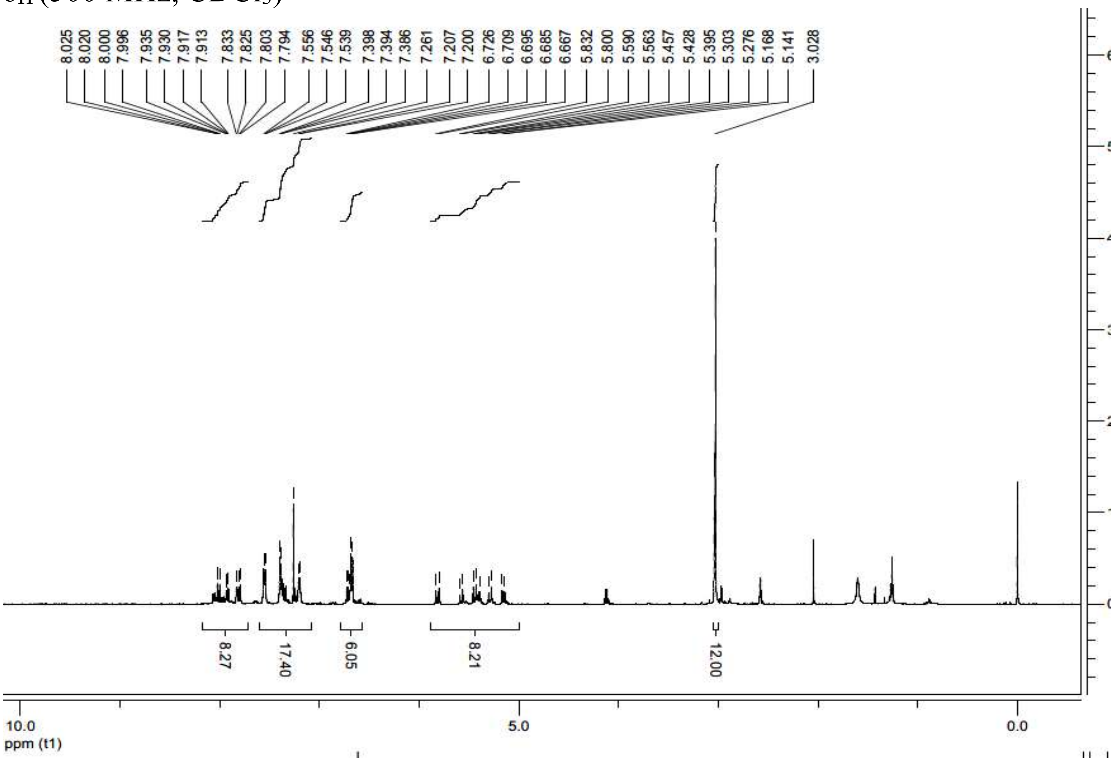

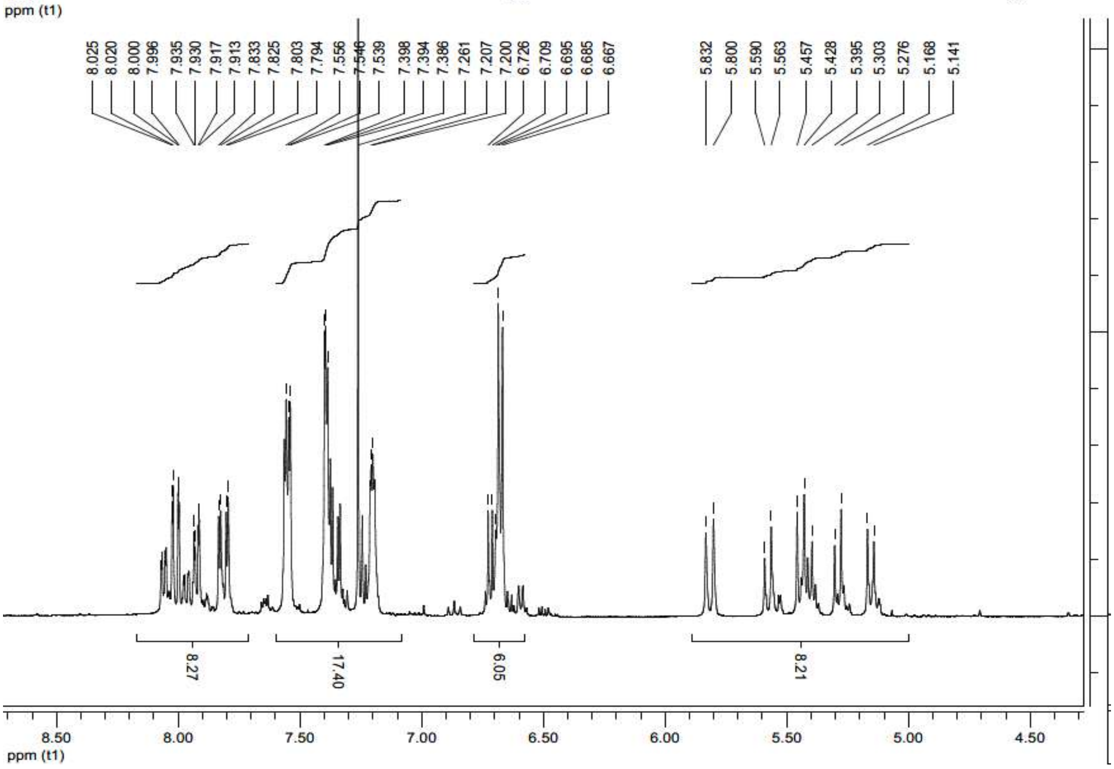

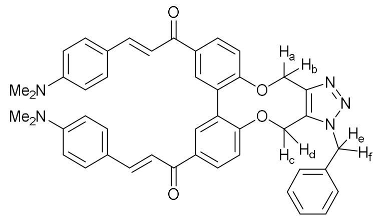

δ_c (126 MHz, $CDCl_3$)

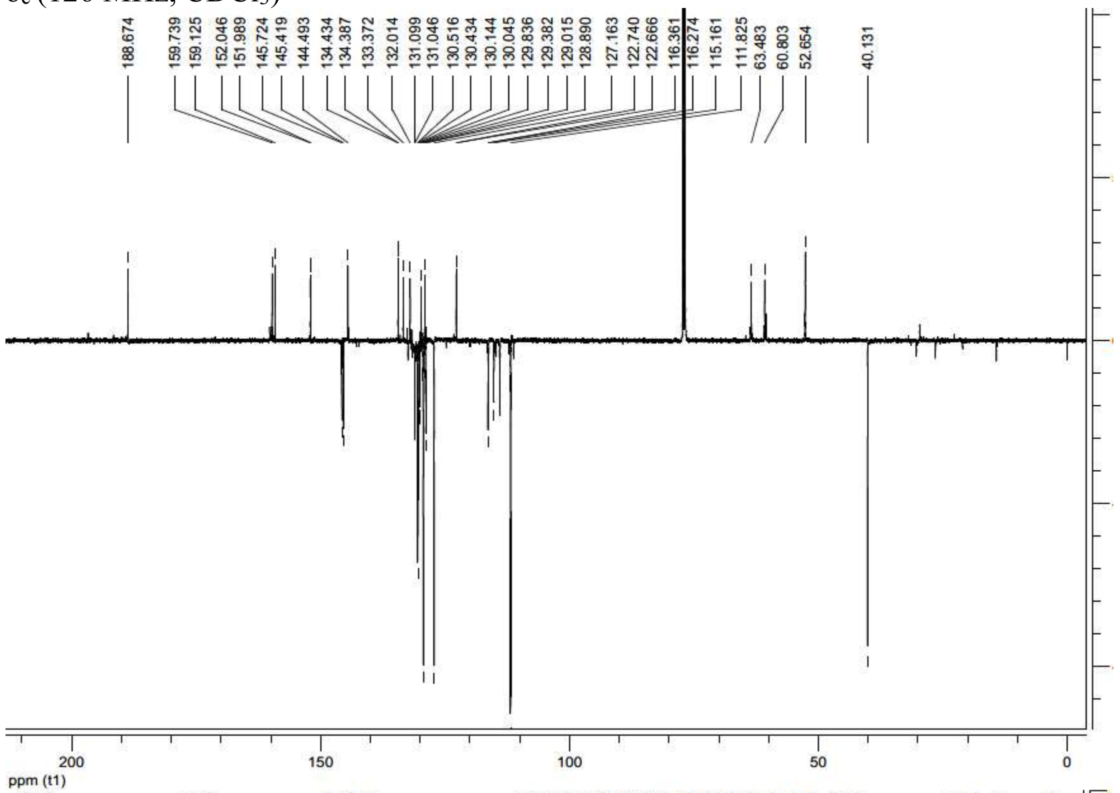

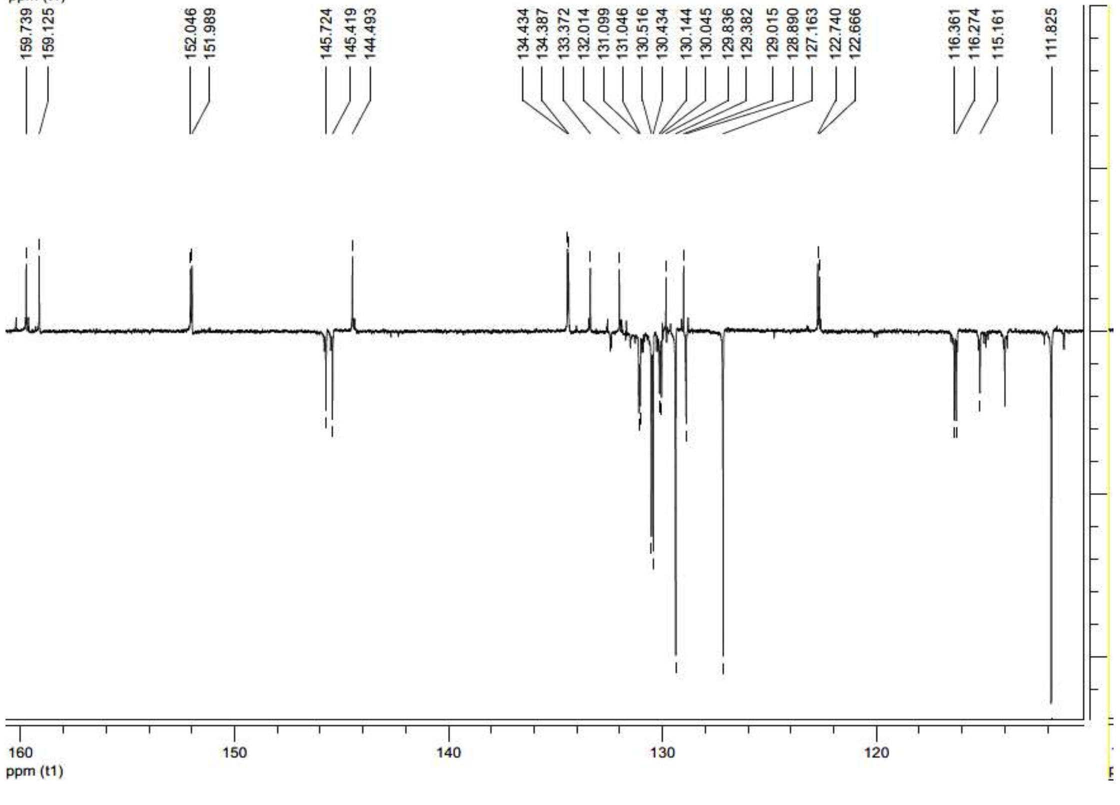

Compound 24.

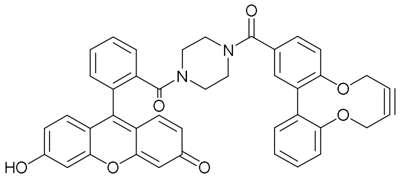

^{1}**H NMR** spectrum (500 MHz, CD_3OD)

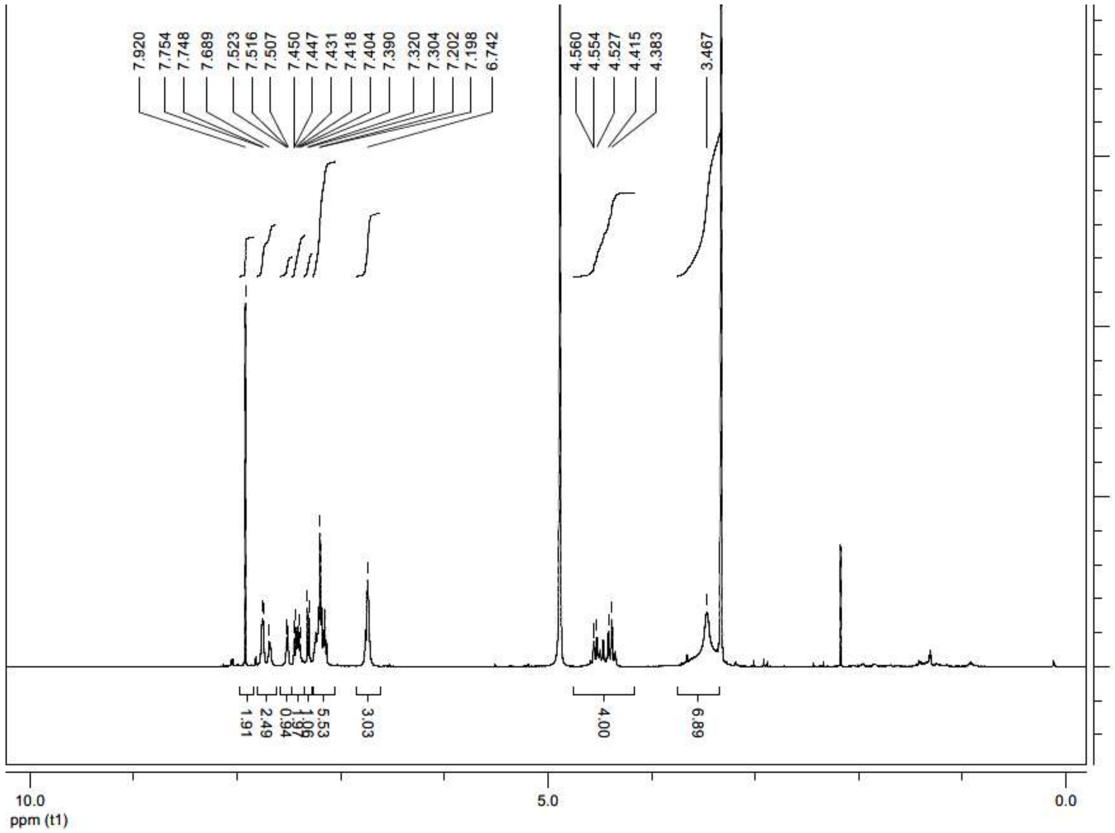

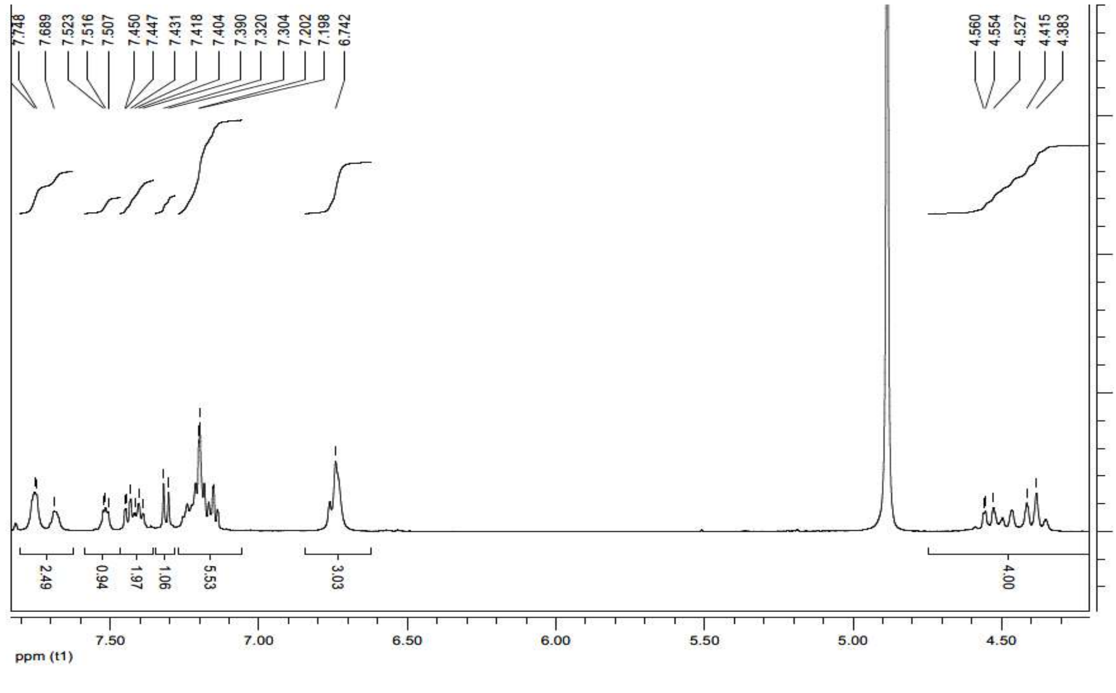

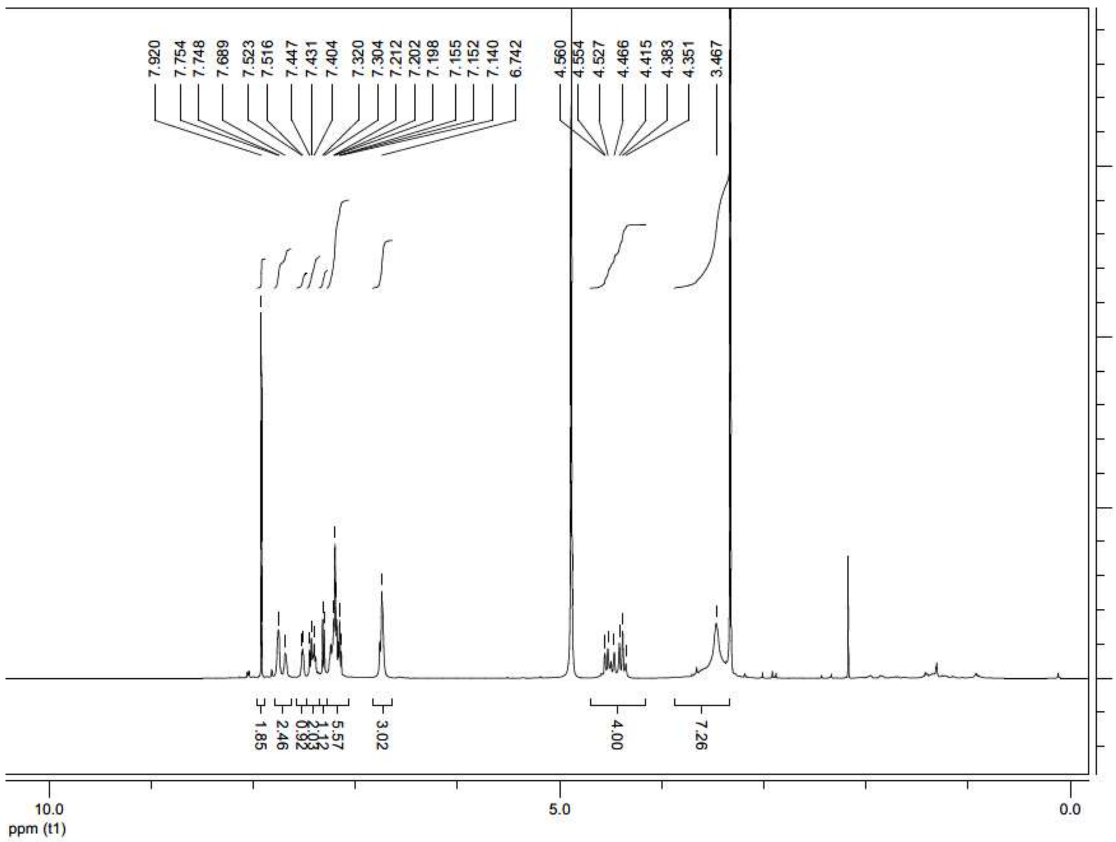
7.920
7.754
7.748
7.689
7.523
7.516
7.447
7.431
7.404
7.320
7.304
7.212
7.202
7.198
7.155
7.152
7.140
6.742
4.560
4.554
4.527
4.466
4.415
4.383
4.351
3.467
1.85
2.46
0.92
2.03
1.12
5.57
3.02
4.00
7.26
10.0
5.0
0.0
ppm (t1)

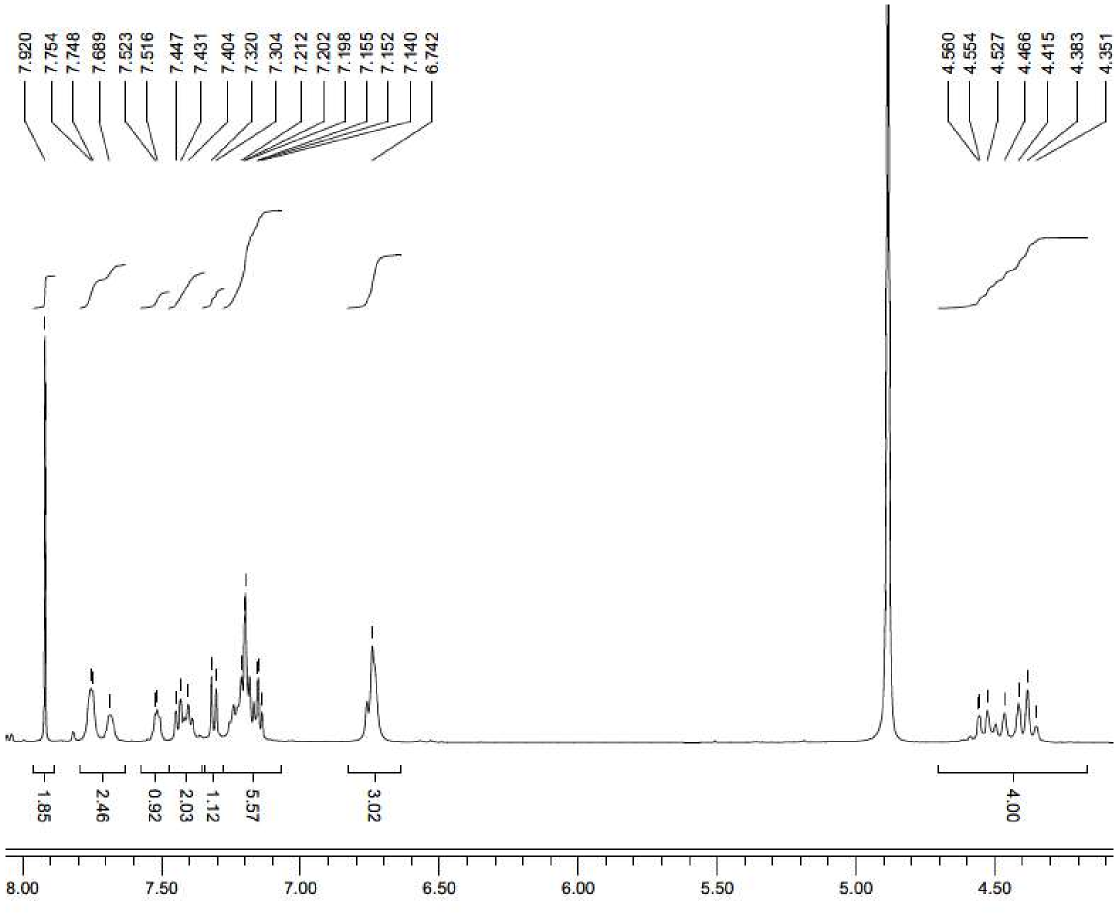
7.920
7.754
7.748
7.689
7.523
7.516
7.447
7.431
7.404
7.320
7.304
7.212
7.202
7.198
7.155
7.152
7.140
6.742
4.560
4.554
4.527
4.466
4.415
4.383
4.351
1.85
2.46
0.92
2.03
1.12
5.57
3.02
4.00
8.00
7.50
7.00
6.50
6.00
5.50
5.00
4.50

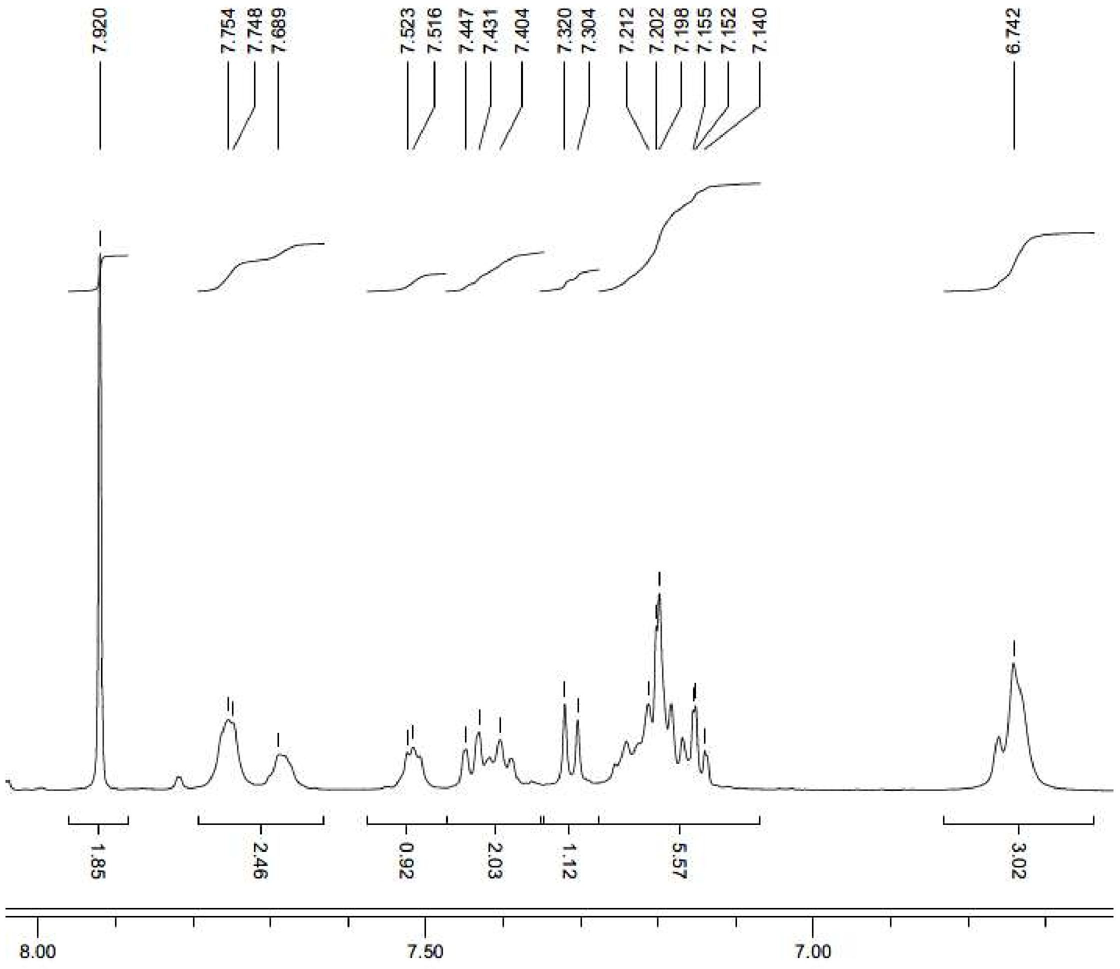
7.920
7.754
7.748
7.689
7.523
7.516
7.447
7.431
7.404
7.320
7.304
7.212
7.202
7.198
7.155
7.152
7.140
6.742
1.85
2.46
0.92
2.03
1.12
5.57
3.02
8.00
7.50
7.00

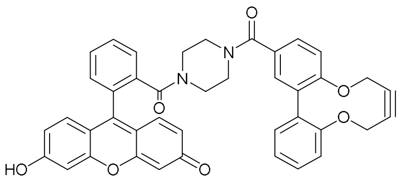

^{13}C NMR spectrum (126 MHz, CD_3OD)

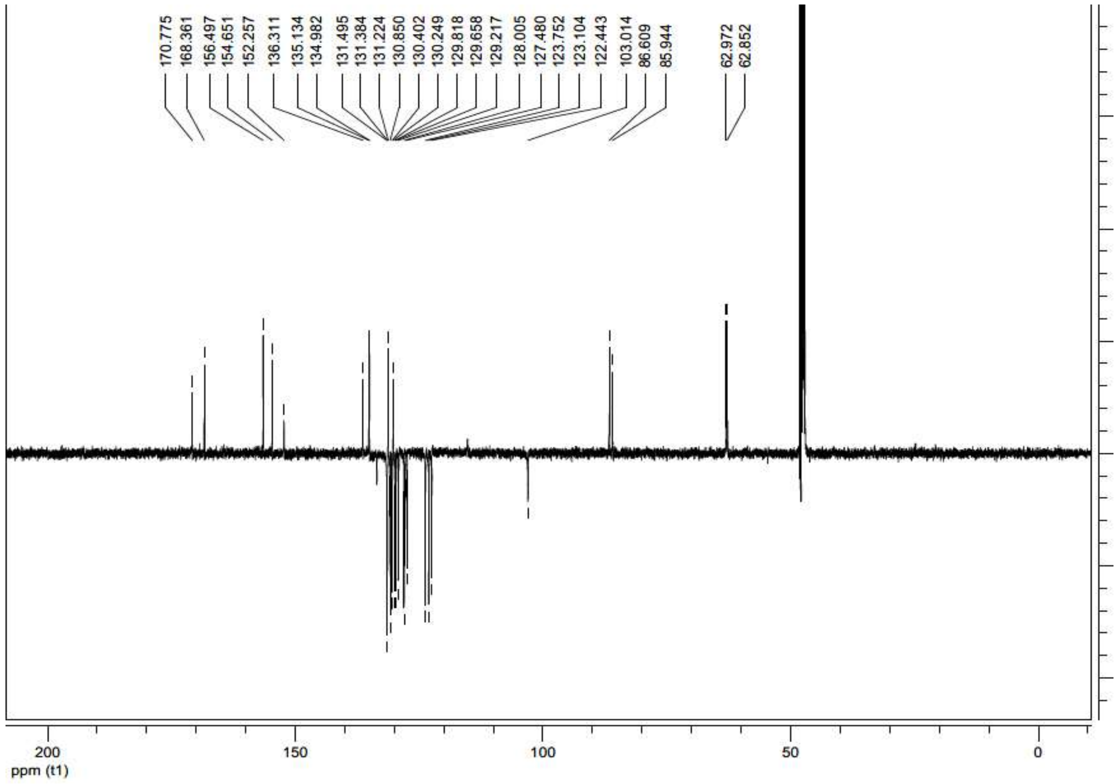

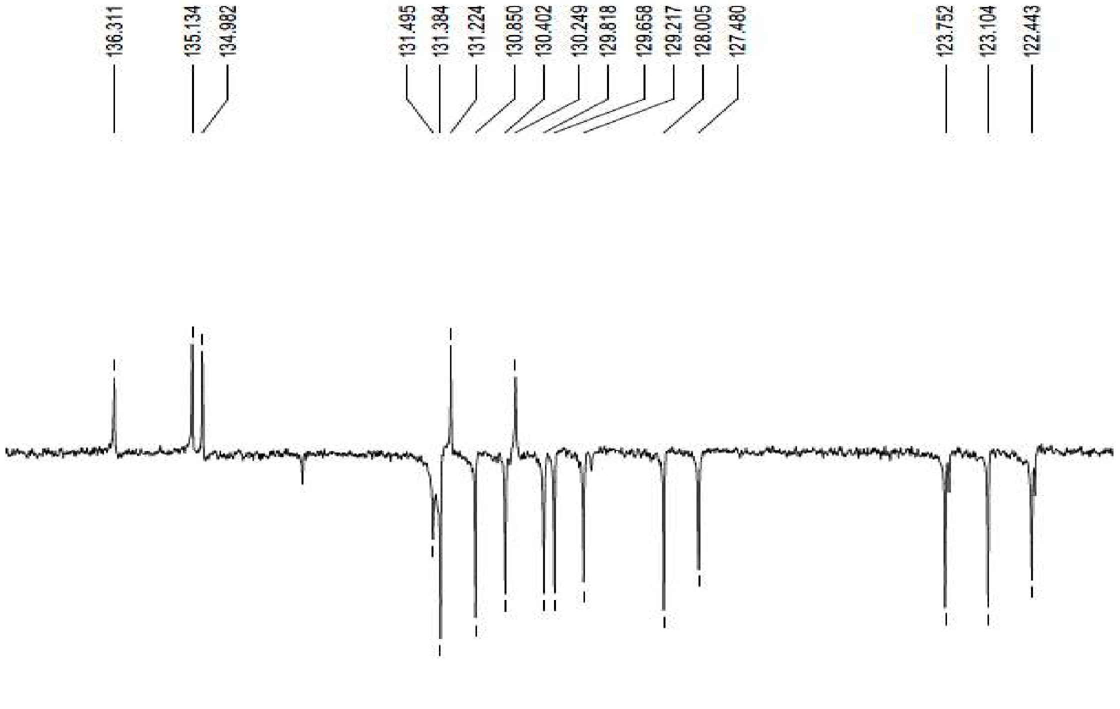

Compound 25

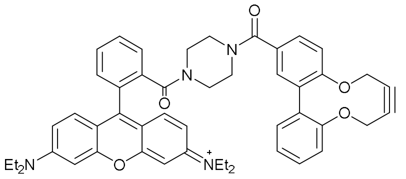

^{1}H NMR spectrum (500 MHz, CD_3OD)

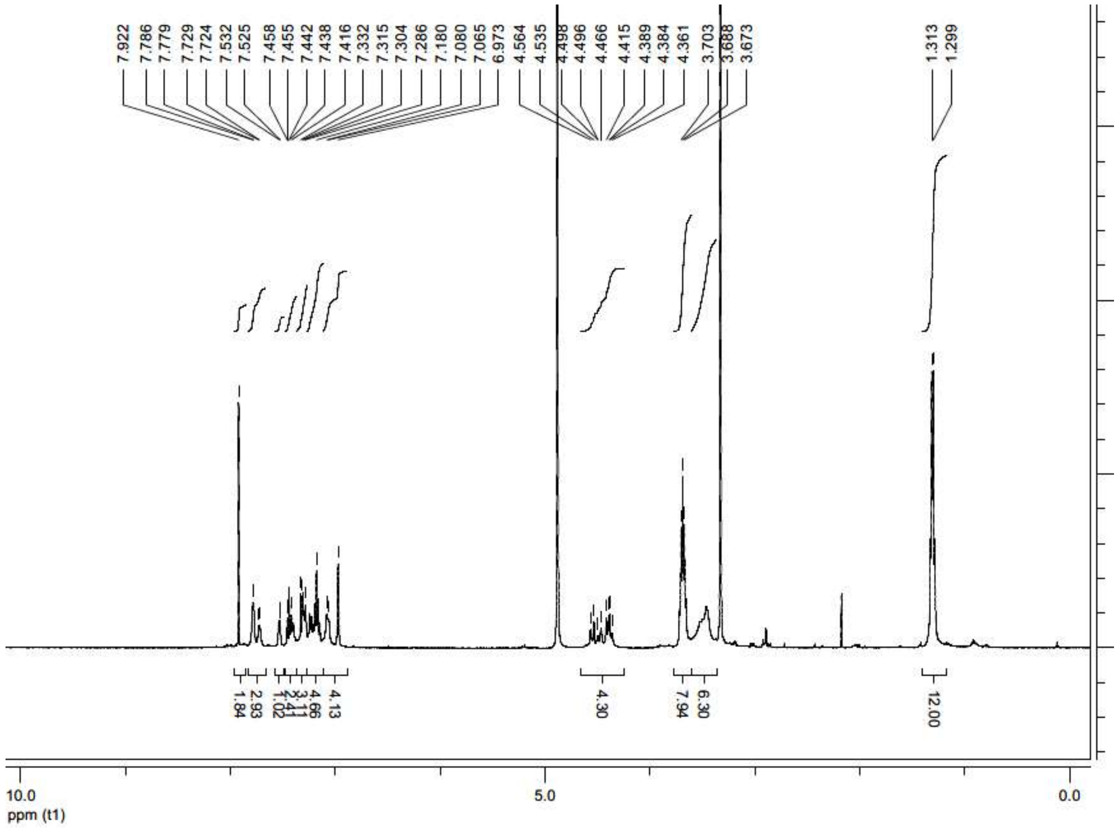

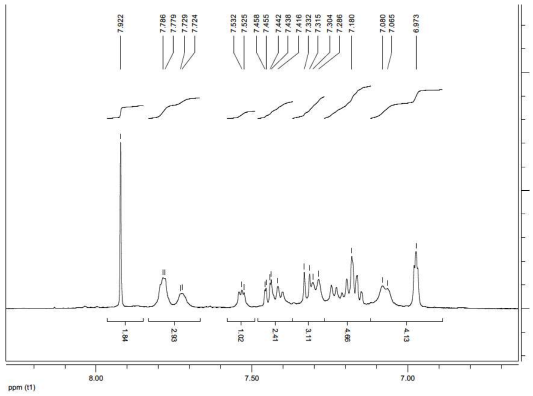

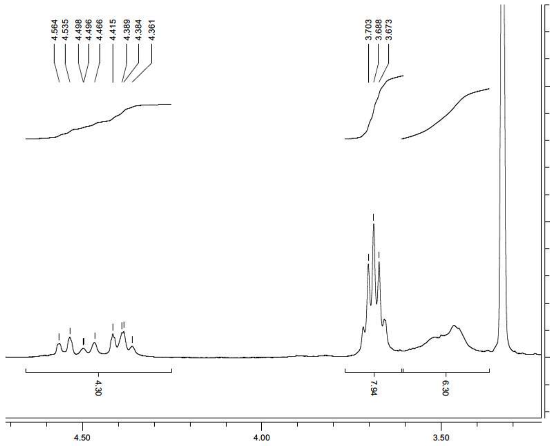

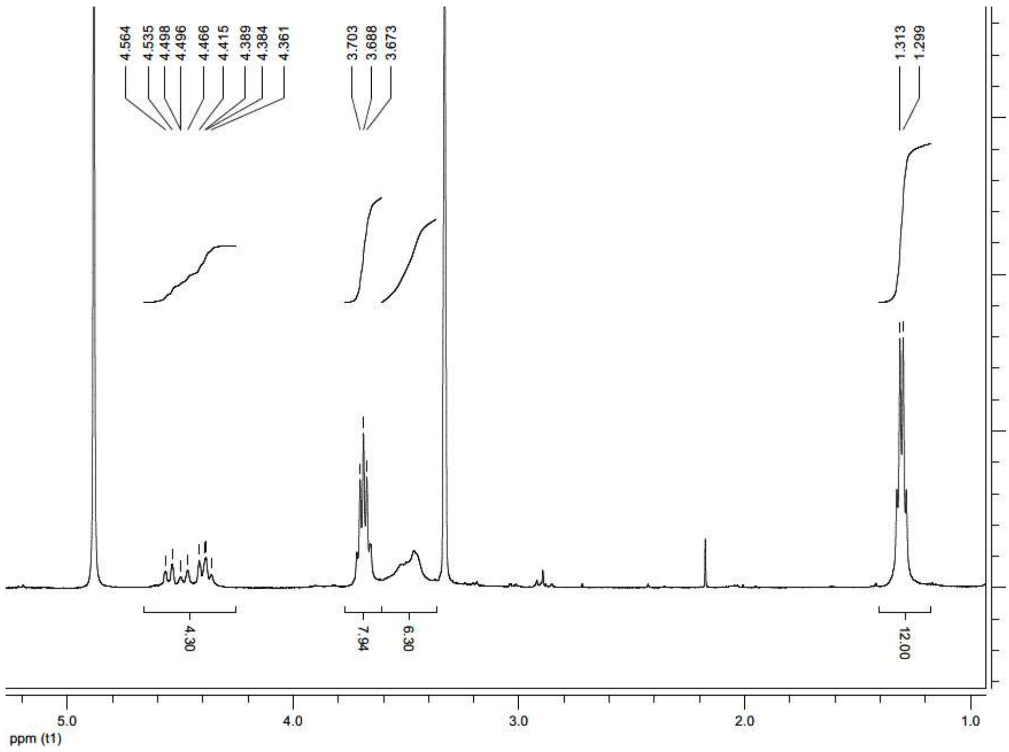

4.564
4.535
4.498
4.496
4.466
4.415
4.389
4.384
4.361
3.703
3.688
3.673
1.313
1.299
4.30
7.94
6.30
12.00
5.0
4.0
3.0
2.0
1.0
ppm (t1)

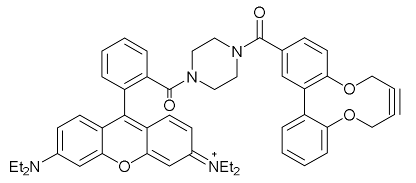

^{13}C NMR spectrum (126 MHz, CD_3OD)

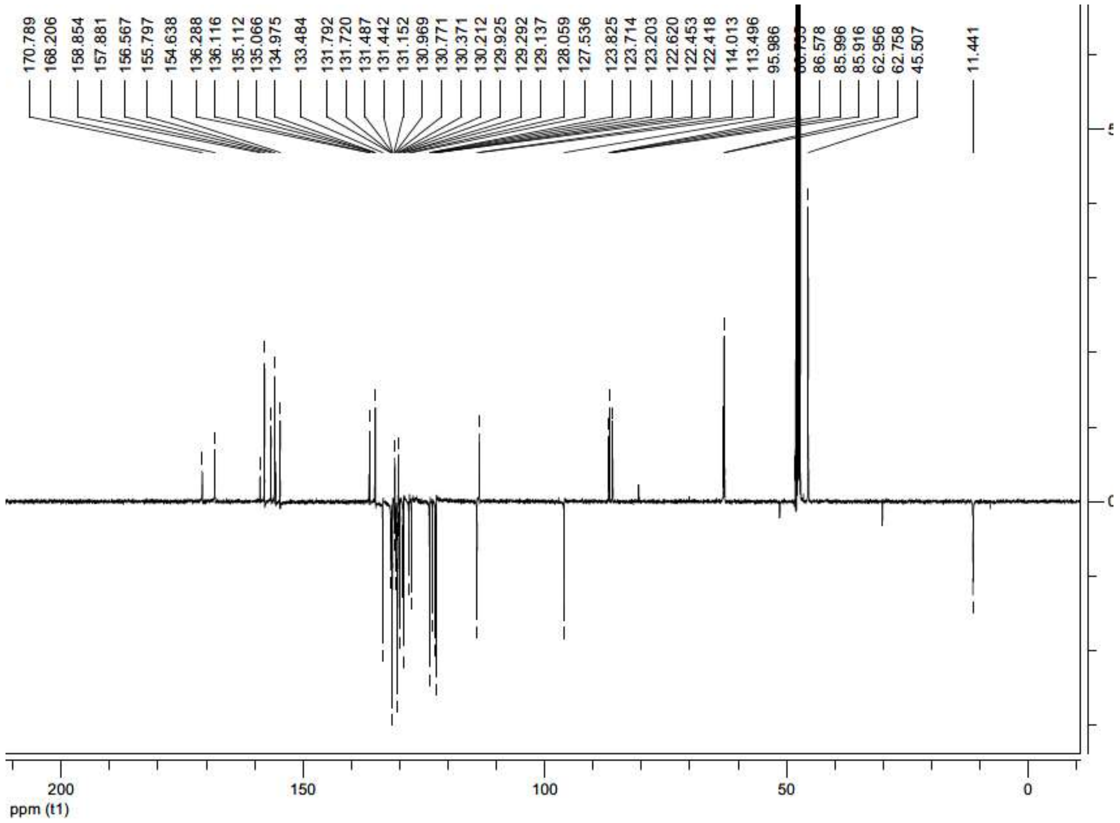

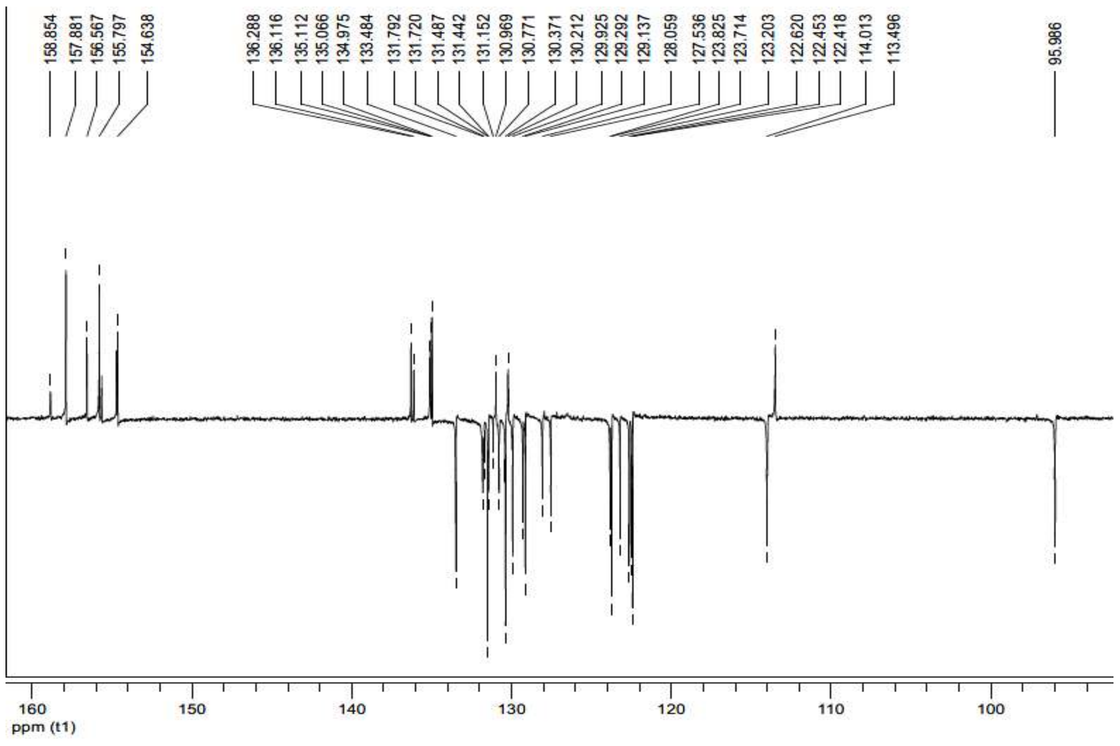

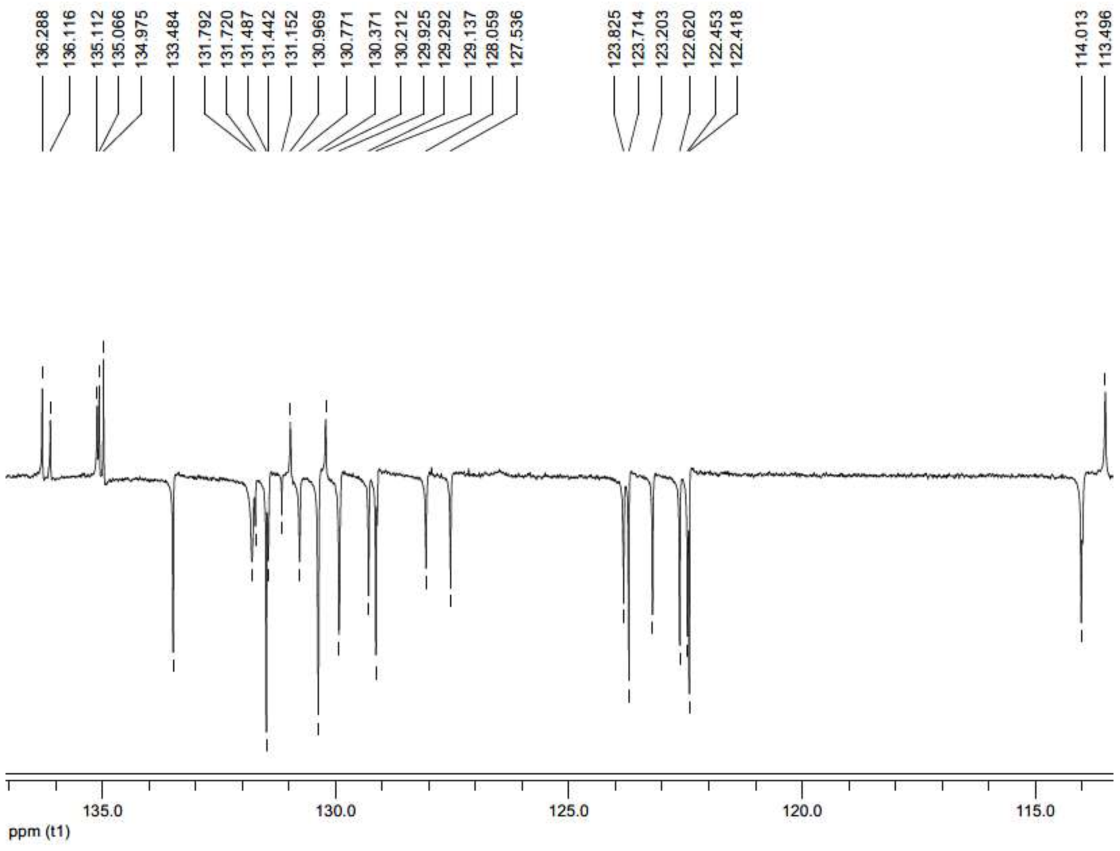
136.288
136.116
135.112
135.066
134.975
133.484
131.792
131.720
131.487
131.442
131.152
130.969
130.771
130.371
130.212
129.925
129.292
129.137
128.059
127.536
123.825
123.714
123.203
122.620
122.453
122.418
114.013
113.496
135.0
130.0
125.0
120.0
115.0
ppm (t1)

Compound 31.

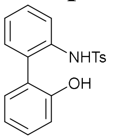

δ_H (500 MHz, $CDCl_3$)

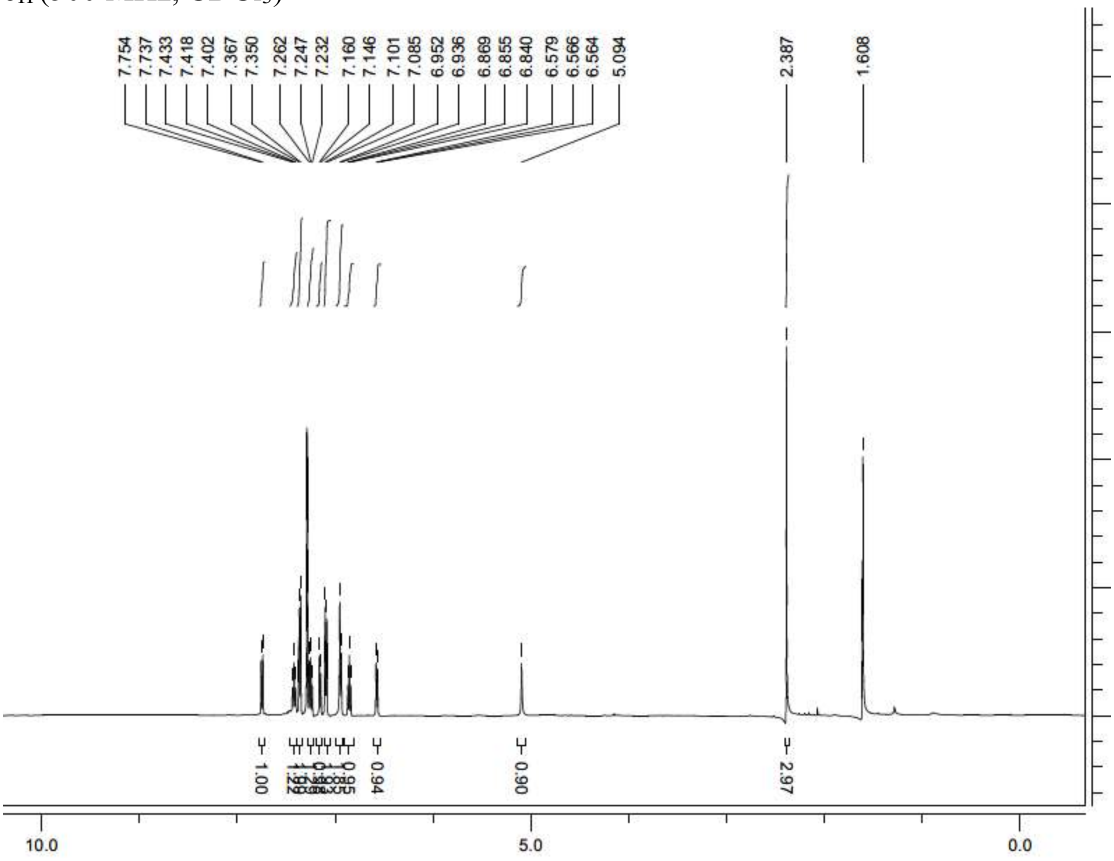

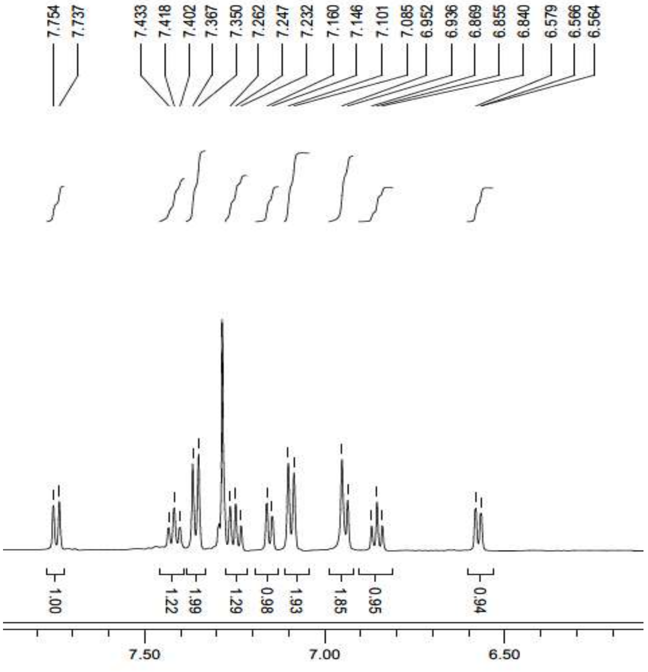

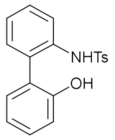

δ_C (126 MHz, $CDCl_3$)

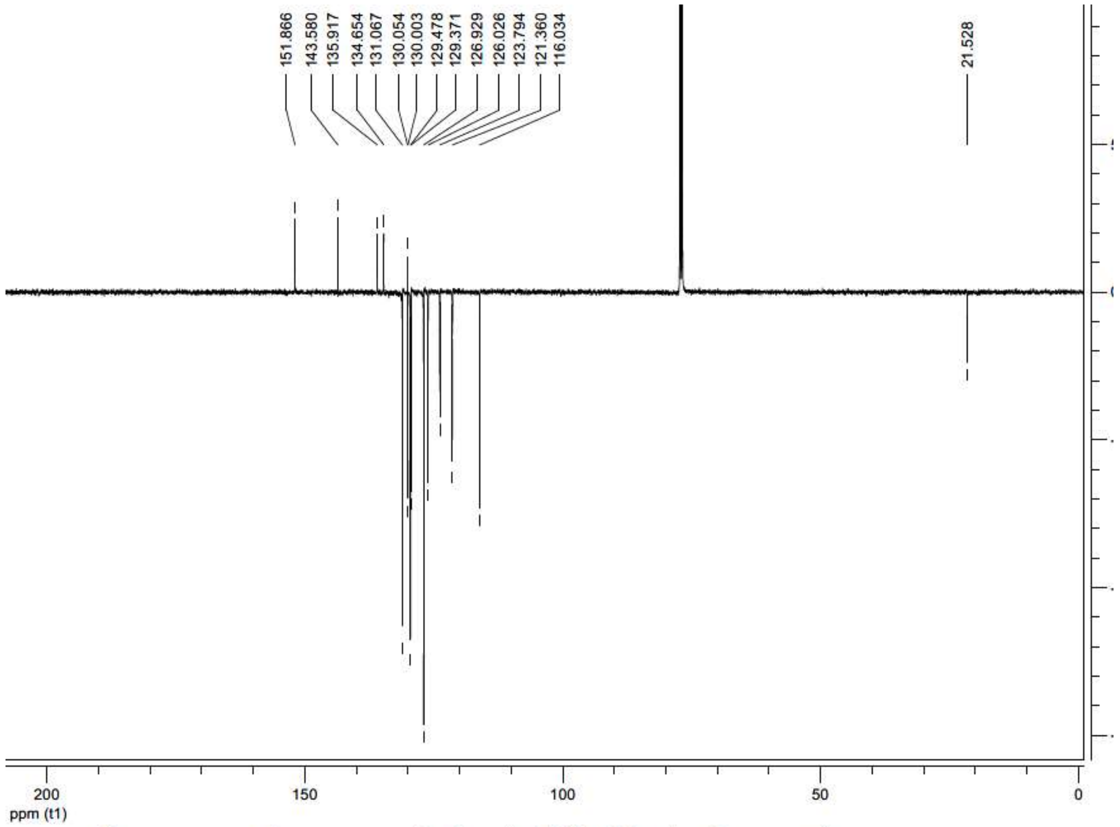

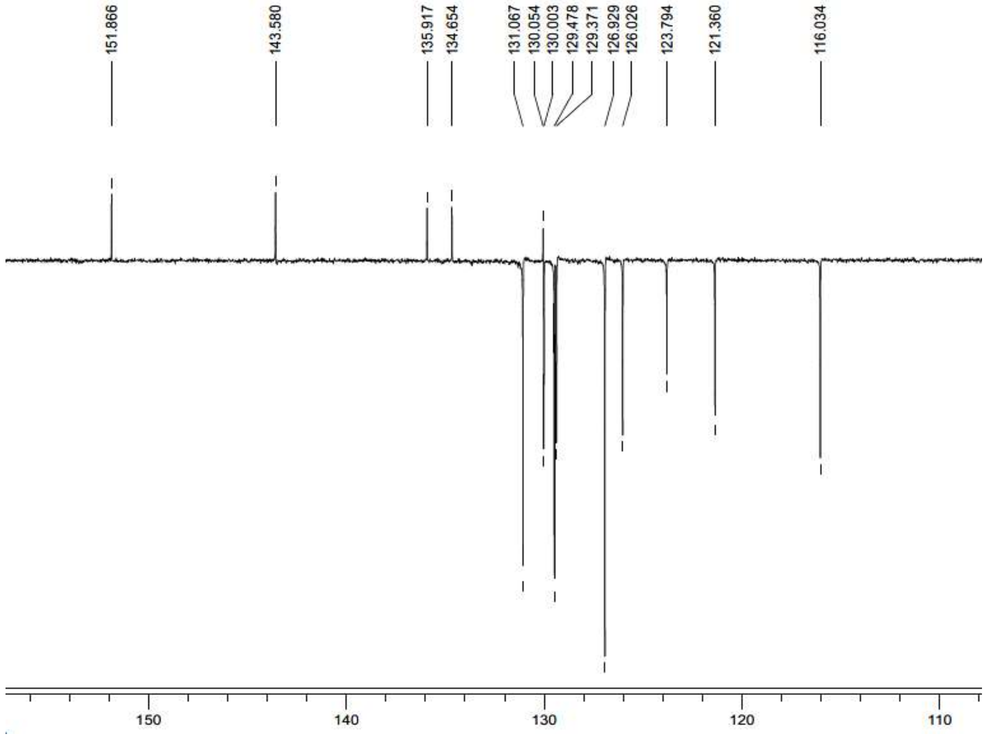

Compound 29.

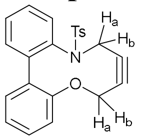

δ_H (500 MHz, $CDCl_3$)

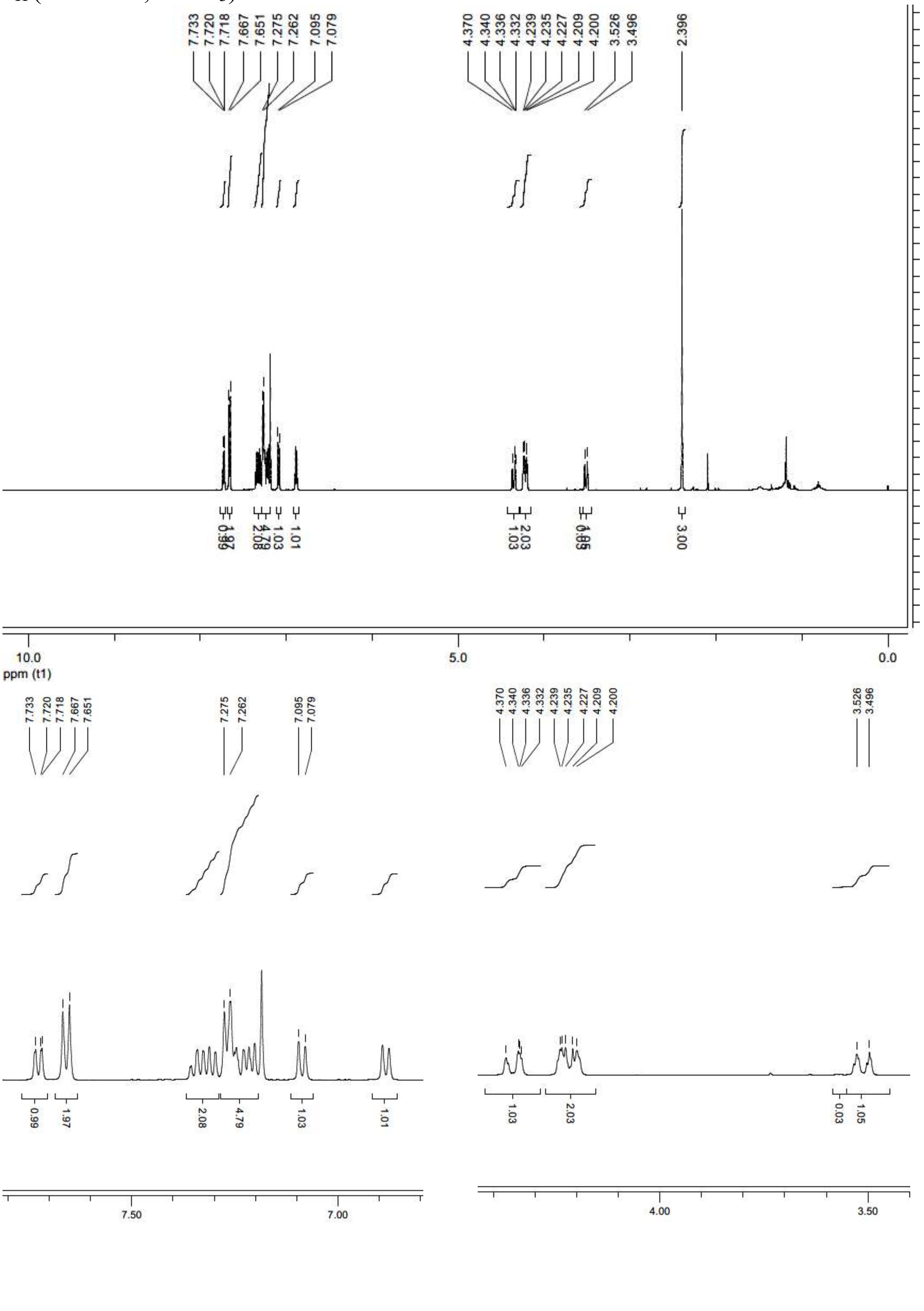

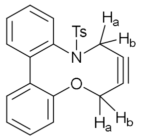

δ_C (126 MHz, $CDCl_3$)

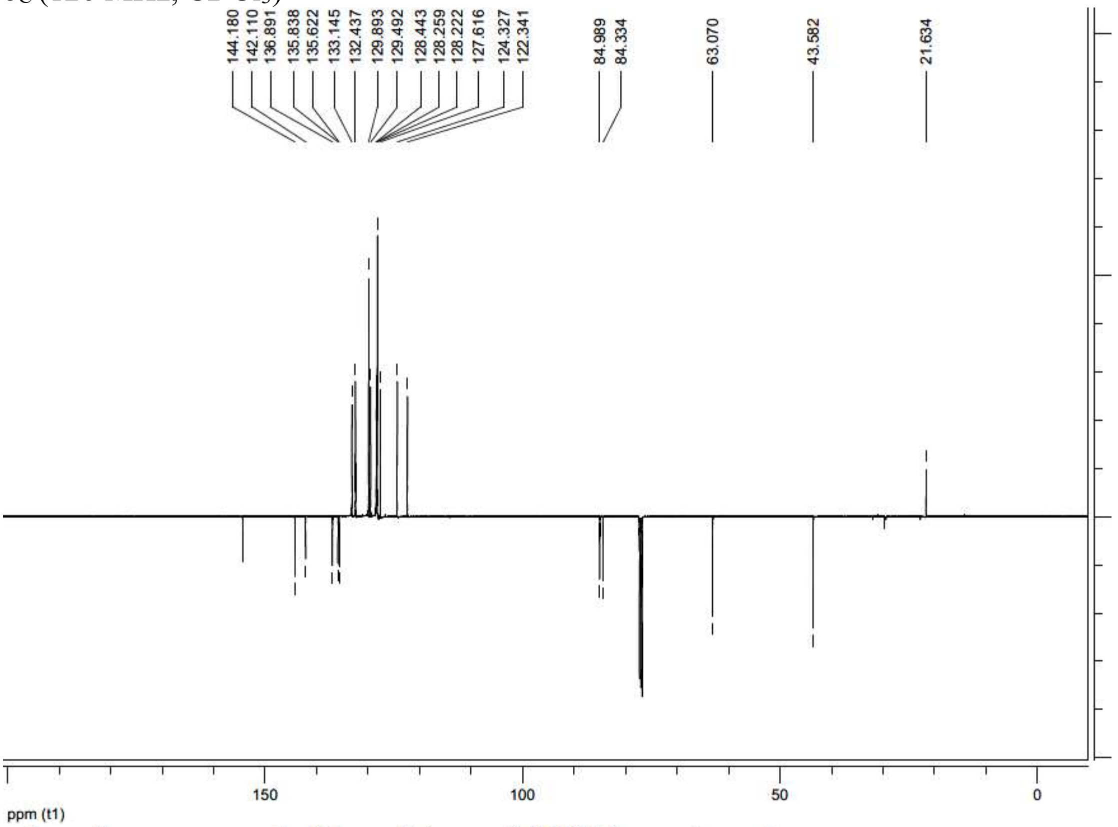

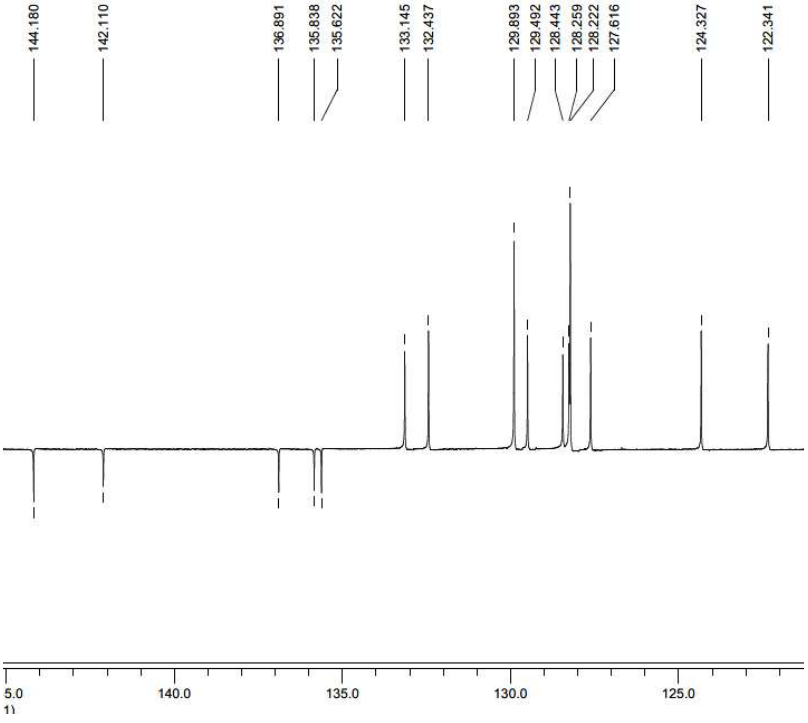

Compound 29 azide adduct 32A.

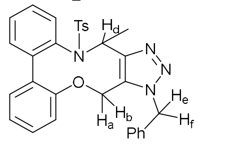

A

δ_H (500 MHz, $CDCl_3$)

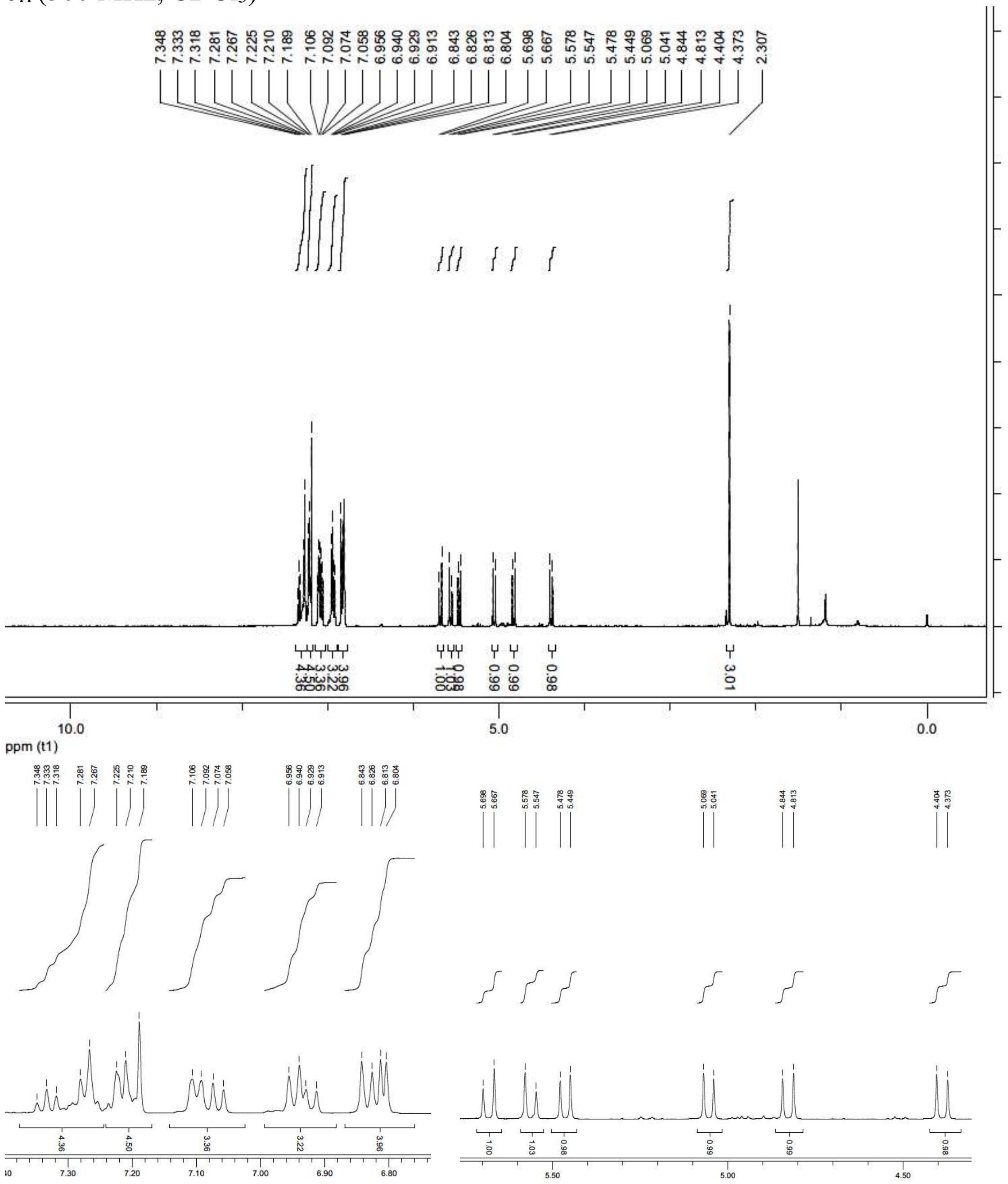

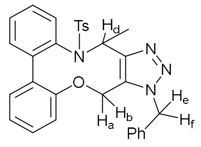

A

δC (126 MHz, CDCl3)

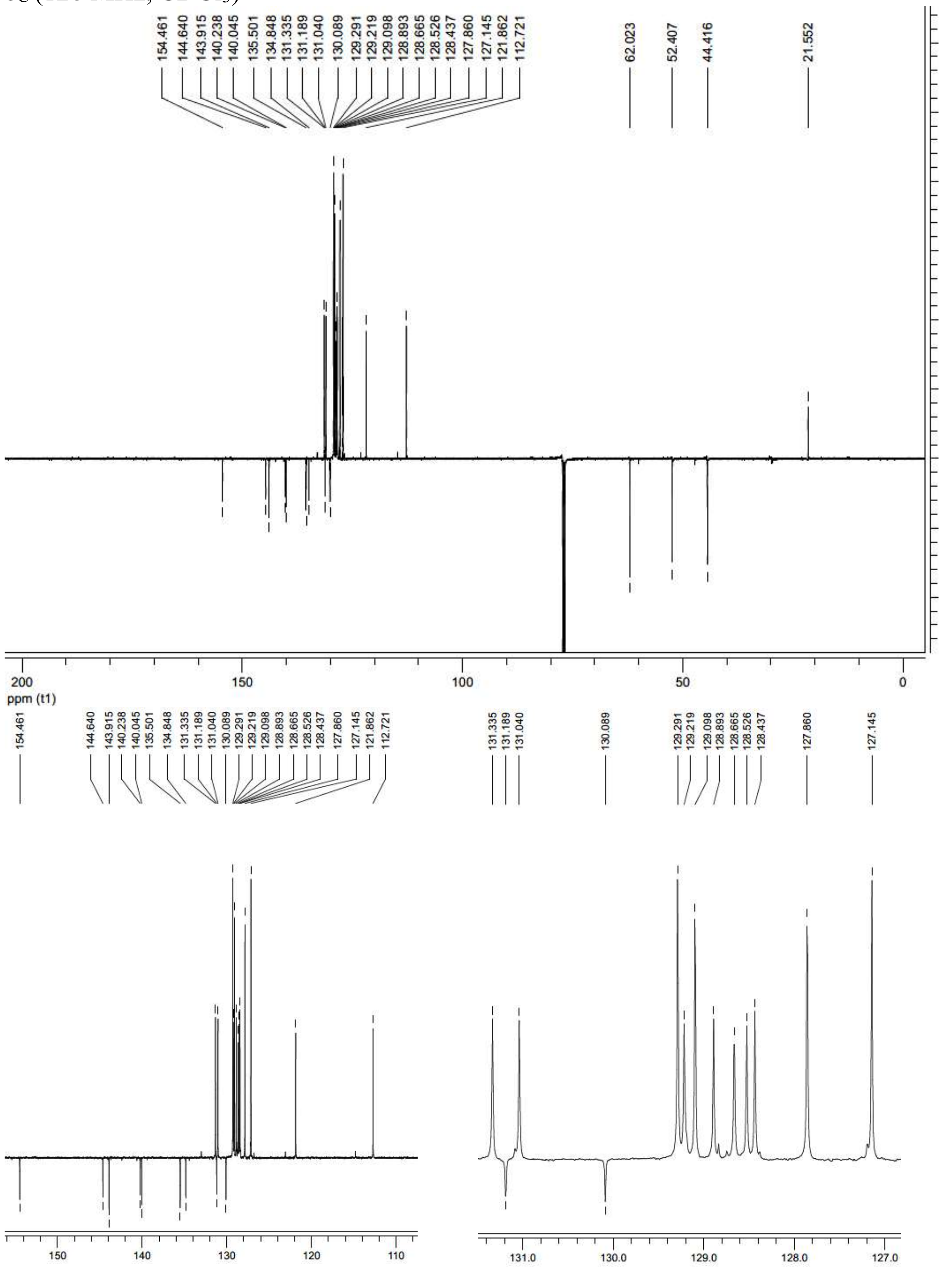

Compound 29 azide adduct 32B.

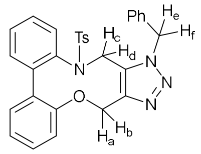

B

δ_H (500 MHz, $CDCl_3$)

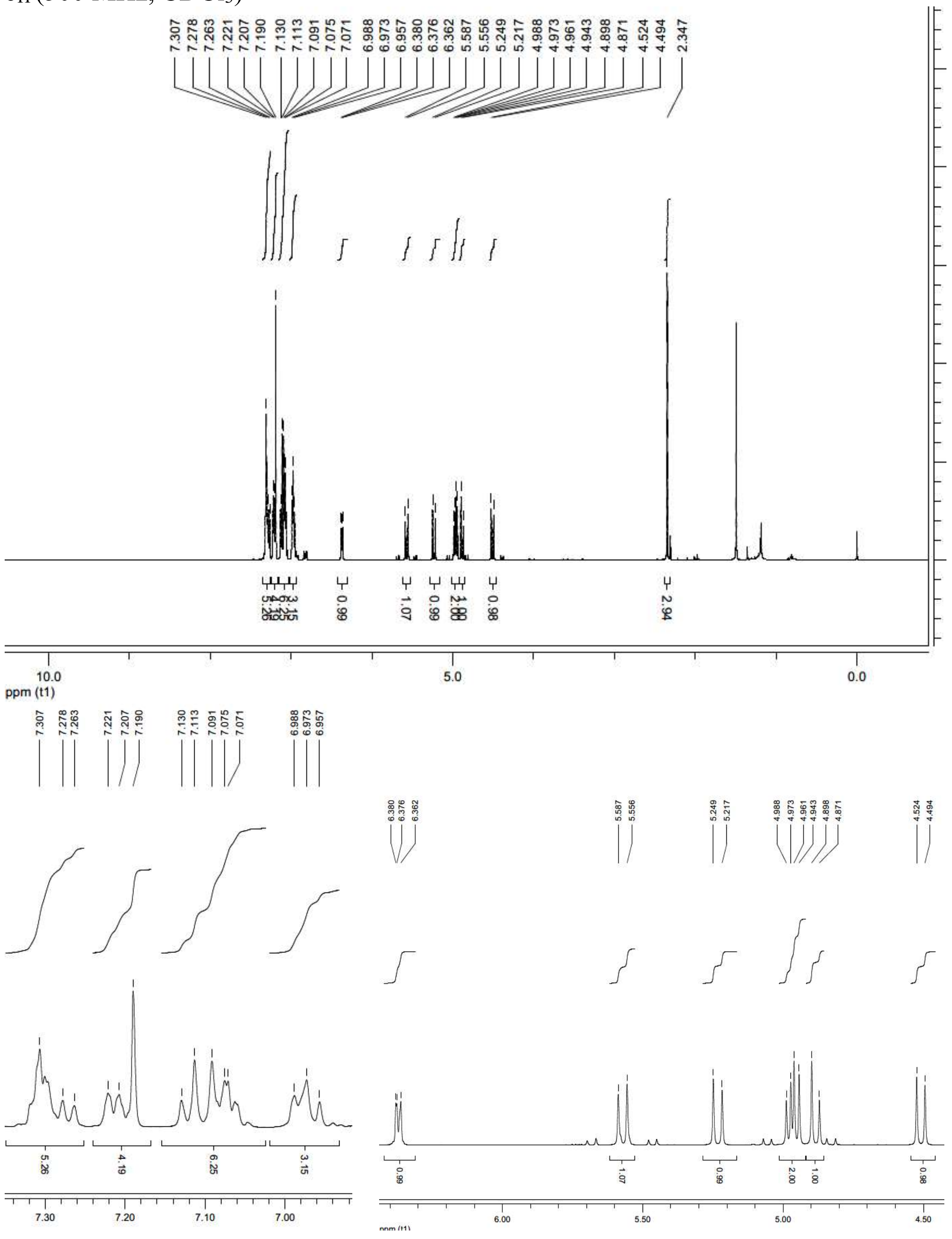

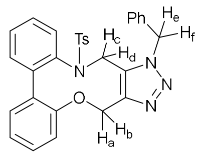

B

δ$_C$ (126 MHz, $CDCl_3$)

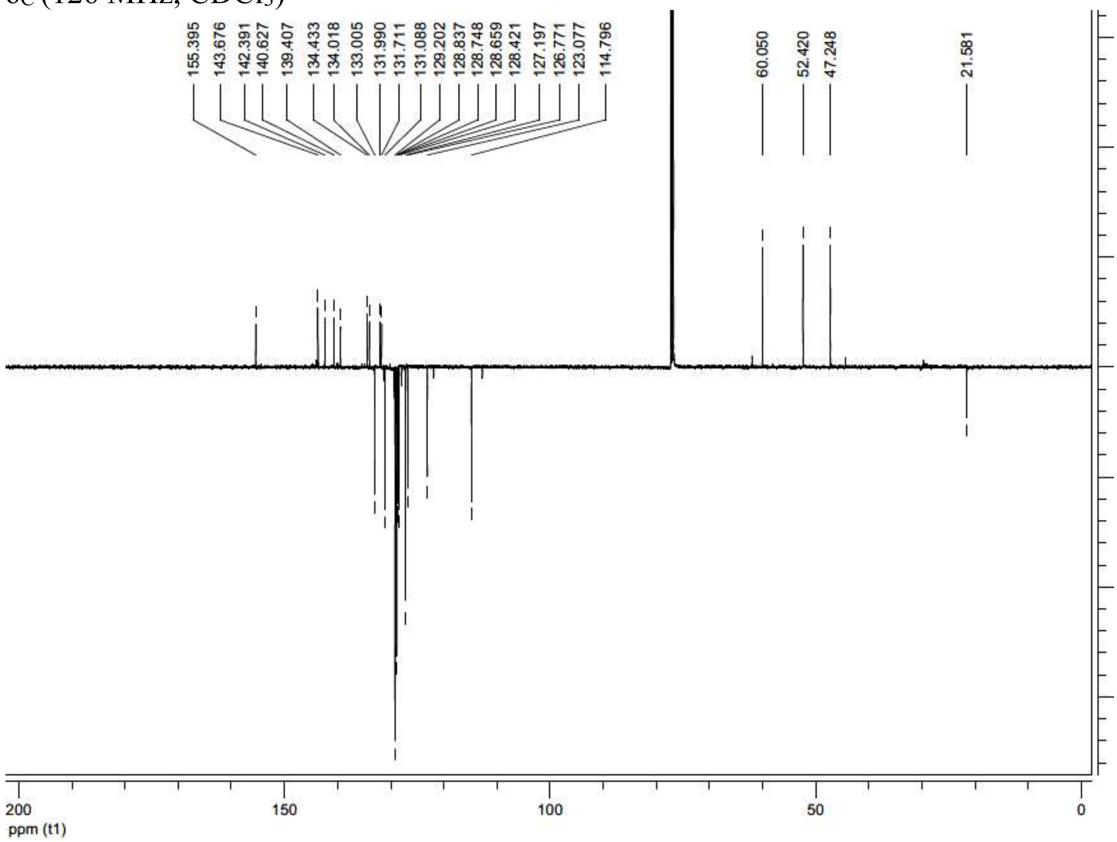

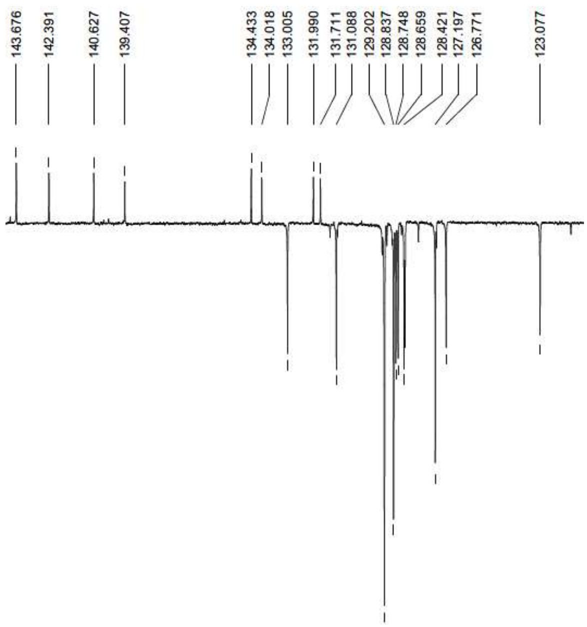

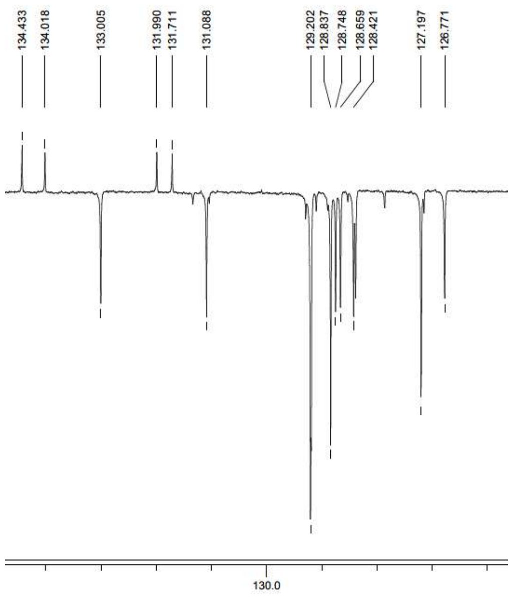

Compound 37.

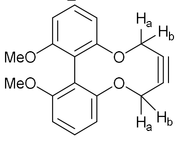

δH (500 MHz, CDCl3)

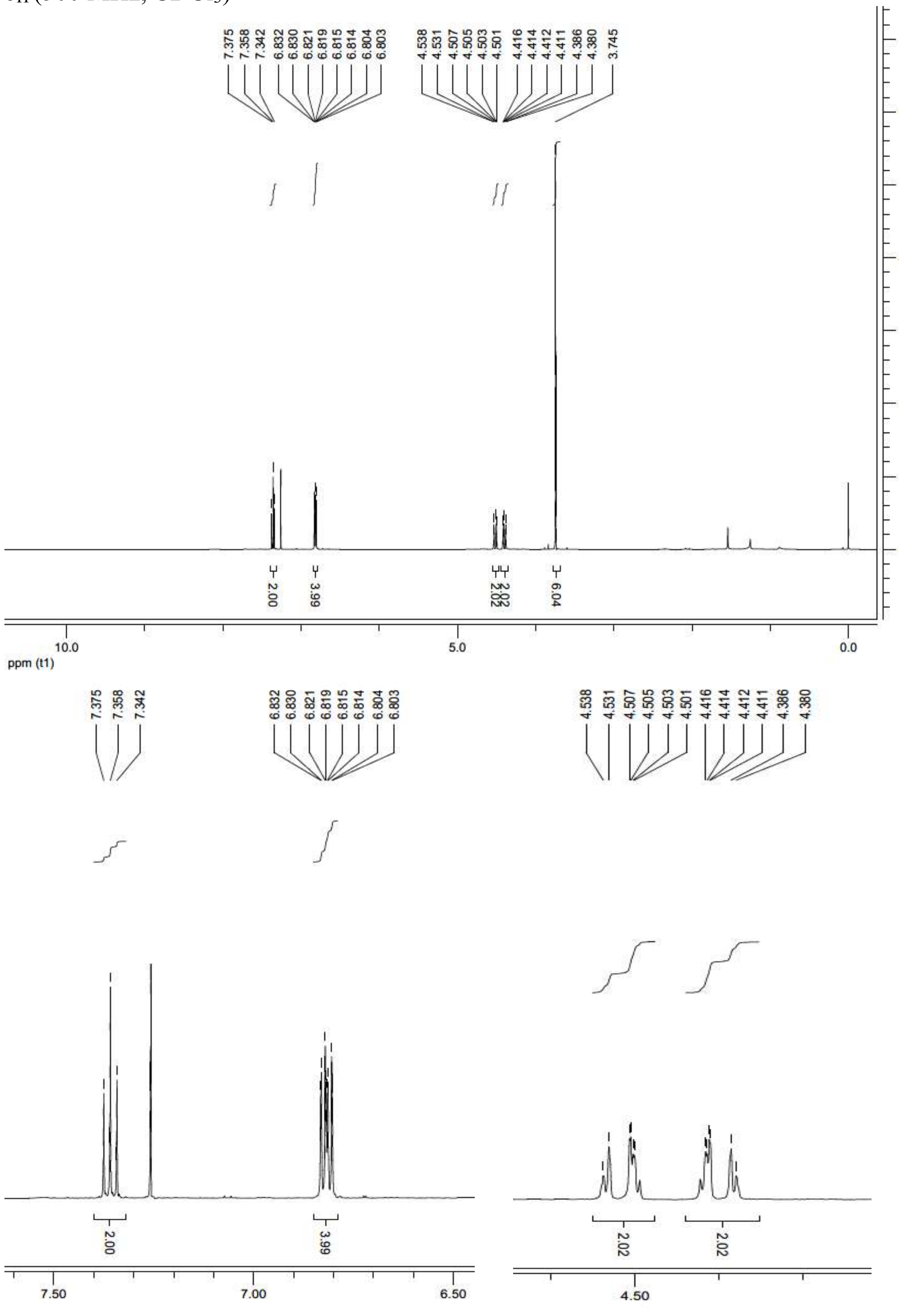

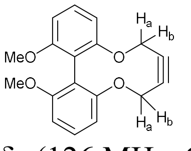

δ_C (126 MHz, $CDCl_3$)

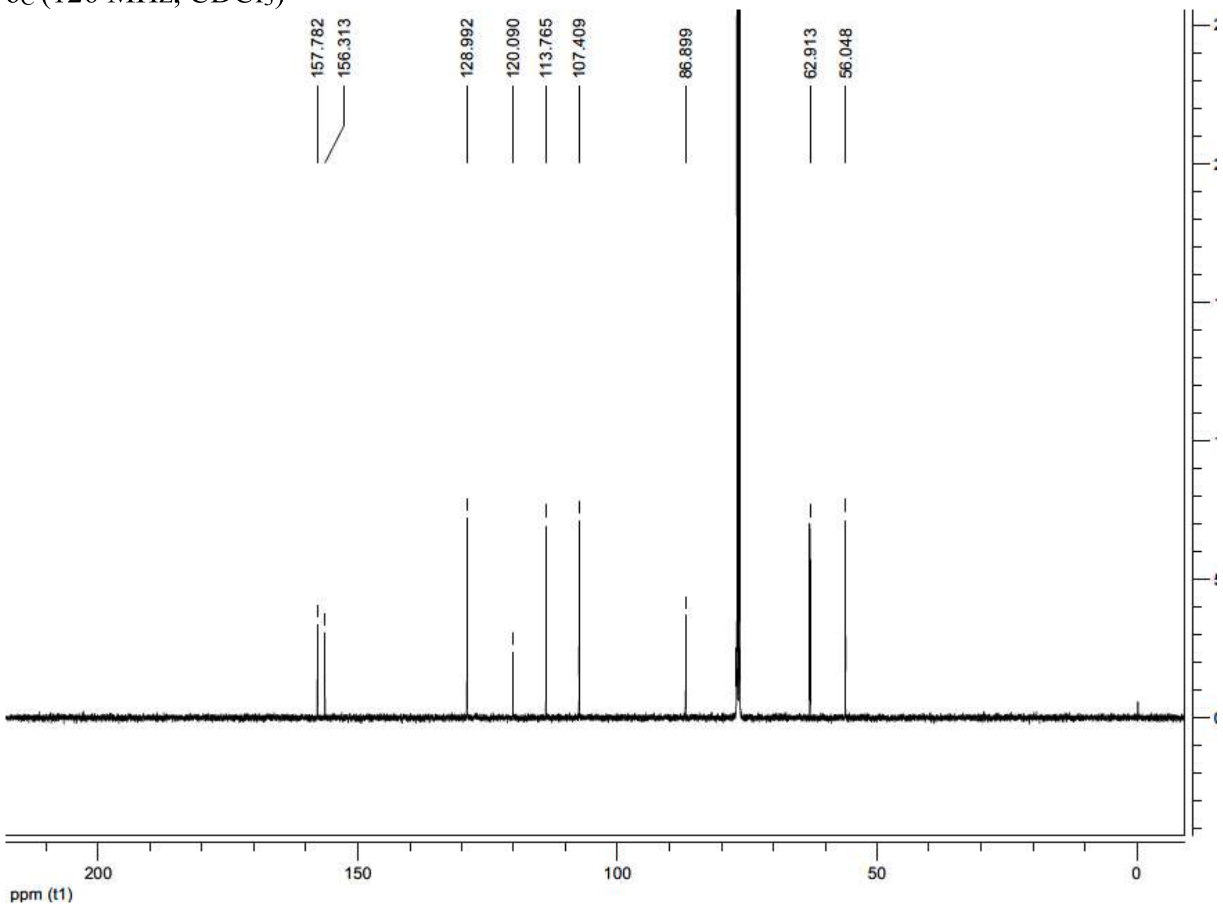

Compound 37 azide adduct 38.

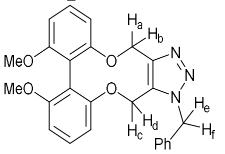

δ_H (500 MHz, $CDCl_3$)

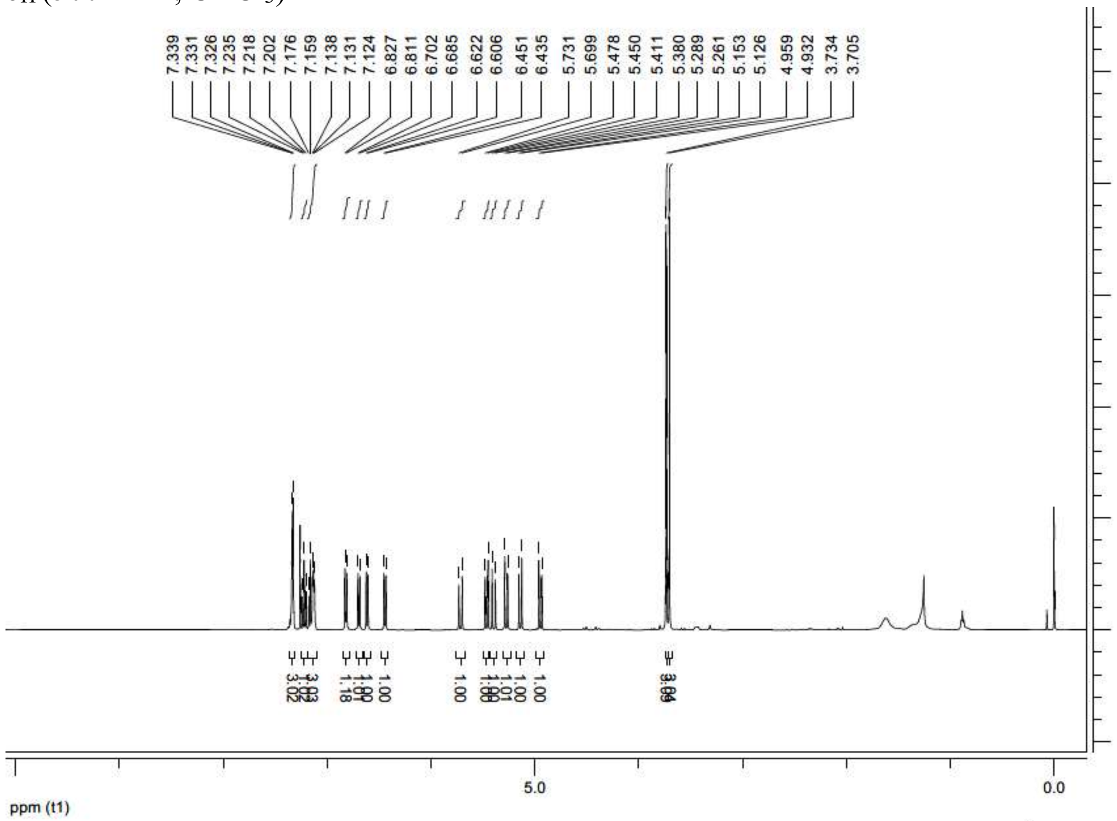

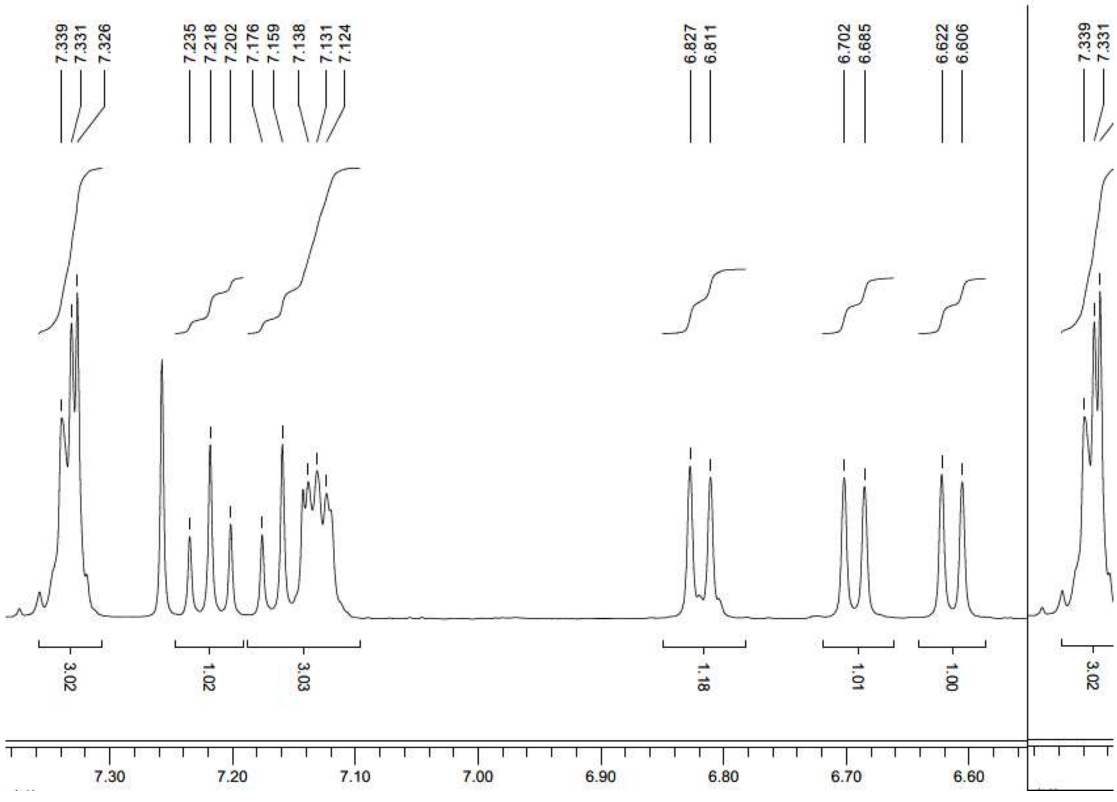

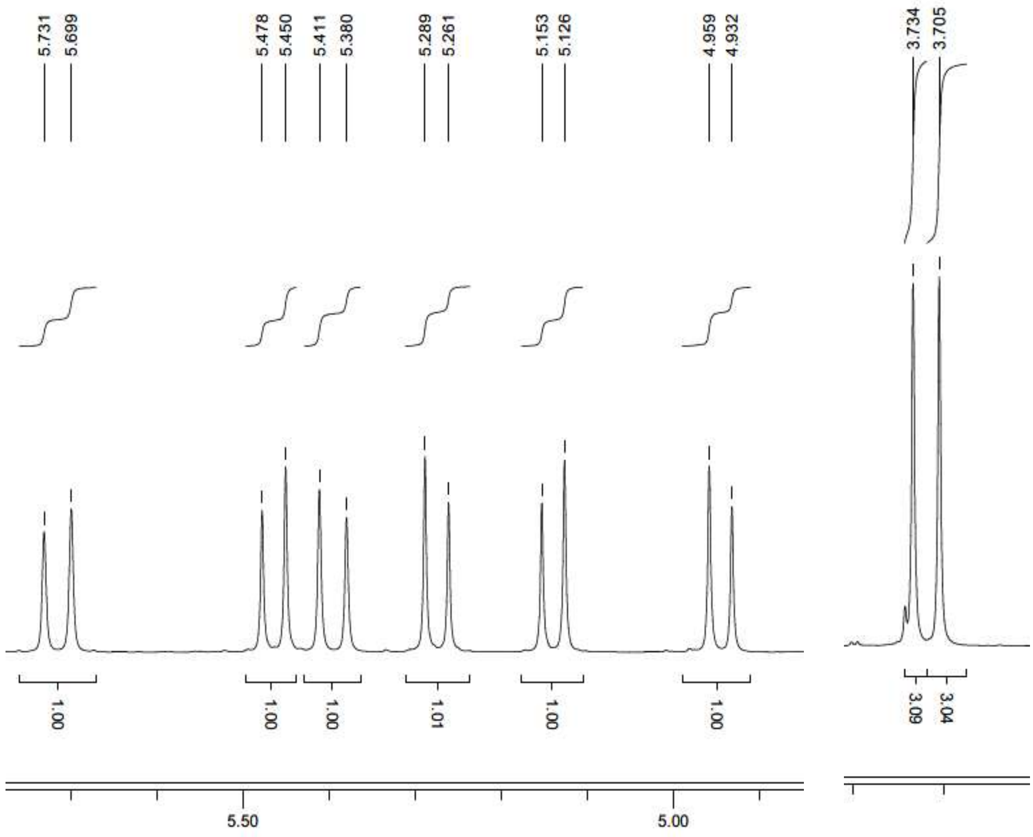
5.731
5.699
5.478
5.450
5.411
5.380
5.289
5.261
5.153
5.126
4.959
4.932
3.734
3.705
1.00
1.00
1.00
1.01
1.00
1.00
3.09
3.04
5.50
5.00

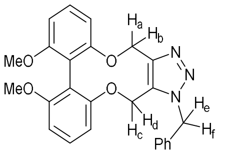

δ$_C$ (126 MHz, $CDCl_3$)

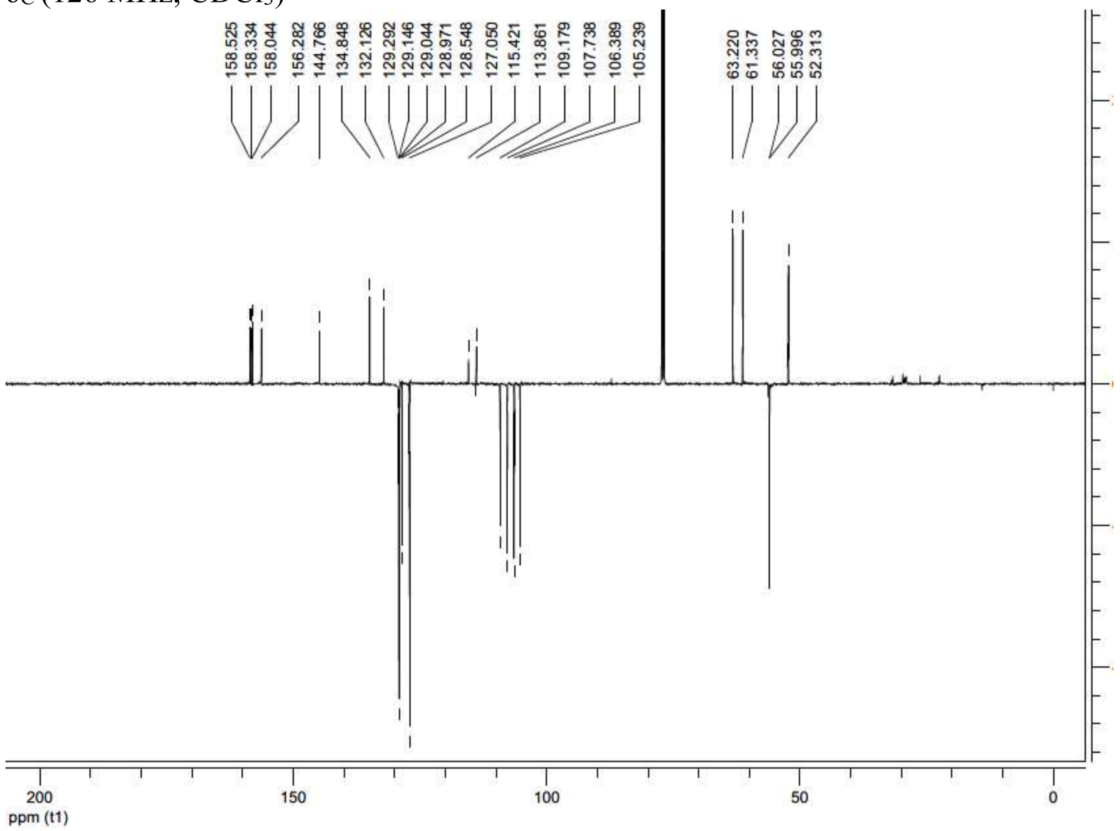

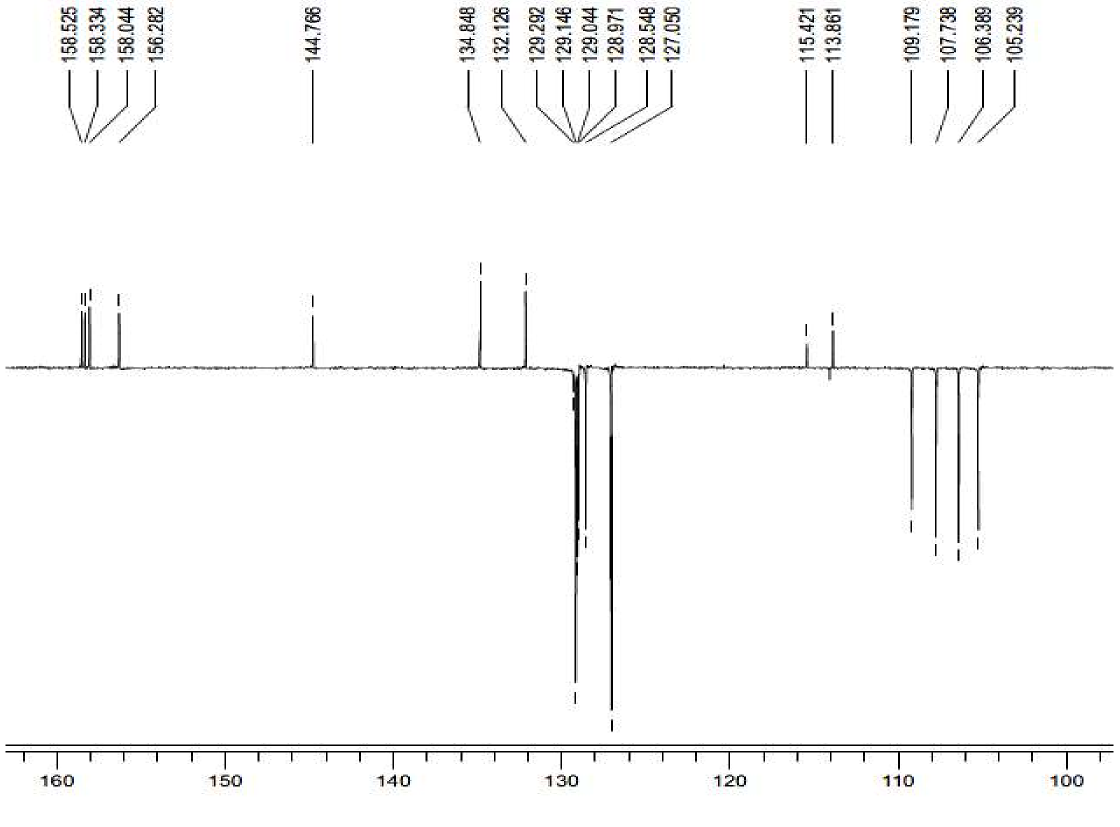

Compound 42.

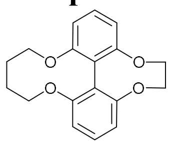

δ_H (500 MHz, $CDCl_3$)

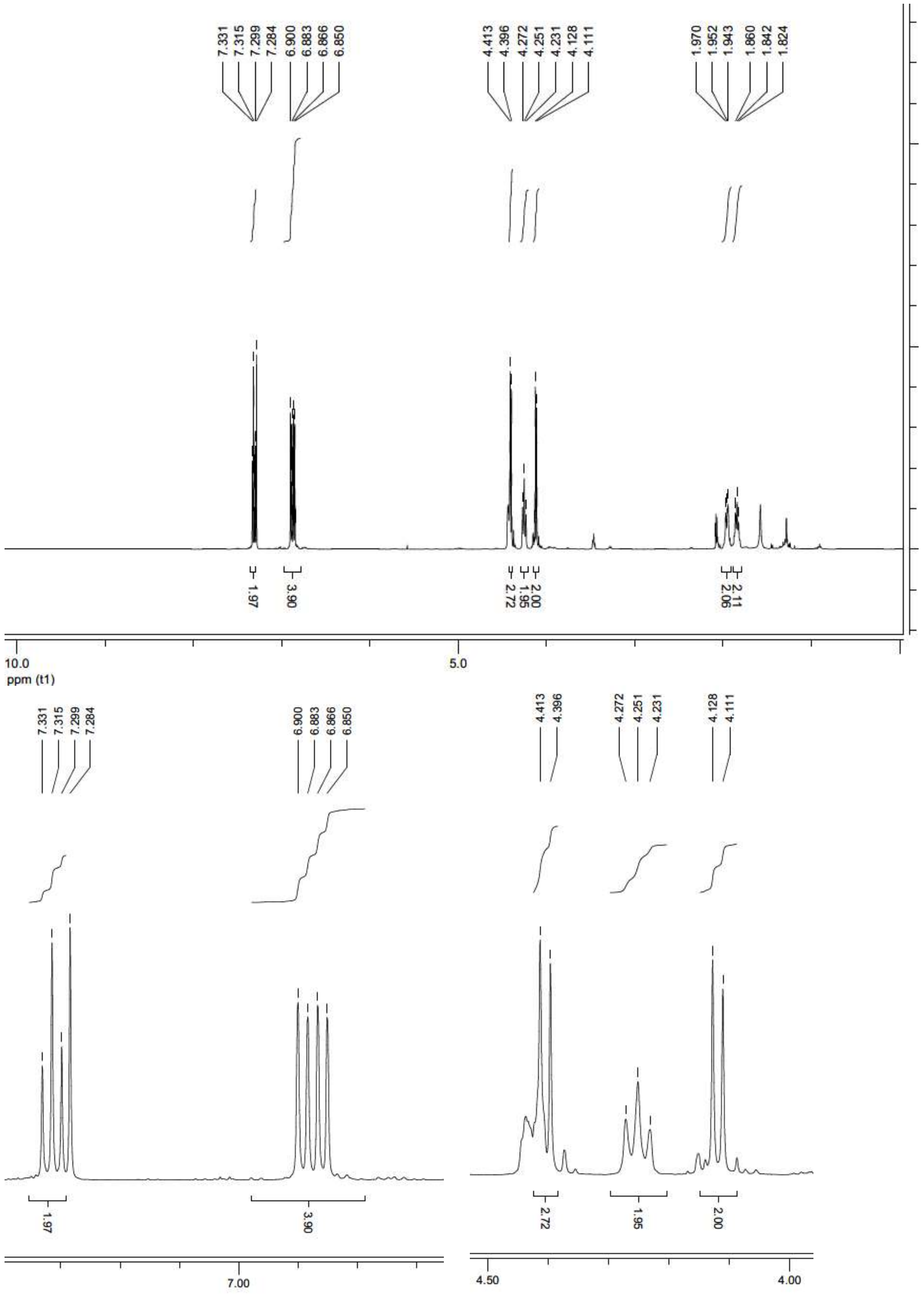

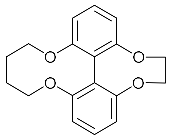

δ$_C$ (126 MHz, $CDCl_3$)

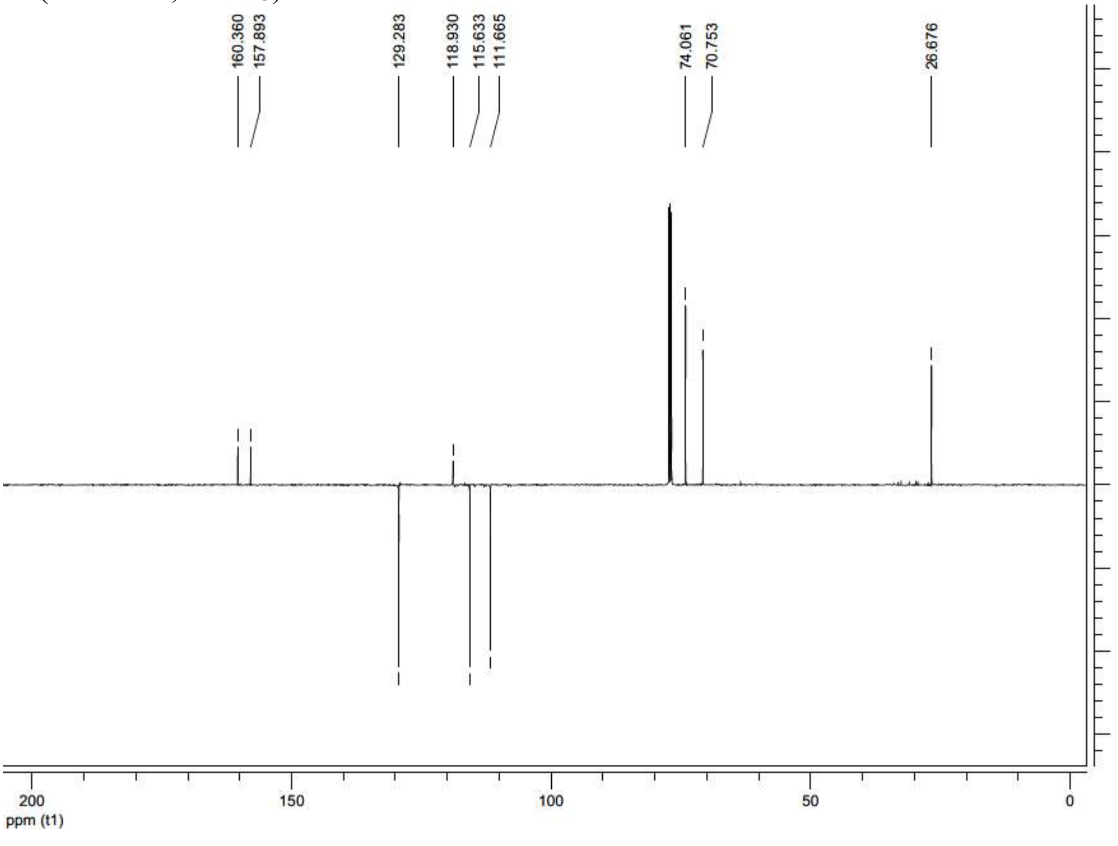

Compound 44.

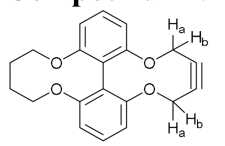

δH (500 MHz, CDCl3)

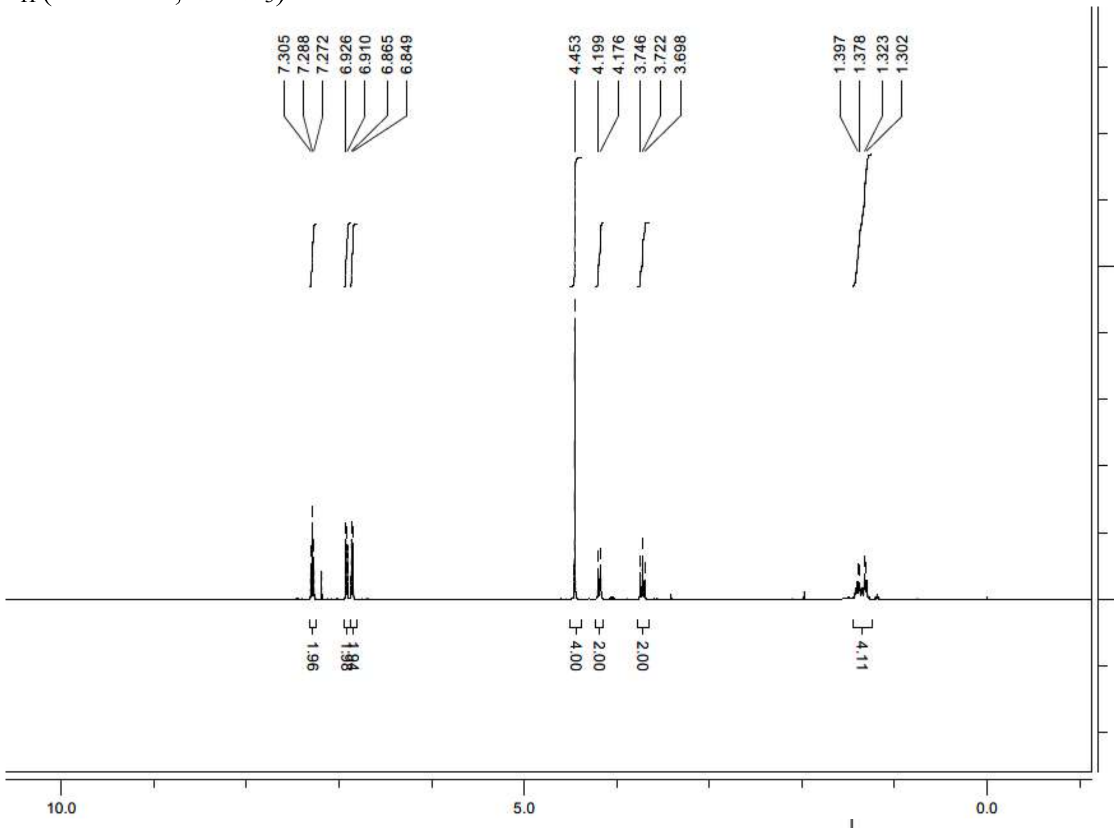

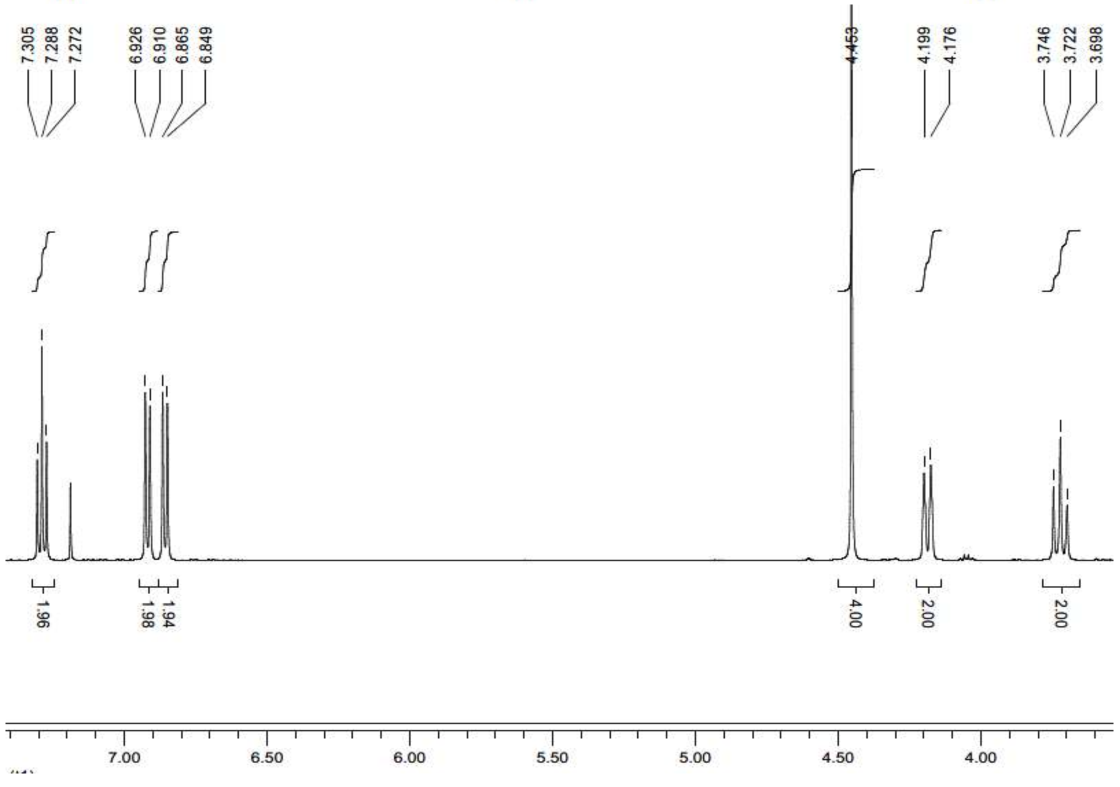

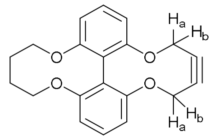

δC (126 MHz, $CDCl_3$)

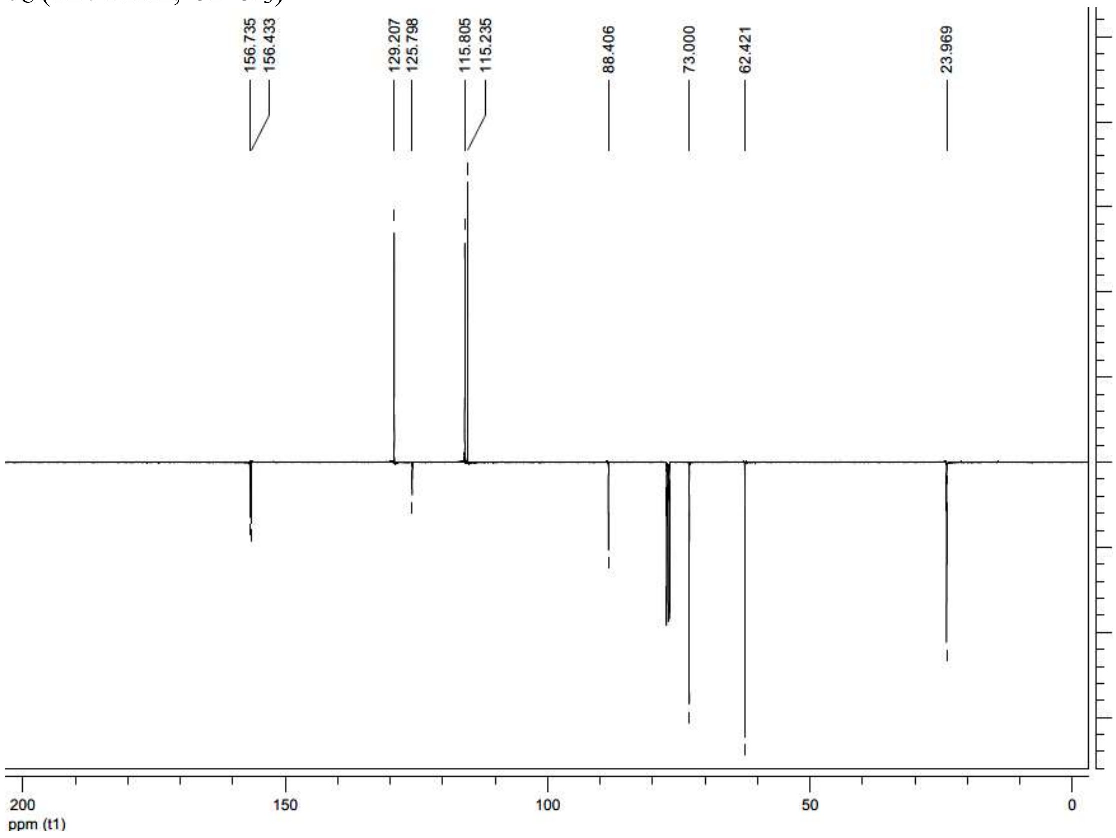

Compound 45.

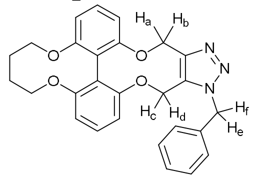

δ_H (500 MHz, $CDCl_3$)

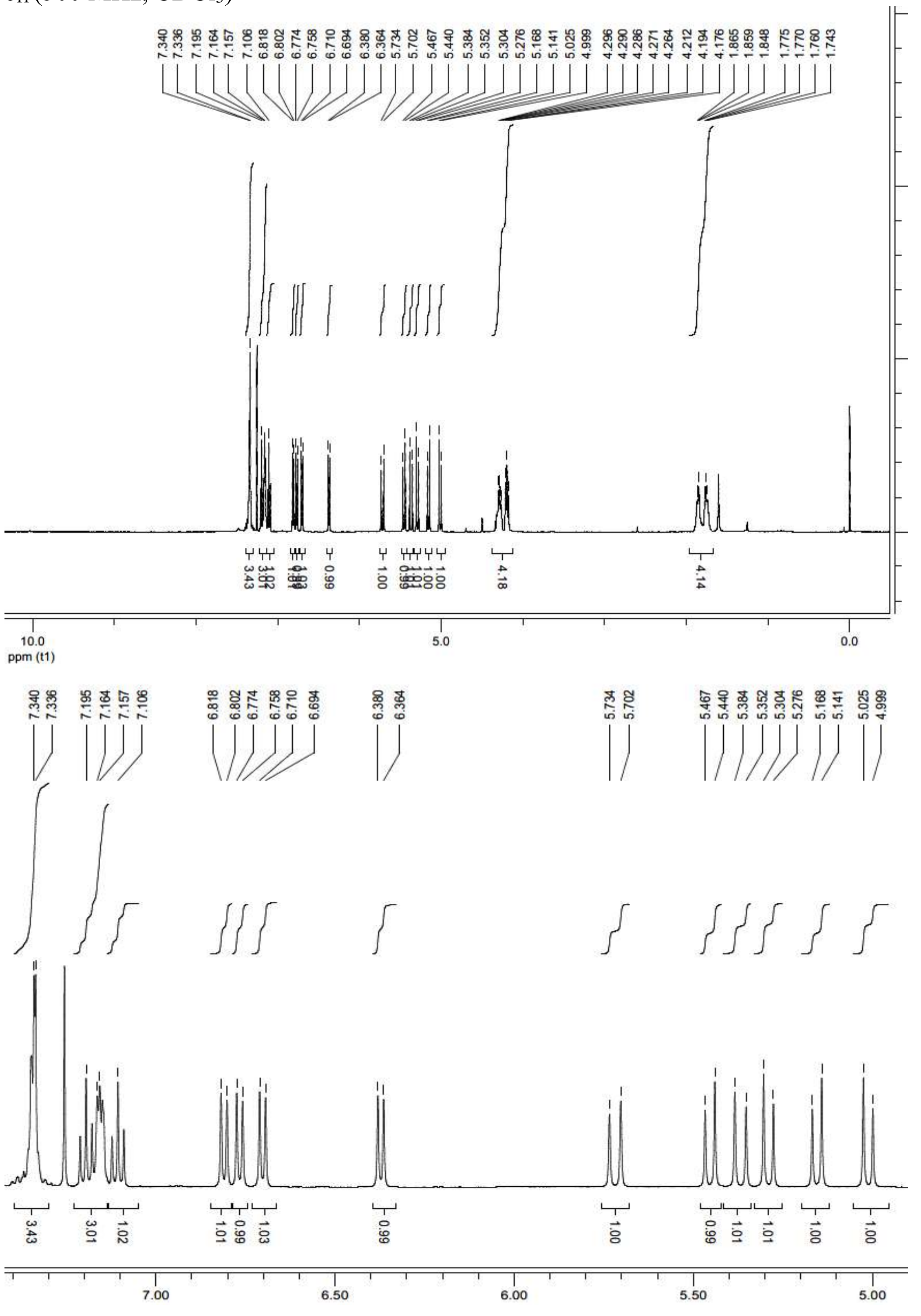

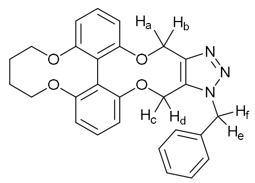

δ_C (126 MHz, $CDCl_3$)

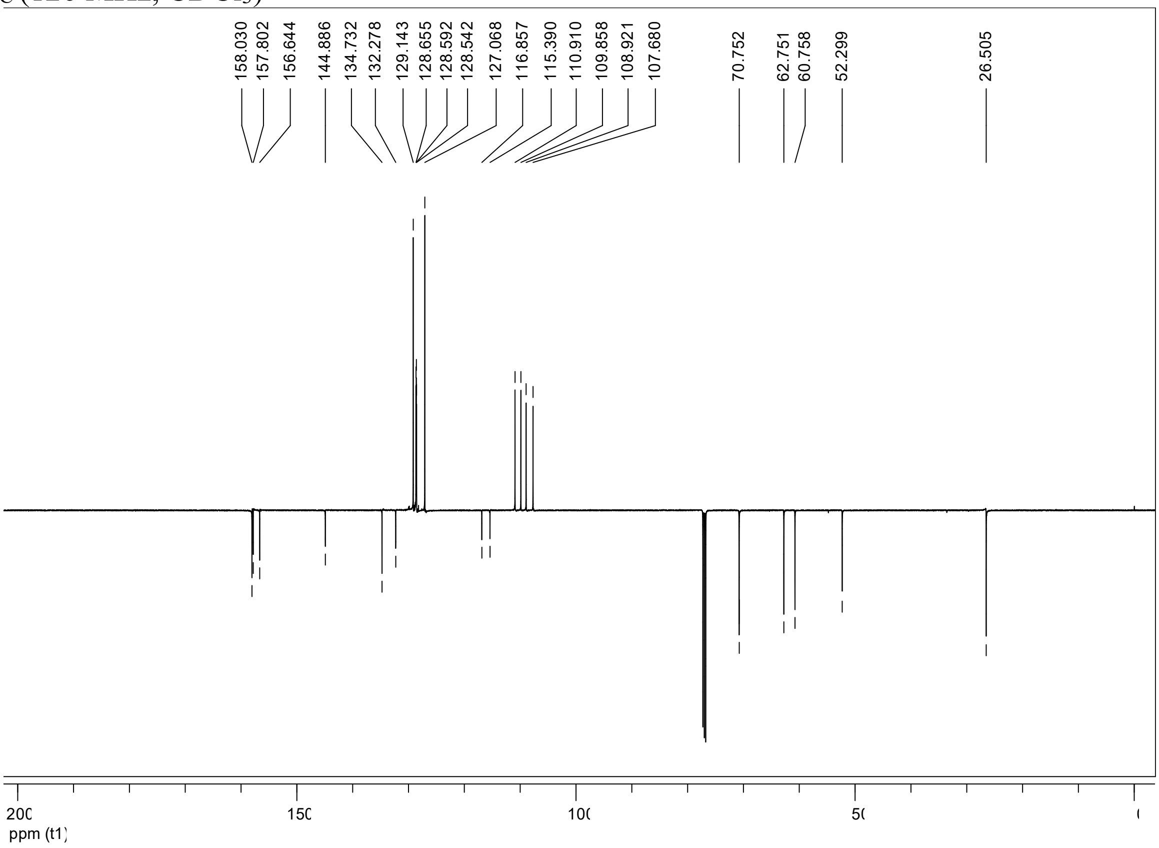

Compound 47.

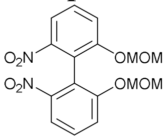

δ_H (500 MHz, $CDCl_3$)

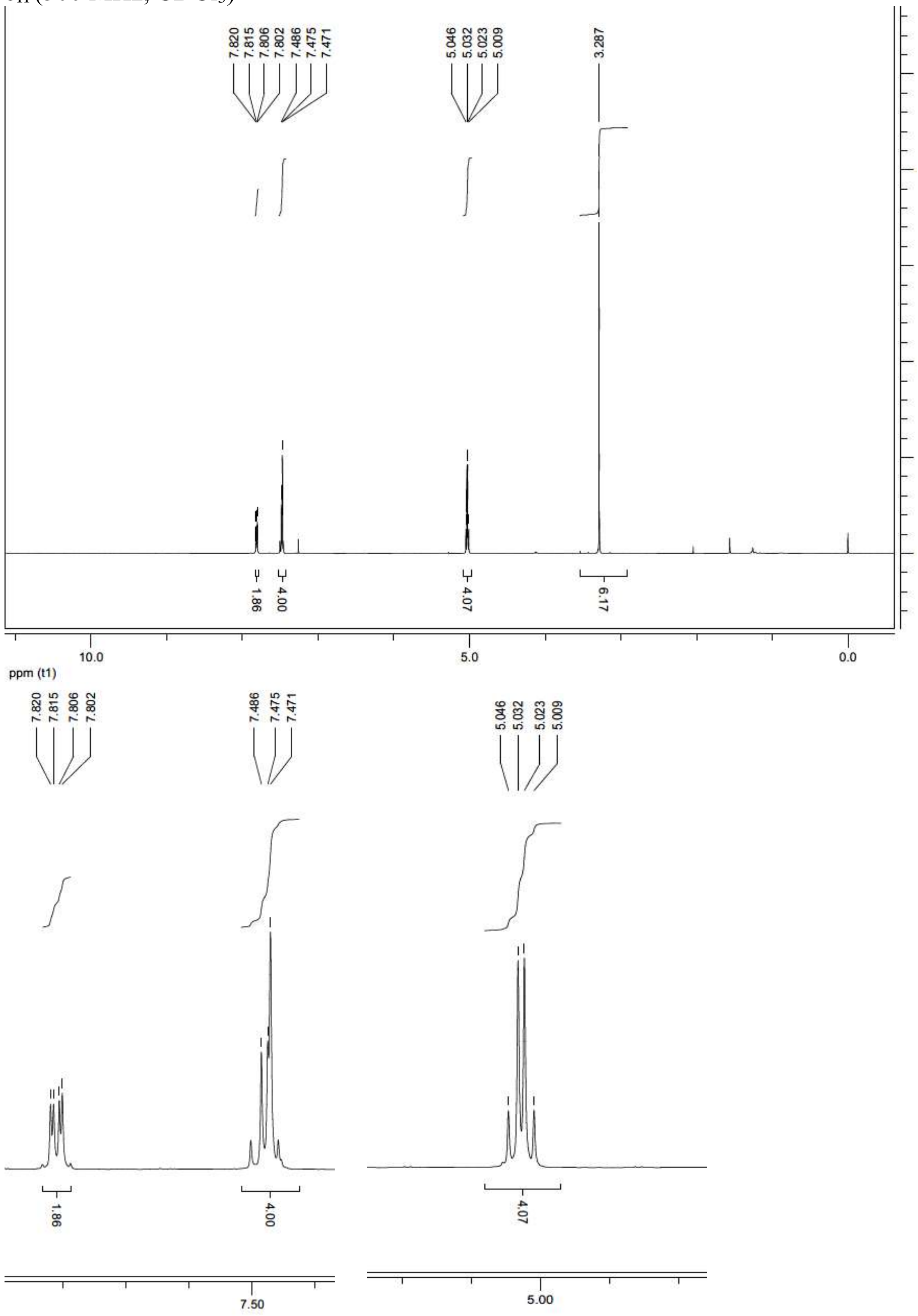

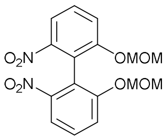

δ_C (126 MHz, $CDCl_3$)

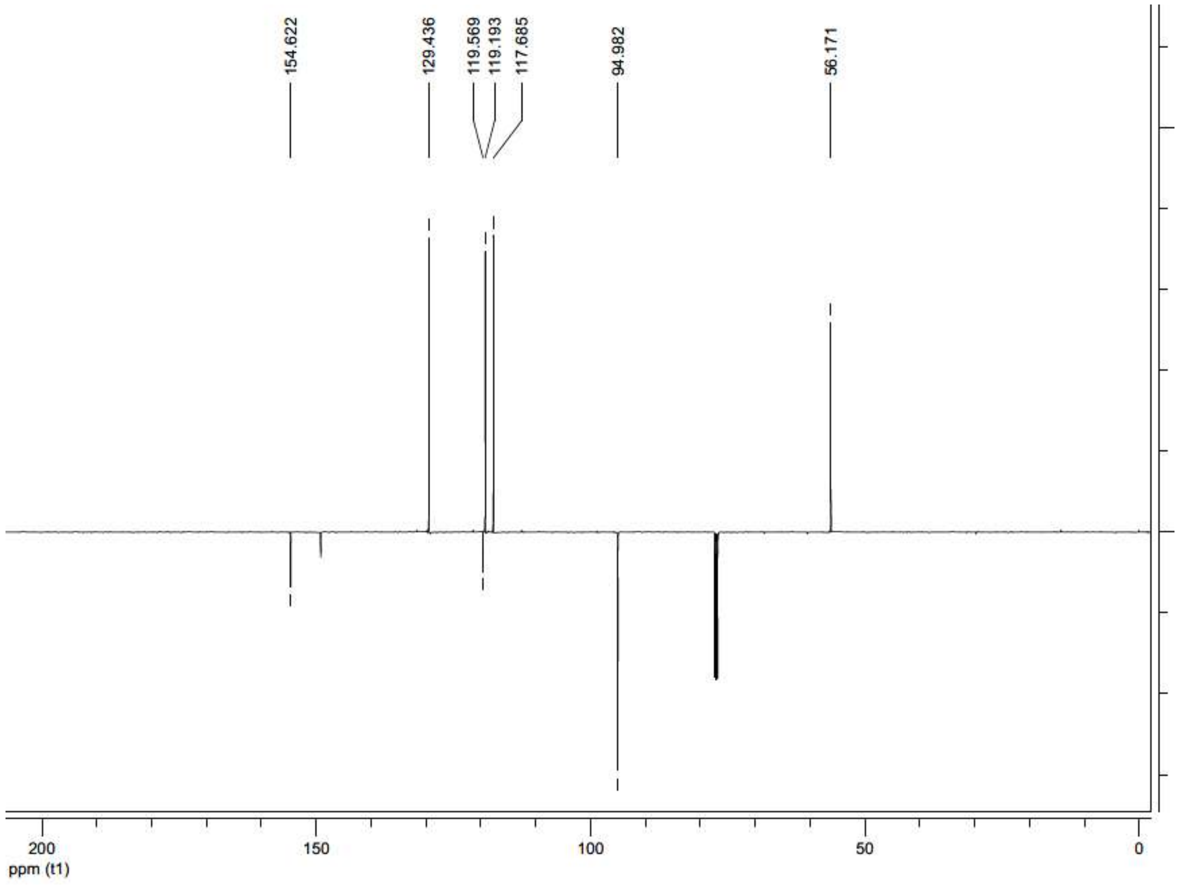

Compound 48.

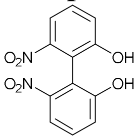

δ_H (500 MHz, CD_3CN)

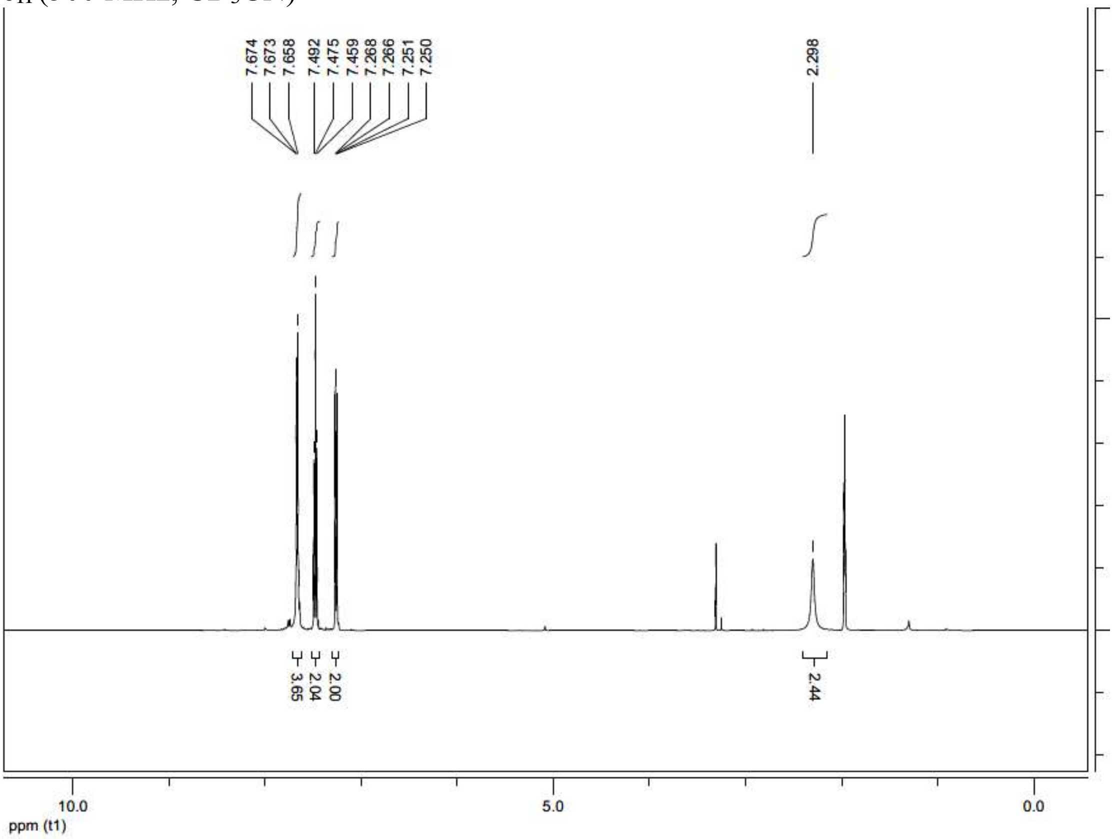

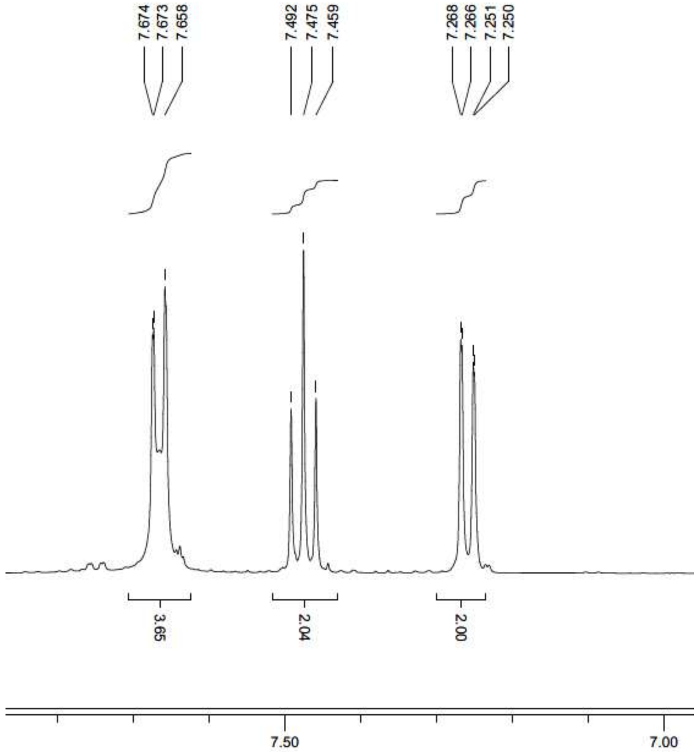

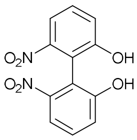

δ_C (126 MHz, CD_3CN)

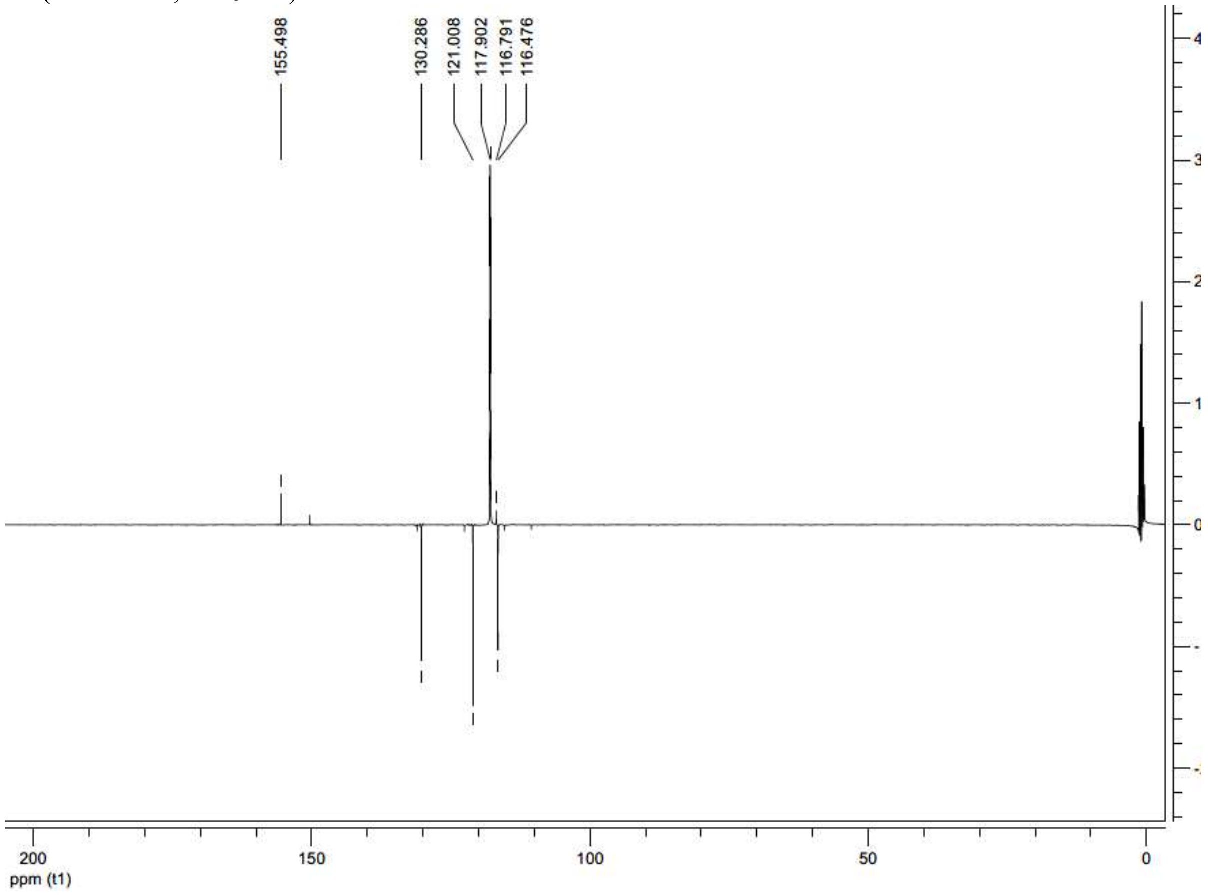

Compound 49.

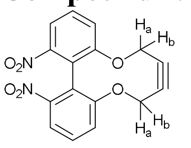

δ$_H$ (500 MHz, $CDCl_3$)

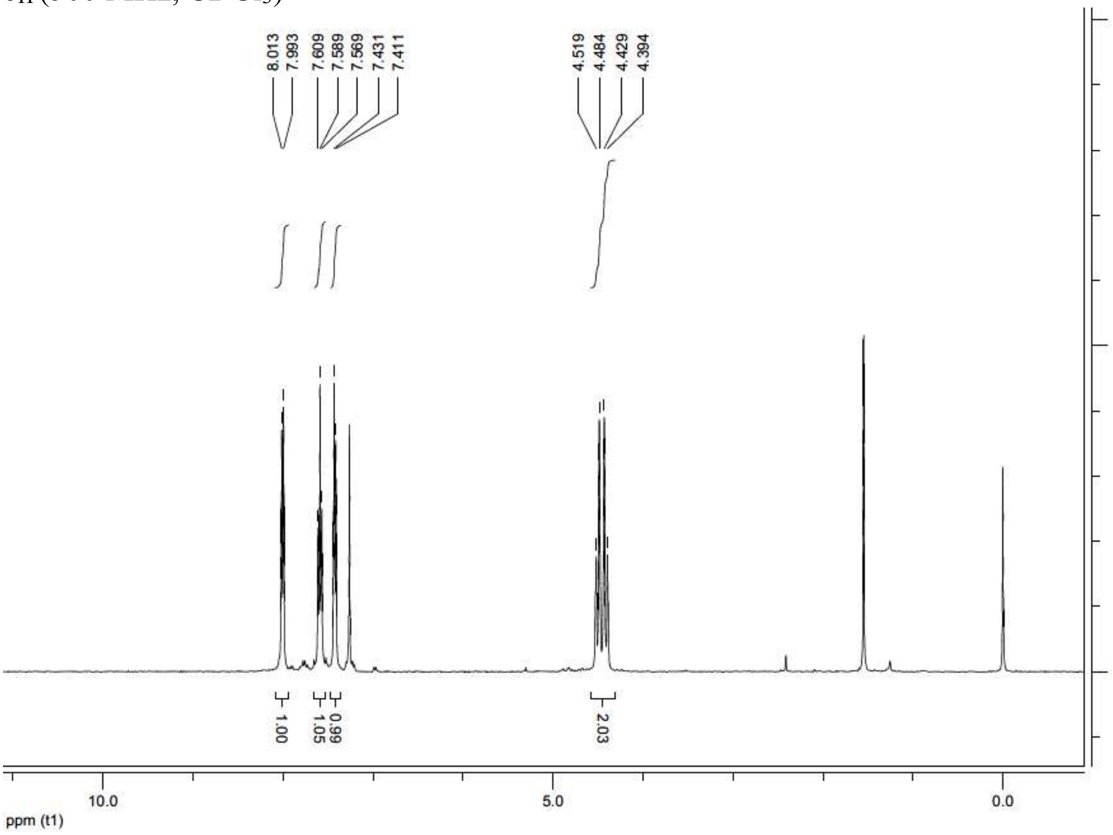

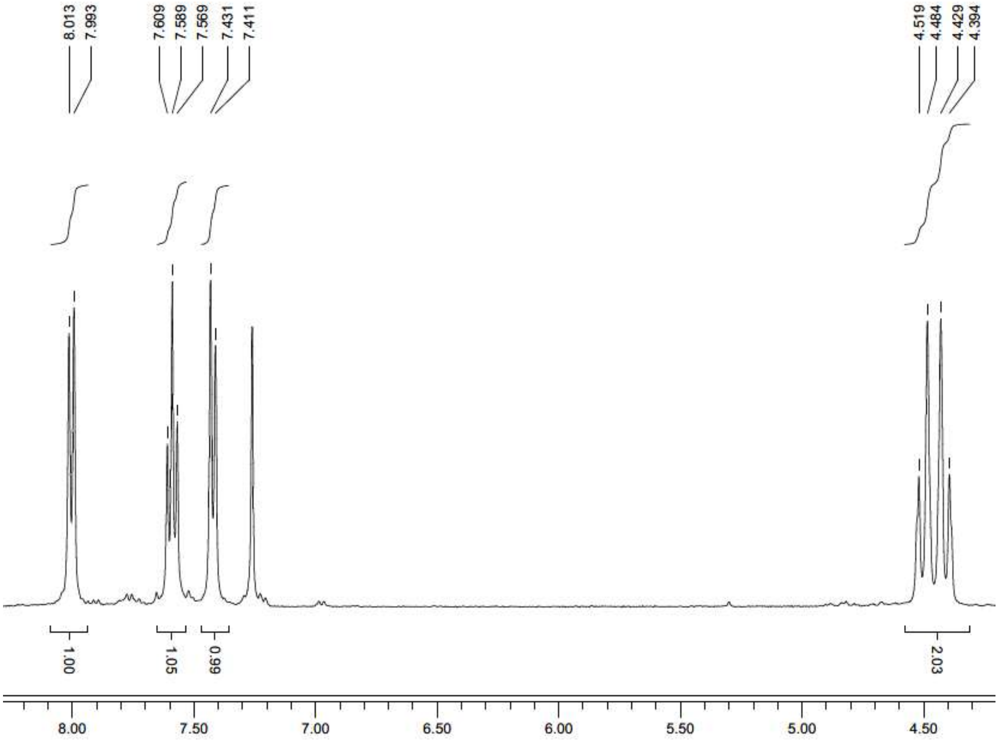

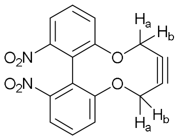

δ$_C$ (126 MHz, $CDCl_3$)

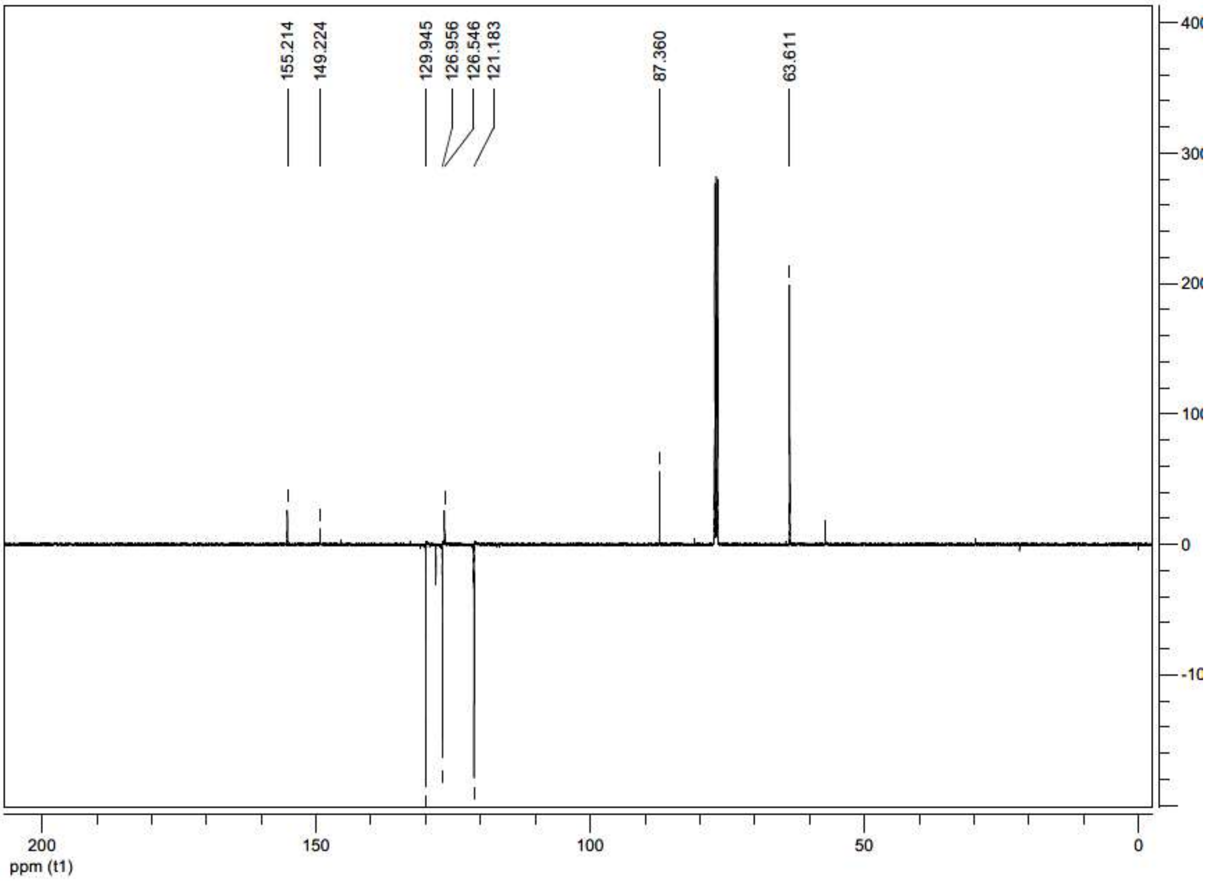

Benzyl azide adduct of compound 49.

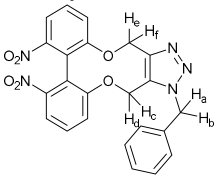

δ_H (500 MHz, $CDCl_3$)

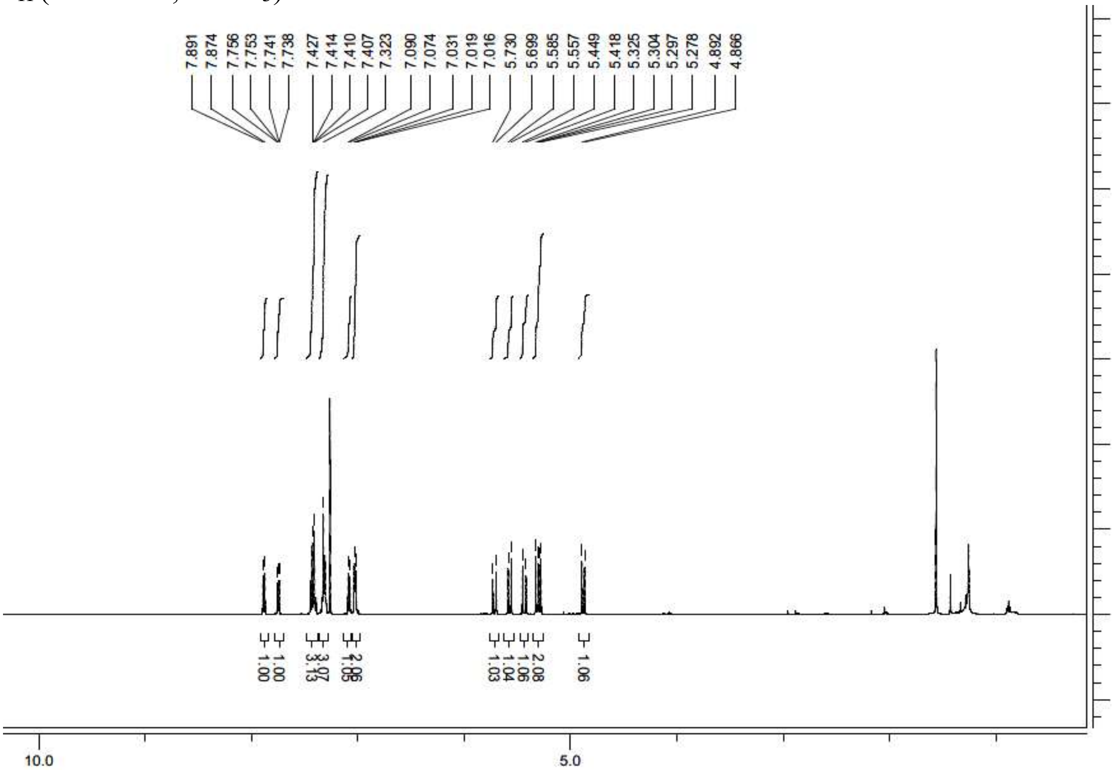

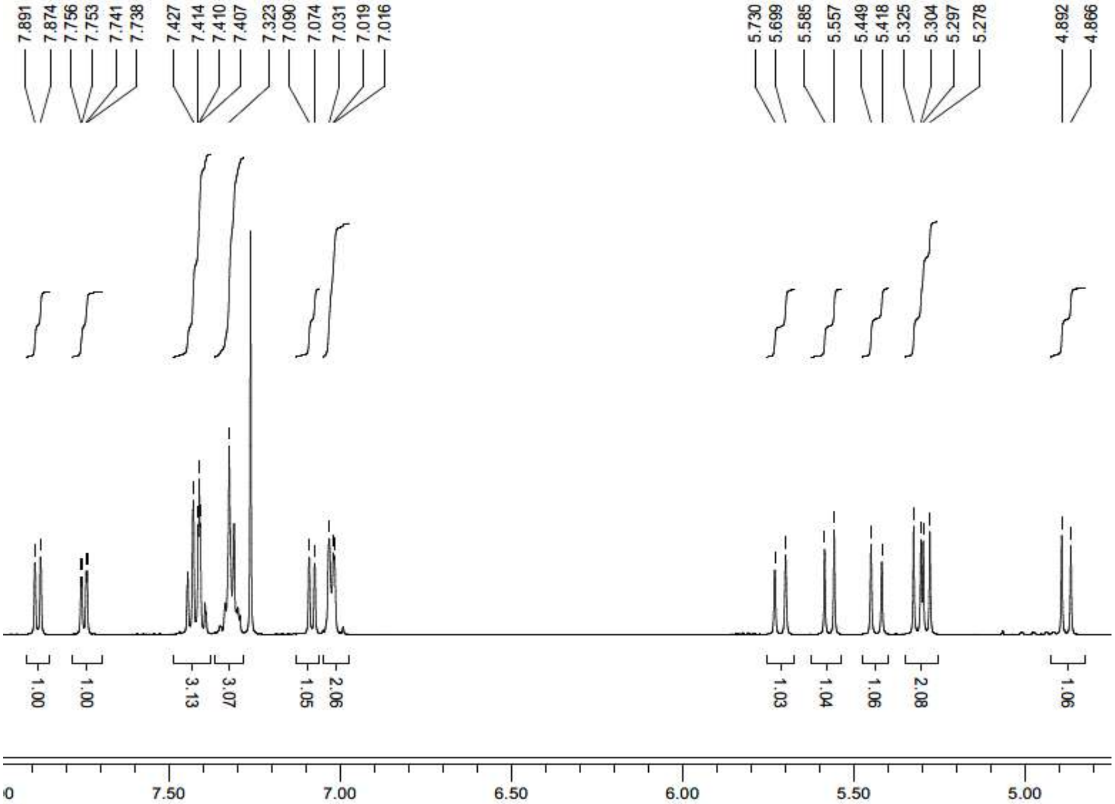

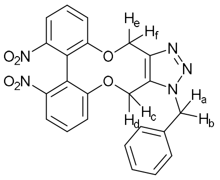

δC (126 MHz, $CDCl_3$)

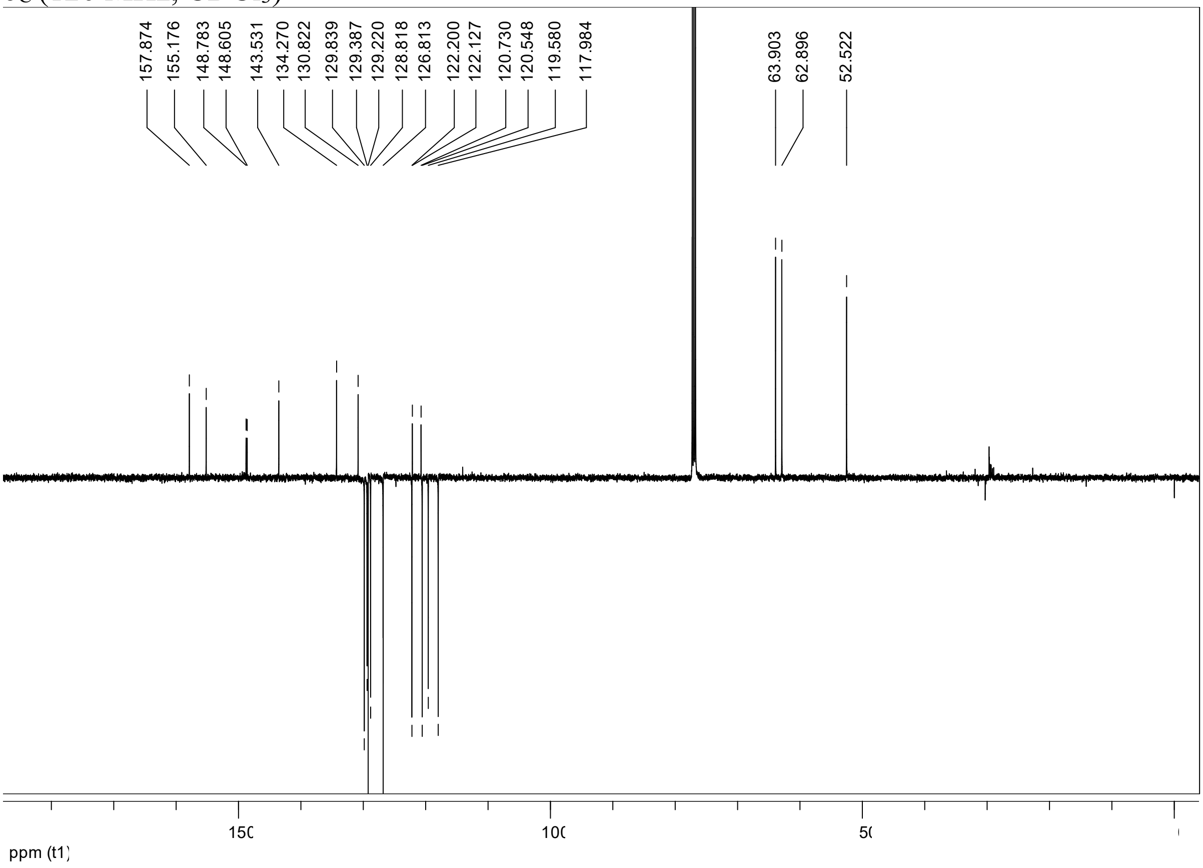

Compound 50.

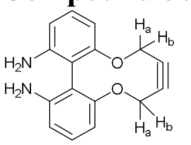

δ_H (500 MHz, $CDCl_3$)

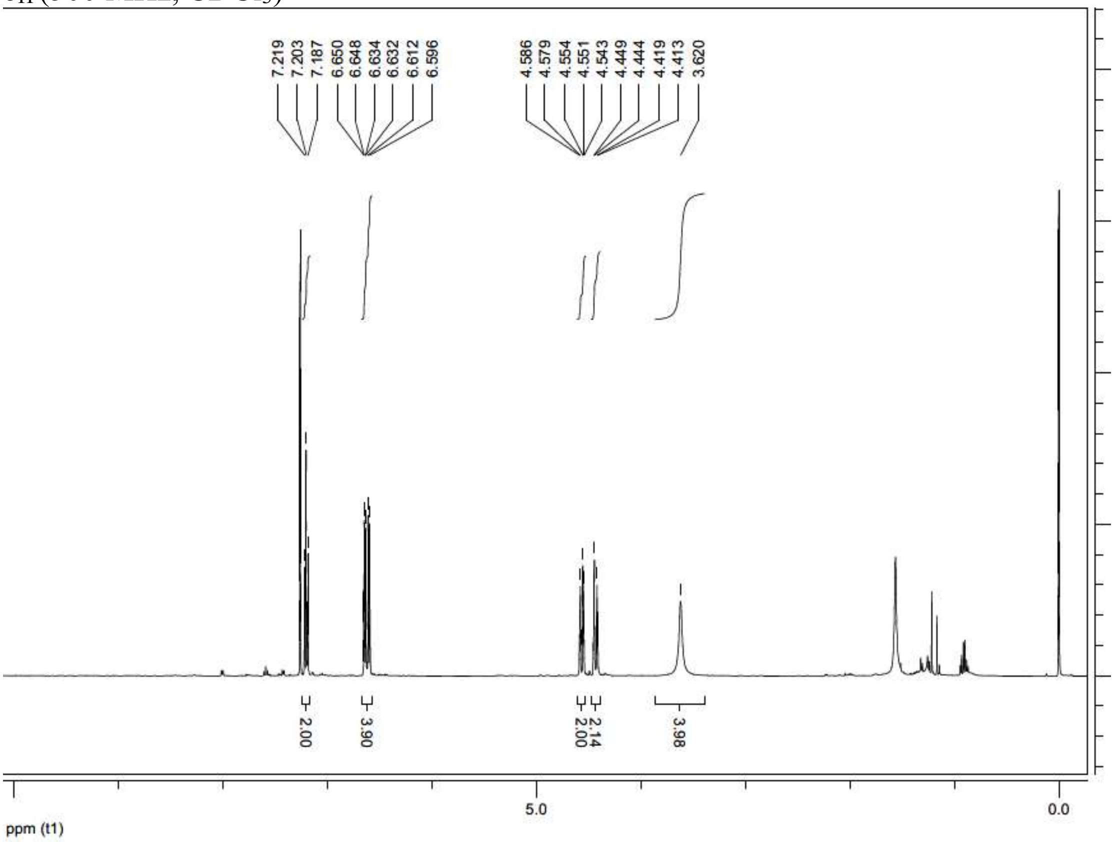

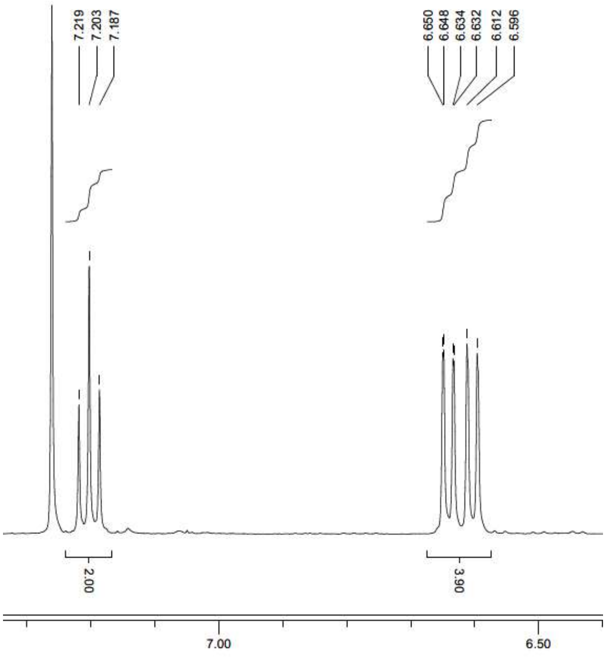

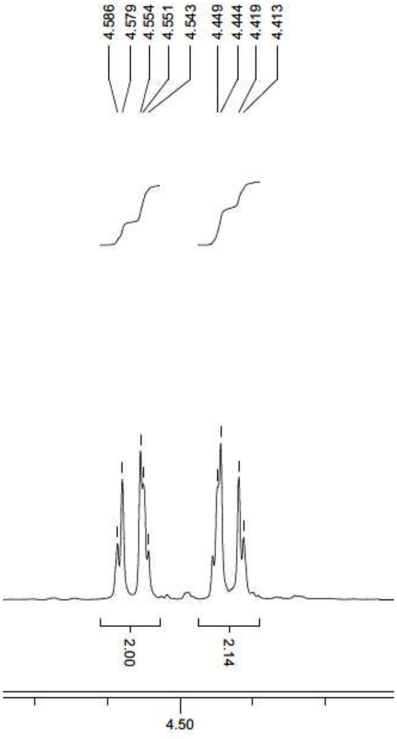

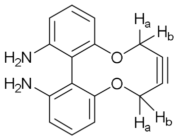

δ_C (126 MHz, $CDCl_3$)

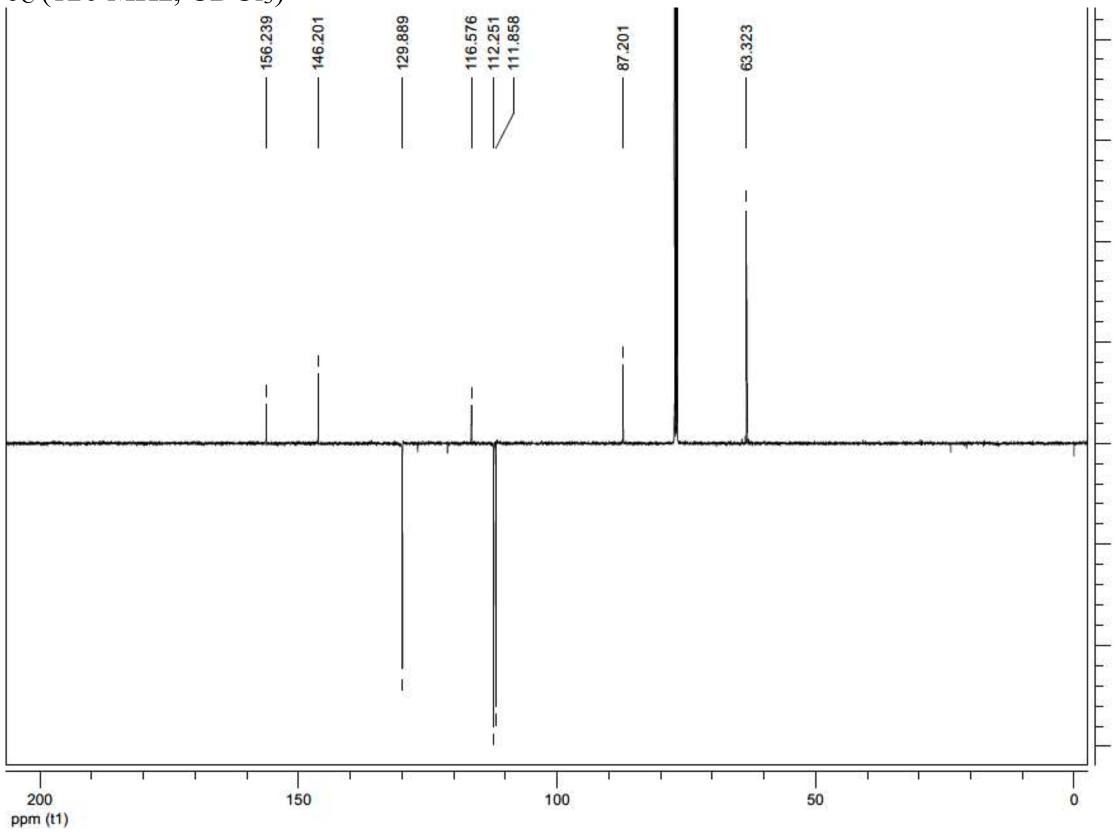

Benzyl azide adduct of compound 50.

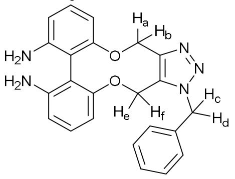

δ_H (500 MHz, $CDCl_3$)

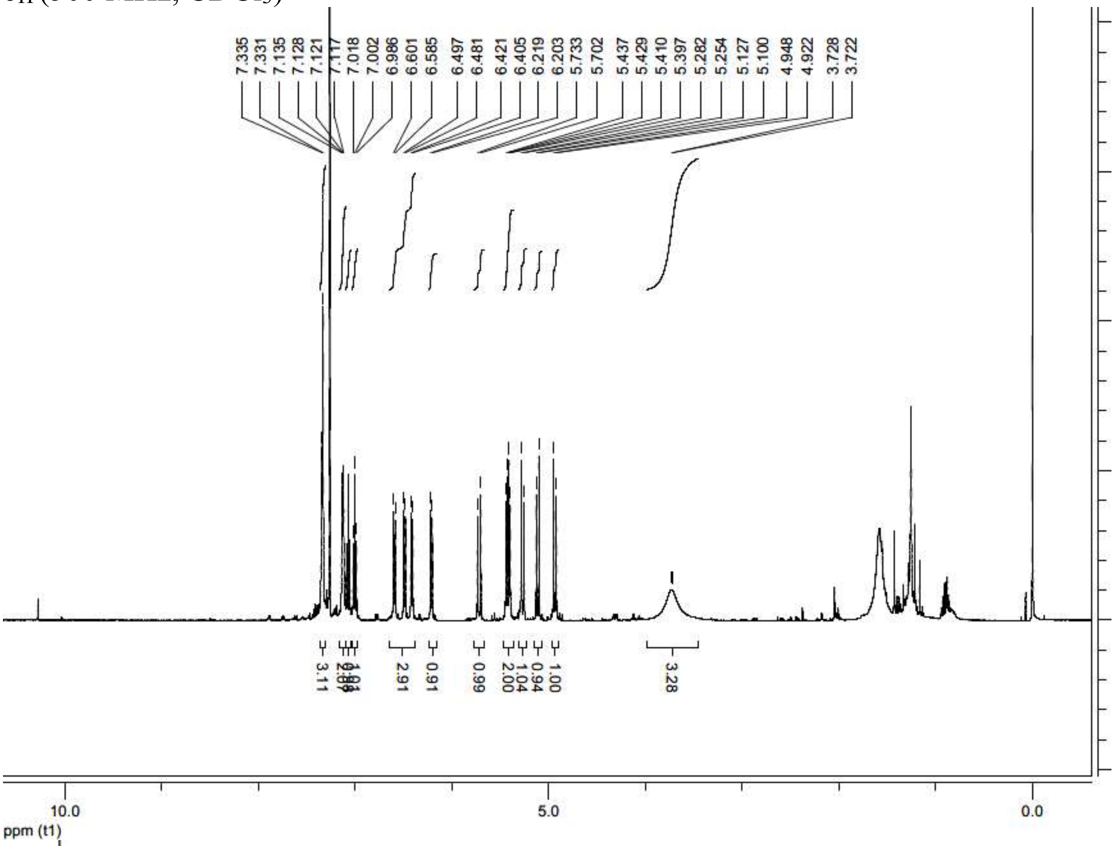

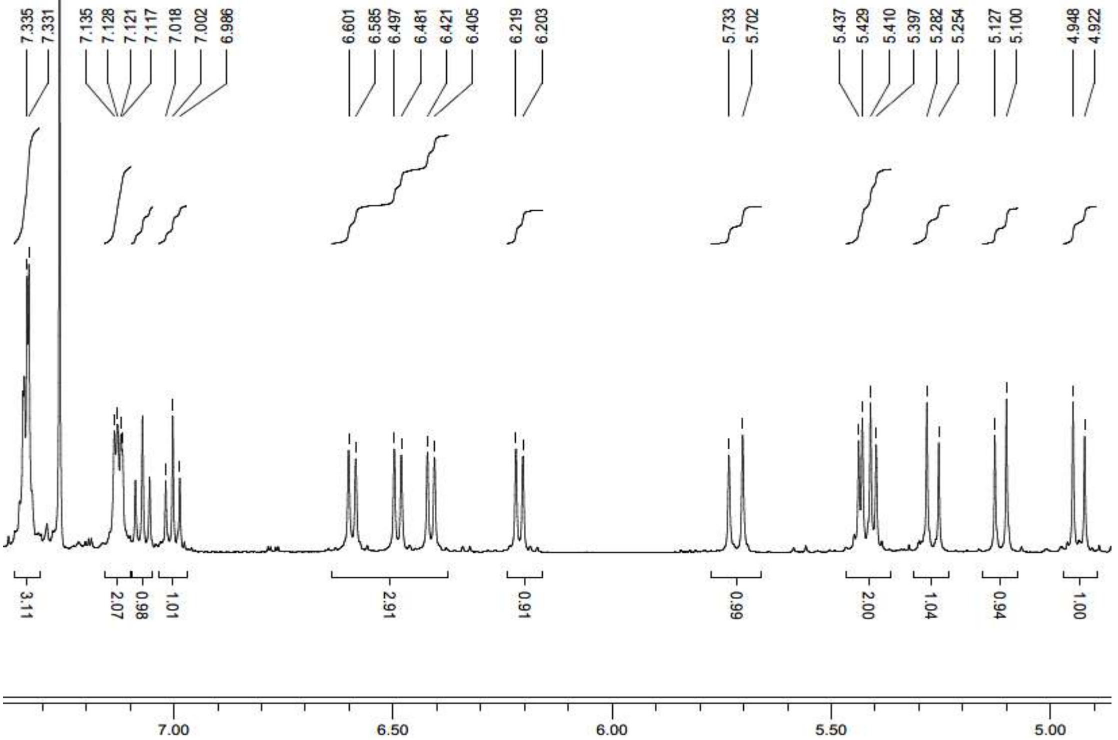

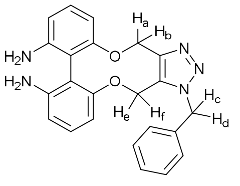

δC (126 MHz, CDCl3)

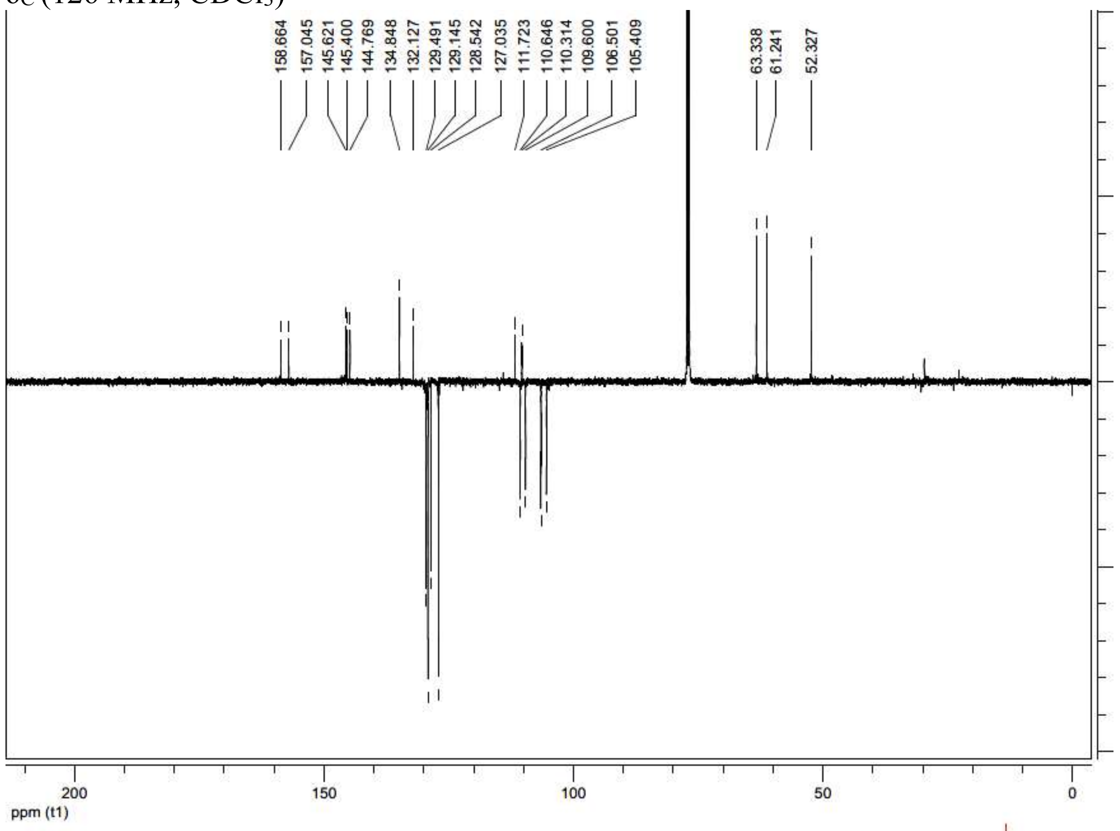

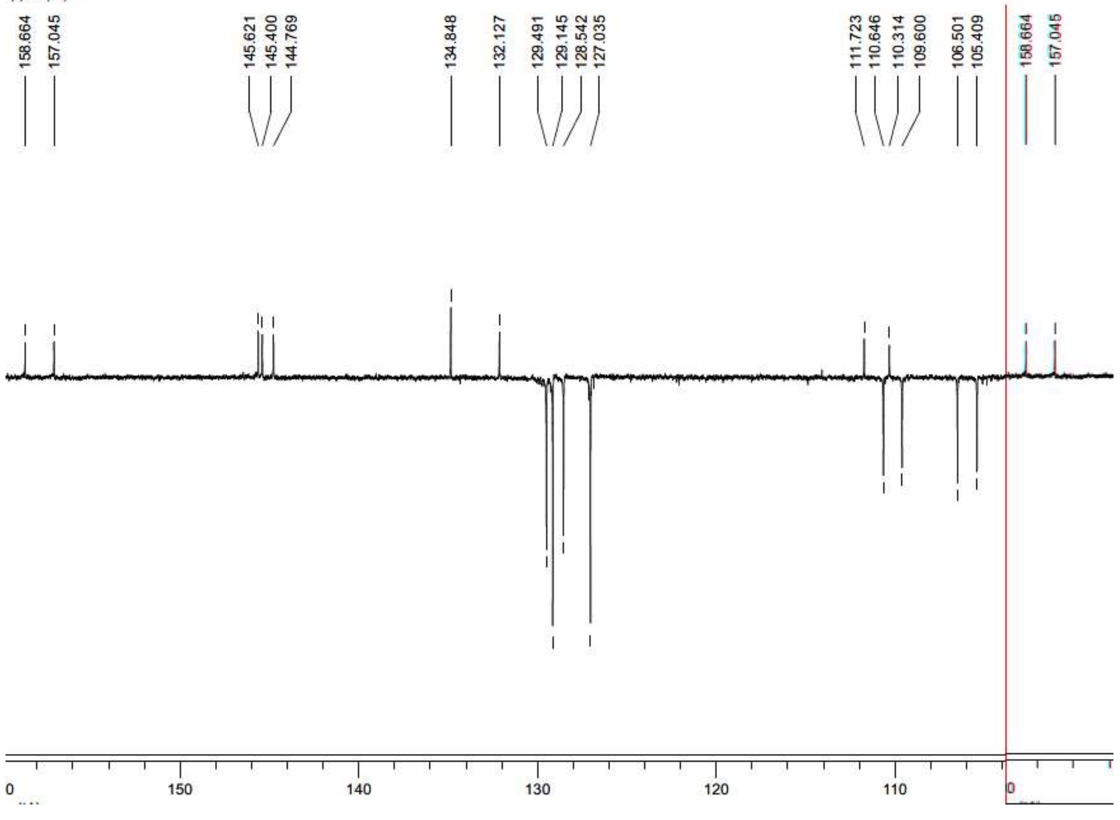

Compound 51.

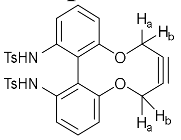

δ_H (500 MHz, $CDCl_3$)

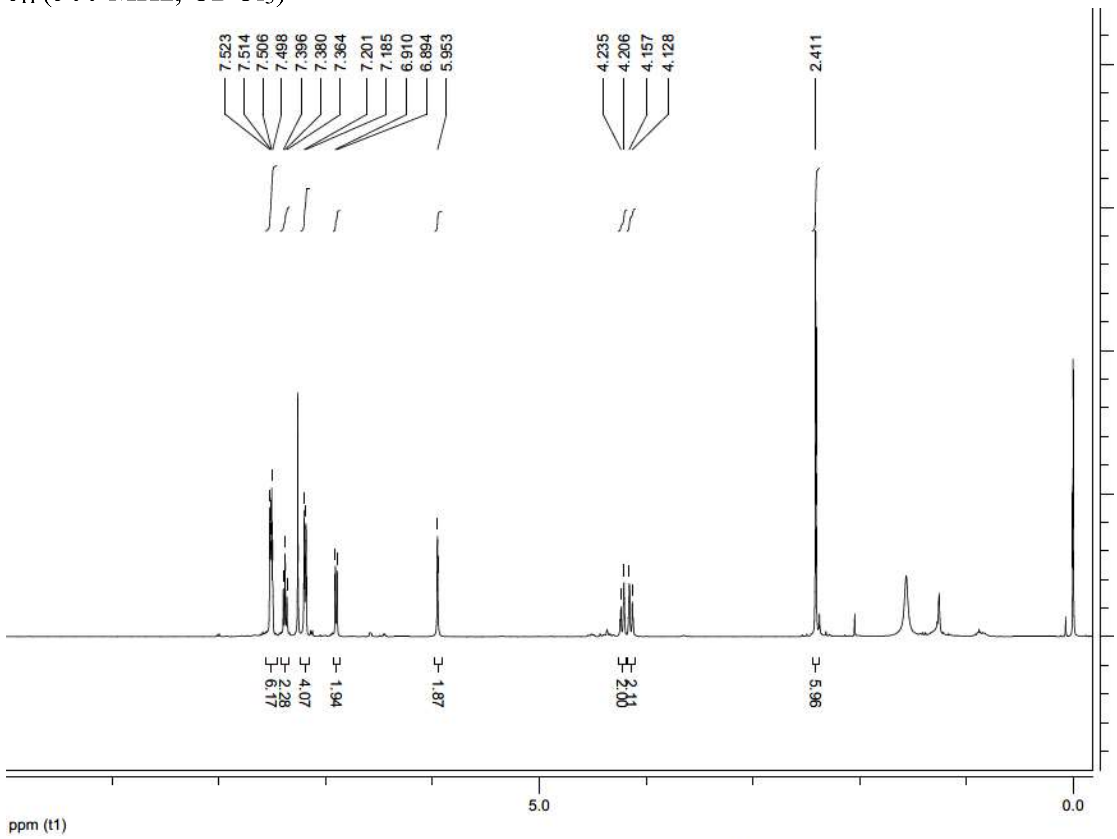

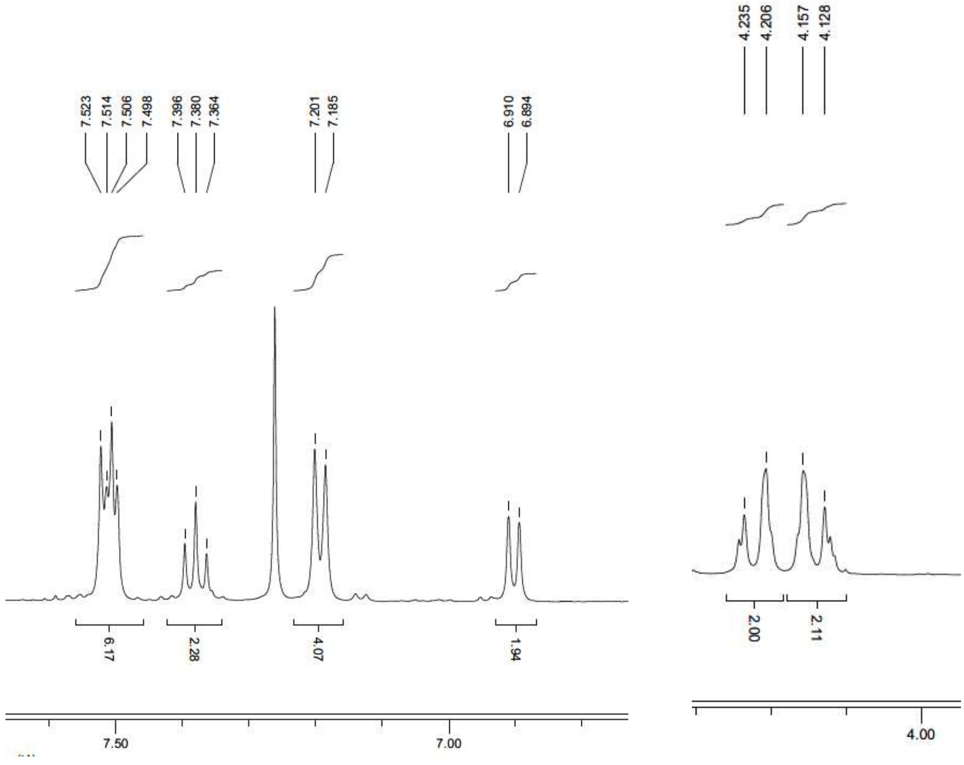

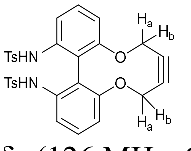

δC (126 MHz, CDCl3)

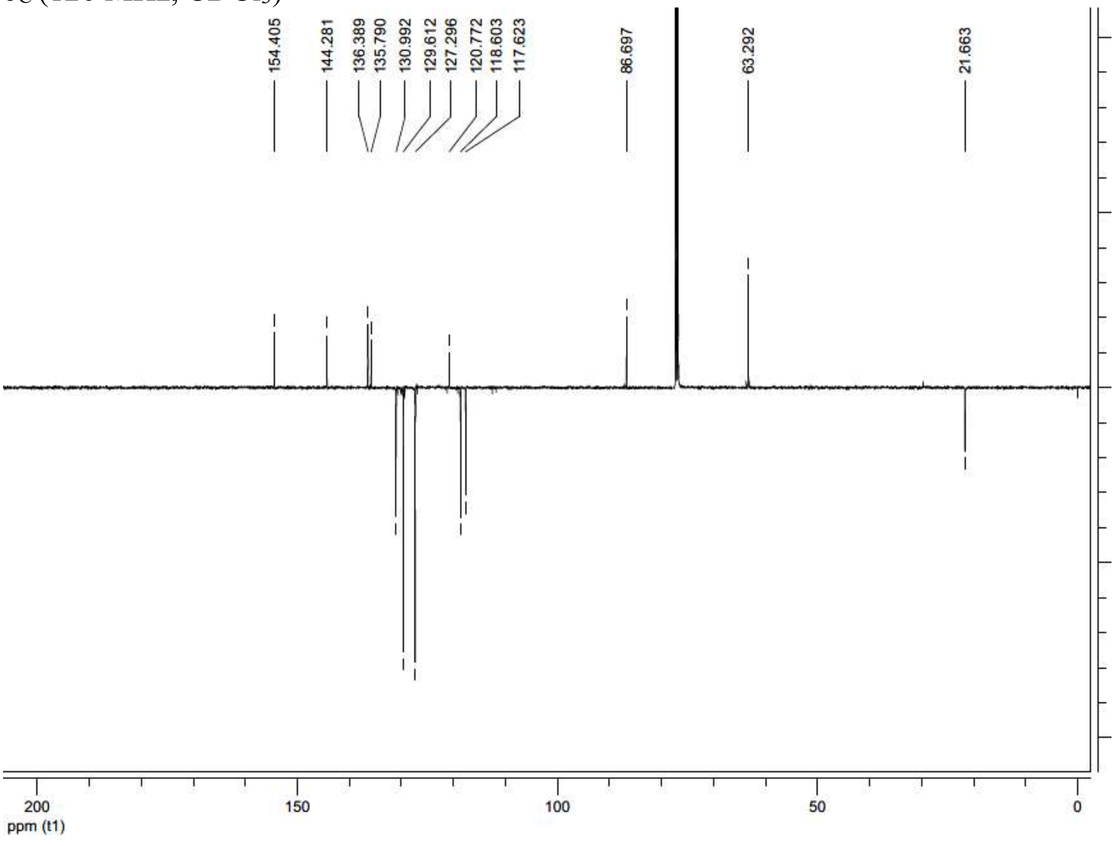

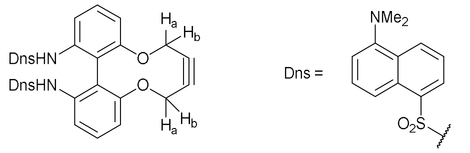

Compound 52.

δ_H (400 MHz, $CDCl_3$)

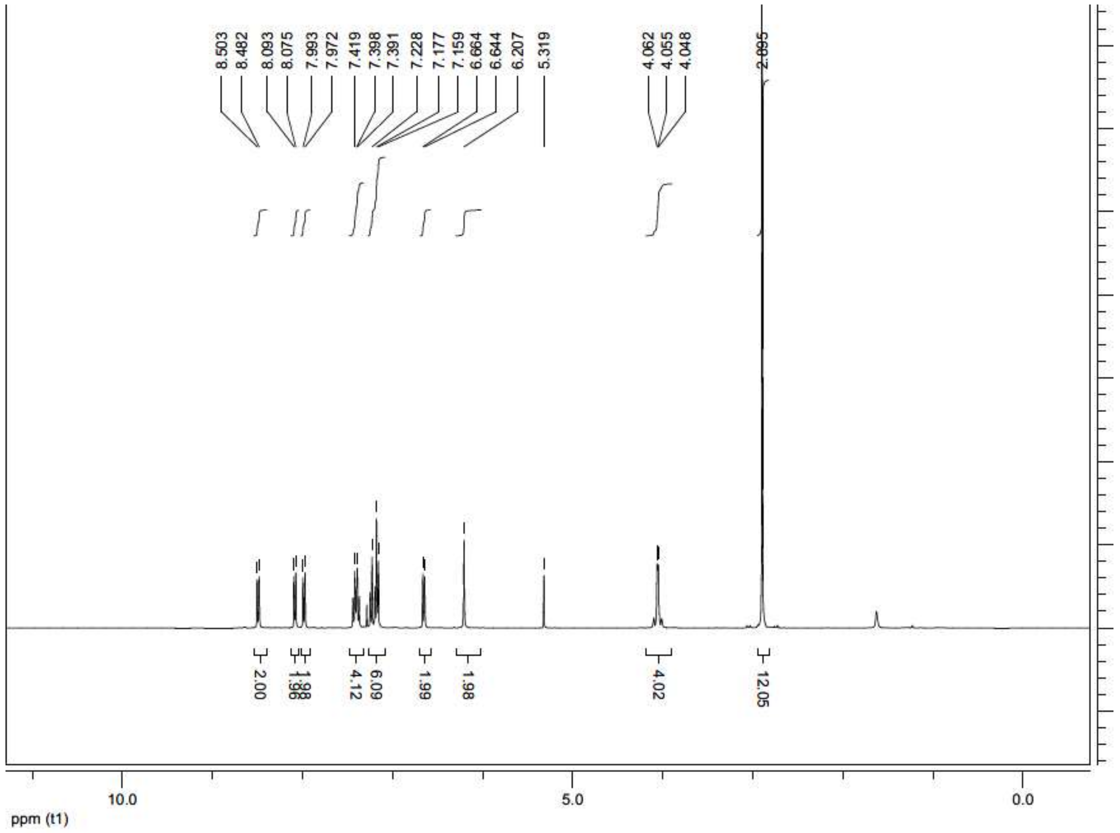

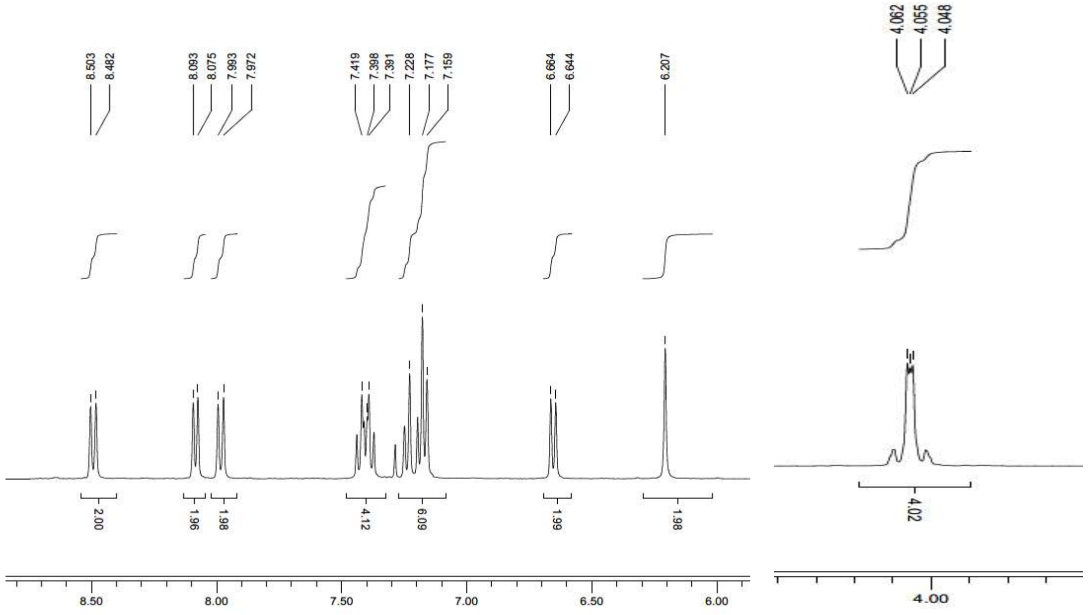

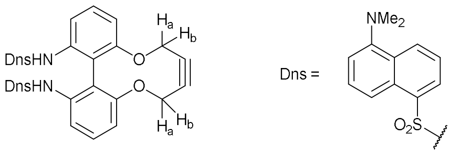

δC (126 MHz, CDCl3)

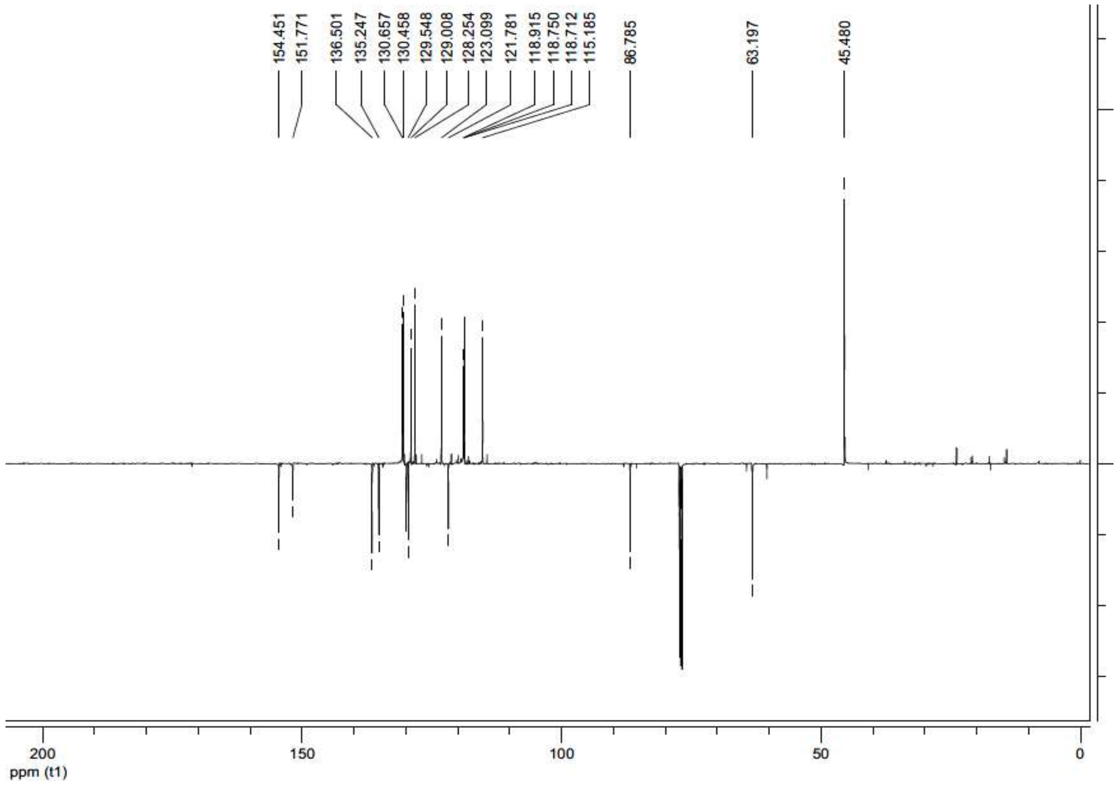

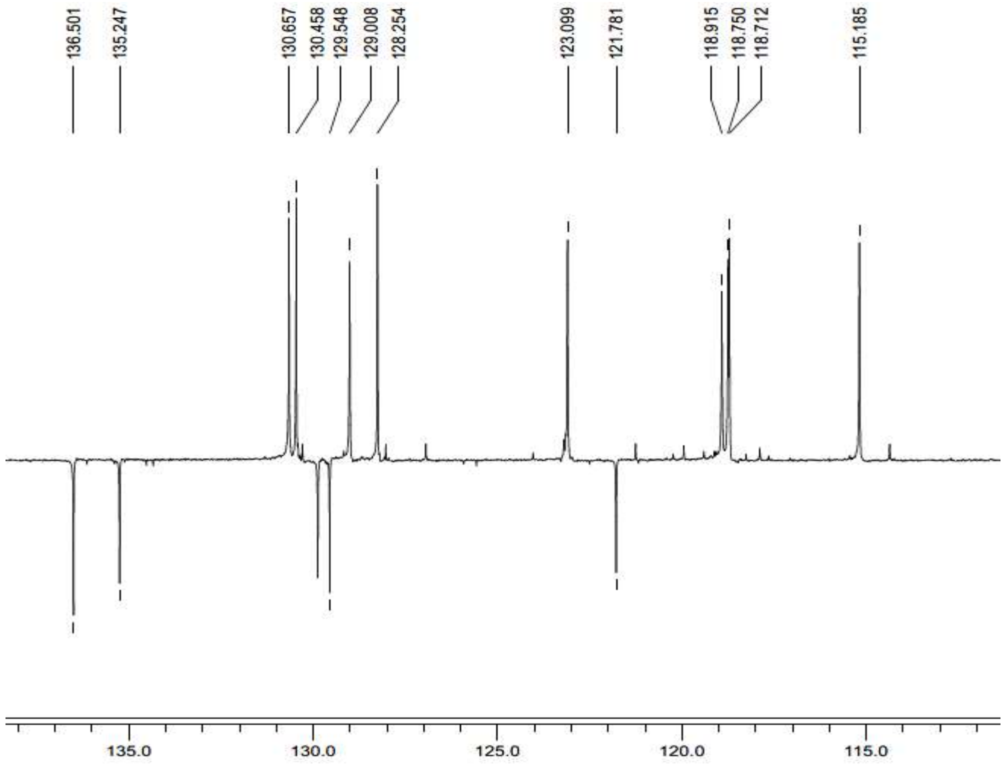

Compound 53.

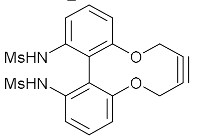

δ_H (500 MHz, $CDCl_3$)

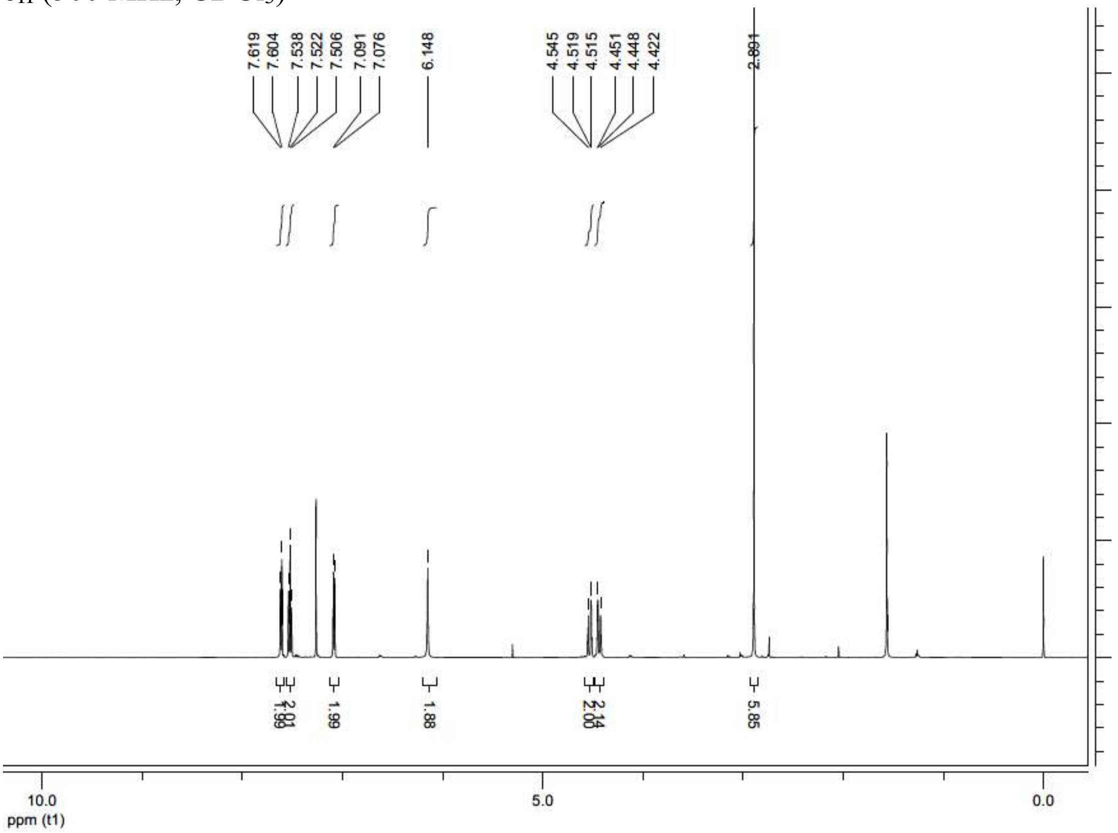

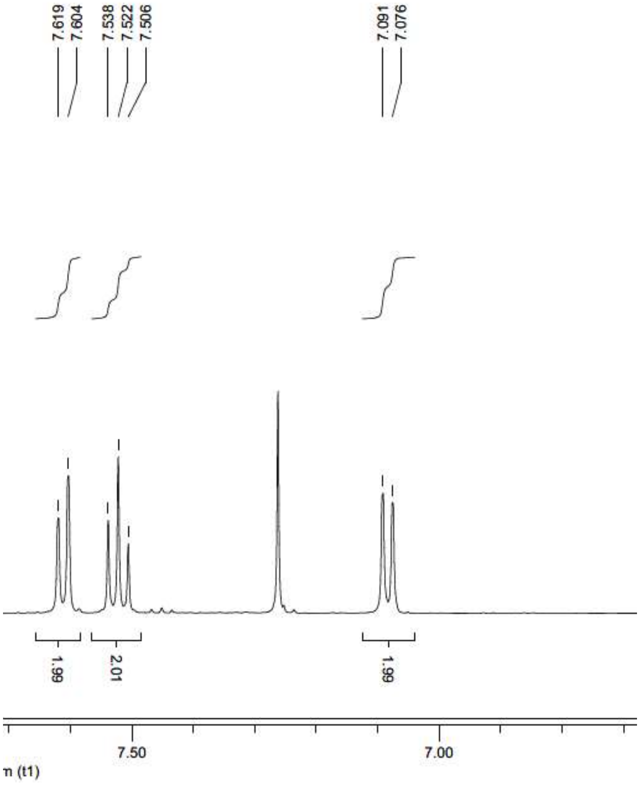

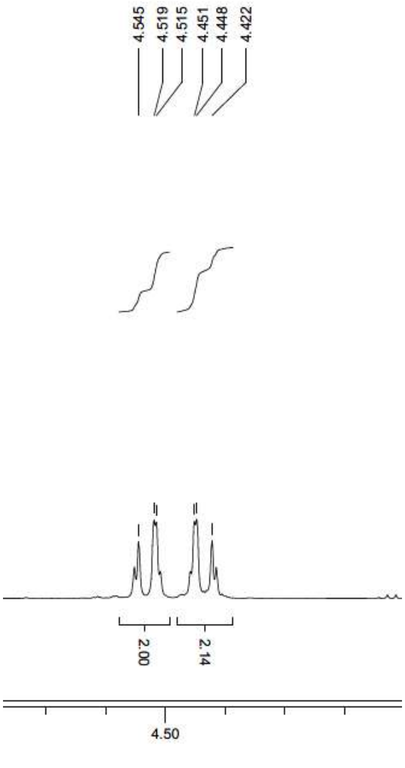

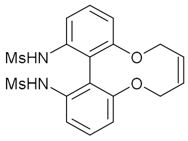

δ$_C$ (125 MHz, $CDCl_3$)

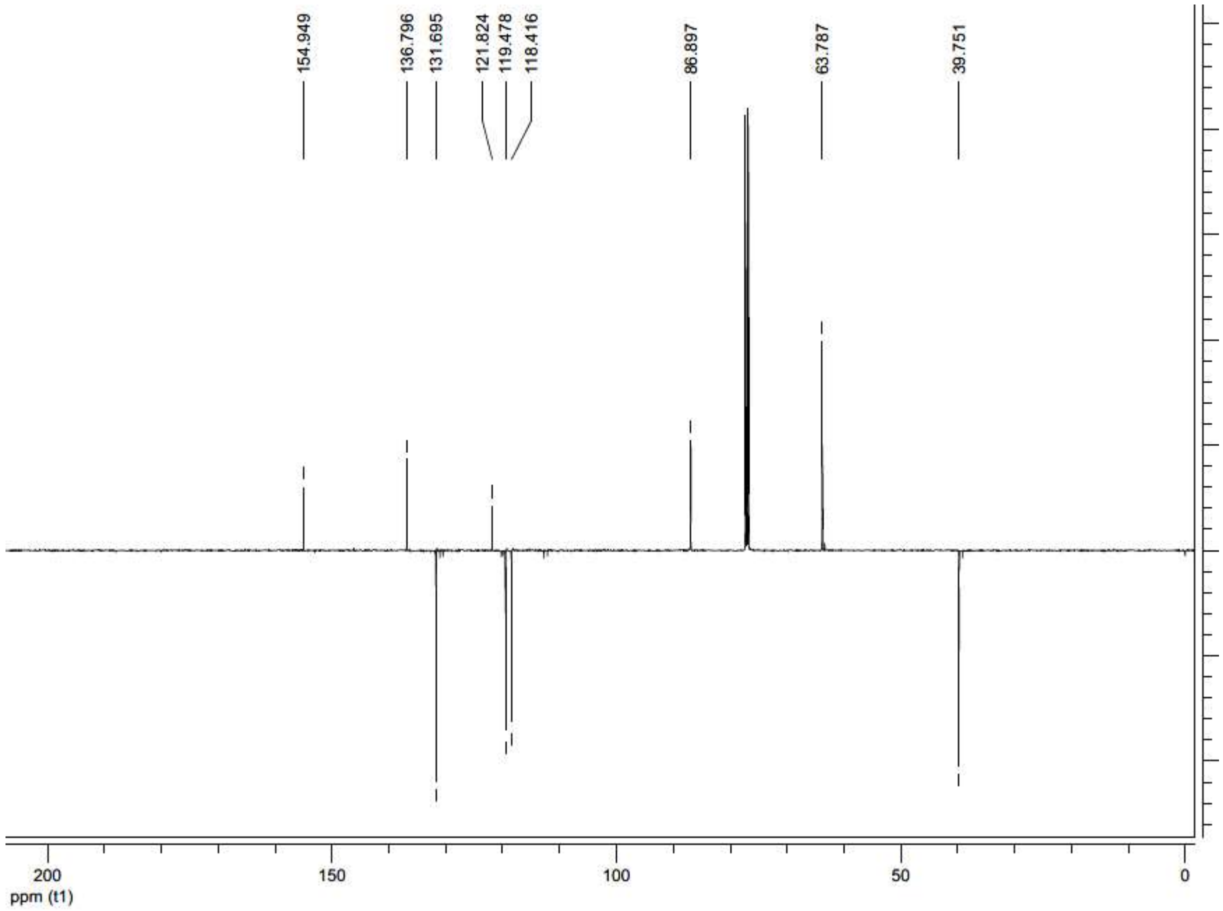

Compound 54.

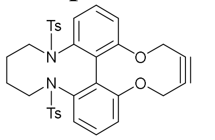

δ_H (500 MHz, $CDCl_3$)

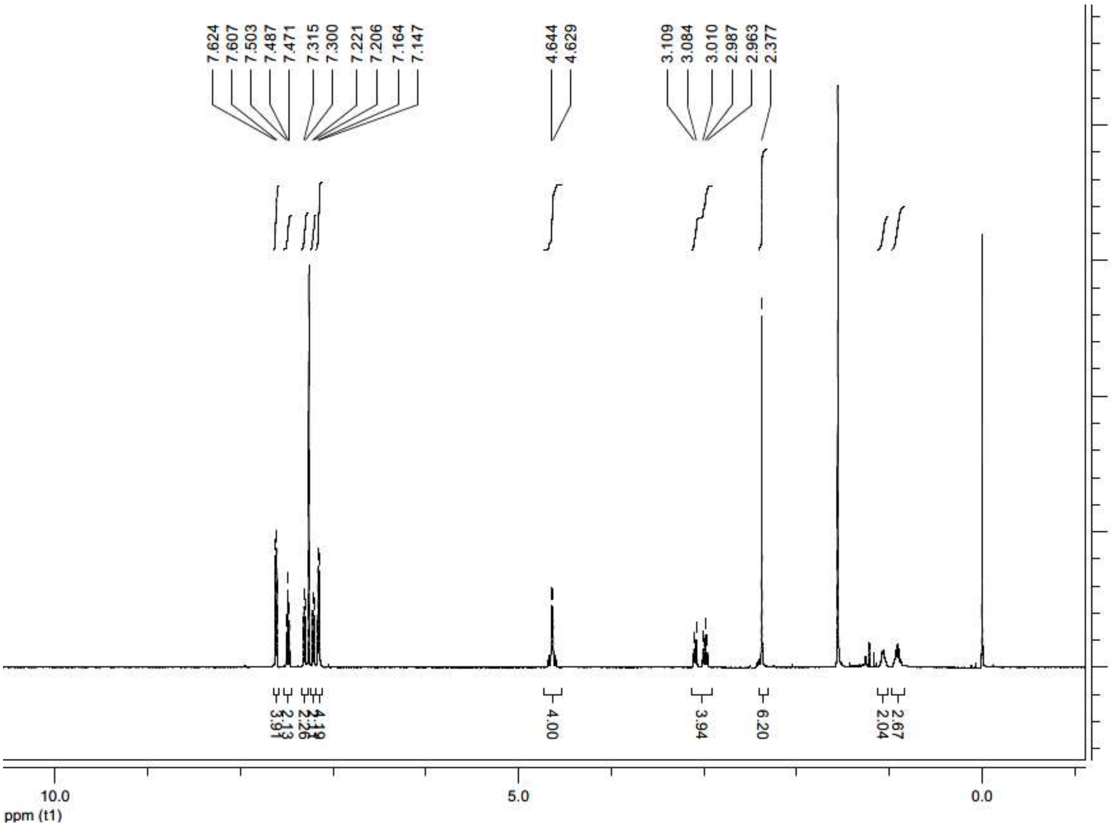

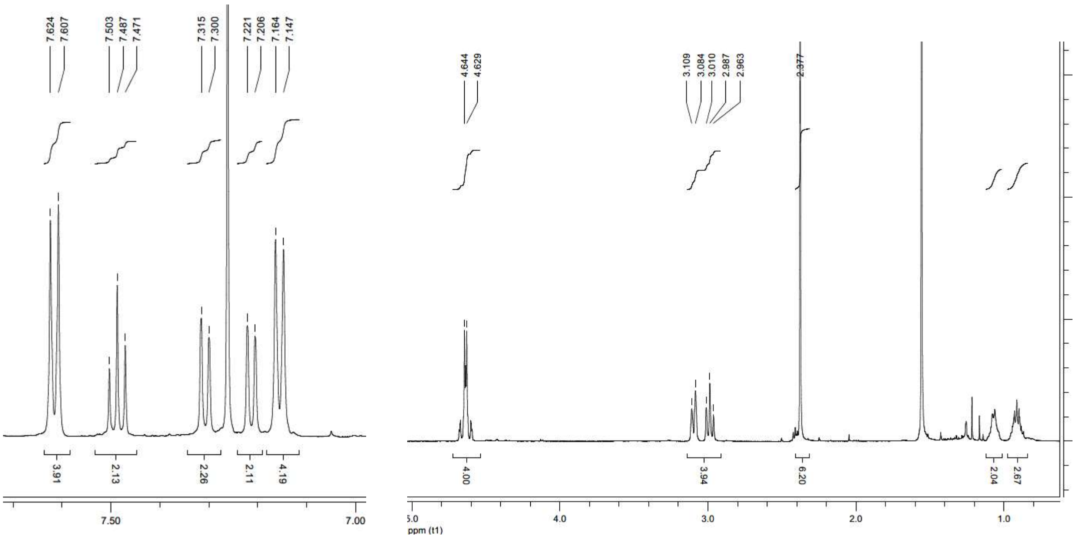

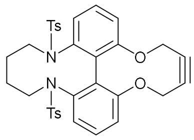

δ_C (126 MHz, $CDCl_3$)

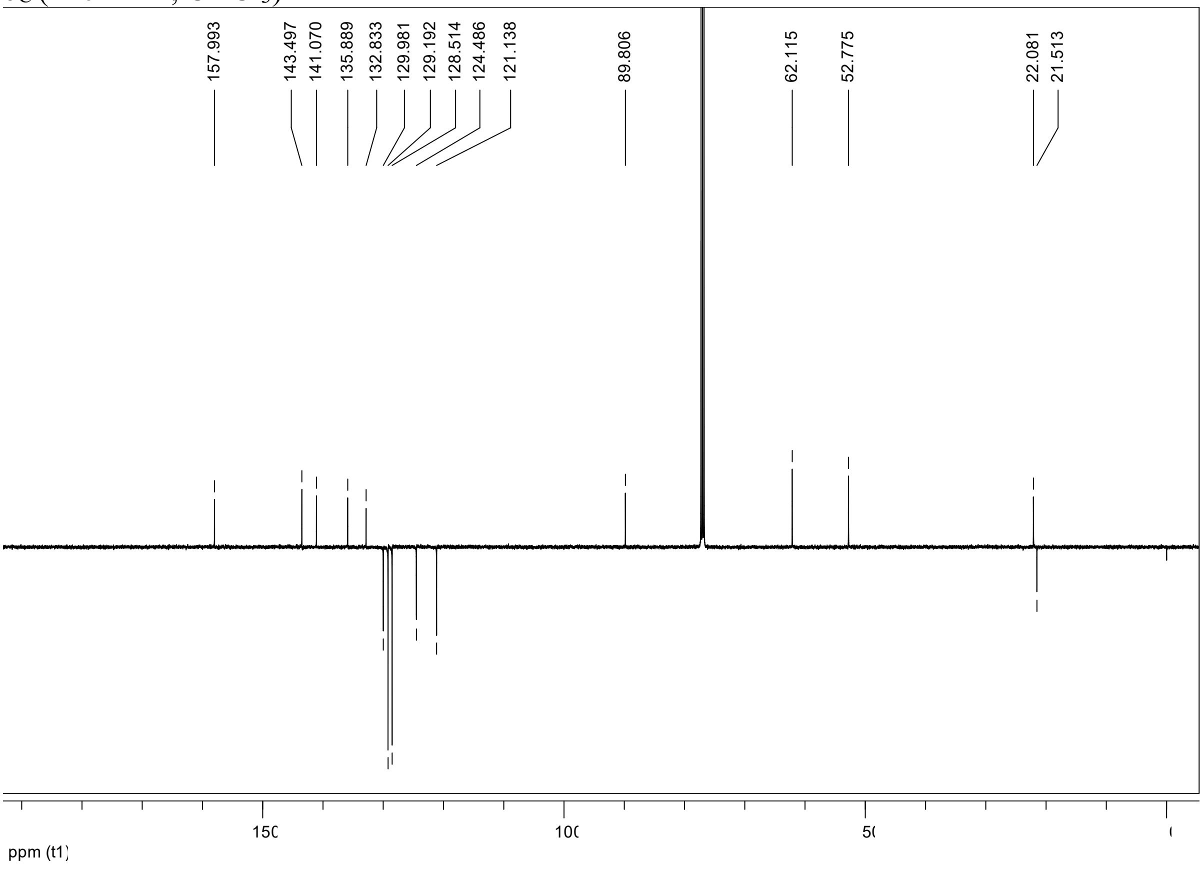

Compound 56.

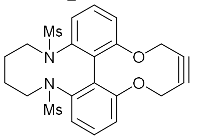

δ_H (500 MHz, $CDCl_3$)

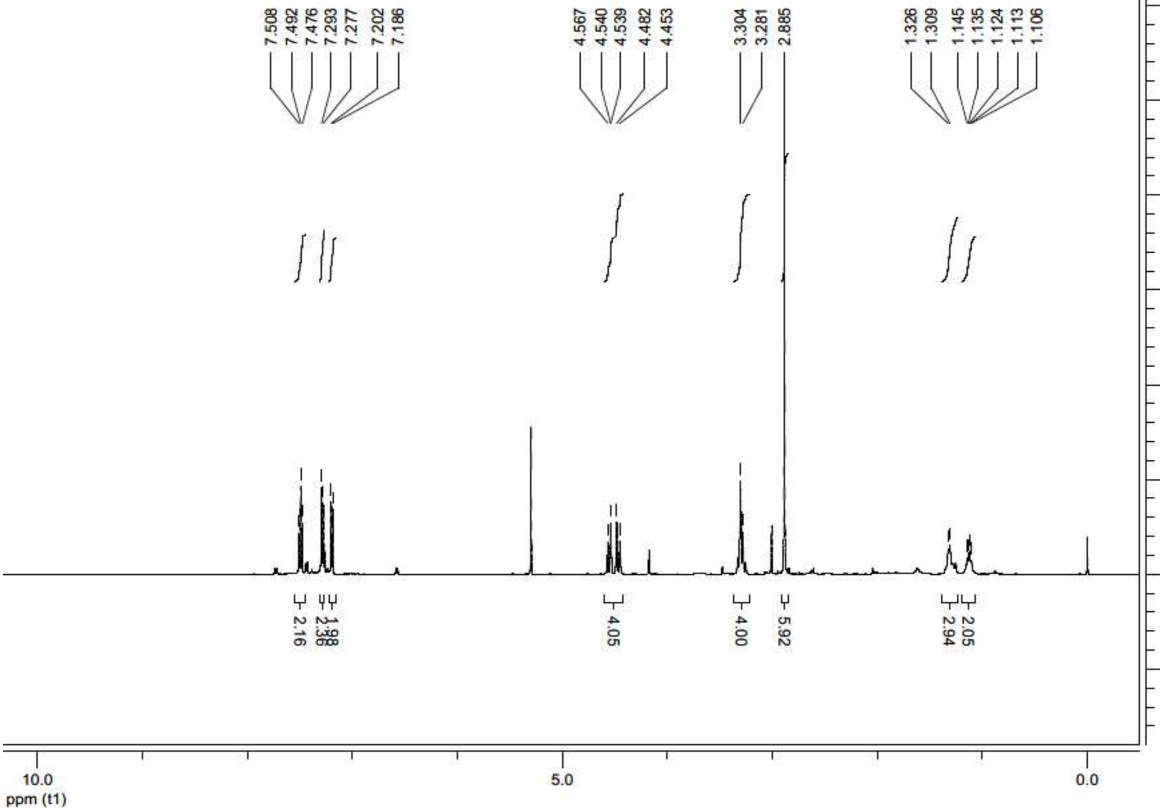

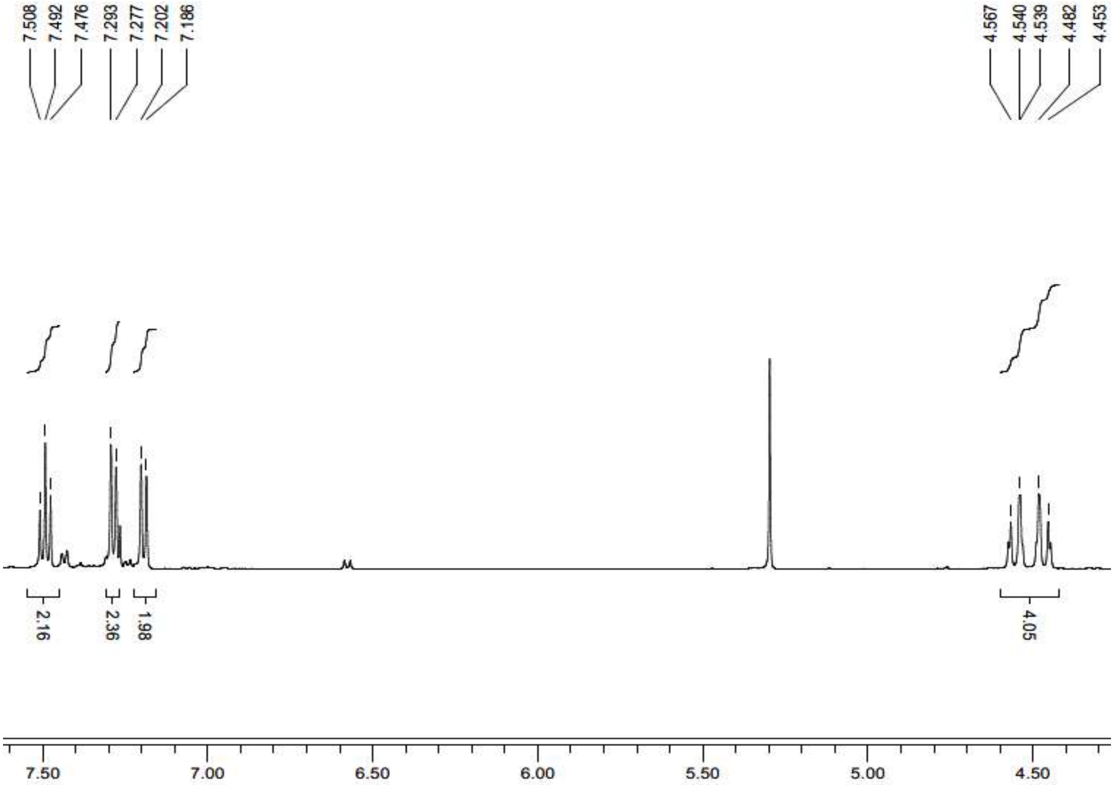

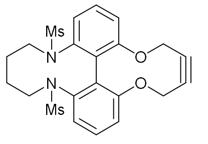

δC (125 MHz, $CDCl_3$)

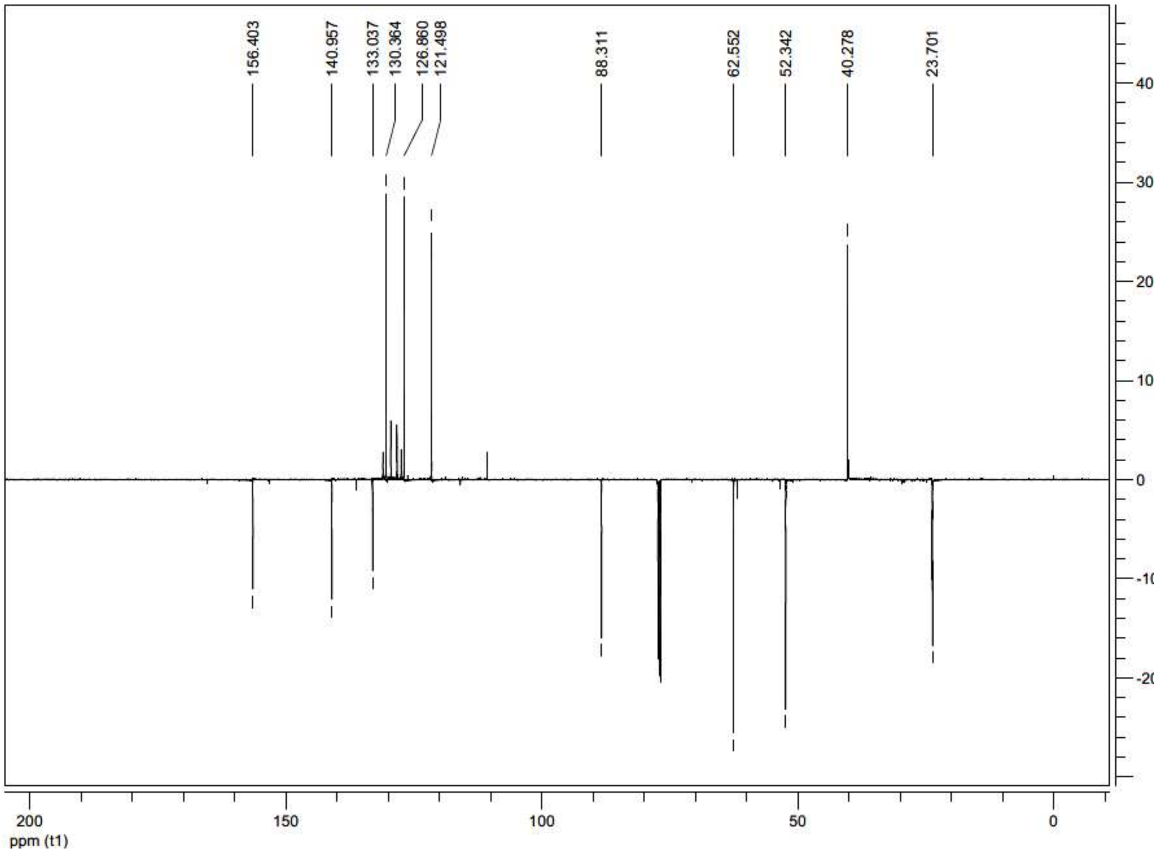

Compound 57.

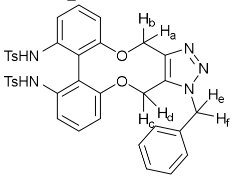

δ_H (500 MHz, $CDCl_3$)

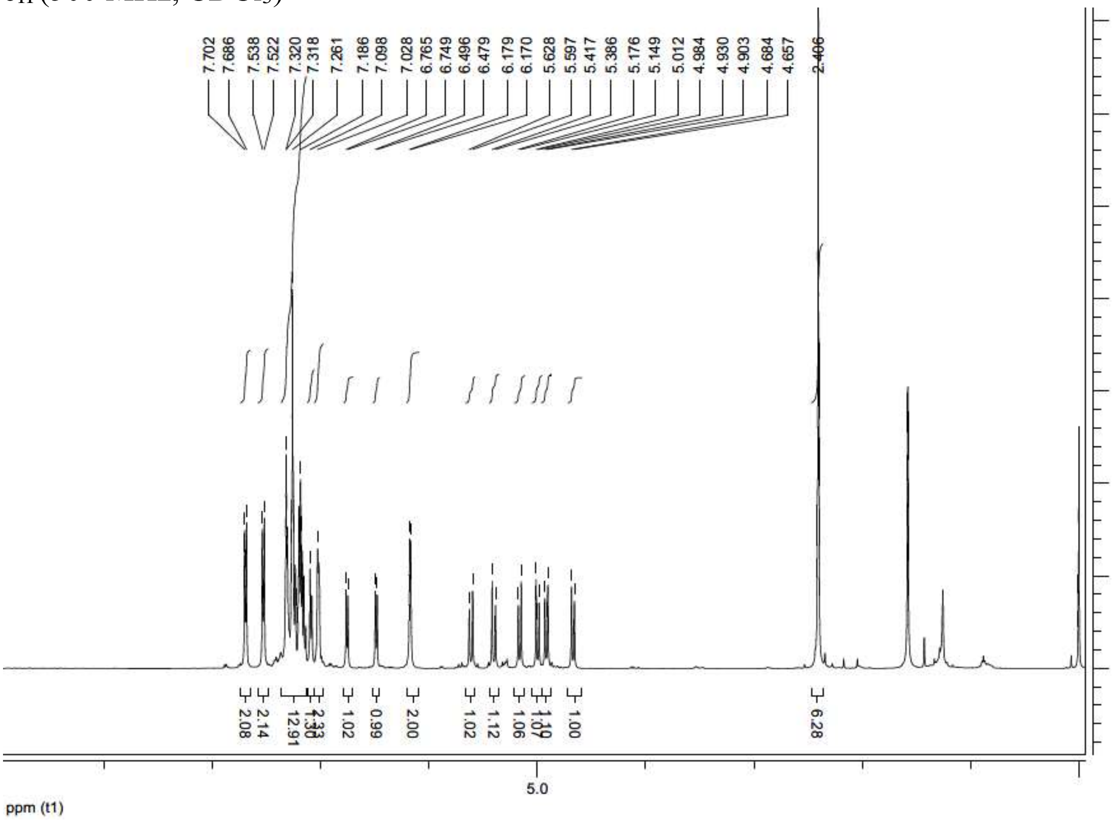

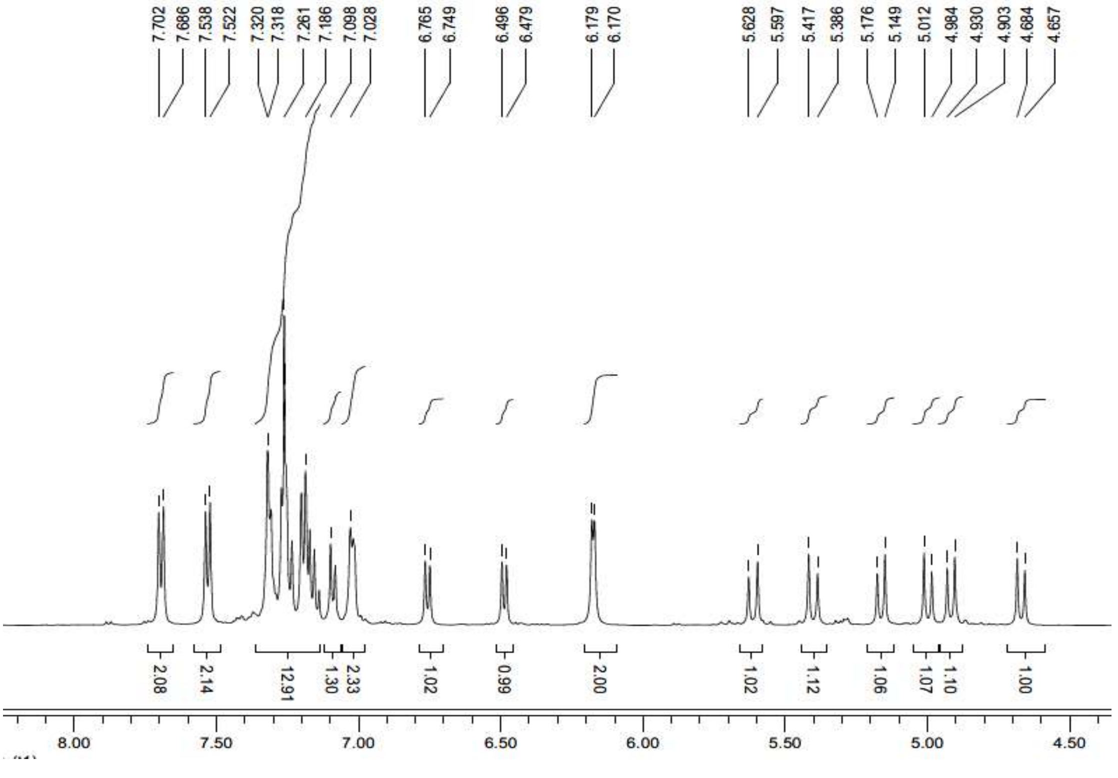

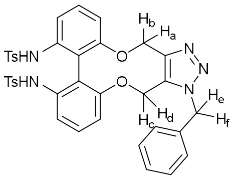

δC (126 MHz, CDCl3)

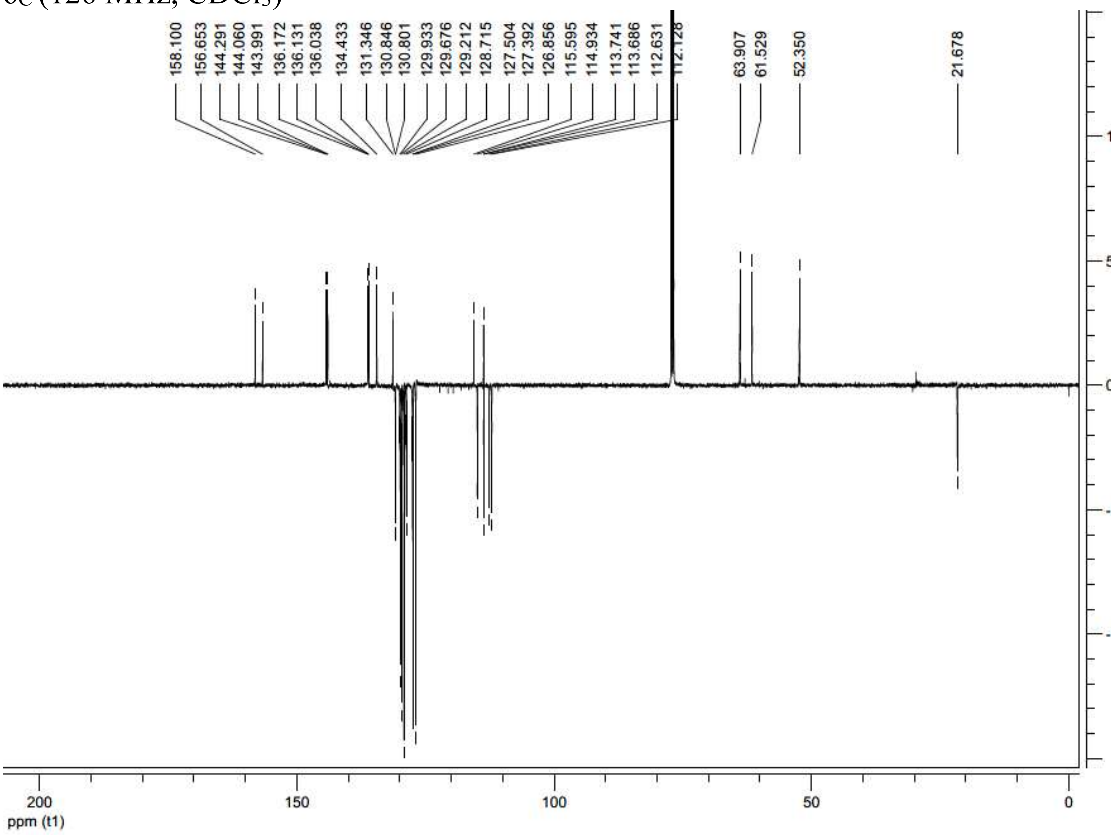

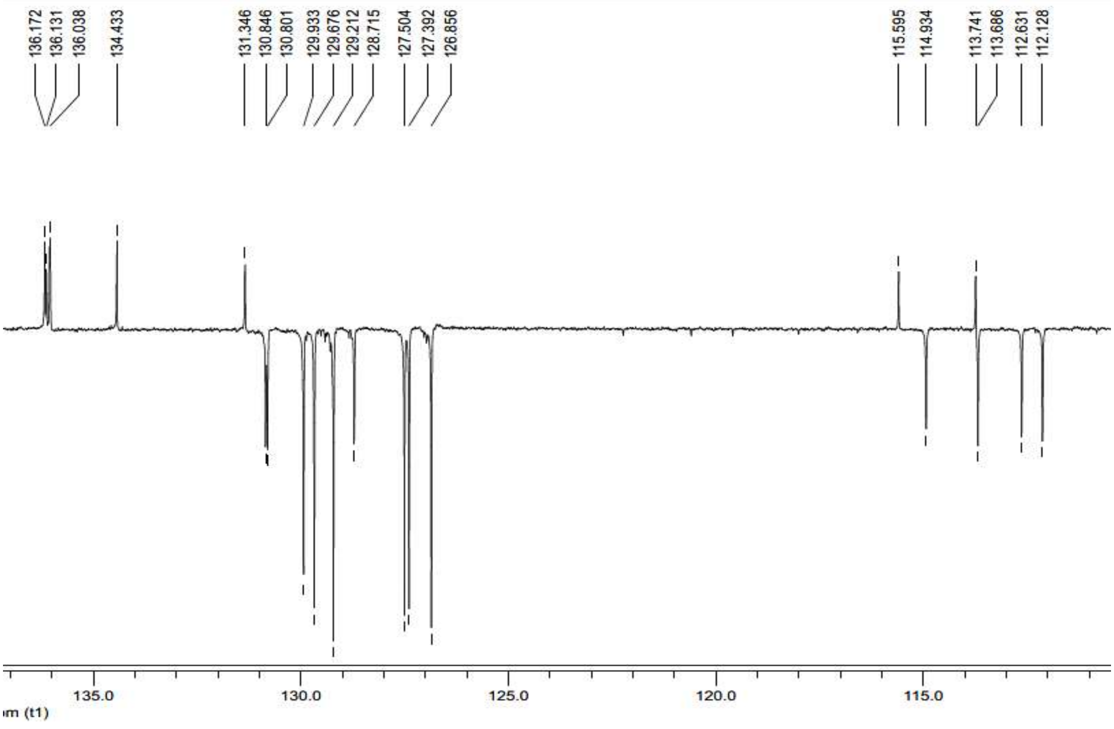

Compound 59.

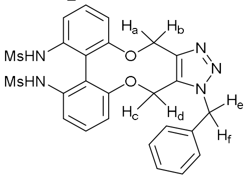

δ_H (500 MHz, $CDCl_3$)

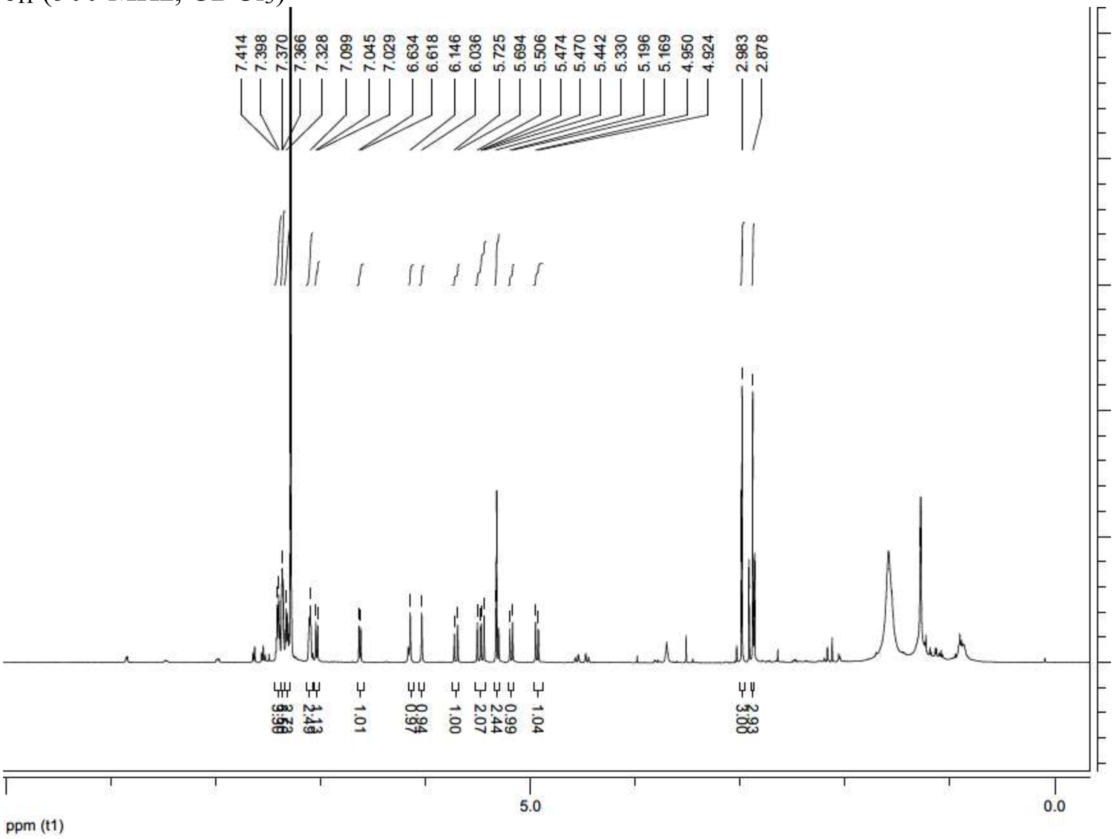

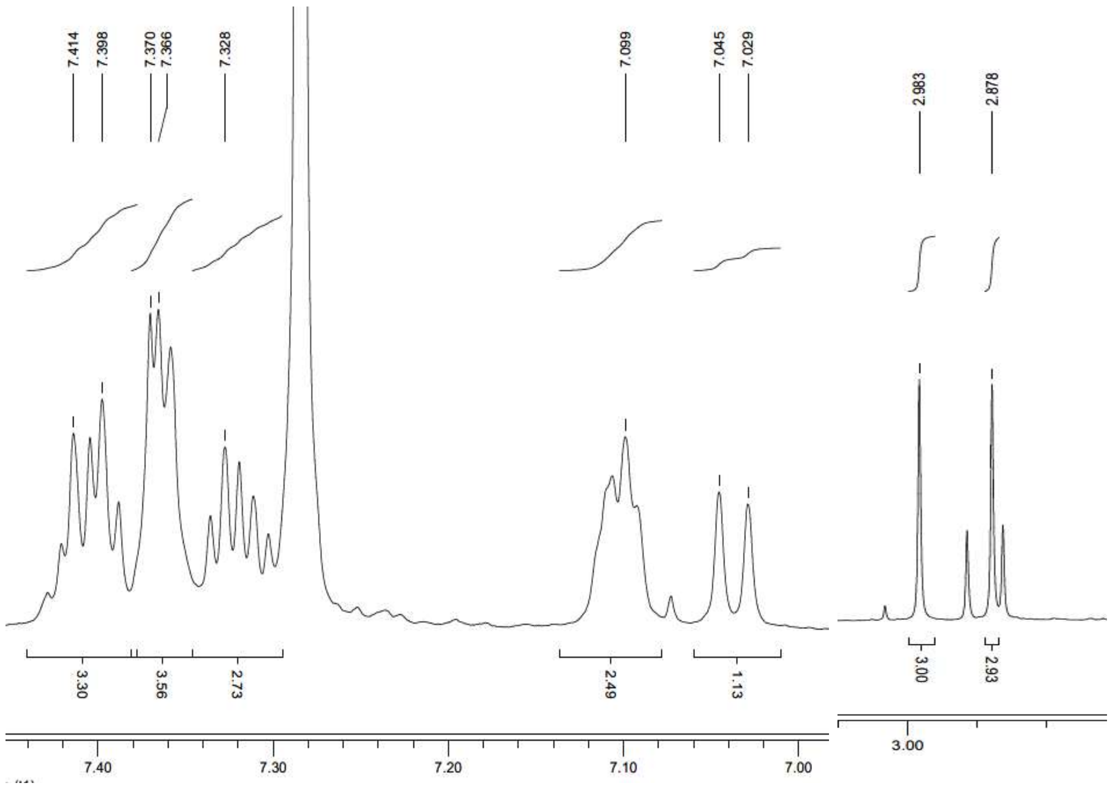
7.414
7.398
7.370
7.366
7.328
7.099
7.045
7.029
2.983
2.878
3.30
3.56
2.73
2.49
1.13
3.00
2.93
7.40
7.30
7.20
7.10
7.00
3.00

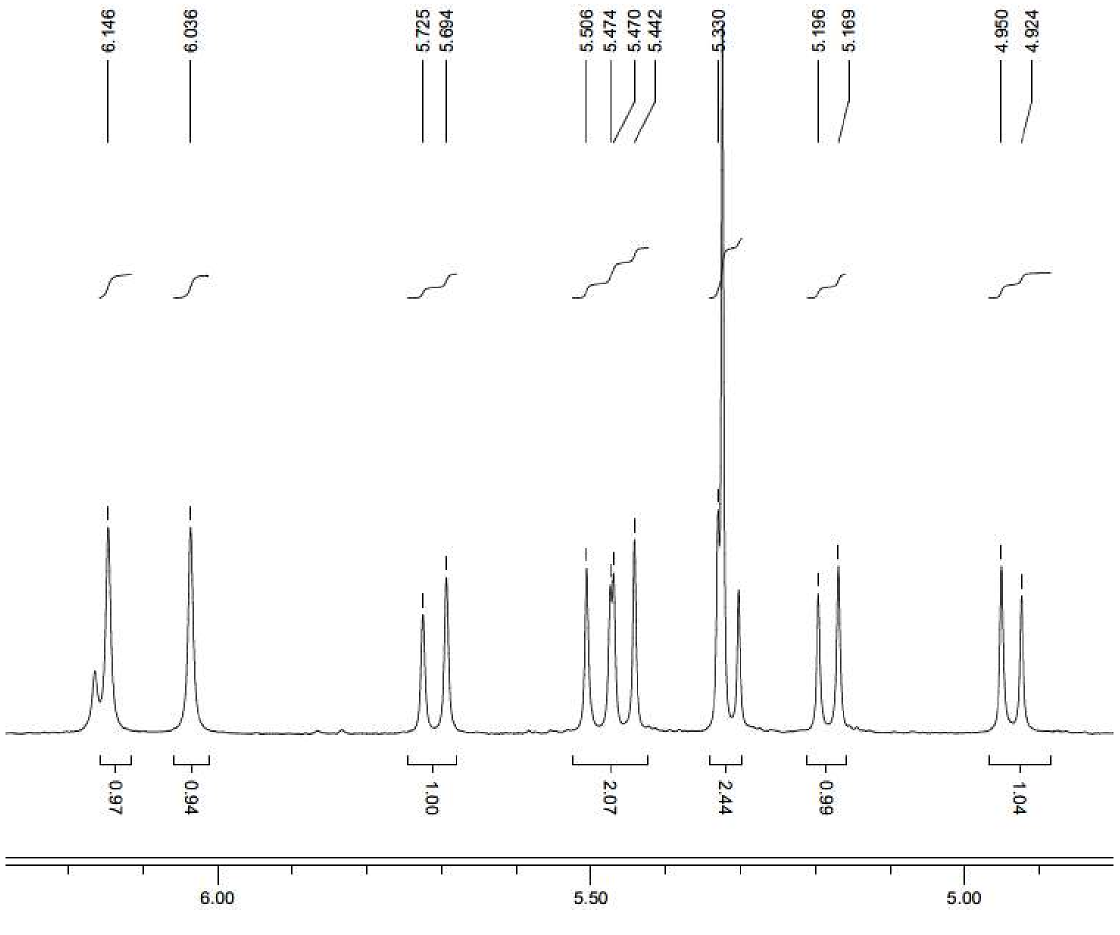
6.146
6.036
5.725
5.694
5.506
5.474
5.470
5.442
5.330
5.196
5.169
4.950
4.924
0.97
0.94
1.00
2.07
2.44
0.99
1.04
6.00
5.50
5.00

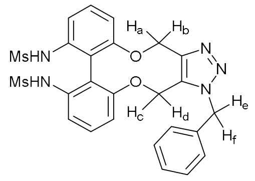

δ_C (126 MHz, $CDCl_3$)

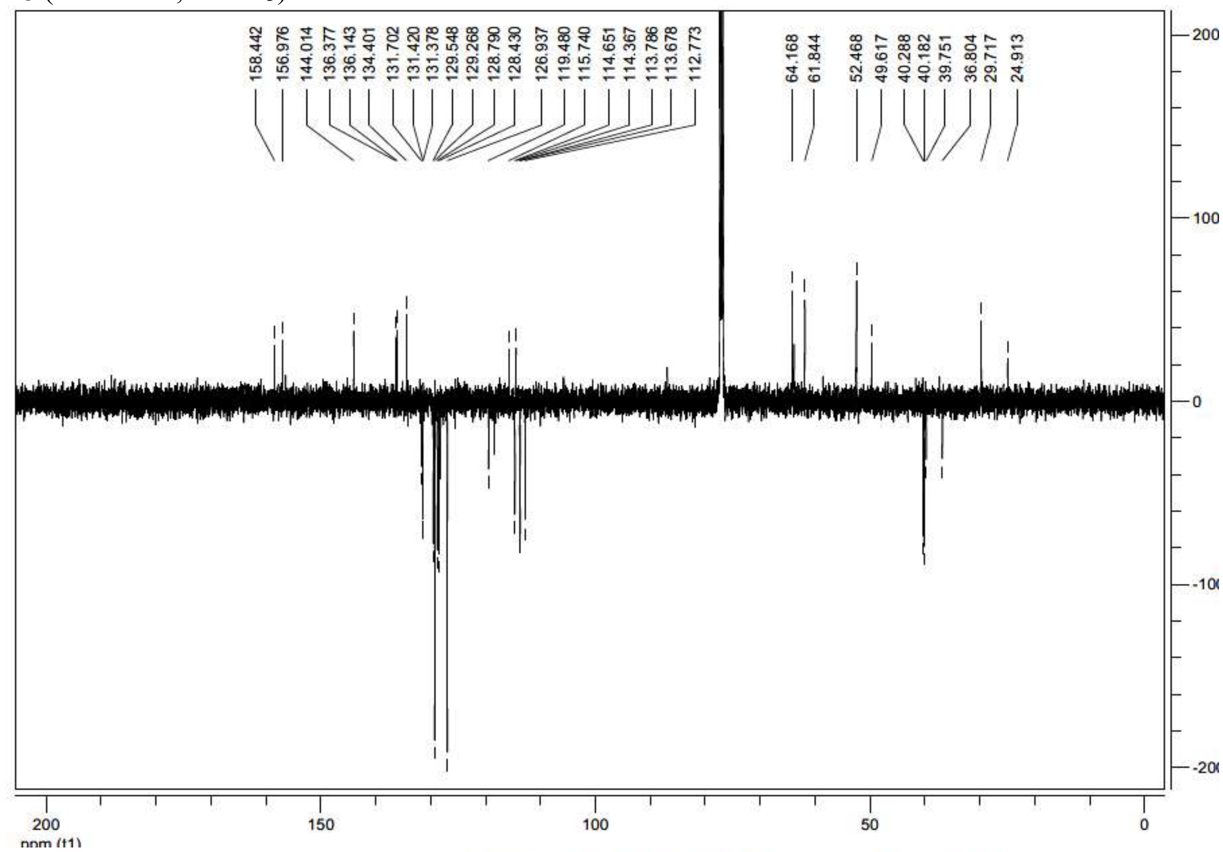

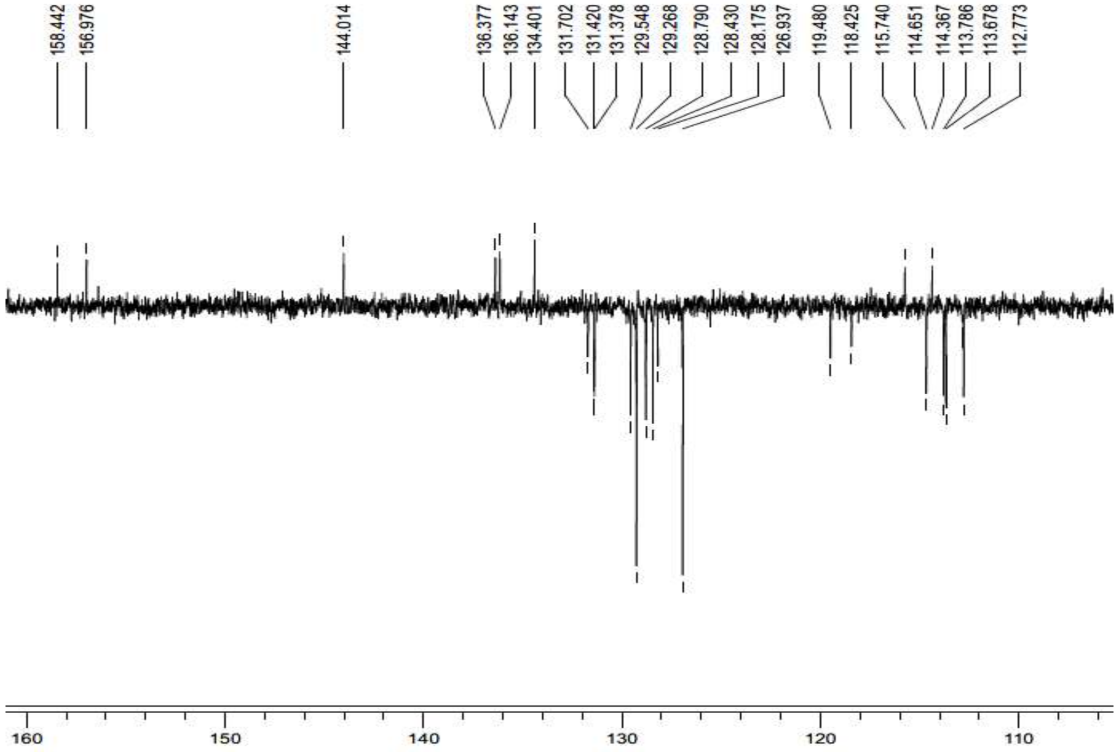

Compound 60.

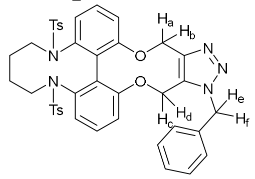

δ_H (500 MHz, $CDCl_3$)

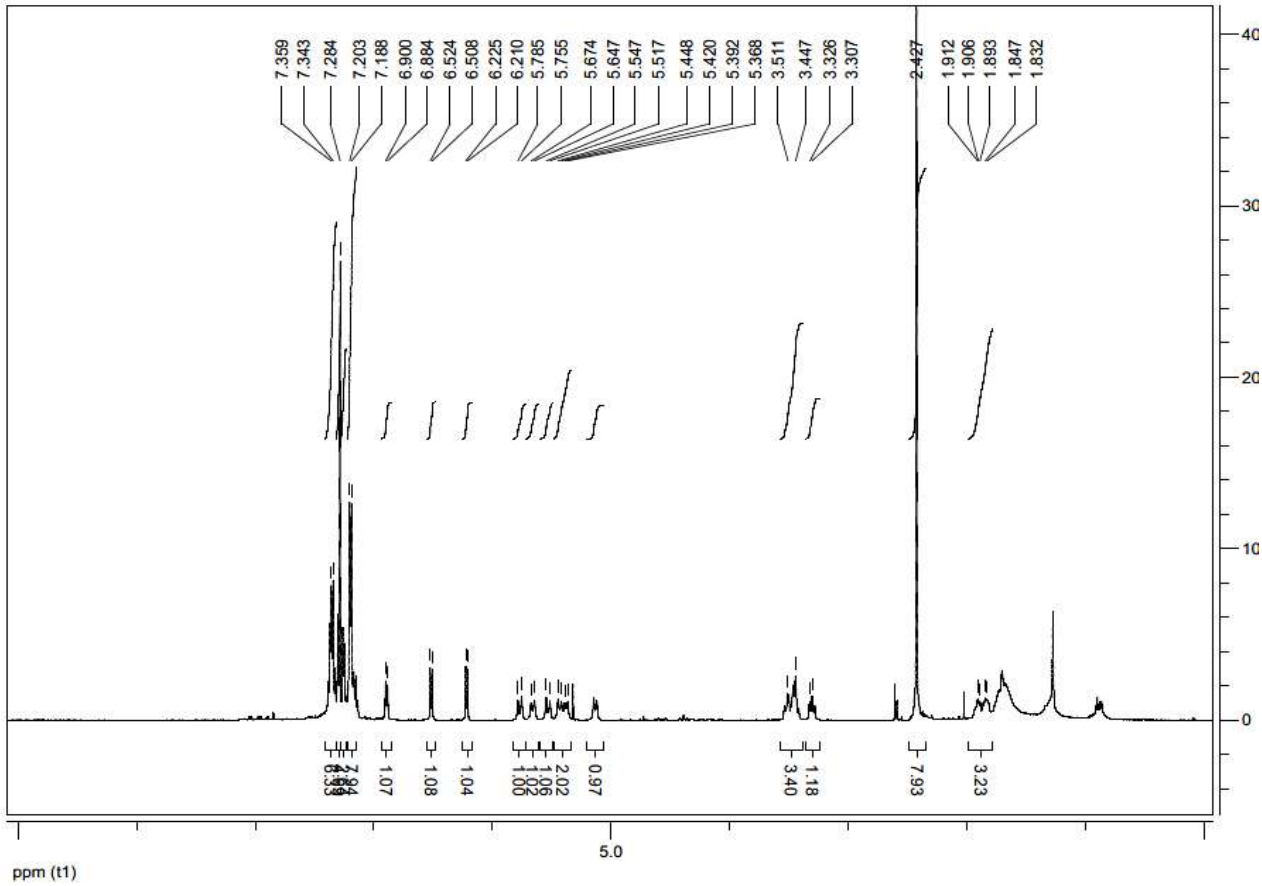

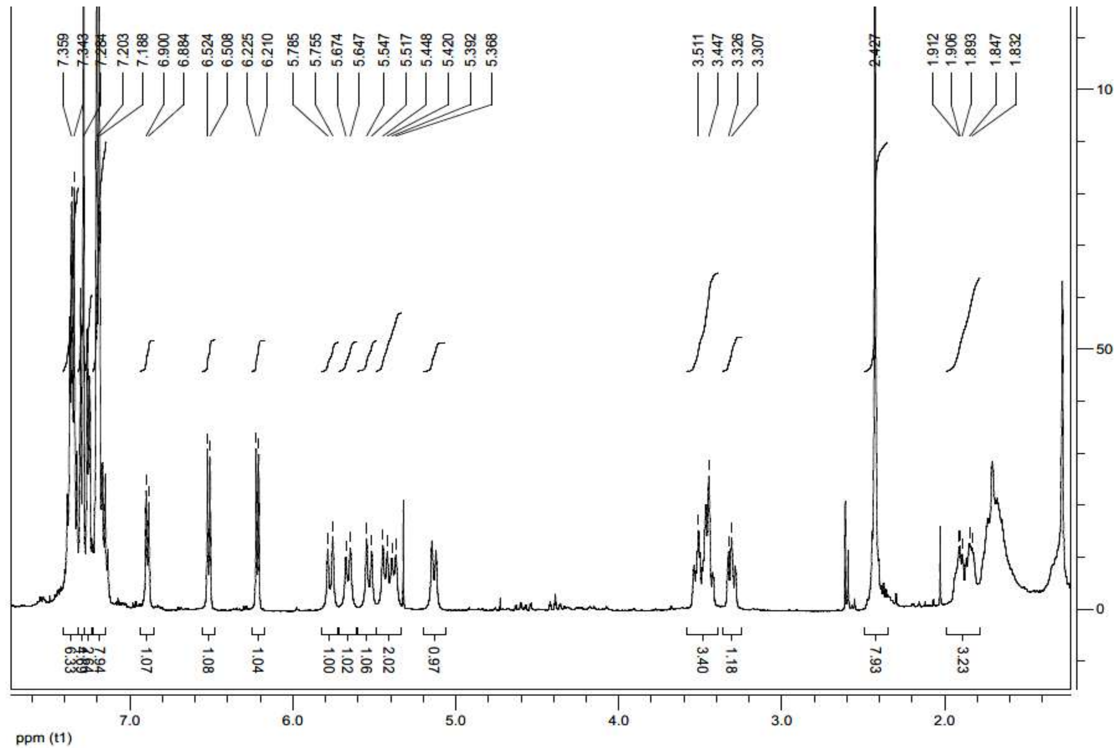

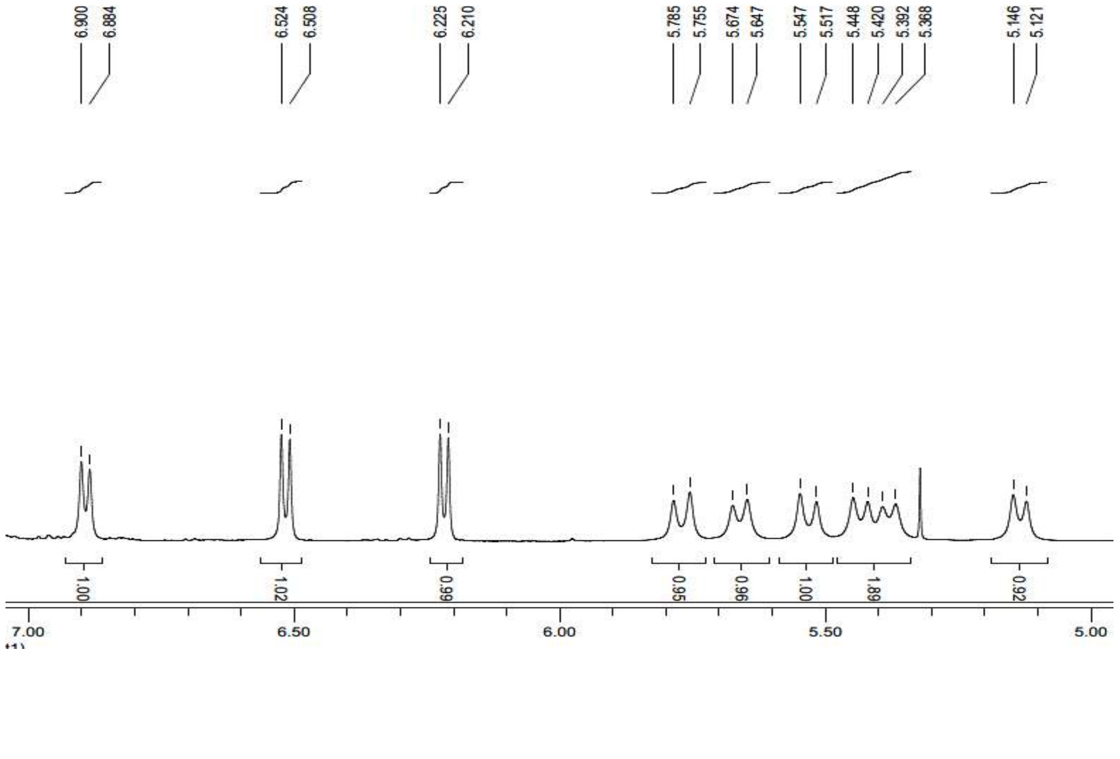

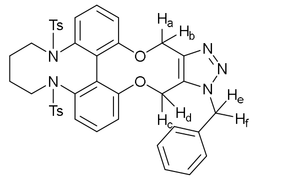

δ_C (126 MHz, $CDCl_3$)

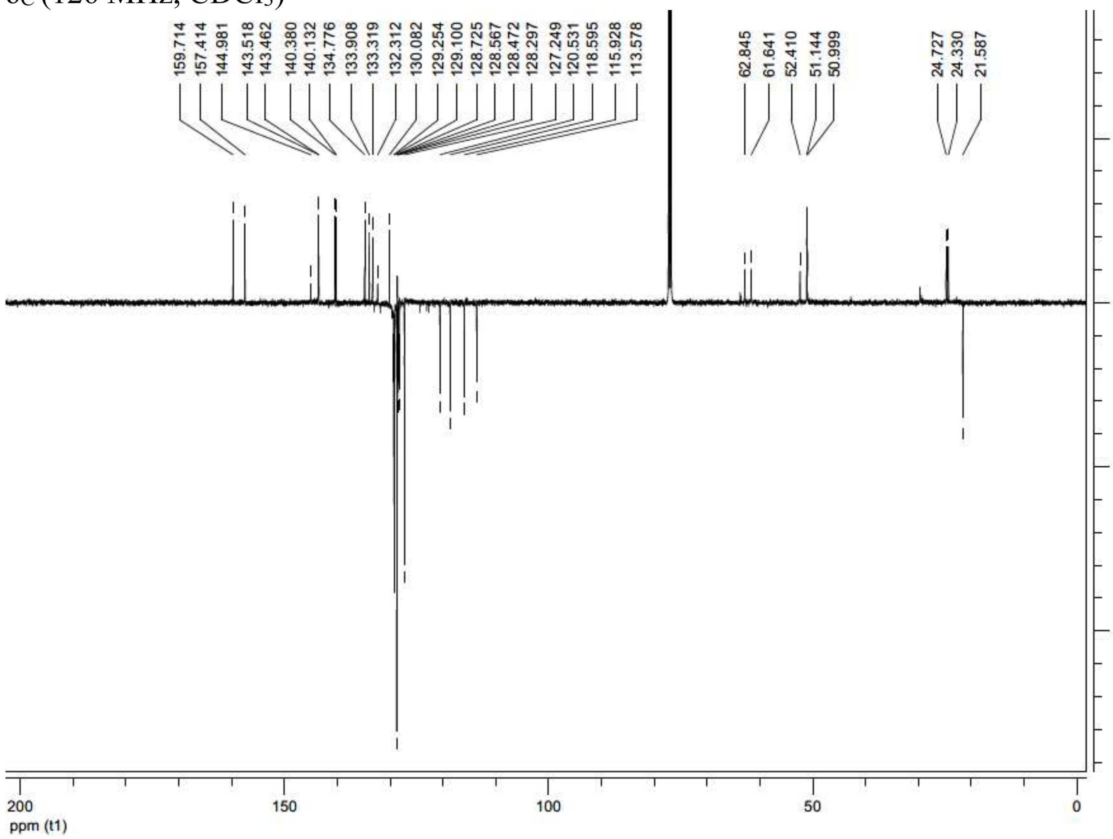

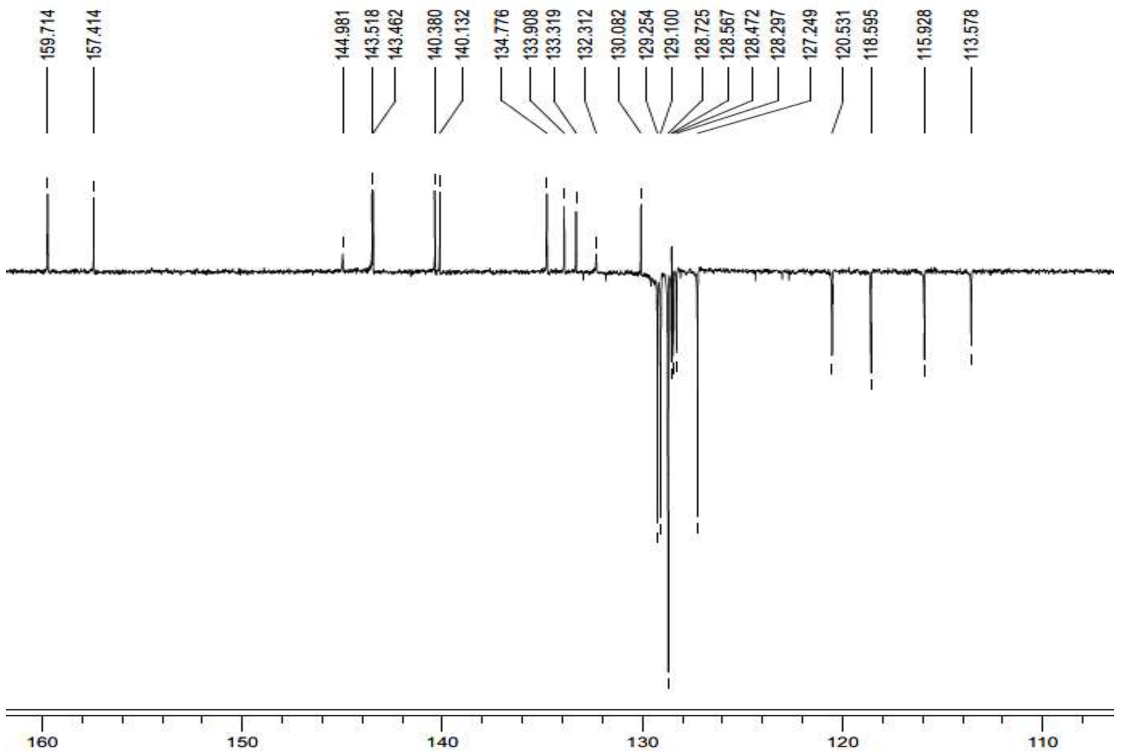

Compound 61.

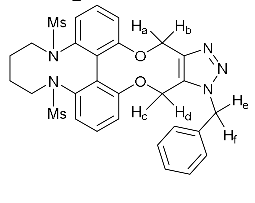

δH (500 MHz, CDCl3)

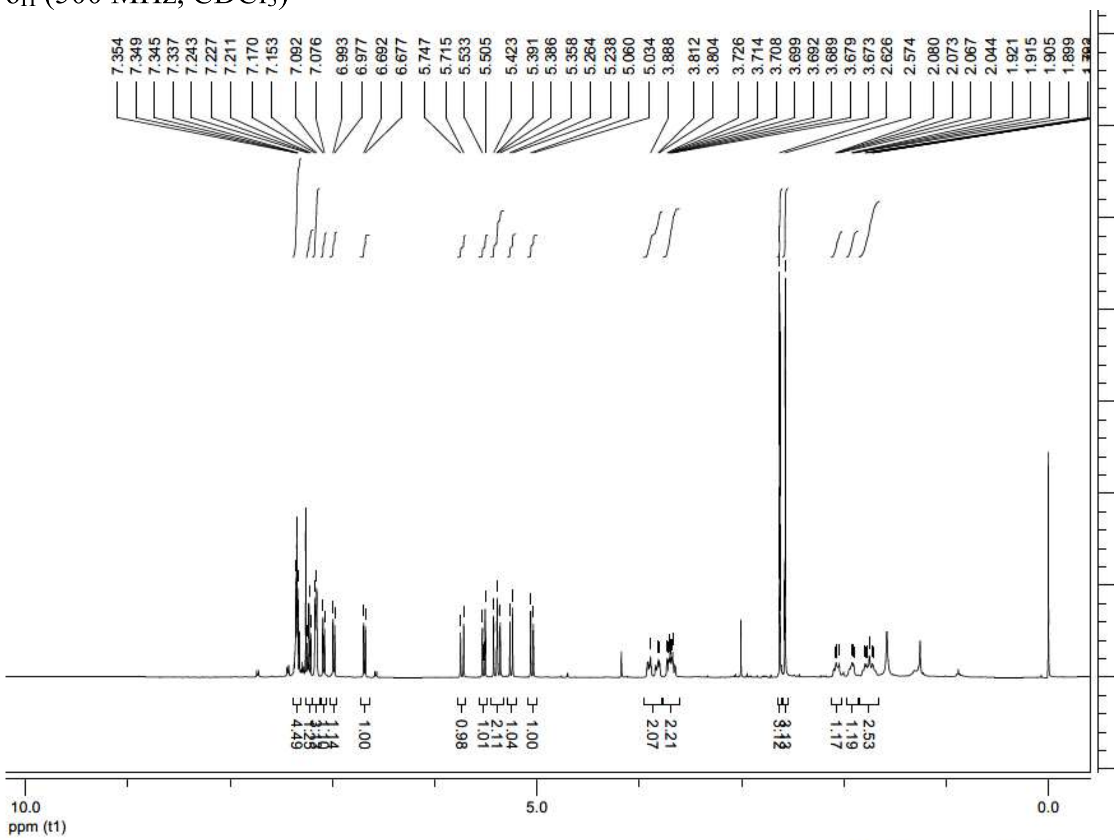

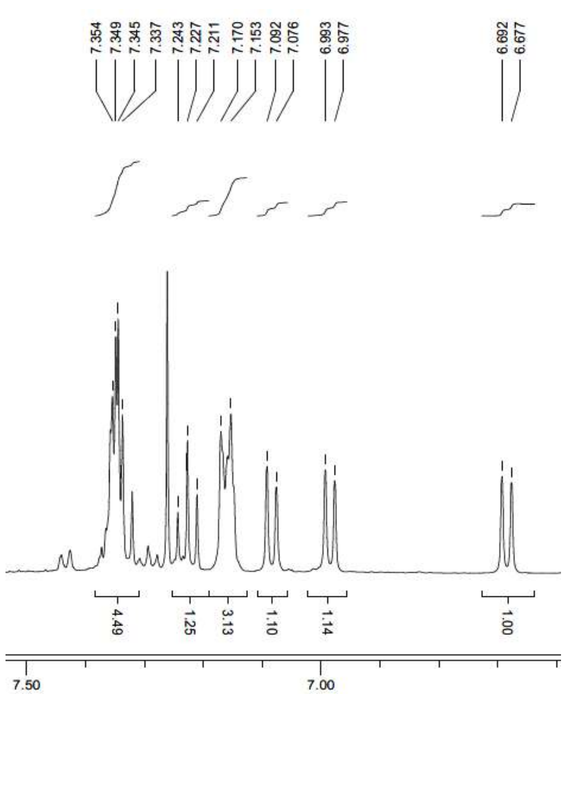

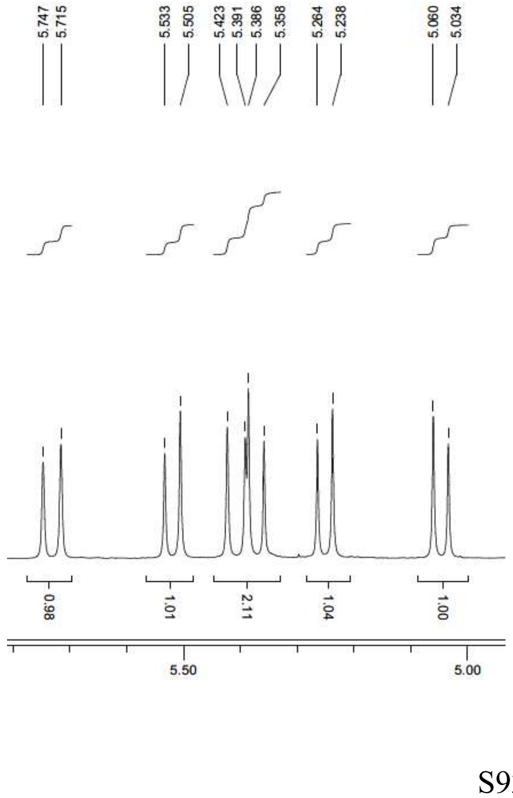

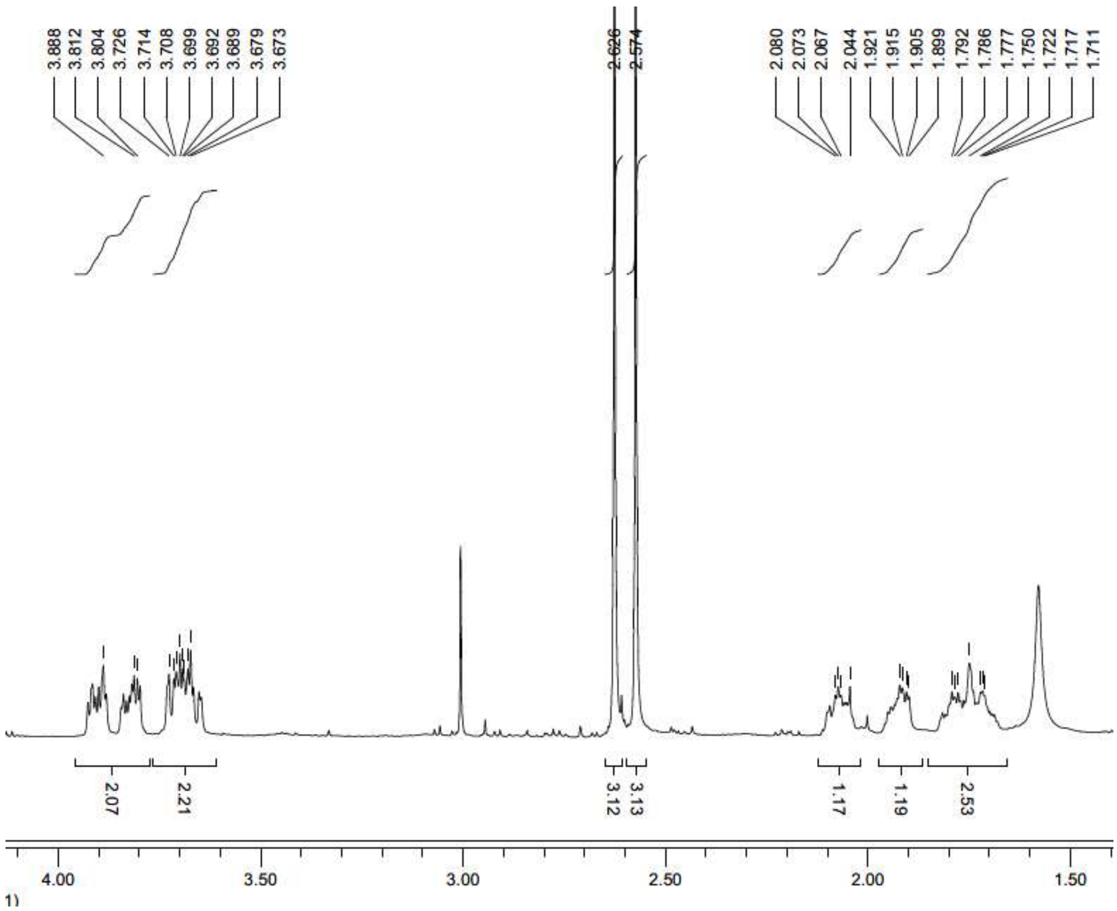
3.888
3.812
3.804
3.726
3.714
3.708
3.699
3.692
3.689
3.679
3.673
2.626
2.574
2.080
2.073
2.067
2.044
1.921
1.915
1.905
1.899
1.792
1.786
1.777
1.750
1.722
1.717
1.711
2.07
2.21
3.12
3.13
1.17
1.19
2.53
4.00
3.50
3.00
2.50
2.00
1.50

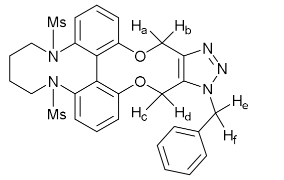

δ$_C$ (125 MHz, $CDCl_3$)

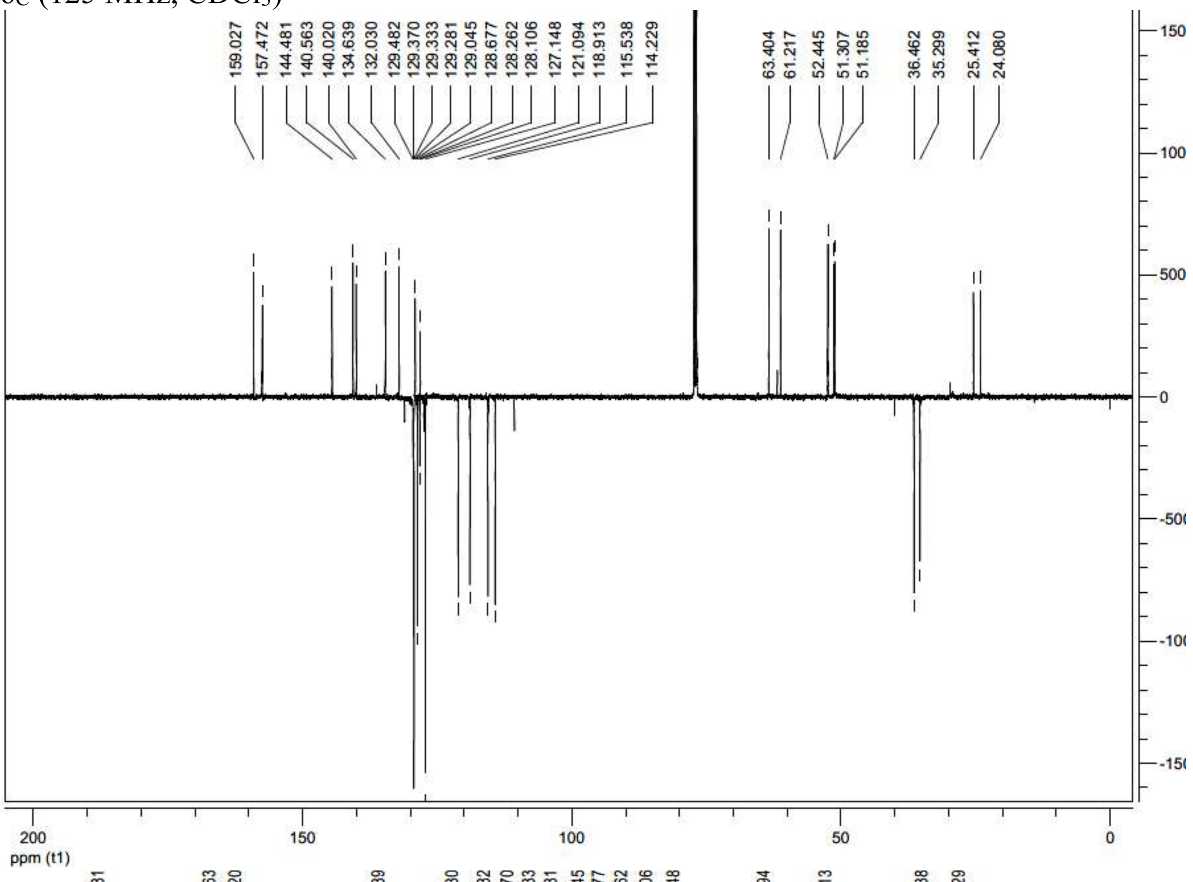

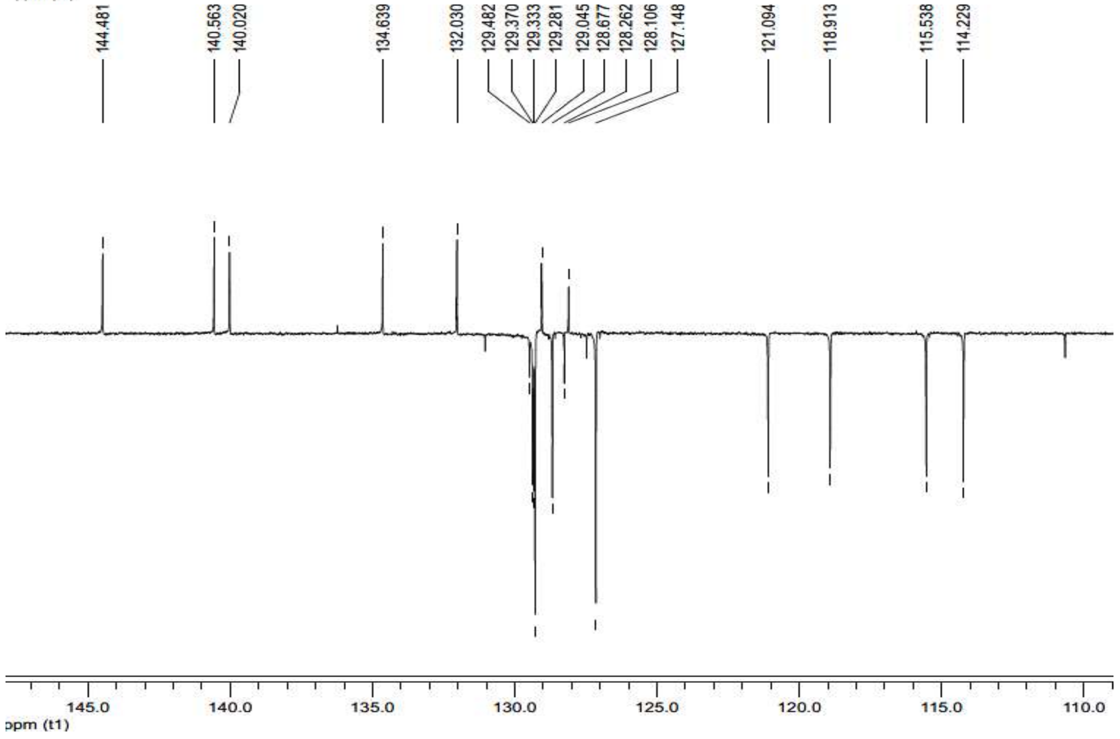

X-ray structure data.

Compound 29. CCDC Deposition Number 2273606.

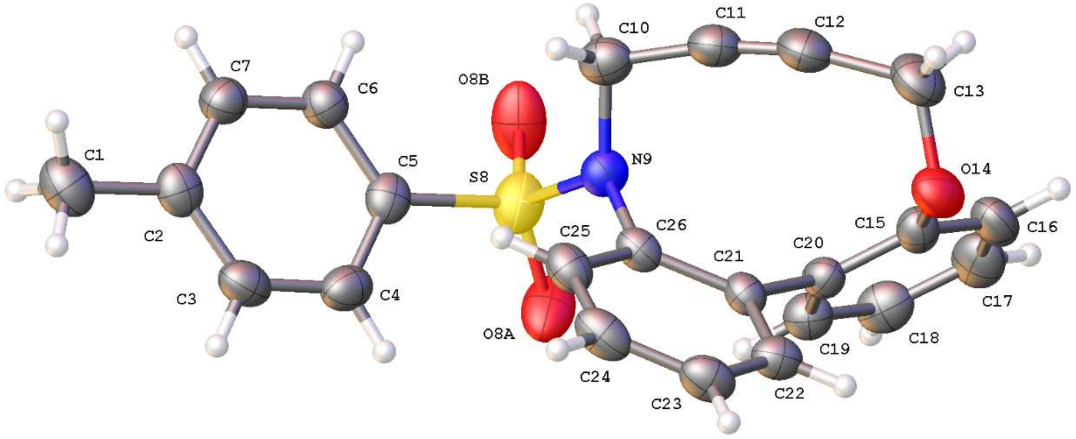

*solid state structure of Compound **29** with atom labelling and thermal ellipsoids drawn at 50% probability level*

Crystal structure determination of Compound 29.
The asymmetric unit contains the strained alkyne, there are 4 in the unit cell.
Angle between a mean plane through atoms C15 C16 C17 C18 C19 C20 to a mean plane through atoms C21 C22 C23 C24 C25 C26 is 64.194 (0.050) degrees.

Experimental
Single crystals of $C_{23}H_{19}NO_3S$ **Compound 29** were grown from **MeOH**. A suitable crystal was selected and mounted on a glass fibre with Fomblin oil and placed on an Xcalibur Gemini diffractometer with a Ruby CCD area detector. The crystal was kept at 150(2) K during data collection. Using Olex2 [1], the structure was solved with the ShelXT [2] structure solution program using Intrinsic Phasing and refined with the ShelXL [3] refinement package using Least Squares minimisation.

Crystal Data for $C_{23}H_{19}NO_3S$ (M =389.45 g/mol): monoclinic, space group $P2_1/c$ (no. 14), a = 15.14881(14) Å, b = 8.79337(7) Å, c = 15.81288(19) Å, β = 112.2723(12)°, V = 1949.27(4) Å^3, Z = 4, T = 150(2) K, μ(CuKα) = 1.669 mm^{-1}, $Dcalc$ = 1.327 g/cm^3, 26230 reflections measured (11.324° ≤ 2Θ ≤ 179.506°), 4102 unique (R_{int} = 0.0497, R_{sigma} = 0.0325) which were used in all calculations. The final R_1 was 0.0469 (I > 2σ(I)) and wR_2 was 0.1333 (all data).

**Table 1 Crystal data and structure refinement for Compound 29.**	
Identification code	sf2
Empirical formula	$C_{23}H_{19}NO_3S$
Formula weight	389.45
Temperature/K	150(2)
Crystal system	Monoclinic
Space group	$P2_1/c$
a/Å	15.14881(14)
b/Å	8.79337(7)

c/Å	15.81288(19)
α/°	90
β/°	112.2723(12)
γ/°	90
Volume/Å^3	1949.27(4)
Z	4
ρ_{calc}g/cm^3	1.327
μ/mm^{-1}	1.669
F(000)	816.0
Crystal size/mm^3	0.12 × 0.12 × 0.06 colourless block
Radiation	CuKα (λ = 1.54184)
2Θ range for data collection/°	11.324 to 179.506
Index ranges	-19 ≤ h ≤ 19, -11 ≤ k ≤ 10, -19 ≤ l ≤ 14
Reflections collected	26230
Independent reflections	4102 [R_{int} = 0.0497, R_{sigma} = 0.0325]
Data/restraints/parameters	4102/0/254
Goodness-of-fit on F^2	1.056
Final R indexes [I>=2σ (I)]	R_1 = 0.0469, wR_2 = 0.1271
Final R indexes [all data]	R_1 = 0.0521, wR_2 = 0.1333
Largest diff. peak/hole / e Å^{-3}	0.73/-0.44

1. Dolomanov, O.V., Bourhis, L.J., Gildea, R.J, Howard, J.A.K. & Puschmann, H. (2009), J. Appl. Cryst. 42, 339-341.
2. Sheldrick, G.M. (2015). Acta Cryst. A71, 3-8.
3. Sheldrick, G.M. (2015). Acta Cryst. C71, 3-8.

Compound 49. CCDC Deposition Number 2273607.

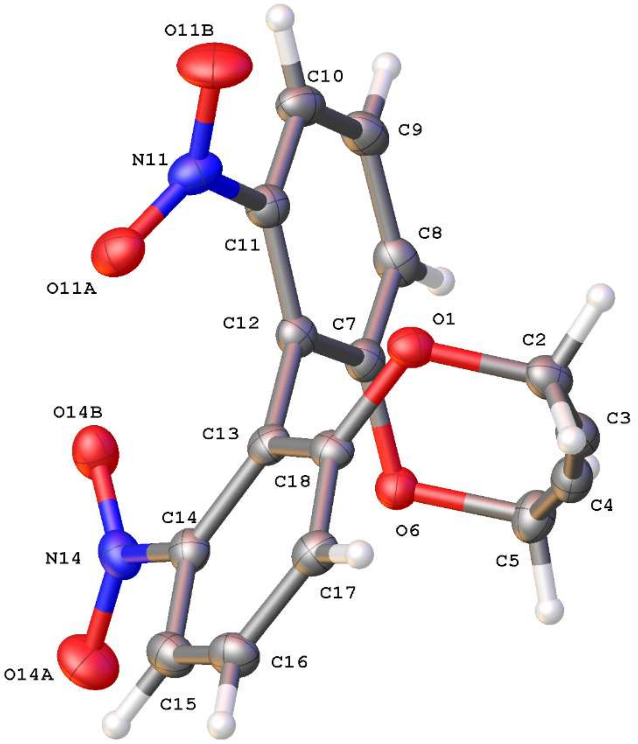

*Solid state structure of Compound **49** with atom labelling and thermal ellipsoids drawn at 50% probability level.*
Below – looking down the biphenyl bond to highlight the dihedral angle and the alkyne geometry.

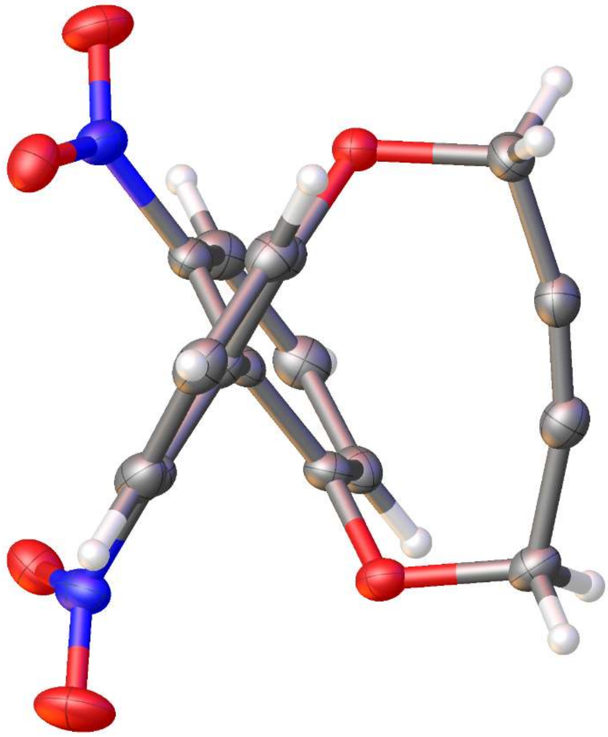

The asymmetric unit contains the strained alkyne macrocycle, there are eight molecules in the unit cell. The dihedral angle between the two phenyl rings was measured in two separate ways:

i) As the angle between mean planes through the two aromatic rings. Atoms used to define the mean planes through either aromatic ring was C7 C8 C9 C10 C11 C12 and C13 C14 C15 C16 C17 C18. Angle between mean planes 64.677 (0.046) degrees.
ii) As the dihedral angle using the atoms C11 – C12 – C13 – C18 is -64.55 (0.24) degrees. Angle between the two oxygens in the ring is O1 to O6 is 4.2570 (0.0017) Angstroms.

Experimental
Single crystals of $C_{16}H_{10}N_2O_6$ Compound **49** were grown from MeOH. A suitable crystal was selected and mounted on a Mitegen head with Fomblin oil and placed on a Rigaku Oxford Diffraction Synergy-S diffractometer with a dual source equipped with a Hybrid pixel array detector. The crystal was kept at 100(2) K during data collection. Using Olex2 [1], the structure was solved with the SHELXT [2] structure solution program using Intrinsic Phasing and refined with the SHELXL [3] refinement package using Least Squares minimisation.

Crystal Data for $C_{16}H_{10}N_2O_6$ (M =326.26 g/mol): orthorhombic, space group Pbca (no. 61), a = 13.2109(2) Å, b = 7.63020(10) Å, c = 27.8997(3) Å, V = 2812.34(6) Å^3, Z = 8, T = 100(2) K, μ(Cu Kα) = 1.028 mm^{-1}, $Dcalc$ = 1.541 g/cm^3, 79612 reflections measured (6.336° ≤ 2Θ ≤ 173.698°), 3102 unique (R_{int} = 0.0631, R_{sigma} = 0.0179) which were used in all calculations. The final R_1 was 0.0461 (I > 2σ(I)) and wR_2 was 0.1432 (all data).

**Table 1 Crystal data and structure refinement for compound 49**	
Identification code	**Sf7**
Empirical formula	$C_{16}H_{10}N_2O_6$
Formula weight	326.26
Temperature/K	100(2)
Crystal system	orthorhombic
Space group	Pbca
a/Å	13.2109(2)
b/Å	7.63020(10)
c/Å	27.8997(3)
α/°	90
β/°	90
γ/°	90
Volume/Å^3	2812.34(6)
Z	8
ρ_{calc}mg/mm^3	1.541
μ/mm^{-1}	1.028
F(000)	1344.0
Crystal size/mm^3	0.2 × 0.1 × 0.01 colourless block
2Θ range for data collection	6.336 to 173.698°
Index ranges	-16 ≤ h ≤ 16, -9 ≤ k ≤ 9, -35 ≤ l ≤ 35
Reflections collected	79612
Independent reflections	3102[R(int) = 0.0631]
Data/restraints/parameters	3102/0/217

Goodness-of-fit on F^2	1.177
Final R indexes [I>=2σ (I)]	R_1 = 0.0461, wR_2 = 0.1262
Final R indexes [all data]	R_1 = 0.0523, wR_2 = 0.1432
Largest diff. peak/hole / e Å^{-3}	0.26/-0.34

1. Dolomanov, O.V., Bourhis, L.J., Gildea, R.J, Howard, J.A.K. & Puschmann, H. (2009), J. Appl. Cryst. 42, 339-341.

2. Sheldrick, G.M. (2015). Acta Cryst. A71, 3-8.

3. Sheldrick, G.M. (2015). Acta Cryst. C71, 3-8.

Compound 54. CCDC Deposition Number 2273608.

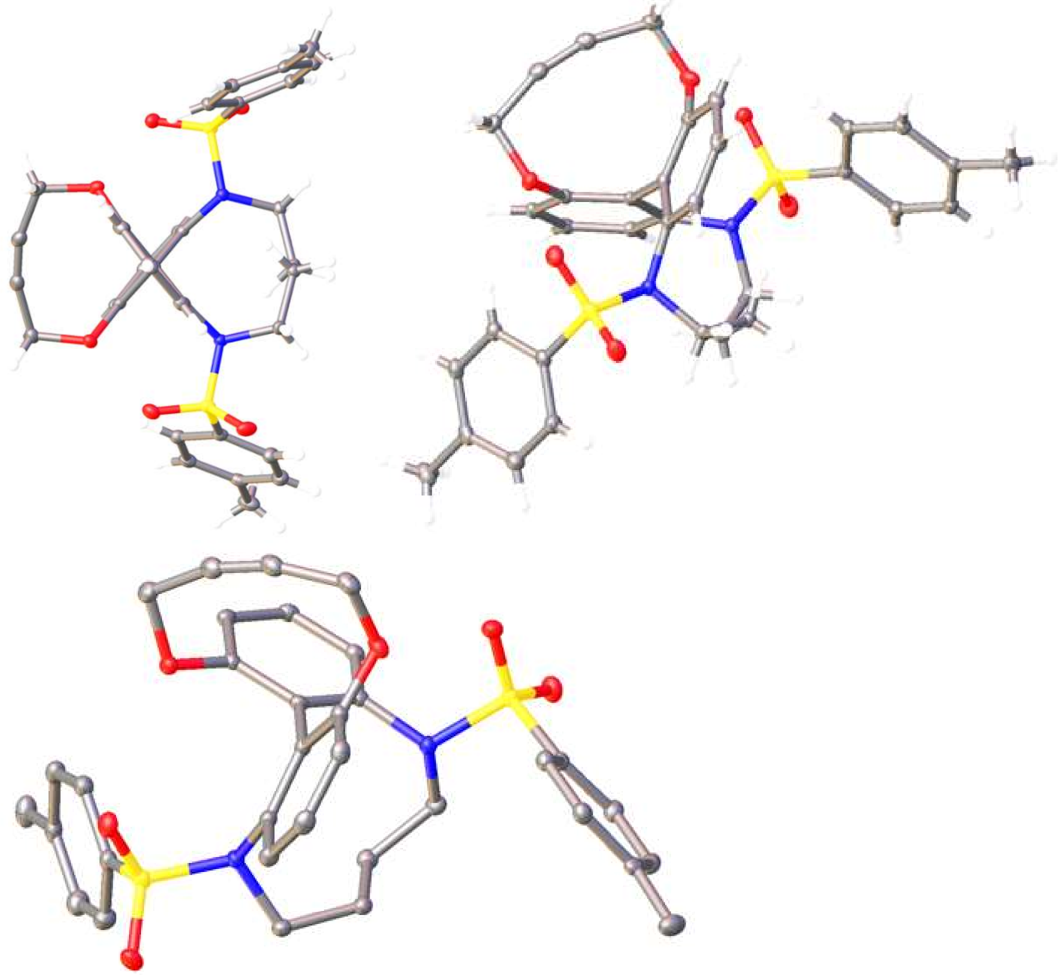

*Views of compound **54** to illustrate extend of alkyne bending, position of C4 bridging chain and the tosyl groups.*

Crystal structure determination of Compound 54.
There are two molecules in the asymmetric unit, like one of each stereoisomer around the biaryl bond. There are very small variations in bond angles between the two molecules as well as the stereo-inversion. There are four molecules in the unit cell related by an inversion centre.

The bond angles in the alkyne:

162.82 (0.16) C127 - C128 - C129
163.78 (0.16) C128 - C129 - C130
163.15 (0.16) C227 - C228 - C229
164.56 (0.16) C228 - C229 - C230

Using mean planes through the relevant aromatic rings to measure biaryl angle. This is described as atoms used to define the mean plane and the angle between these mean planes. Angle between mean plane through C114 C115 C116 C117 C118 C119 to mean plane through C120 C121 C122 C123 C124 C125 is (with approximate esd) = 79.644 (0.044). Angle between mean plane through C214 C215 C216 C217 C218 C219 to mean plane through C220 C221 C222 C223 C224 C225 is (with approximate

esd) = 78.562 (0.044). The small difference are likely crystal packing effects as the mirror image of these two enantiomers is also in the unit cell.

Experimental

Single crystals of $C_{34}H_{32}N_2O_6S_2$ **Compound 54** were grown from methanol. A suitable crystal was selected and mounted on a glass fibre with Fomblin oil and placed on a Rigaku Oxford Diffraction SuperNova diffractometer with a duel source (Cu at zero) equipped with an AtlasS2 CCD area detector. The crystal was kept at 150(2) K during data collection. Using Olex2 [1], the structure was solved with the SHELXT [2] structure solution program using Intrinsic Phasing and refined with the SHELXL [3]

Crystal Data for $C_{34}H_{32}N_2O_6S_2$ (M =628.73 g/mol): triclinic, space group P-1 (no. 2), a = 10.96210(10) Å, b = 17.2319(2) Å, c = 17.6310(2) Å, α = 90.8900(10)°, β = 108.0600(10)°, γ = 105.6680(10)°, V = 3030.74(6) Å^3, Z = 4, T = 150(2) K, μ(Cu Kα) = 2.004 mm^{-1},$Dcalc$ = 1.378 g/cm^3, 146215 reflections measured (5.304° ≤ 2Θ ≤ 147.292°), 12178 unique (R_{int} = 0.0632, R_{sigma} = 0.0203) which were used in all calculations. The final R_1 was 0.0333 (I > 2σ(I)) and wR_2 was 0.0921 (all data).

**Table 1 Crystal data and structure refinement for Compound 54**	
Identification code	MW14
Empirical formula	$C_{34}H_{32}N_2O_6S_2$
Formula weight	628.73
Temperature/K	150(2)
Crystal system	triclinic
Space group	P-1
a/Å	10.96210(10)
b/Å	17.2319(2)
c/Å	17.6310(2)
α/°	90.8900(10)
β/°	108.0600(10)
γ/°	105.6680(10)
Volume/Å^3	3030.74(6)
Z	4
ρ_{calc}mg/mm^3	1.378
μ/mm^{-1}	2.004
F(000)	1320.0
Crystal size/mm^3	0.12 × 0.12 × 0.06 colourless block
2Θ range for data collection	5.304 to 147.292°
Index ranges	-13 ≤ h ≤ 13, -21 ≤ k ≤ 21, -21 ≤ l ≤ 21
Reflections collected	146215
Independent reflections	12178[R(int) = 0.0632]
Data/restraints/parameters	12178/0/797
Goodness-of-fit on F^2	1.020
Final R indexes [I>=2σ (I)]	R_1 = 0.0333, wR_2 = 0.0894
Final R indexes [all data]	R_1 = 0.0364, wR_2 = 0.0921

Largest diff. peak/hole / e Å^{-3}	0.36/-0.37

1. Dolomanov, O.V., Bourhis, L.J., Gildea, R.J, Howard, J.A.K. & Puschmann, H. (2009), J. Appl. Cryst. 42, 339-341.
2. Sheldrick, G.M. (2015). Acta Cryst. A71, 3-8.
3. Sheldrick, G.M. (2015). Acta Cryst. C71, 3-8.

Compound 56. CCDC Deposition Number 2273609

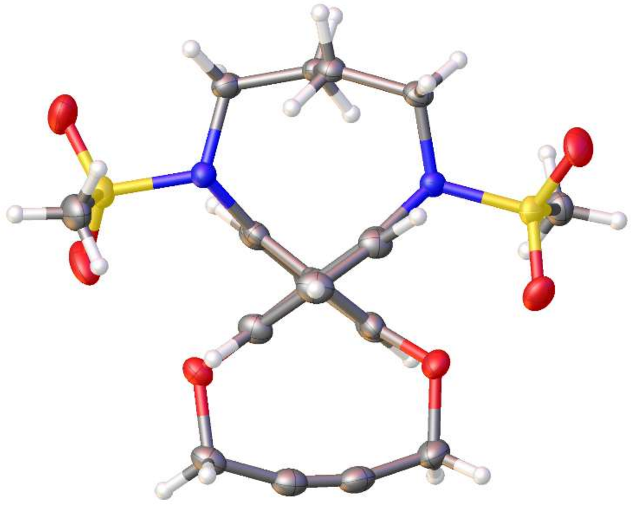

*Solid state structure of **56** looking down the biphenyl axis. Thermal ellipsoids are drawn at 50% probability level*

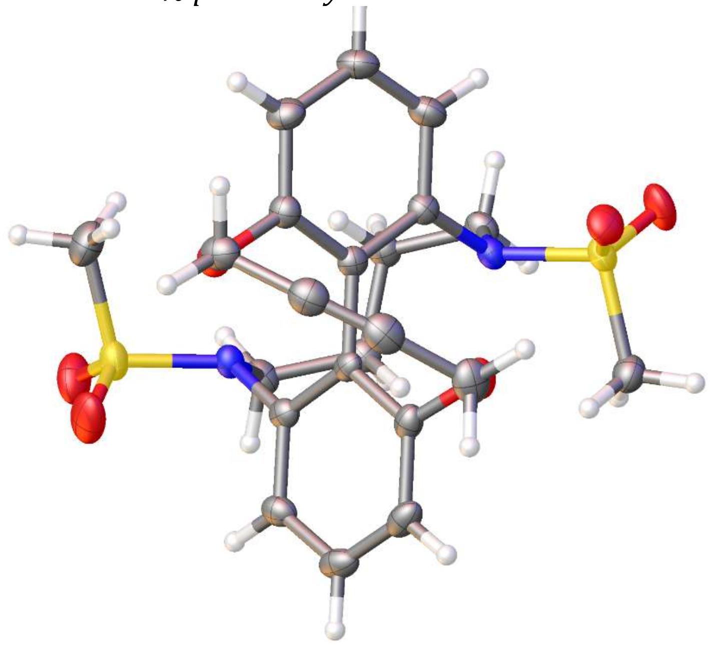

*A view of **56** looking roughly straight down the strained alkyne across the biphenyl bond to the butyl chain below*

Crystal structure determination of 56.

The asymmetric unit contains the alkyne, there are four molecules in the unit cell. Both handedness of rotomers around the biphenyl bond are in the cell related by centres. The angle between the two phenyl rings is defined by the angle between mean planes through the two rings. This is described as the atoms used to define the rings and the angle between the mean planes through them. The angle between mean plane through ring C9 C10 C11 C12 C13 C14 and ring C7 C8 C21 C22 C23 C24 is 74.543 (0.050) degrees.

Experimental

Single crystals of $C_{22}H_{24}N_2O_6S_2$ **56** were grown from DCM. A suitable crystal was selected and mounted on a Mitegen head with Fomblin oil and placed on a Rigaku Oxford Diffraction Synergy-S diffractometer with a dual source equipped with a HyPix-Arc 100 pixel hybrid photon counting X-ray detector. The crystal was kept at 100(2) K during data collection. Using Olex2 [1], the structure was solved with the SHELXS [2] structure solution program using Direct Methods and refined with the SHELXL [3] refinement package using Least Squares minimisation.

Crystal Data for $C_{22}H_{24}N_2O_6S_2$ (M =476.55 g/mol): monoclinic, space group $P2_1/c$ (no. 14), a = 13.6603(3) Å, b = 11.3987(2) Å, c = 14.7403(3) Å, β = 106.075(2)°, V = 2205.45(7) Å^3, Z = 4, T = 100(2) K, μ(Cu Kα) = 2.558 mm^{-1}, $Dcalc$ = 1.435 g/cm^3, 64880 reflections measured (9.96° ≤ 2Θ ≤ 156.876°), 4739 unique (R_{int} = 0.0896, R_{sigma} = 0.0335) which were used in all calculations. The final R_1 was 0.0407 (I > 2σ(I)) and wR_2 was 0.1049 (all data).

**Table 1 Crystal data and structure refinement for 56.**	
Identification code	mw50
Empirical formula	$C_{22}H_{24}N_2O_6S_2$
Formula weight	476.55
Temperature/K	100(2)
Crystal system	monoclinic
Space group	$P2_1/c$
a/Å	13.6603(3)
b/Å	11.3987(2)
c/Å	14.7403(3)
α/°	90
β/°	106.075(2)
γ/°	90
Volume/Å^3	2205.45(7)
Z	4
ρ_{calc}g/cm^3	1.435
μ/mm^{-1}	2.558
F(000)	1000.0
Crystal size/mm^3	0.12 × 0.06 × 0.02
Radiation	Cu Kα (λ = 1.54184)
2Θ range for data collection/°	9.96 to 156.876
Index ranges	$-17 \le h \le 17, -14 \le k \le 14, -18 \le l \le 18$
Reflections collected	64880
Independent reflections	4739 [R_{int} = 0.0896, R_{sigma} = 0.0335]
Data/restraints/parameters	4739/0/291
Goodness-of-fit on F^2	1.075
Final R indexes [I>=2σ (I)]	R_1 = 0.0407, wR_2 = 0.1012
Final R indexes [all data]	R_1 = 0.0471, wR_2 = 0.1049
Largest diff. peak/hole / e Å^{-3}	0.28/-0.48

1. Dolomanov, O.V., Bourhis, L.J., Gildea, R.J, Howard, J.A.K. & Puschmann, H. (2009), J. Appl. Cryst. 42, 339-341.
2. Sheldrick, G.M. (2008). Acta Cryst. A64, 112-122.
3. Sheldrick, G.M. (2015). Acta Cryst. C71, 3-8.

Studies of binding to azide-functionalised proteins.

Two runs of tests of an azide-labelled protein with strained alkymes were undertaken. The azide-labelled protein used for these tests was glutathione S-transferase (GST) containing an azidophenylalanine residue at position 52, allowing it to undergo click cycloaddition reaction with strained alkynes. The MW of the wild type is ca 29 kDa.

Sequence details:>GST (Highlighted position of azide incorporation is ***F***$^{52\text{-}AzF)}$
MSPILGYWKI KGLVQPTRLL LEYLEEKYEE HLYERDEGDK WRNKKFELGL
E**F**PNLPYYID GDVKLTQSMA IIRYIADKHN MLGGCPKERA EISMLEGAVL
DIRYGVSRIA YSKDFETLKV DFLSKLPEML KMFEDRLCHK TYLNGDHVTH
PDFMLYDALD VVLYMDPMCL DAFPKLVCFK KRIEAIPQID KYLKSSKYIA
WPLQGWQATF GGGDHPPKSD LVPRGSPGIH RD

Materials and methods for the first set of attempted click reactions.

Expression and induction of GST-WT and GST-F52TAG in *E. coli*.
E. coli BL21-AI competent cells were used to produce GST with 52nd amino acid position replaced by TAG codon while *E. coli* BL21(DE3) was used for GST wild type expression. *E. coli* BL21(DE3) was transformed with plasmid pGex-5X1-WT expressing GST as the method described above for *E. coli* DH5α. The transformed cells were amplified overnight in 15 to 20 mL of LB broth containing ampicillin at 1 µg/mL at 36 ˚C with shaking at 200 rpm. The next day, the culture was diluted to an OD_{600} at 0.2 in 100 mL of LB broth containing ampicillin. GST-WT was induced when the culture reached an OD_{600} of 0.6 to 0.8 by the addition of IPTG to a final concentration of 0.5 mM, followed by four hours of incubation at 30 ˚C with shaking at 200 rpm. Cells were spun at 4,000 rpm for 20 minutes at 4 ˚C and washed with ice cold PBS containing phenylmethylsulfonyl fluoride (PMSF). The cells were spun down again and the pellets were kept at -20◦C for storage.

GST-F52TAG was expressed in *E. coli*-BL21-AI. The competent cells were co-transformed with plasmids pDule2-pCNF to express orthogonal aaRS/tRNA pair under the control of AraC and with plasmid pGex-5X1-F52TAG expressing GST mutant. The cells were electroporated with 2.5 kV for a few seconds and recovered in 300 µL of SOC medium. The cells were incubated at 36 ˚C for two hours with shaking at 850-900 rpm. Subsequently, the cells were added into 100 mL of ZY non-inducing media for overnight incubation at 36 ˚C with shaking at 300 rpm. The next day, 500 µL of the culture was added into 100 mL of ZY autoinducing media with a final concentration of 1 mM IPTG, 1 mM AzF, 0.05% arabinose and 1 µg/mL of spectinomycin. The ZY non-inducing and autoinducing medium were made as described by Peeler et al. (*Peeler, J. C., & Mehl, R. A. (n.d.). Site-specific incorporation of unnatural amino acids as probes for protein conformational changes. In L. Pollegioni & S. Servi (Eds.), Unnatural amino acids: Methods and protocols (pp. 125–134). Totowa, NJ: Humana Press. doi: 10.1007/978-1-61779-331-8 8*). The cells were collected as described above for the induction of GST-WT.

GST Pull Down Assay.
For cell lysis, the pellets obtained from the protein induction were mixed with resuspension buffer with a final concentration of 2.5 mM Benzamidine, 1.0 mM PMSF, 1 mM EDTA, 1mM DTT, 1x protease inhibitor (50x protease inhibitor: one tablet of cOmplete™, EDTA-free Protease Inhibitor Cocktail from COEDTAF-RO ROCHE

dissolved in 1 mL of ddH_2O). After the addition of lysozyme to a final concentration of 1mg/mL, the suspension was kept on ice for 30 minutes. All the centrifugation was performed at 4 °C and the incubation was carried out on ice unless otherwise stated. Subsequently, the cells were sonicated (QSonica Sonicator Q125) on ice with 60% amplitude, pulse 15 seconds on and 5 seconds off, for 1 minute. Sonication was repeated for 6 to 10 times until about 75% of the cells were lysed. The lysate was chilled on ice for another 30 minutes with 1.0% Triton X100, followed by centrifugation at 15, 000 rpm for 20 minutes. The supernatant was retained as whole cell extract (WCE) and the pellet (PEL) was resuspended with the resuspension buffer.

For the pull-down of GST, sepharose beads were pre-washed with PBS containing 1% Triton X100 and spun down at 2, 000 rpm for 2 minutes for 4 to 6 times. WCE was mixed with the beads and rolled at 50 rpm for two hours. The mixture was spun down at 1, 500 rpm for 3 minutes after incubation. The supernatant (SUP) was sampled to check the GST-sepharose binding efficiency. The beads bound with GST were washed with PBS containing 0.1% Triton X100 for 6 to 8 times to remove the non-specific proteins. The GST was eluted for a few times with elution buffer with 50 mM Tris pH 8.0 and 10 mM glutathione as the final concentration. The centrifugation speed for the beads after bound with GST should not exceed 1,500 rpm. All the eluents (E1-E5) were collected and sampled to be resolved by SDS-PAGE together with WCE, PEL and SUP. After SDS-PAGE was performed, the gel was stained with Coomassie Brilliant Blue (CBB) to determine the amount of GST in the respective fractions.

A minimum of 30 ncAAs were successfully incorporated with high yield and fidelity in response to triplet or quadruplet codons in 2006 (*Wang, L., Xie, J., & Schultz, P. G. (2006). Expanding the Genetic Code. Annual Review of Biophysics and Biomolecular Structure. doi: 10.1146/annurev.biophys.35.101105.121507*). The Amber stop codon was chosen to encode ncAAs due to its low occurring frequency in *E. coli* of 7% (*Nakamura, Y., Gojobori, T., & Ikemura, T. (2000). Codon usage tabulated from international DNA sequence databases: status for the year 2000 [In Process Citation]. Nucleic Acids Res. doi:10.1093/nar/28.1.292*). It is a nonsense codon which does not specify any of the natural amino acids in most biological systems. A gel analysis showed that amber suppression has successfully rescued the expression of full-length GST-F52TAG expressed in E. coli, and this is illustrated below.

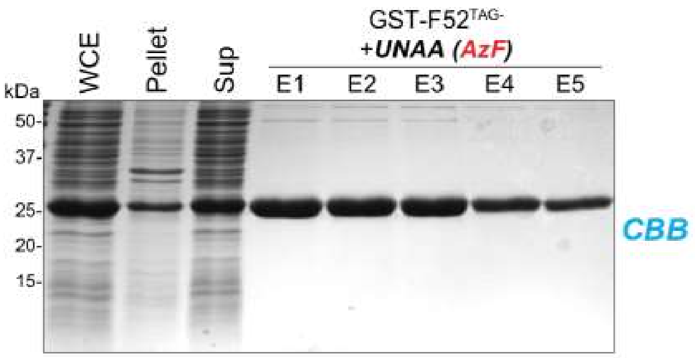

GST-F52TAG expressed in E. coli by amber suppression. WCE: whole cell extract, sup: supernatant obtained after GST bound to sepharose beads, E: eluent.

Click Reaction of strained alkynes with GST-F52TAG.

The cycloaddition between 100 µM of the alkyne probes and 50 µM of GST-F52TAG was used to determine the minimum time and temperature required for the reaction. This would provide information for future applications as some proteins are sensitive to heat, thus, would be ideal to click at lower temperature. The strained alkynes and control molecules (see diagram below) were dissolved in DMSO to make a 5 mM stock concentration. Click reactions were run at 15 µL per reaction with a final concentration of 50 µM GST-F52TAG and 100 µM of respective probes in PBS. The commercial DIBO dyes were obtained from Invitrogen™ Click-IT™ DIBO alkyne conjugated with respective Alexa Fluor™ 488, 555 and 647 for copper free click chemistry. Each of the probes was clicked with GST-52AzF. The experiment was run at 4 °C, 16 °C and room temperature 25 °C with shaking at 500 rpm for 2, 4, 6 and 8 hours respectively. After incubation, the samples were mixed with 5 µL 4x protein solubilisation buffer and boiled at 95 °C for 7 five minutes to be resolved by SDS-PAGE. The bands were visualised by SynGene PXI with respective filter modules.

(A) BoDIPY dye with not strained alkyne (control):

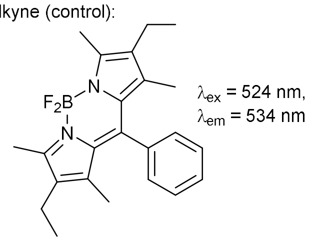

Chemical Formula: $C_{23}H_{27}BF_2N_2$
Exact Mass: 380.22
Molecular Weight: 380.29

(B) BoDIPY dye linked to strained alkyne (previously reported):

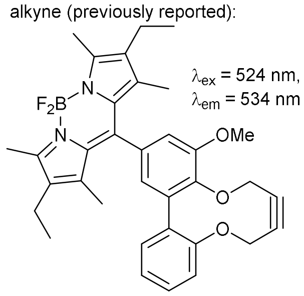

Chemical Formula: $C_{34}H_{35}BF_2N_2O_3$
Exact Mass: 568.27
Molecular Weight: 568.47

(C) Fluorescein amide derivative **24**:

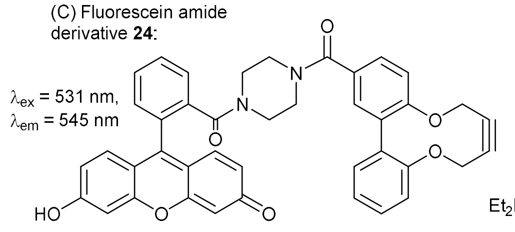

Chemical Formula: $C_{41}H_{30}N_2O_7$
Molecular Weight: 662.70
Exact Mass: 662.21

(D) Rhodamine amide derivative25**)**:

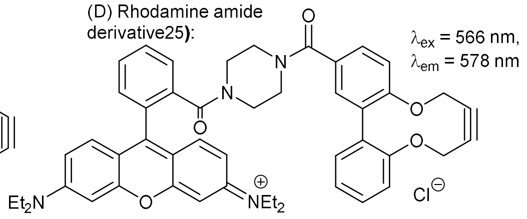

Chemical Formula: $C_{49}H_{49}N_4O_5$
Molecular Weight: 773.95 (cation)
Exact Mass: 773.37

D- Rhodamine with no strained alkyne (control)

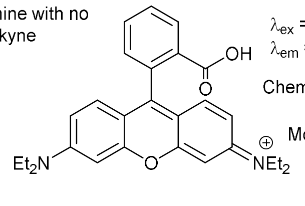

λex = 553 nm,
λem = 627 nm

Chemical Formula: $C_{28}H_{31}N_2O_3$
Exact Mass: 443.23
Molecular Weight: 443.57

Above: Compound (A) contains a BoDIPY group but no strained alkyne and was a control compound for the previously-reported BoDIPY reagent (B). Compounds (C) and (D) are alkynes **24** and **25** respectively. Compound (D-) is a control for compound (D). Three commercial strained alkyne/dyes were also tested as controls for the reactions.

All the commercial fluorescent alkynes clicked with GST-F52TAG readily at room temperature and gave the strongest signals. Of the novel alkynes tested, compound D produced the best signal among the three fluorescent alkynes (B, C and D) when excited by light at wavelength 568 nm. The gels are shown in the Figure below. Control

compound A showed minimal non-specific binding. In addition, weak fluorescence was also observed in DIBO-Alkyne Alexa 488 even at λ_{ex} 568 nm. The fluorescein-containing alkyne C also emitted a signal at λ_{ex} 488 nm. The electrophoresis gels indicated that compound (B), (C) and (D) had bound to the azide-loaded protein, as indicated by the red traces which are visible under 568 nm irradiation. The control compounds, lacking the strained alkynes, do not give red traces in the gel. The red traces in the gel are, however, much weaker than those of the commercial click reagents in which DIBO is the strained alkynes.

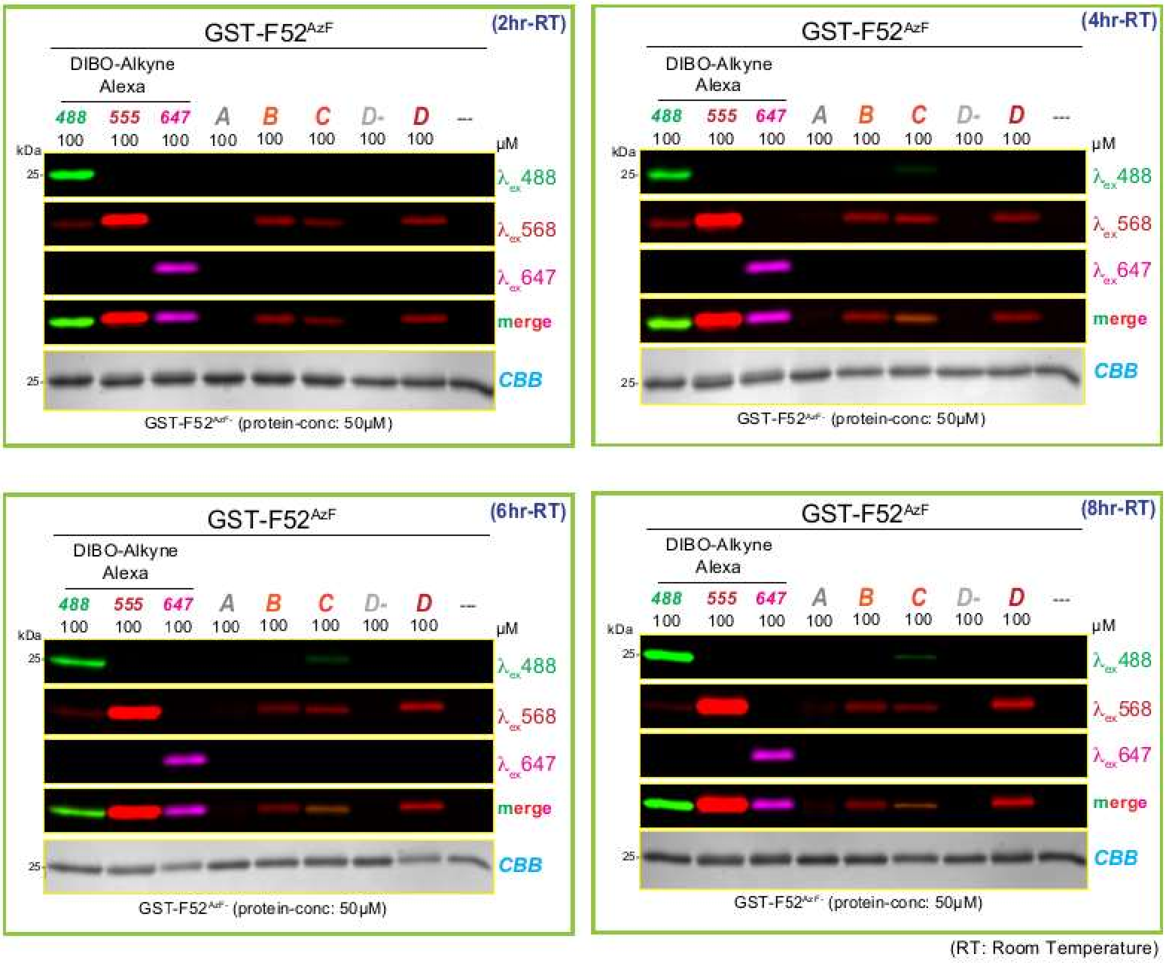

Above. Gel electrophoresis of the click reactions of three commercial strained alkynes with azide-labelled GST at various temperatures and visualised at varying wavelengths (note: Alexa 488; λ_{ex} 488, λ_{em} 494, Alexa 555; λ_{ex} 555, λ_{em} 565, Alexa 647; λ_{ex} 647, λ_{em} 667 nm)..

Analysis by mass spectrometry:
Although the gel results were promising, the subsequent mass spectrometry analysis of a selection of the products only revealed new molecular ions corresponding to the clicked product for compound (C) and not for either (B) or (D) (see below). This indicates that either the extent of click reaction is very low and below the level which can be determined by mass spectrometry or that the alkyne is attached to the protein in the gels through non-covalent interactions. One of the DIBO-Alexafluor standards was confirmed to attach strongly in the mass spectrometry analysis. In several cases, glutathionylation of the protein was observed, which is a known process for this protein

(J. Melchers, N. Dirdjaja, T. Ruppert and R. L. Krauth-Siegel, *J. Biool. Chem.* 2006, **282**, 8678 –8694).

Mass spectrometry procedure:
Instrument: Bruker MaXis II coupled with Dionex 3000RS UHPLC
Column: ACE C4, 300A, 100x2.1mm,
Mobile phases: A: Water with 0.1% Formic acid
B: Acetonitrile with 0.1% Formic acid
Gradient: 0-5mins, 95% A/5% B
5-20mins: 95 %A /5% B to 100% B
20-25mins, 100% B
25-27mins, 100% B to 95% A/5%B
Flow rate: 0.2ml/min
Injection volume: 10uL

1) Azide-functionalised GST protein standard. No glutathionylation observed.

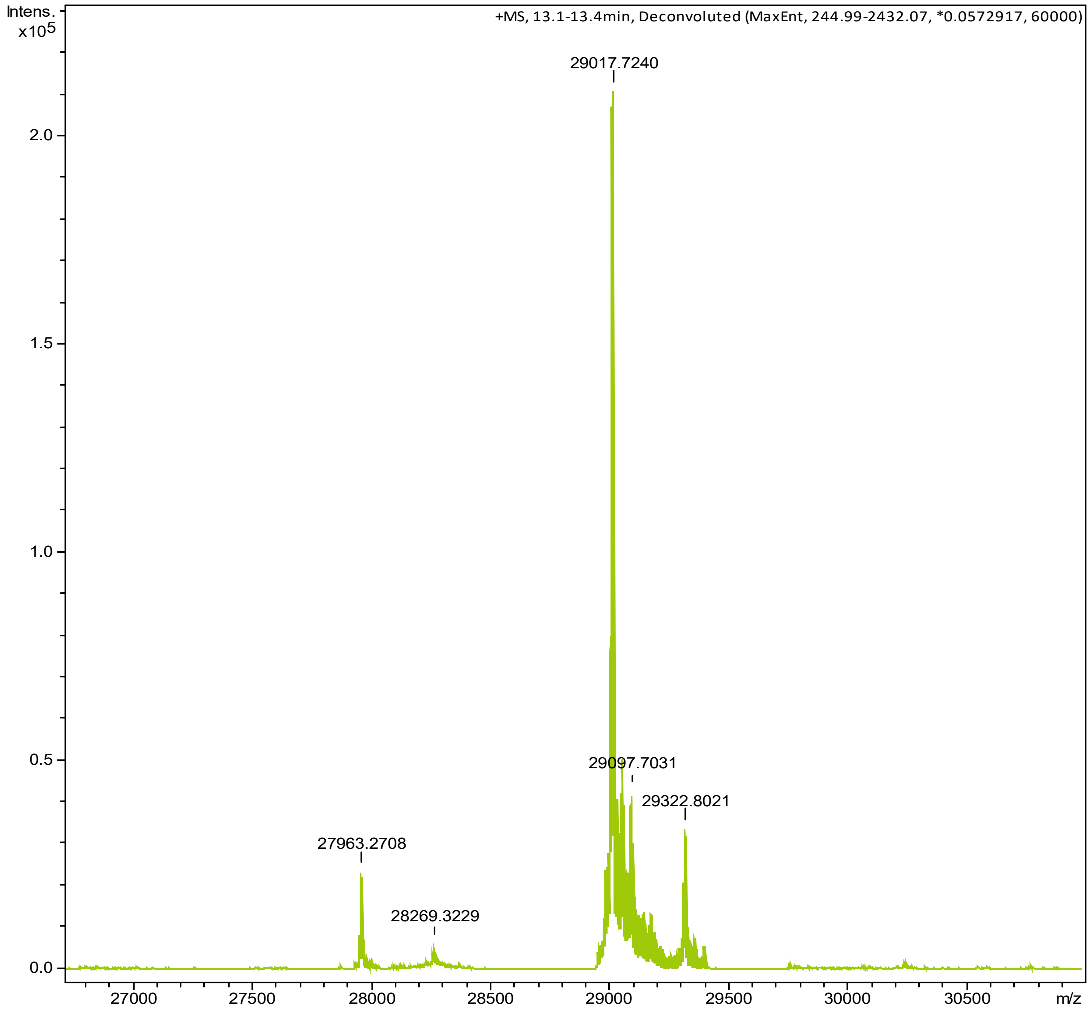

The signal at 29,017 is from the azide-containing protein.

2) GST azide+ addition of a DIBO-Alexa 488 commercial dye (MW = 834 (of the positively charged fluorescent component; **https://www.fishersci.co.uk/shop/products/click-it-alexa-fluor-488-dibo-alkyne-copper-free-click-chemistry-detection-azide/10716241** total MW = 1039.23 Molecular formula = $C_{53}H_{62}N_6O_{12}S_2$ Molecular Formula. This compound adds fully in the click reaction as analysed by mass spectrometry:

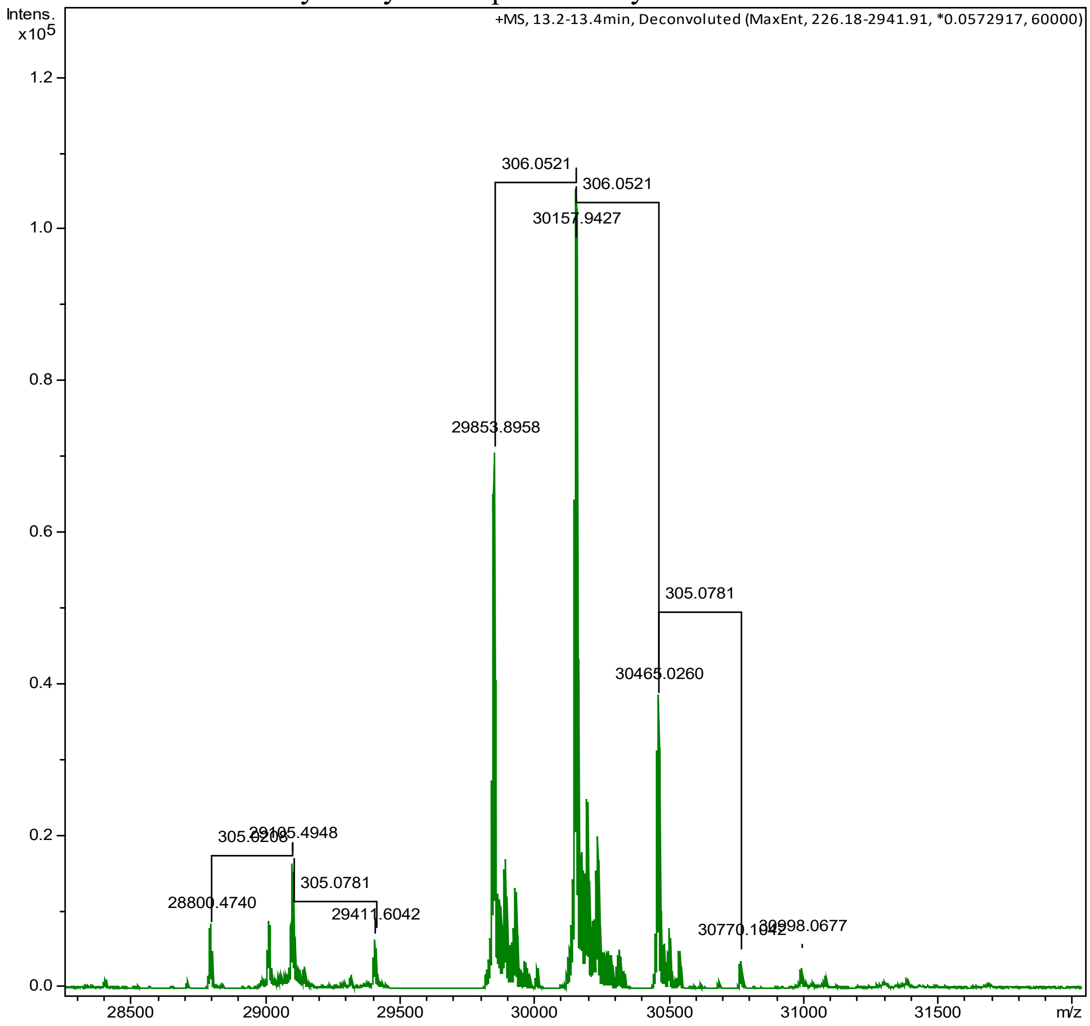

Peak at M/Z 29,853 corresponds to unclicked at 29,017 + 834 = 29,851.
Peak at M/Z 30,157 corresponds to unclicked at 29,332 + 834 = 30,166.
Peak at m/z 30,465 corresponds to unclicked at 29,627 +834 = 30.461.

3) BoDIPY functionalised alkyne B (previously reported in A. Mistry, R. C. Knighton, S. Forshaw, Z. Dualeh, J. S. Parker and M. Wills, *Org. Biomol. Chem.*, 2018, **16**, 8965-8975).

The main visible peaks are of glutathionylation are observed, which adds steps of 306 to the mass, and is a known process for this protein. No click product was detected.

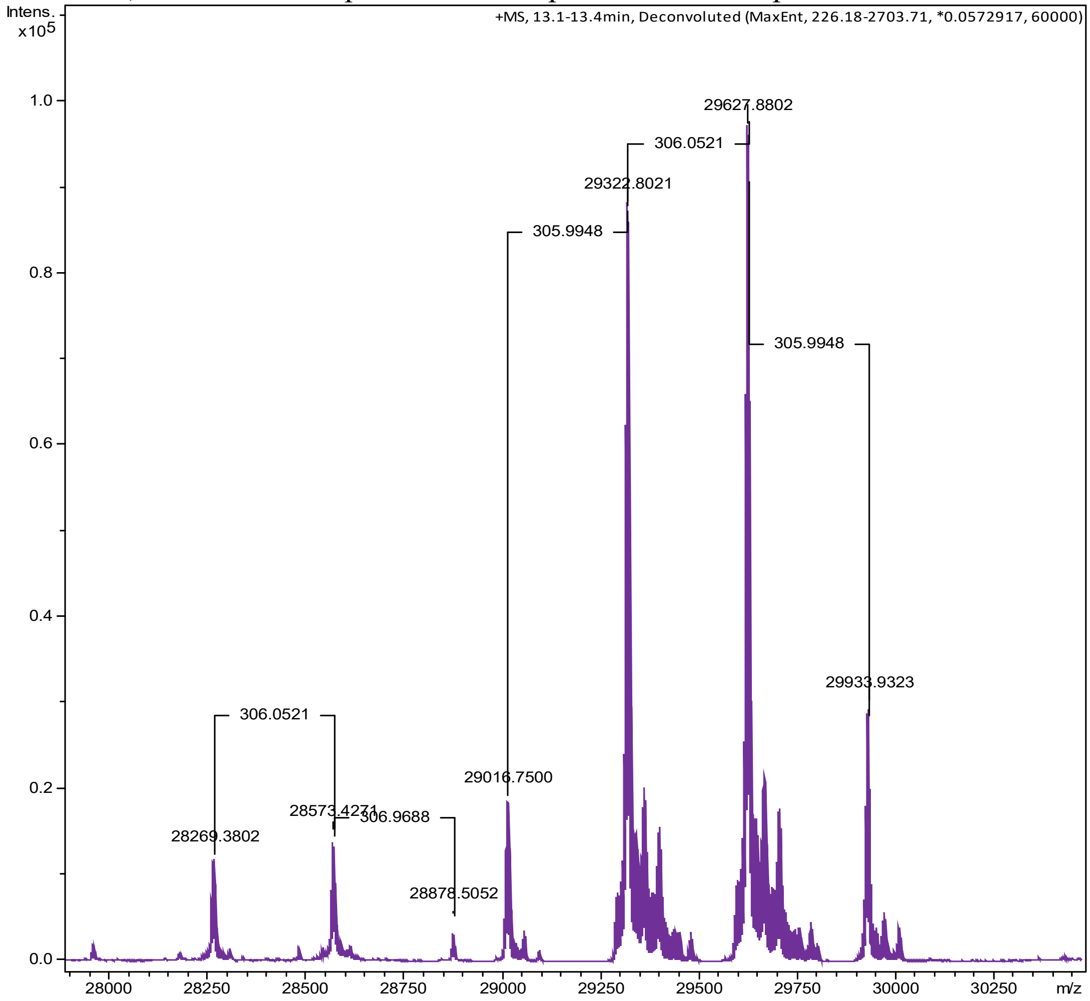

4) GST-azide protein with fluorescein-labelled alkyne C (compound 24).

In this structure, products of glutathionylation are observed.are observed, as are products from addition of the alkyne, although at low intensity:

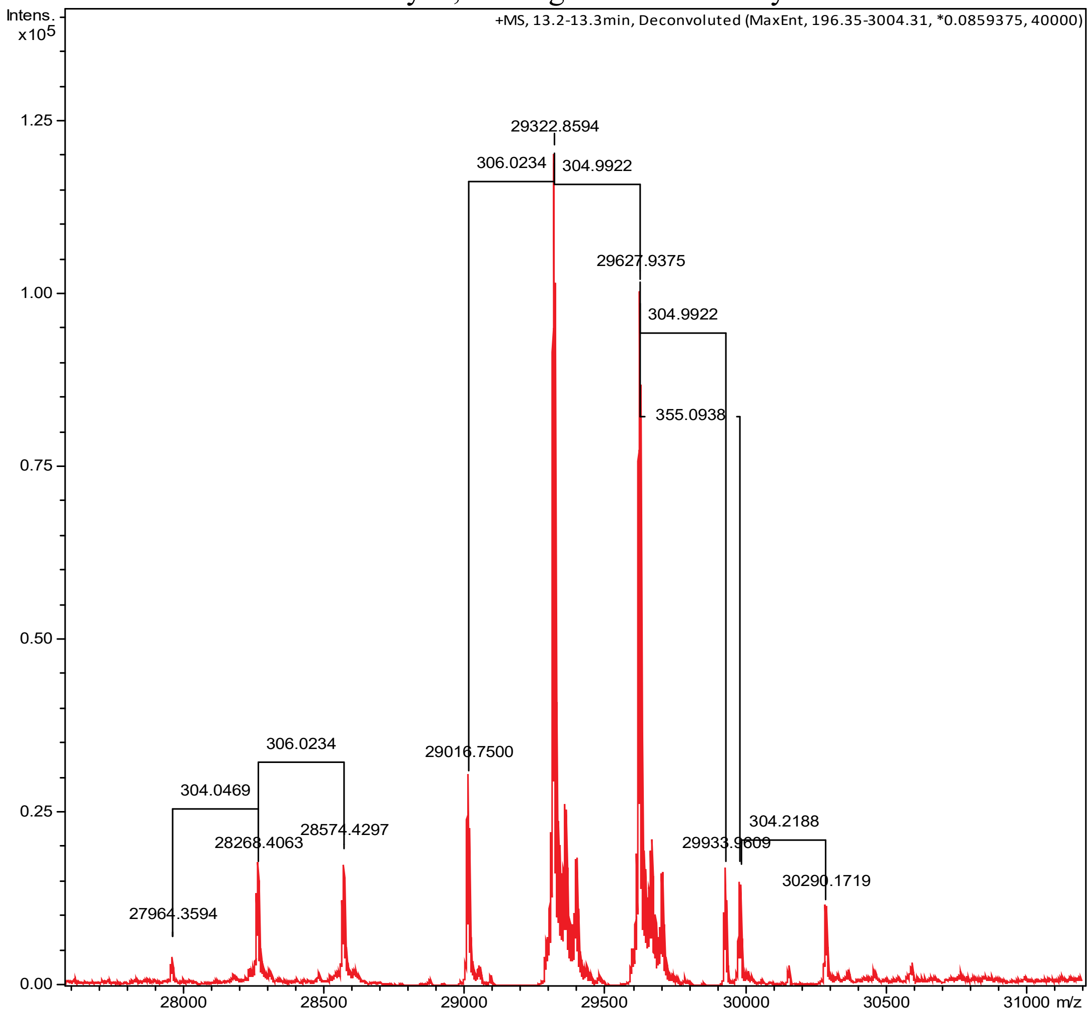

The major peaks correspond to additions of 306 to the protein due to glutathionylation. However, an addition of 662 can be seen to the 29,332 peak at 29,994, and to the 29,628 peak at 30,290, corresponding to addition of compound **C/24**.

5) GST-azide+ Rhodamine alkyne D (25).

No addition of alkyne is observed. Only peaks from glutathionylation are observed.

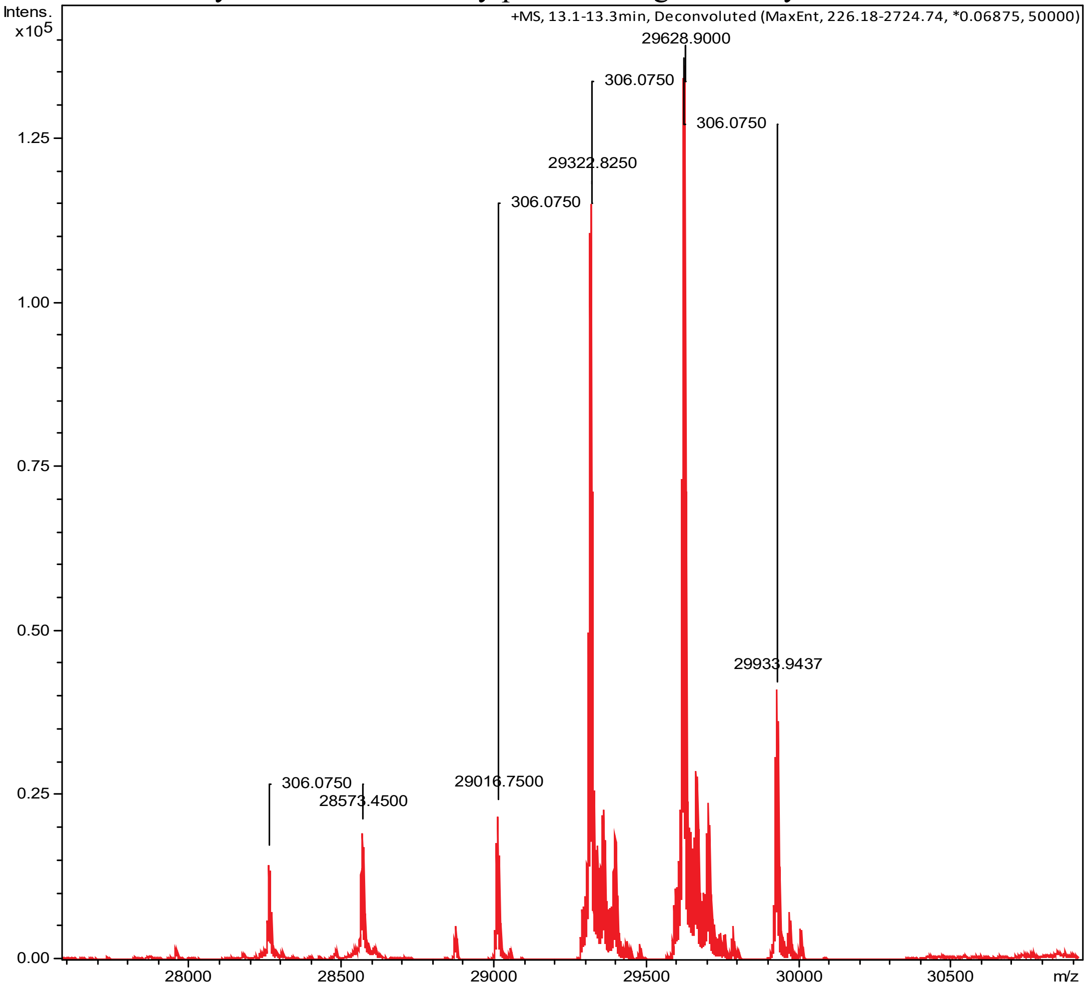

Comparison of mass spectrometric analyses of all samples in first set of tests:

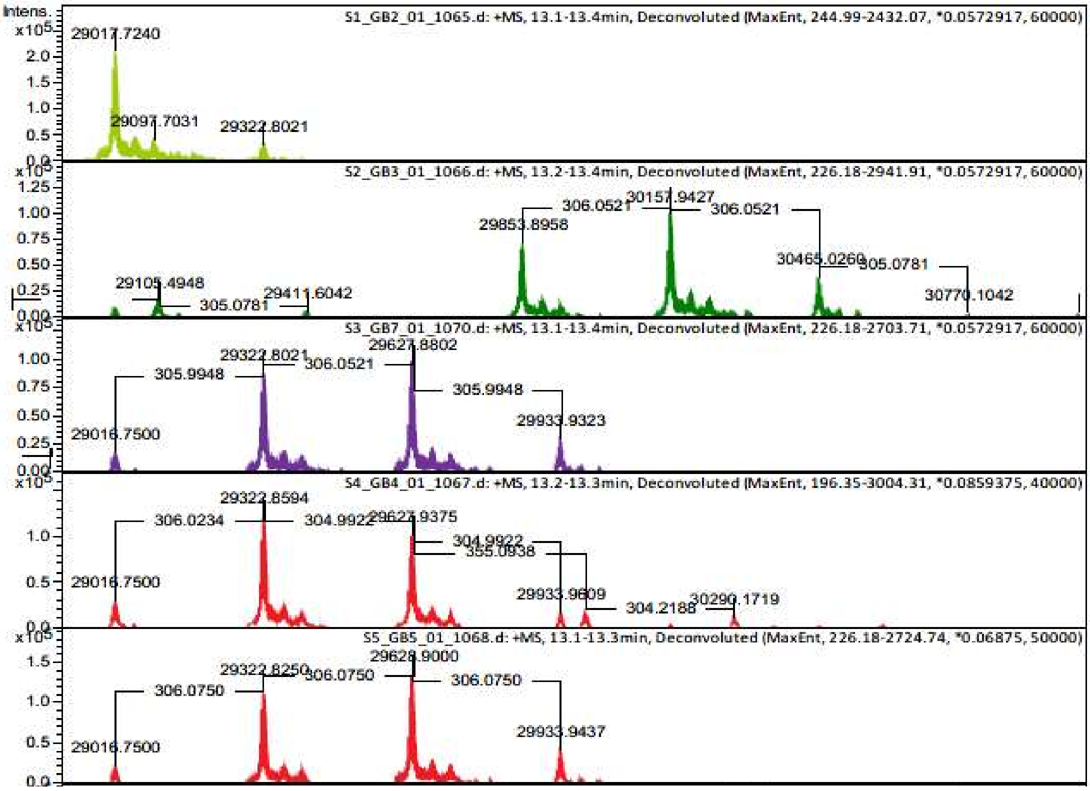

Above: The reference sample of GST-azide alone (row 1) is not glutathionylated. In the clicked samples (rows 2-5) the protein is significantly glutathionylated (steps of +306 from 29.017. The In row 2, the successful addition of a DIBO-AlexaFluor 488 dye (MW 834) is clear from the mass increases observed. There is no evidence of a successful click reaction in rows 3 (Biodipy **B**) or 5 (Rhodamine functionalised **D/25**). The peaks in row 4 at 30,290 and 29,994 correspond to an addition of ca 662, (corresponding to the MW of the fluorescein compound **C/24**) to the 29,628 and 29,332 peaks respectively, indicating a low but distinctive level of click reaction.

<u>Second series of click reactions.</u>

<u>Method – Click Reactions for MS Detection</u>

Genetic Code Expanded GST Protein Purification

An amber stop codon was introduced via site-directed mutagenesis into the F52 position of Glutathione S-Transferase (GST) tagged with a thrombin site-linked 6xHis-tag in a pET vector (pET His6 GST TEV LIC) (Addgene, #29707). For synthetase/tRNA expression, pDule-pCNF plasmid (Addgene, #85494) was used. These constructs were chemically transformed into BL21(DE3) *E.coli*. 5ml cultures of transformed cells were grown overnight, and then diluted to 0.1 OD_{600} before growth to 0.3 OD_{600}. 100mM H-4-Azido-Phe-OH/azidophenylalanine (AzF) (Bachem, 4096192) in 0.2M HCl was added to each culture to a final concentration of 1mM AzF. All procedures were done in the dark where possible from this point. Cultures were grown to 0.5 OD_{600} and 0.5mM IPTG was added. After a further 3-4 hours incubation, cultures were centrifuged at 4,000 rpm for 10 minutes. Pellets were stored at -80ºC until protein purification.

For purification, 5X Protein Prep Buffer (500mM Tris-HCl, 2.5M NaCl) and 1M imidazole were prepared, adjusted to pH 8.5 and autoclaved. Pellets were each dissolved in 30ml Lysis Buffer (1x Protein Prep Buffer, 5mM imidazole, 1 cOmplete^{TM}

EDTA-free protease inhibitor cocktail tablet (Roche, 05056489001)) and lysed via sonication. They were then centrifuged at 15,000 rpm for 30 minutes. The supernatant was incubated for 1h at 4°C with 200µl HisPur^{TM} Ni-NTA Resin (Thermo Scientific, 88222) pre-equilibrated in 1x Protein Prep Buffer, before centrifugation at 700 g for 2 minutes. The supernatants were decanted and the beads washed six times in Wash Buffer (1X Protein Prep Buffer, 30mM imidazole), spinning at 700 g for 1 minute each time. The beads were then eluted five times in Elution Buffer (1X Protein Prep Buffer, 300mM imidazole), spinning at 700 g for 2 minutes each time.

For dialysis, eluted protein was dialysed in Spectra/Por®6 Dialysis Membrane 15kDa Pre-Wetted RC Tubing (SpectrumLabs, 132562), stirring in 1L Storage Buffer (20mM Tris/HCl (pH7.5), 100mM NaCl, 0.5mM EDTA, 20% glycerol, 1mM DTT) at 4°C overnight. The resulting samples were aliquoted, snap frozen and stored at -80°C. Protein concentration was determined via Bradford assays and Coomassie blue-stained 12% SDS-PAGE gels.

Proteomics Mass Spectrometry
To check successful AzF incorporation into GST, the protein was run on a 12% SDS-PAGE gel, and an in-gel trypsin digestion carried out. Briefly, the gel bands were cut out after Coomassie blue staining, and de-stained in 50% ethanol and 50mM ammonium bicarbonate (ABC) three times for 20 minutes each. After dehydration in ethanol for 5 minutes, the gels were reduced in 10mM TCEP and 40mM CAA for 5 minutes at 70°C. After three further 10-minute washes in 50% ethanol and 50mM ABC, the gels were dehydrated again in ethanol for 5 minutes. 2.5 ng/µl trypsin was added in sufficient quantity to hydrate the gel, and then topped up with 50mM ABC to submerge the gels, which were left at 37°C overnight, after which liquid was collected. To extract any remaining peptides, the gels were submerged in 25% acetonitrile and 5% formic acid and sonicated for 10 minutes in a water bath three times, keeping the liquid each time. All liquid extractions were combined and concentrated via speed vacuum. Finally, these peptides were resuspended in 2% acetonitrile and 0.1% trifluoroacetic acid to a final volume of 50µl.

For mass spectrometry of peptides, NanoLC-ESI-MS/MS analysis was used. Reversed phase chromatography was used to separate tryptic peptides prior to mass spectrometric analysis. Two C18 columns were utilised, an Acclaim PepMap µ-precolumn cartridge 300 µm i.d. x 5 mm 5 µm 100 Å (Thermo Fisher Scientific) and a 75 µm x 40 cm 1.9 µm (Bruker nanoElute Forty Analytical column). The columns were installed on a NanoElute UHPLC system (Bruker Daltonics, Germany). Mobile phase buffer A was composed of 0.1% formic acid in water and mobile phase B 0.1 % formic acid in acetonitrile. Samples were loaded on onto the µ-precolumn equilibrated in 2% aqueous acetonitrile containing 0.1% formic acid and peptides were eluted onto the analytical column at 350 nL min^{-1} by increasing the mobile phase B concentration from 3% B to 15% over 31 min, then to 35% B over 10 min, and to 85% B over 3 min, followed by a 10 min re-equilibration at 0% B. NanoElute was coupled online to a hybrid timsTOF Pro (Bruker Daltonics, Germany) via a CaptiveSpray nano-electrospray ion source [1]. The timsTOF Pro was operated in Data-Dependent Parallel Accumulation-Serial Fragmentation (PASEF) mode. Peptides were separated by ion mobility depending on their collisional cross sections and charge states. The method settings were as follows: mass range 100 to 1700 m/z, ion mobility range 1/K0 Start 0.6 Vs/cm^2 End 1.6 Vs/cm^2, Ramp rate 9.42 Hz and Duty cycle 100%. For data analysis, the raw data were searched

using FragPipe version 18.0 (https://fragpipe.nesvilab.org/) against *Homo sapiens* database (www.uniprot.org/proteomes) and common contaminant database. For the database search, peptides were generated from a tryptic digestion with up to two missed cleavages, carbamidomethylation of cysteines as fixed modifications. Oxidation of methionine and acetylation of the protein N-terminus were added as variable modifications. Scaffold software version 5 (https://www.proteomesoftware.com/products/scaffold-5), Perseus (https://maxquant.net/perseus/) and Mascot (http://www.matrixscience.com) were used to analyse the results. The authors Acknowledge Dr Cleidi Zampronio and the Warwick Proteomics Research Technology Platform for the mass spectrometry analysis of the protein sequence.

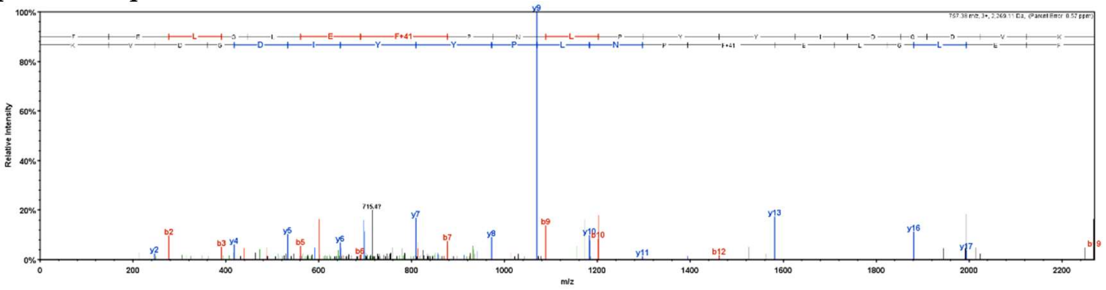

[1] Meier F., Brunner A.D., Koch S., Koch H., Lubeck M., Krause M., Goedecke N., Decker J., Kosinski T., Park M.A., Bache N., Hoerning O., Cox J., Räther O., Mann M. Online parallel accumulation–serial fragmentation (PASEF) with a novel trapped ion mobility mass spectrometer. Mol. Cell. Proteom. 2018;17:2534–2545. doi: 10.1074/mcp.TIR118.000900.

In vitro **Click Reactions and Mass Spectrometry**

Each strained alkyne was added to a final concentration of 5mM in 20µl 150µM GST-AzF protein and left at room temperature in the dark overnight. 1M pH 7.5 Tris/HCl was added to a final volume of 500µl. The samples were spun for 15 minutes at 14,000 g in a 0.5ml 10kDa Amicon® filter (Millipore, UFC501008). The filters were washed six times in 500µl 35mM ammonium acetate (pH 7.4), spinning at 14,000 g for 15 minutes each time. Samples were collected by centrifuging the inverted filter at 1,000 g for 2 minutes. Each sample was topped up to 500µl in 35mM ammonium acetate (pH7.4).

Procedure for mass spectrometry of clicked proteins:

Instrument: Bruker MaXis II coupled with Dionex 3000RS UHPLC
Column: C4, 100x2.1mm, 2.7um
Gradient: 5-100% Water/CAN
Flow rate: 0.2ml/min

The results of the mass spectrometric analyses of the click reactions are listed below:

1) Azide-labelled GST protein before click reactions;

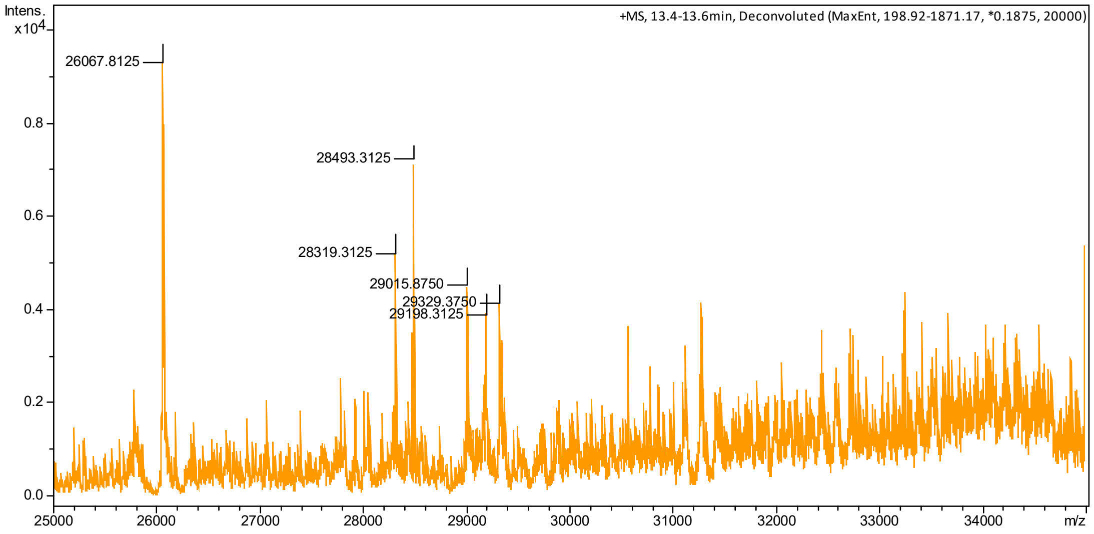

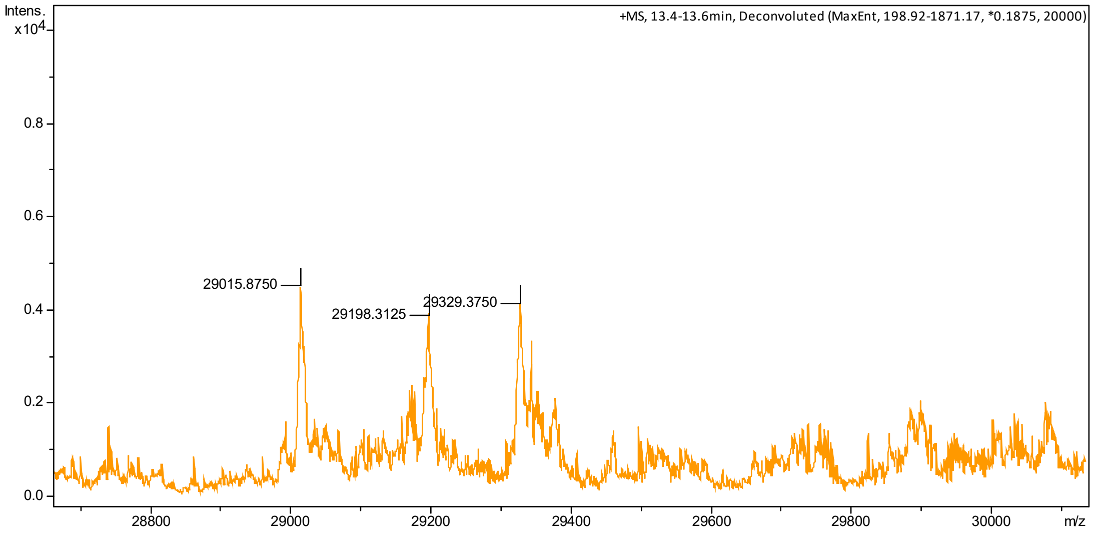

2) **WS9. Mono-Chalcone** Diphenyl Strained Alkyne **16** MW = 409 Da
Fluorescent (Ex = 420nm, Em = 520nm)

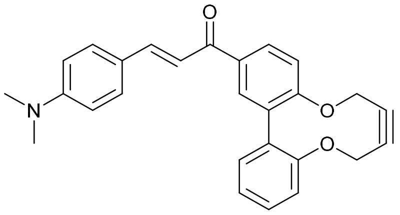

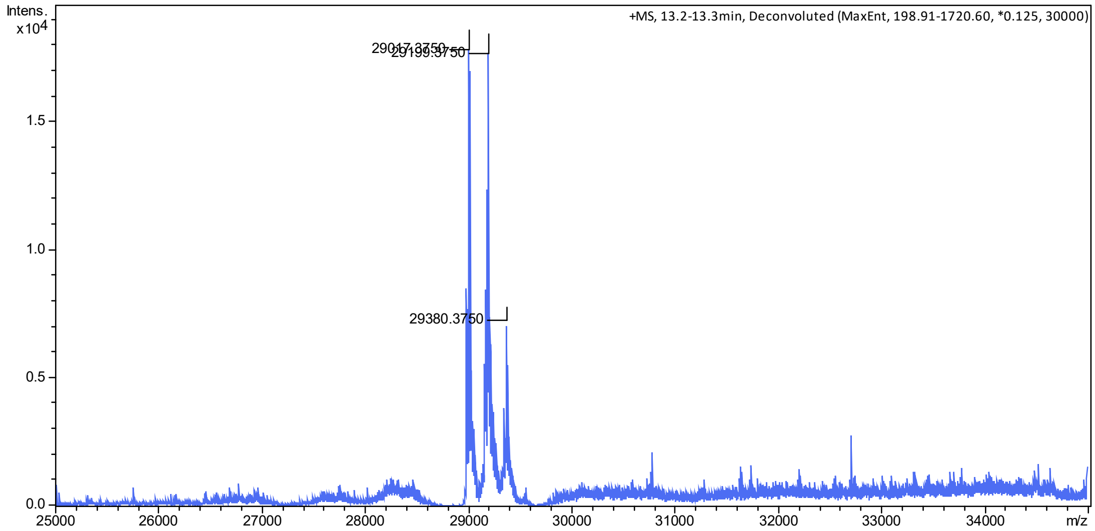

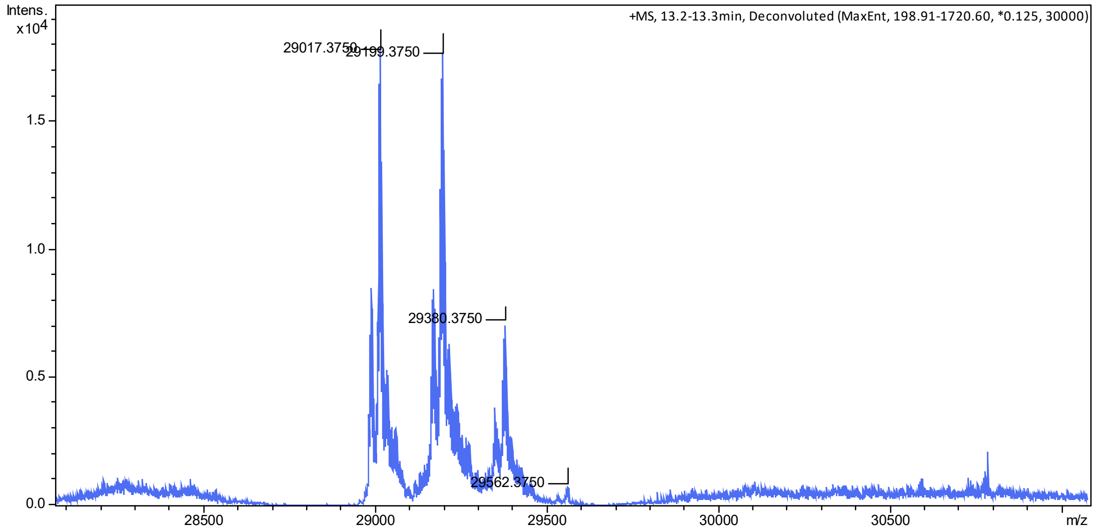

No click reaction observed.

3) Bis-chalcone 22: MW = 582 Da
Fluorescent (Ex = 420nm, Em = 520nm)

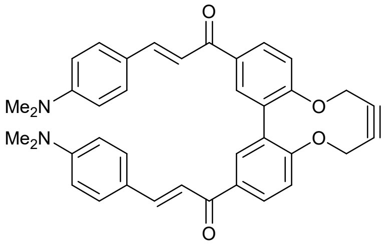

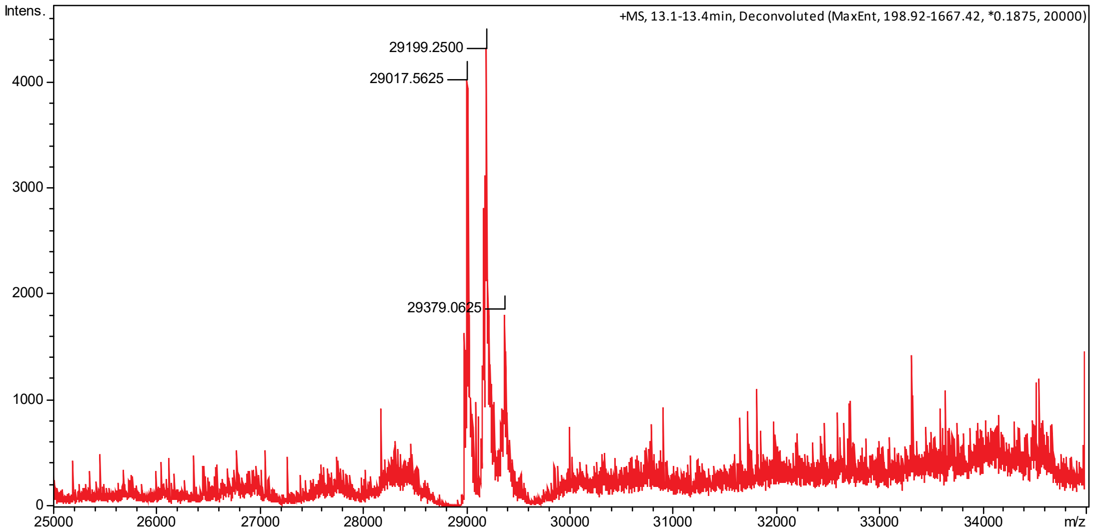

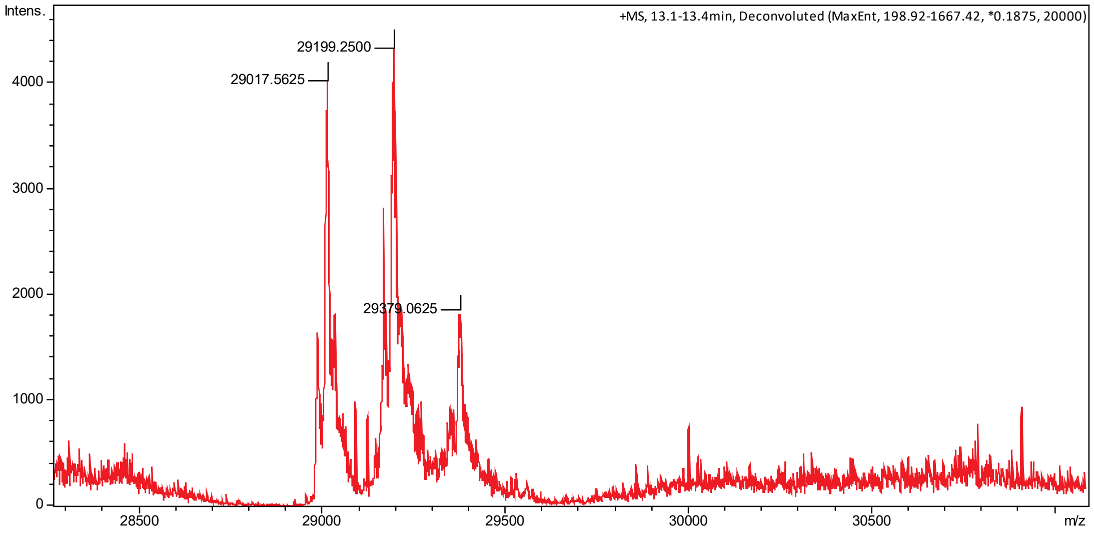

No click reaction observed.

4) 4C-Bridged Diphenyl Strained Alkyne 44.

MW = 322 Da Not fluorescent

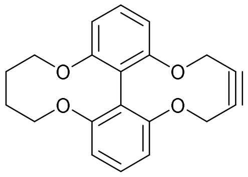

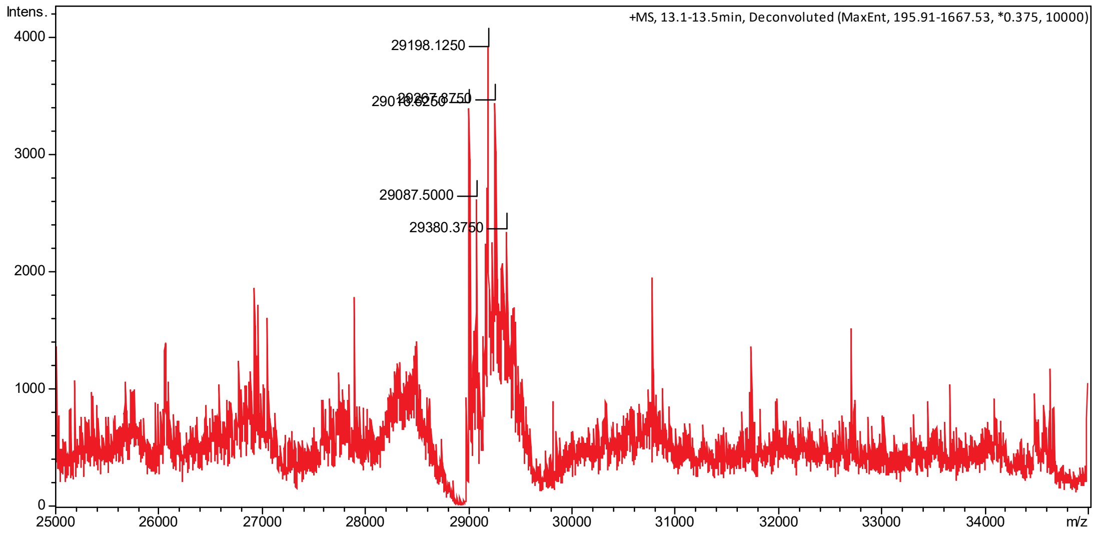

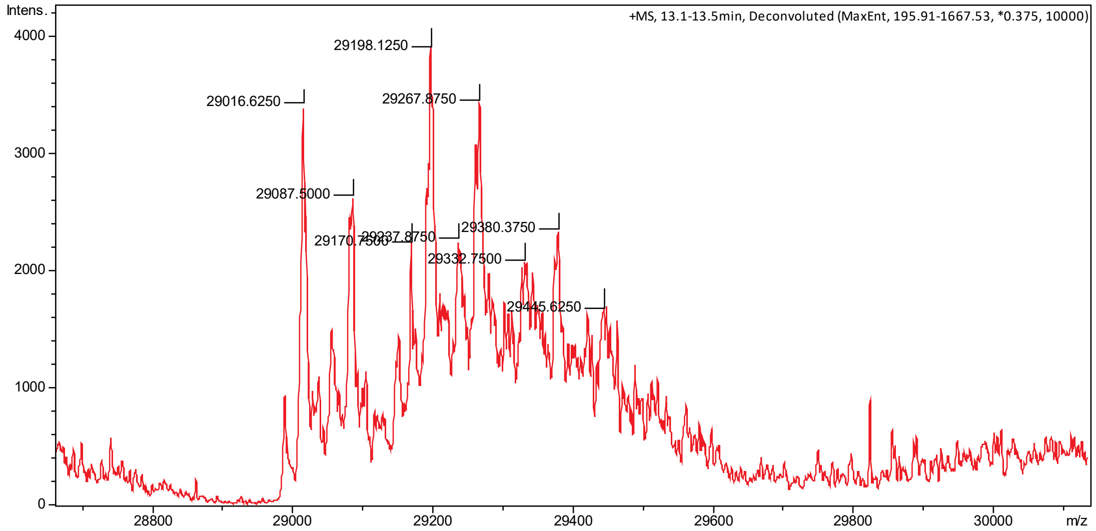

Weak click reaction observed; 29017 to 29332.

5) Bis-Dansyl Diphenyl Strained Alkyne 52.

MW = 732 Da Fluorescent.

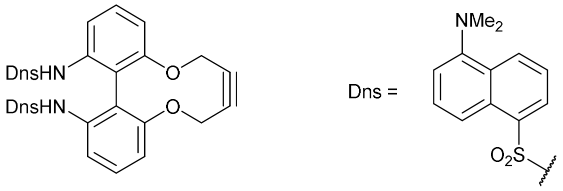

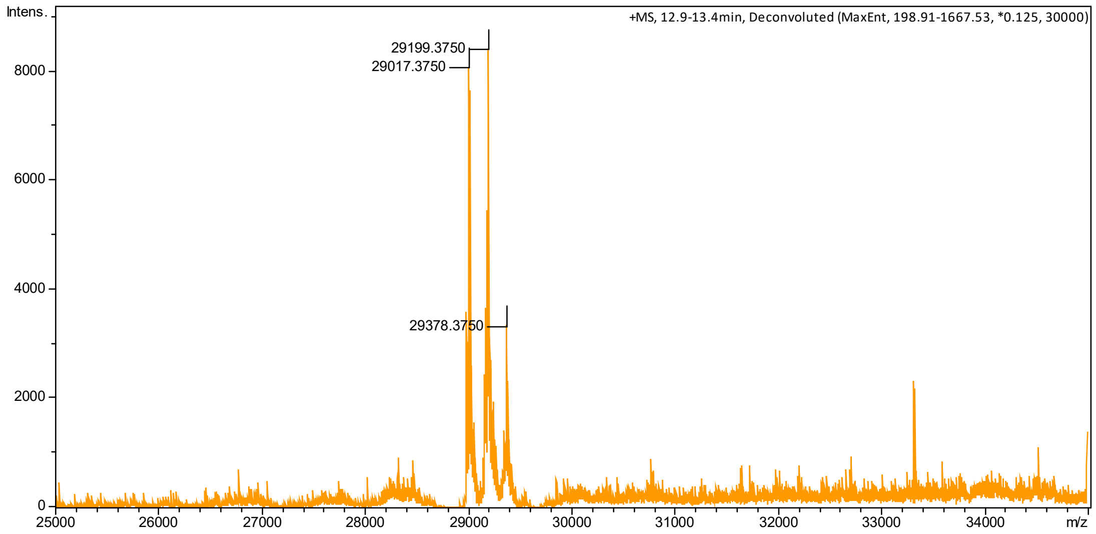

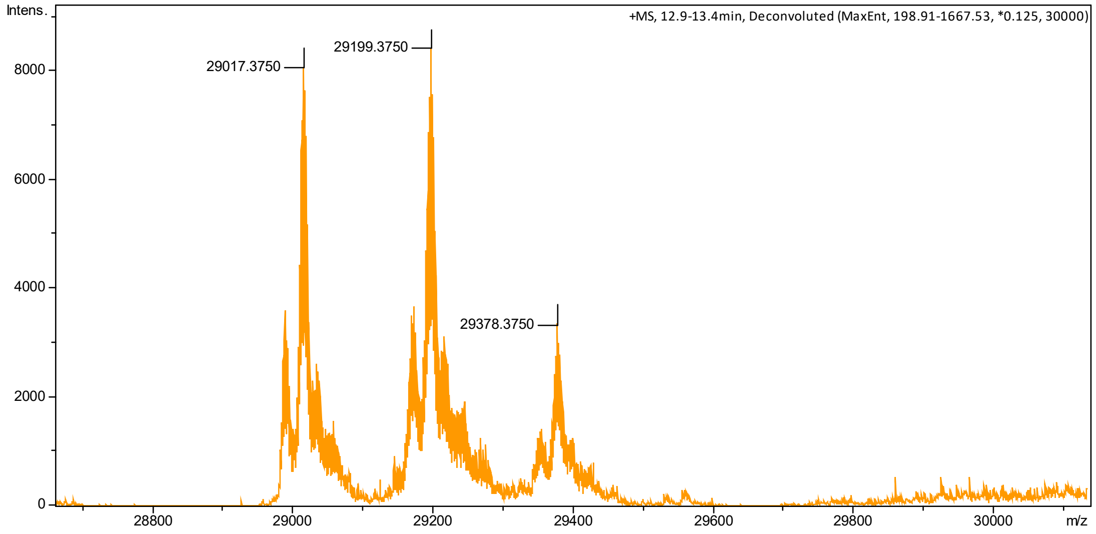

No click product observed.

6) 4C-Bridged Bis-Mesyl Diphenyl Strained Alkyne 56.

MW = 476 Da

Not fluorescent

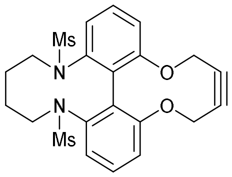

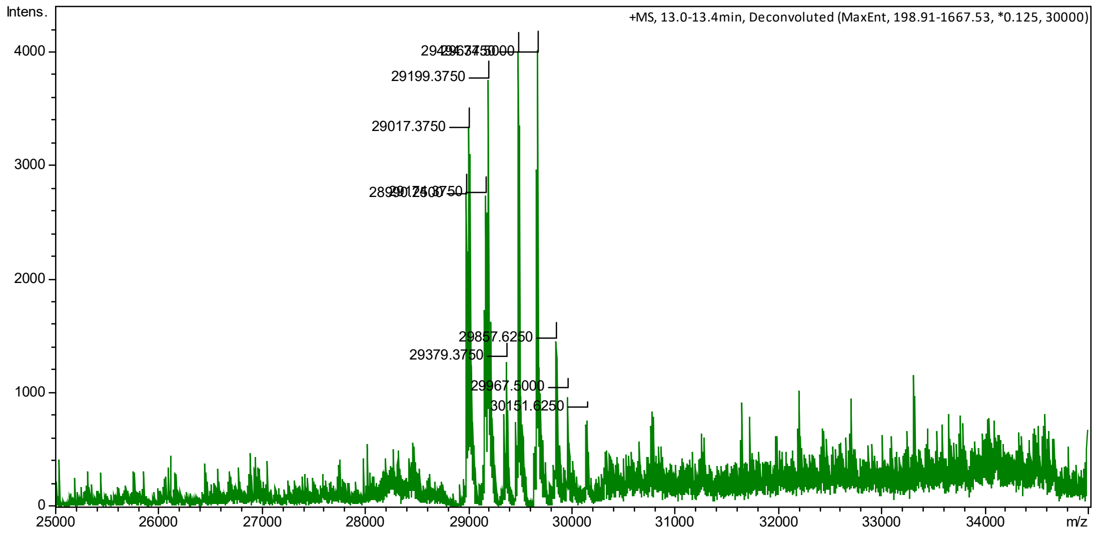

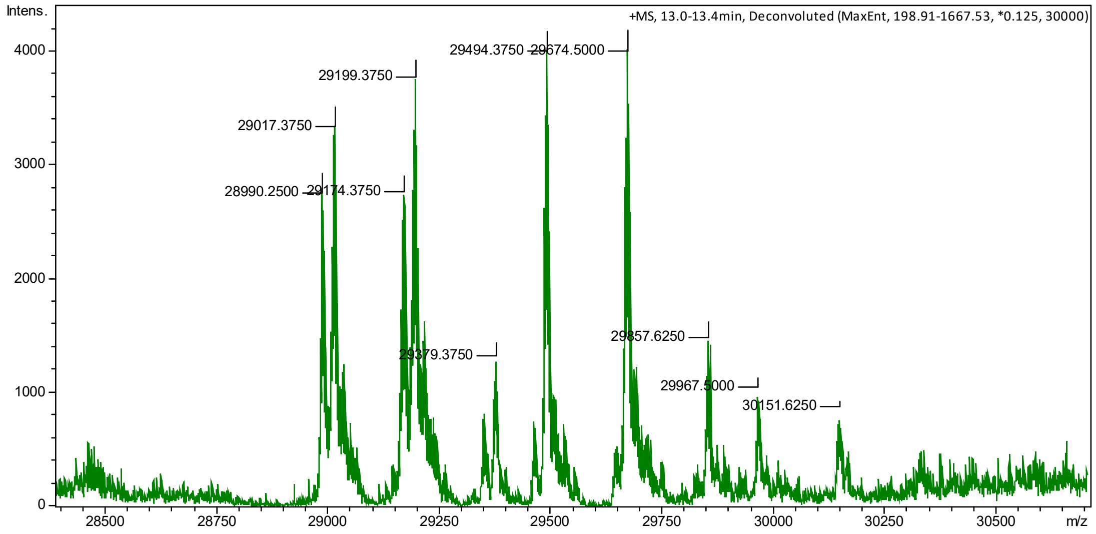

Click addition observed. 29017 to 29494 (+477), 29199 to 29675 (+476).

8) 4C-bridged Bis-Tosyl Diphenyl Strained Alkyne 54.

MW = 628 Da

Not fluorescent

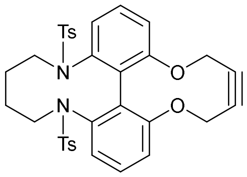

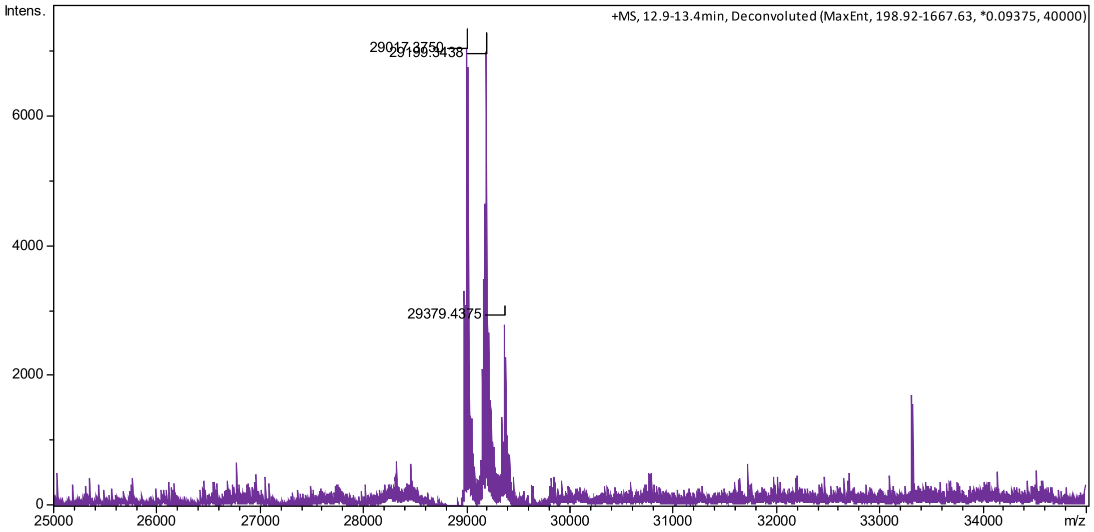

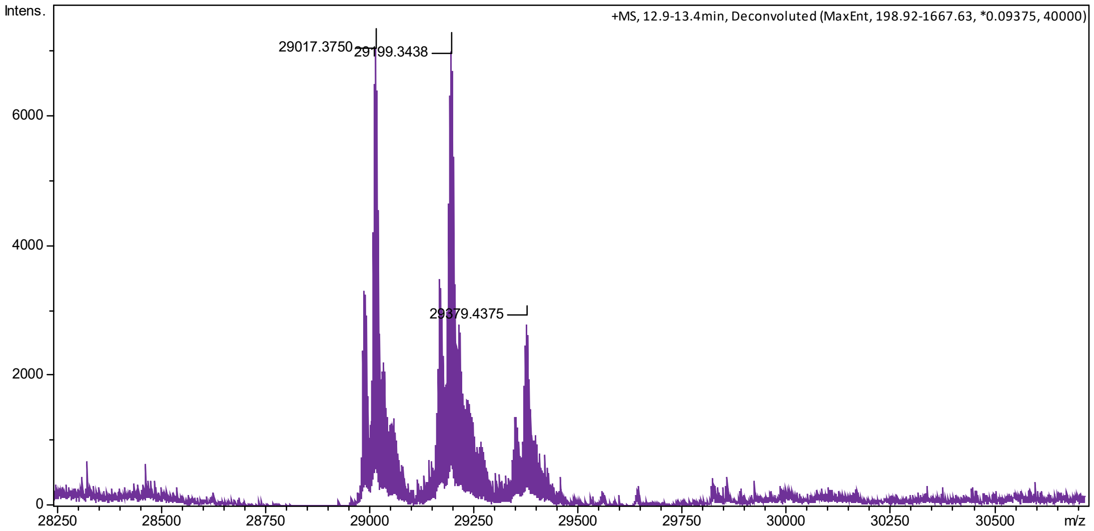

No click reaction observed.

Summary Table of second set of mass spectrometry studies:

sample	MW of click reagent	Observed unmodified	observed modified	difference vs observed peaks	difference vs standard peaks	Clicked?
Azidoprotein		29015.875				
GSTAzF	n/a	29198.3125				
		29329.3759				
**16**		29017.375				no click
Mono Chalcone	409	29199.375				
		29380.375				
**22**		29017.5625				no click
DiChal	582	29199.25				
		29379.0625				
**44**		29016	29332		316.125	low level
4Calkyne	322	29198	29380			
		29380	29445			
**52**		29017.375				no click
BisDans	732	29199.375				
		29378.375				
**56**		29017.375	29494.38	477	478.5	excellent fit
4CBisMs	476	29199.375	29674.5	475.125	476.1875	excellent fit
		29379.375	29857.63	478.25	528.2491	n/a
**54**		29017.375				no click
4CBisTs	628	29199.3438				
		29379.4379				

Excel sheets for each kinetic run:

Reaction of compound **16** (monochalcone) with benzylazide in $CDCl_3$.

Compound **16** (9.7 mg, 24 µmol) and BnN3 (3.2 mg, 24 µmol) in $CDCl_3$ (0.5 mL, [alkyne](initial) = 47 mM.

* [alkyne] is in M/1000 so that gradient of 1/[S] vs time/s is given as $mM^{-1}s^{-1}$ instead of $M^{-1}s^{-1}$

start date/time	end of interval	interval time elapsed/s	total time elapsed/s	time/h	integration of product peak	No. Hs in environment	Integration of SM peak	No. Hs in environment	[Alkyne]/M/1000 *	1/[Alkyne]	Conv %	Time in s
15/03/2021 08:33	15/03/2021 09:07	0:0:34:00	2040	0.57	0	1	4	4	4.74E-05	21097	0	2040
15/03/2021 09:07	15/03/2021 15:05	0:5:58:00	21480	5.97	0.25	6	1	2	4.37538E-05	22855	7.7	21480
15/03/2021 15:05	16/03/2021 09:11	0:18:06:00	86640	24.07	1.26	6	1	2	3.33803E-05	29958	29.6	86640
16/03/2021 09:11	16/03/2021 15:02	0:5:51:00	107700	29.92	1.58	6	1	2	3.1048E-05	32208	34.5	107700
16/03/2021 15:02	17/03/2021 09:05	0:18:03:00	172680	47.97	2.74	6	1	2	2.47735E-05	40366	47.7	172680
17/03/2021 09:05	17/03/2021 15:07	0:6:02:00	194400	54.00	3.26	6	1	2	2.27157E-05	44023	52.1	194400
17/03/2021 15:07	18/03/2021 09:09	0:18:02:00	259320	72.03	4.64	6	1	2	1.86126E-05	53727	60.7	259320
18/03/2021 09:09	18/03/2021 15:04	0:5:55:00	280620	77.95	5.04	6	1	2	1.76866E-05	56540	62.7	280620
18/03/2021 15:04	19/03/2021 09:06	0:18:02:00	345540	95.98	6.48	6	1	2	0.000015	66667	68.4	345540
19/03/2021 09:06	19/03/2021 15:07	0:6:01:00	367200	102.00	7.05	6	1	2	1.41493E-05	70675	70.1	367200
19/03/2021 15:07	20/03/2021 09:06	0:17:59:00	431940	119.98	8.8	6	1	2	1.20508E-05	82982	74.6	431940
20/03/2021 09:06	20/03/2021 15:05	0:5:59:00	453480	125.97	9.35	6	1	2	1.15142E-05	86850	75.7	453480
20/03/2021 15:05	21/03/2021 09:06	0:18:01:00	518340	143.98	11.47	6	1	2	9.82723E-06	101758	79.3	518340
21/03/2021 09:06	21/03/2021 15:05	0:5:59:00	539880	149.97	11.96	6	1	2	9.50535E-06	105204	79.9	539880
21/03/2021 15:05	22/03/2021 09:06	0:18:01:00	604740	167.98	14.39	6	1	2	8.17711E-06	122293	82.7	604740
22/03/2021 09:06	22/03/2021 15:05	0:5:59:00	626280	173.97	14.62	6	1	2	8.07037E-06	123910	83.0	626280
22/03/2021 15:05	23/03/2021 09:07	0:18:02:00	691200	192.00	17.89	6	1	2	6.80708E-06	146906	85.6	691200
23/03/2021 09:07	23/03/2021 15:06	0:5:59:00	712740	197.98	18.47	6	1	2	6.6232E-06	150985	86.0	712740
23/03/2021 15:06	24/03/2021 09:06	0:18:00:00	777540	215.98	21.53	6	1	2	5.79698E-06	172504	87.8	777540
24/03/2021 09:06	24/03/2021 15:05	0:5:59:00	799080	221.97	22.65	6	1	2	5.54386E-06	180380	88.3	799080

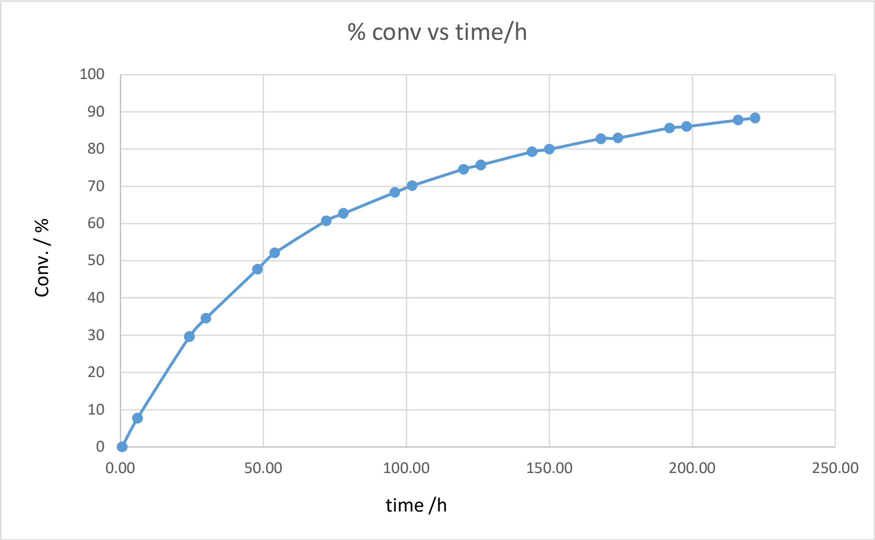

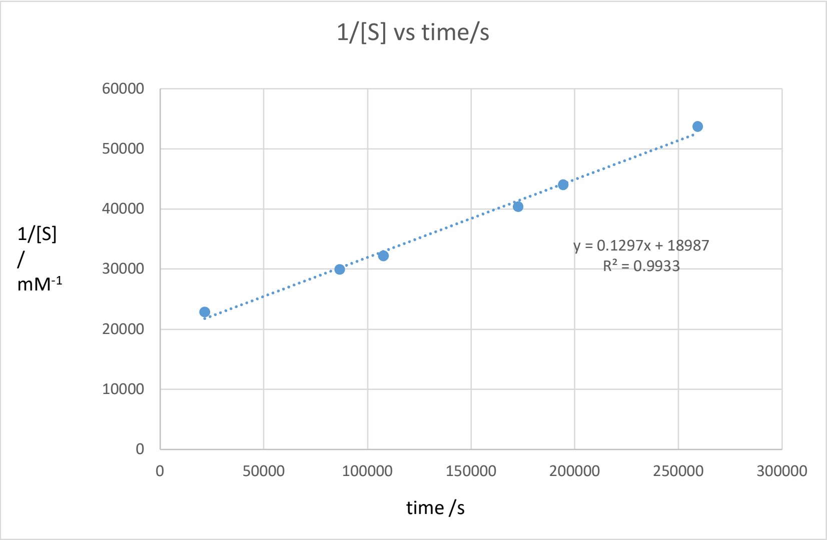

k = 0.13 mM^{-1} s^{-1}

Reaction of compound **22** (dichalcone) with benzylazide in $CDCl_3$.

Compound **16** (10 mg, 17 µmol) and BnN_3 (2.3 mg, 17 µmol) in $CDCl_3$ (0.5 mL), [alkyne](initial) = 34 mM.

* [alkyne] is in M/1000 so that gradient of 1/[S] vs time/s is given as $mM^{-1}\ s^{-1}$ instead of $M^{-1}s^{-1}$

start date/time	end of interval	time elapsed	interval time/s	time/s	ime/h	integration of product peak	No. Hs in environment	Integration of SM peak	No. Hs in environment	Conv	[Alkyne]/M/ 1000	1/[Alkyne]	Conv %	time/s
15/03/2021 08:35	15/03/2021 09:13	0:0:38:00	2280	2280	0.63	0	6	0.4	2	0.000	3.40E-05	29412	0.0	2280
15/03/2021 09:07	15/03/2021 15:11	0:6:04:00	21840	21840	6.07	0.24	6	1	2	0.074	3.1481E-05	31765	7.4	21840
15/03/2021 15:11	16/03/2021 09:18	0:18:07:00	65220	87060	24.18	1.54	6	1	2	0.339	2.2467E-05	44510	33.9	87060
16/03/2021 09:18	16/03/2021 15:09	0:5:51:00	21060	108120	30.03	1.92	6	1	2	0.390	2.0732E-05	48235	39.0	108120
16/03/2021 15:09	17/03/2021 09:11	0:18:02:00	64920	173040	48.07	3.65	6	1	2	0.549	1.5338E-05	65196	54.9	173040
17/03/2021 09:11	17/03/2021 15:13	0:6:02:00	21720	194760	54.10	4.2	6	1	2	0.583	1.4167E-05	70588	58.3	194760
17/03/2021 15:13	18/03/2021 09:15	0:18:02:00	64920	259680	72.13	6.76	6	1	2	0.693	1.0451E-05	95686	69.3	259680
18/03/2021 09:15	18/03/2021 15:10	0:5:55:00	21300	280980	78.05	7.06	6	1	2	0.702	1.0139E-05	98627	70.2	280980
18/03/2021 15:10	19/03/2021 09:13	0:18:03:00	64980	345960	96.10	10.22	6	1	2	0.773	7.7156E-06	129608	77.3	345960
19/03/2021 09:13	19/03/2021 15:13	0:6:00:00	21600	367560	102.10	11.16	6	1	2	0.788	7.2034E-06	138824	78.8	367560
19/03/2021 15:13	20/03/2021 09:12	0:17:59:00	64740	432300	120.08	14.91	6	1	2	0.832	5.6951E-06	175588	83.2	432300
20/03/2021 09:12	20/03/2021 15:11	0:5:59:00	21540	453840	126.07	16.6	6	1	2	0.847	5.2041E-06	192157	84.7	453840
20/03/2021 15:11	21/03/2021 09:12	0:18:01:00	64860	518700	144.08	22.16	6	1	2	0.881	4.0541E-06	246667	88.1	518700
21/03/2021 09:12	21/03/2021 15:12	0:6:00:00	21600	540300	150.08	23.86	6	1	2	0.888	3.7975E-06	263333	88.8	540300
21/03/2021 15:12	22/03/2021 09:12	0:18:00:00	64800	605100	168.08	31.58	6	1	2	0.913	2.9497E-06	339020	91.3	605100
22/03/2021 09:12	22/03/2021 15:11	0:5:59:00	21540	626640	174.07	32.89	6	1	2	0.916	2.842E-06	351863	91.6	626640
22/03/2021 15:11	23/03/2021 09:13	0:18:02:00	64920	691560	192.10	44.02	6	1	2	0.936	2.1693E-06	460980	93.6	691560
23/03/2021 09:13	23/03/2021 15:12	0:5:59:00	21540	713100	198.08	49.92	6	1	2	0.943	1.9274E-06	518824	94.3	713100
23/03/2021 15:12	24/03/2021 09:11	0:17:59:00	64740	777840	216.07	66.88	6	1	2	0.957	1.4596E-06	685098	95.7	777840
24/03/2021 09:11	24/03/2021 15:12	0:6:01:00	21660	799500	222.08	73.27	6	1	2	0.961	1.3374E-06	747745	96.1	799500

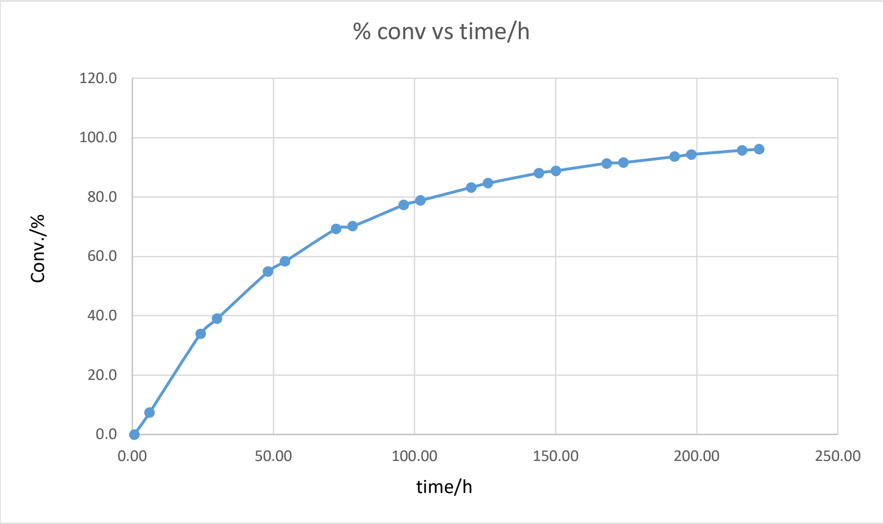

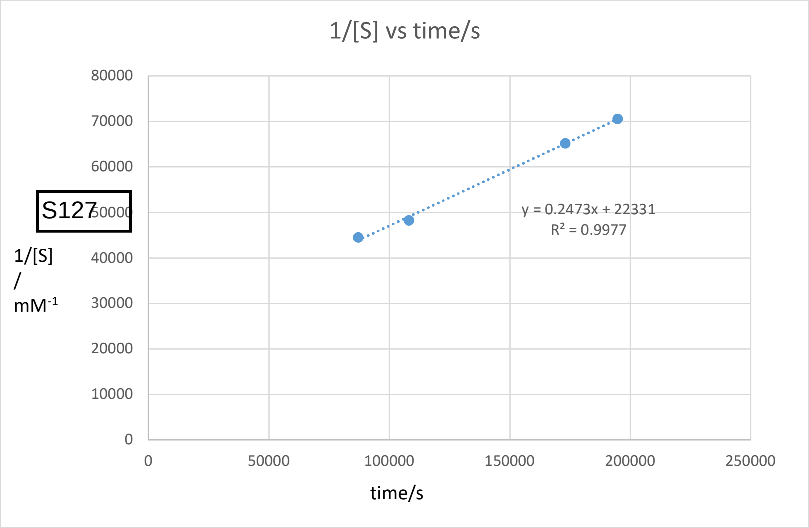

k = 0.025 $mM^{-1}s^{-1}$

Reaction of compound **29** (NTs-O) with benzylazide in $CDCl_3$.

Compound **39** (20 mg, 51 µmol) and BnN_3 (6.6 mg, 51 µmol) in $CDCl_3$ (0.5 mL), [alkyne](initial) = 97 mM.

* [alkyne] is in M/1000 so that gradient of 1/[S] vs time/s is given as $mM^{-1}\ s^{-1}$ instead of $M^{-1}s^{-1}$

start date/time	end of interval	time elapsed	Interval time/s	time/h	time/s	integration of product peak	No. Hs in environment	Integration of SM peak	No. Hs in environment	Conv	[Alkyne]	1/[Alkyne]	Conv %
	05/11/2018 17:09			0	0	2	3	86.75	3	0.000	9.70E-05	10309	0.0
05/11/2018 17:09	06/11/2018 10:29	0:17:20:00	62400	17.3	62400	2	3	11.7	3	0.146	8.28394E-05	12072	14.6
06/11/2018 10:29	07/11/2018 10:01	0:23:32:00	84720	40.9	147120	2	3	5.37	3	0.271	7.06771E-05	14149	27.1
07/11/2018 10:01	08/11/2018 10:18	1:0:17:00	87420	65.2	234540	3	3	5.04	3	0.373	6.0806E-05	16446	37.3
08/11/2018 10:18	09/11/2018 10:44	1:0:26:00	87960	89.6	322500	3	3	3.5	3	0.462	5.22308E-05	19146	46.2
09/11/2018 10:44	12/11/2018 14:34	3:3:50:00	273000	165.4	595500	5.54	3	3	3	0.649	3.40749E-05	29347	64.9
12/11/2018 14:34	13/11/2018 10:26	0:19:52:00	71520	185.3	667020	3	3	1.46	3	0.673	3.17534E-05	31493	67.3
13/11/2018 10:26	14/11/2018 19:36	1:9:10:00	119400	218.4	786420	3	3	1.21	3	0.713	2.78789E-05	35869	71.3
14/11/2018 19:36	15/11/2018 10:30	0:14:54:00	53640	233.3	840060	10	3	3.83	3	0.723	2.68626E-05	37226	72.3
15/11/2018 10:30	16/11/2018 14:23	1:3:53:00	100380	261.2	940440	10	3	3.25	3	0.755	2.37925E-05	42030	75.5
16/11/2018 14:23	20/11/2018 11:36	3:21:13:00	335580	354.4	1276020	10	3	2.12	3	0.825	1.6967E-05	58938	82.5
20/11/2018 11:36	21/11/2018 13:34	1:1:58:00	93480	380.4	1369500	10	3	1.94	3	0.838	1.57605E-05	63450	83.8
21/11/2018 13:34	22/11/2018 13:16	0:23:42:00	85320	404.1	1454820	10	3	1.75	3	0.851	1.44468E-05	69219	85.1
22/11/2018 13:16	23/11/2018 11:09	0:21:53:00	78780	426.0	1533600	10	3	1.67	3	0.857	1.38809E-05	72041	85.7
23/11/2018 11:09	26/11/2018 13:42	3:2:33:00	268380	500.5	1801980	9.88	3	1.27	3	0.886	1.10484E-05	90511	88.6
26/11/2018 13:42	27/11/2018 14:09	1:0:27:00	88020	525.0	1890000	10	3	1.17	3	0.895	1.01603E-05	98423	89.5

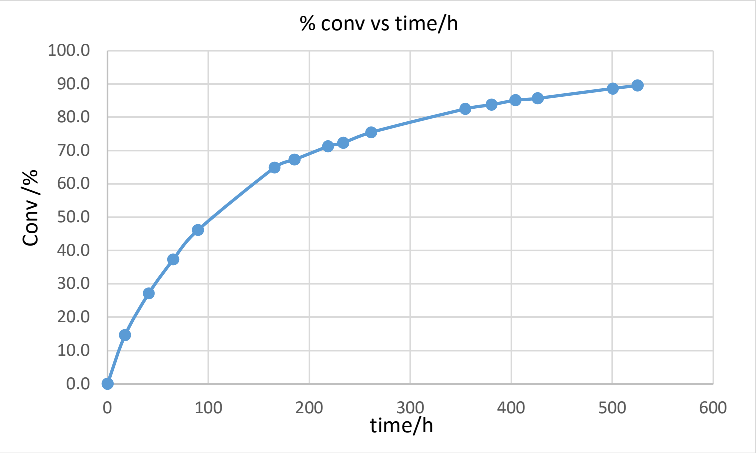

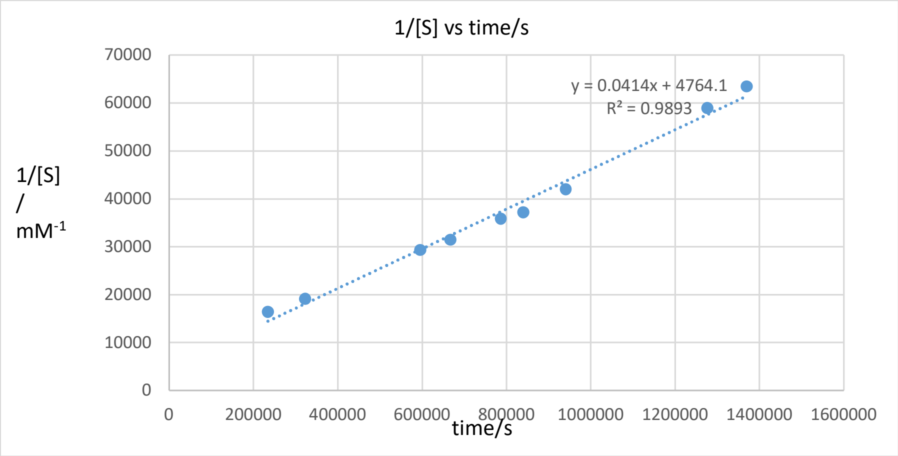

k = 0.041 $mM^{-1}s^{-1}$

Reaction of compound **37** (DiOMe) with benzylazide in $CDCl_3$.

Compound **37** (7.0 mg, 26 µmol) and BnN_3 (2.7 mg, 23 µmol) in $CDCl_3$ (0.5 mL, [alkyne](initial) = 47 mM.

* [alkyne] is in M/1000 so that gradient of 1/[S] vs time/s is given as $mM^{-1}s^{-1}$ instead of $M^{-1}s^{-1}$

start date/time	end of interval	time elapsed	interval time/s	time/s	time/h	integration of product peak	No. Hs in environment	Integration of SM peak	No. Hs in environment	Conv	[Alkyne]	1/[Alkyne]	Conv/%
17/03/2020 09:30	17/03/2020 10:03	0:0:33:00	1980	1980	0.55	1	1	92.44	2	0.021	4.00E-05	25000	2.1
17/03/2020 10:03	17/03/2020 14:55	0:4:52:00	17520	19500	5.4	1	1	12.26	2	0.140	3.44E-05	29078	14.0
17/03/2020 14:55	18/03/2020 09:11	0:18:16:00	65760	85260	23.7	0.69	1	2	2	0.408	2.37E-05	42250	40.8
18/03/2020 09:11	18/03/2020 16:59	0:7:48:00	28080	113340	31.5	0.92	1	2	2	0.479	2.08E-05	48000	47.9
18/03/2020 16:59	19/03/2020 17:28	1:0:29:00	88140	201480	56.0	1.61	1	2	2	0.617	1.53E-05	65250	61.7
19/03/2020 17:28	20/03/2020 11:49	0:18:21:00	66060	267540	74.3	1.09	1	1	2	0.686	1.26E-05	79500	68.6
20/03/2020 11:49	23/03/2020 12:45	3:0:56:00	262560	530100	147.2	2.13	1	1	2	0.810	7.60E-06	131500	81.0
23/03/2020 12:45	24/03/2020 10:22	0:21:37:00	77820	607920	168.9	2.5	1	1	2	0.833	6.67E-06	150000	83.3

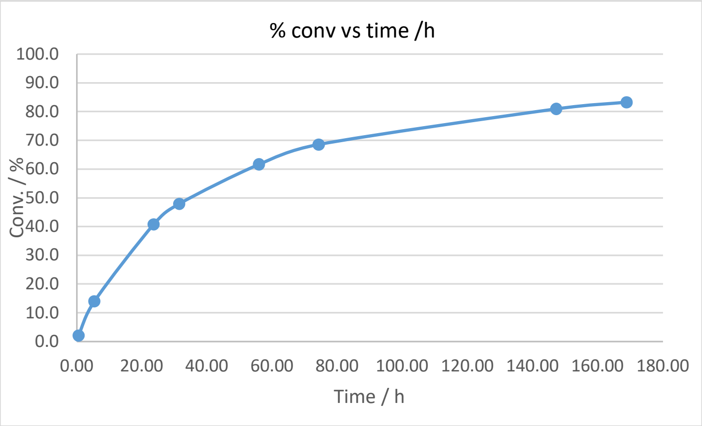

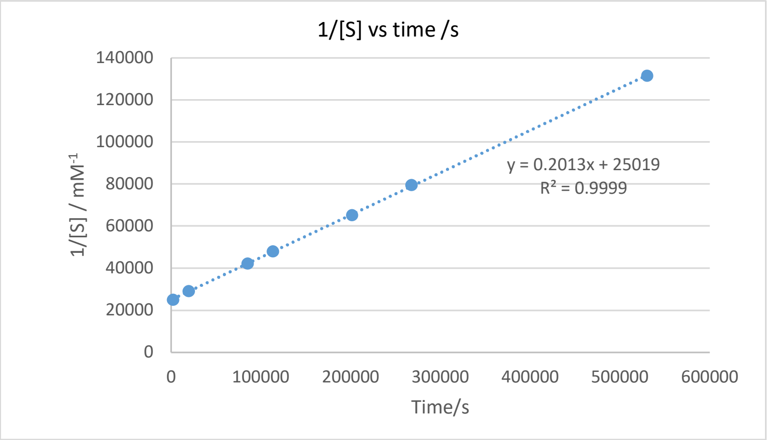

k = 0.20 $mM^{-1}s^{-1}$

Reaction of compound **44** (C4 diether bridged) with benzylazide in $CDCl_3$.

Compound **44** (10 mg, 31 µmol) and BnN_3 (4.0 mg, 30 µmol) in $CDCl_3$ (0.6 mL, [alkyne](initial) = 51.7 mM.

* [alkyne] is in M/1000 so that gradient of 1/[S] vs time/s is given as as $mM^{-1}s^{-1}$ instead of $M^{-1}s^{-1}$

start date/time	end of interval	time elapsed	interval time/s	time/s	time/h	integration of product peak	No. Hs in environment	Integration of SM peak	No. Hs in environment	Conv	[Alkyne]	1/[Alkyne]	Conv %	time/s
16/02/2021 13:55	16/02/2021 13:55	0:0:00:00	0	0	0.0	0	1	4	4	0.000	5.170E-05	19342	0.00	0
16/02/2021 13:55	16/02/2021 16:41	0:2:46:00	9960	9960	2.8	0.72	1	4	4	0.419	3.006E-05	33269	41.86	9960
16/02/2021 16:41	17/02/2021 09:15	0:16:34:00	59640	69600	19.3	1	1	0.66	4	0.858	7.322E-06	136569	85.84	69600
17/02/2021 09:15	17/02/2021 15:13	0:5:58:00	21480	91080	25.3	1	1	0.49	4	0.891	5.642E-06	177239	89.09	91080
17/02/2021 15:13	18/02/2021 09:12	0:17:59:00	64740	155820	43.3	1	1	0.26	4	0.939	3.155E-06	316917	93.90	155820
18/02/2021 09:12	18/02/2021 15:11	0:5:59:00	21540	177360	49.3	1	1	0.21	4	0.950	2.579E-06	387768	95.01	177360
18/02/2021 15:11	19/02/2021 09:19	0:18:08:00	65280	242640	67.4	1	1	0.15	4	0.964	1.869E-06	535139	96.39	242640
19/02/2021 09:19	19/02/2021 15:11	0:5:52:00	21120	263760	73.3	8.32	1	1	4	0.971	1.508E-06	663056	97.08	263760
19/02/2021 15:11	20/02/2021 09:06	0:17:55:00	64500	328260	91.2	1	1	0.09	4	0.978	1.138E-06	879003	97.80	328260
20/02/2021 09:06	20/02/2021 15:06	0:6:00:00	21600	349860	97.2	1	1	0.08	4	0.980	1.014E-06	986460	98.04	349860
20/02/2021 15:06	21/02/2021 09:05	0:17:59:00	64740	414600	115.2	14.92	1	1	4	0.984	8.520E-07	1173694	98.35	414600
21/02/2021 09:05	21/02/2021 15:05	0:6:00:00	21600	436200	121.2	15.09	1	1	4	0.984	8.426E-07	1186847	98.37	436200
21/02/2021 15:05	22/02/2021 09:08	0:18:03:00	64980	501180	139.2	21.24	1	1	4	0.988	6.014E-07	1662669	98.84	501180
22/02/2021 09:08	22/02/2021 15:09	0:6:01:00	21660	522840	145.2	25.78	1	1	4	0.990	4.965E-07	2013926	99.04	522840
22/02/2021 15:09	23/02/2021 09:15	0:18:06:00	65160	588000	163.3	1	1	0.03	4	0.993	3.849E-07	2598324	99.26	588000
23/02/2021 09:15	23/02/2021 15:08	0:5:53:00	21180	609180	169.2	5	1	0.12	4	0.994	3.083E-07	3243069	99.40	609180

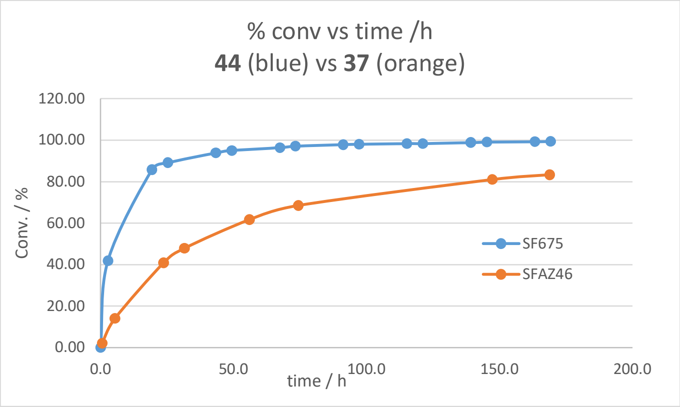

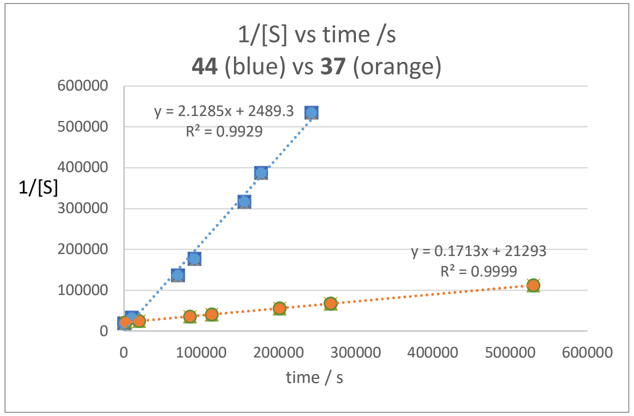

k = 2.13 $mM^{-1}s^{-1}$

Compound **37** (SFAZ46) data for comparison

time/s	time/h	1/[Alkyne]	Conv %
1980	0.55	21277	2.1
19500	5.42	24747	14.0
85260	23.68	35957	40.8
113340	31.48	40851	47.9
201480	55.97	55532	61.7
267540	74.32	67660	68.6
530100	147.25	111915	81.0
607920	168.87	127660	83.3

Reaction of compound **49** (diNitro) with benzylazide in $CDCl_3$.

Compound **49** (9.0 mg, 27 μmol) and BnN_3 (3.6 mg, 27 μmol) in $CDCl_3$ (0.5 mL, [alkyne](initial) = 55 mM.

* [alkyne] is in M/1000 so that gradient of 1/[S] vs time/s is given as $mM^{-1}s^{-1}$ instead of $M^{-1}s^{-1}$

start date/time	end of interval	time elapsed	interval time/s	time/s	time/h	integration of product peak	No. Hs in environment	Integration of SM peak	No. Hs in environment	Conv	[Alkyne]/M	1/[Alkyne]	Conv/%	Time/s
17/05/2021 08:40	17/05/2021 09:07	0:0:27:00	1620	1620	0.45	1	2	20.66	2	0.046	5.50E-05	18182	4.62	1620
17/05/2021 09:07	17/05/2021 15:06	0:5:59:00	21540	21540	5.98	1	2	2.53	2	0.283	3.9419E-05	25368	28.33	21540
17/05/2021 15:06	18/05/2021 09:07	0:18:01:00	64860	86400	24.00	2	2	0.76	2	0.725	1.5145E-05	66029	72.46	86400
18/05/2021 09:07	18/05/2021 15:07	0:6:00:00	21600	108000	30.00	3.78	2	1	2	0.791	1.1506E-05	86909	79.08	108000
18/05/2021 15:07	19/05/2021 09:06	0:17:59:00	64740	172740	47.98	11.07	2	1	2	0.917	4.5568E-06	219455	91.71	172740
19/05/2021 09:06	19/05/2021 15:05	0:5:59:00	21540	194280	53.97	20.28	2	1	2	0.953	2.5846E-06	386909	95.30	194280
19/05/2021 15:05	20/05/2021 09:05	0:18:00:00	64800	259080	71.97	3	2	0.06	2	0.980	1.0784E-06	927273	98.04	259080
20/05/2021 09:05	20/05/2021 15:03	0:5:58:00	21480	280560	77.93	3	2	0.05	2	0.984	9.0164E-07	1109091	98.36	280560
20/05/2021 15:03	21/05/2021 08:22	0:17:19:00	62340	342900	95.25	3	2	0	2	1	0	#DIV/0!	100	342900
21/05/2021 08:22	21/05/2021 20:27	0:12:05:00	43500	386400	107.33	3	2	0	2	1	0	#DIV/0!	100	386400
21/05/2021 20:27	22/05/2021 08:20	0:11:53:00	42780	429180	119.22	3	2	0	2	1	0	#DIV/0!	100	429180
22/05/2021 08:20	22/05/2021 20:20	0:12:00:00	43200	472380	131.22	3	2	0	2	1	0	#DIV/0!	100	472380
22/05/2021 20:20	23/05/2021 08:22	0:12:02:00	43320	515700	143.25	3	2	0	2	1	0	#DIV/0!	100	515700
23/05/2021 08:22	23/05/2021 20:21	0:11:59:00	43140	558840	155.23	3	2	0	2	1	0	#DIV/0!	100	558840
23/05/2021 20:21	24/05/2021 09:06	0:12:45:00	45900	604740	167.98	3	2	0	2	1	0	#DIV/0!	100	604740

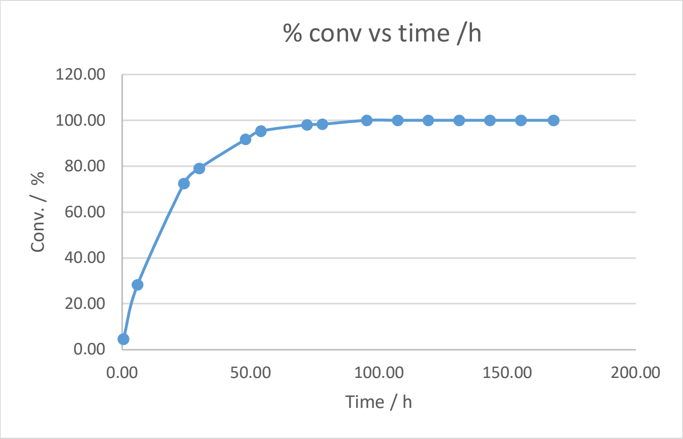

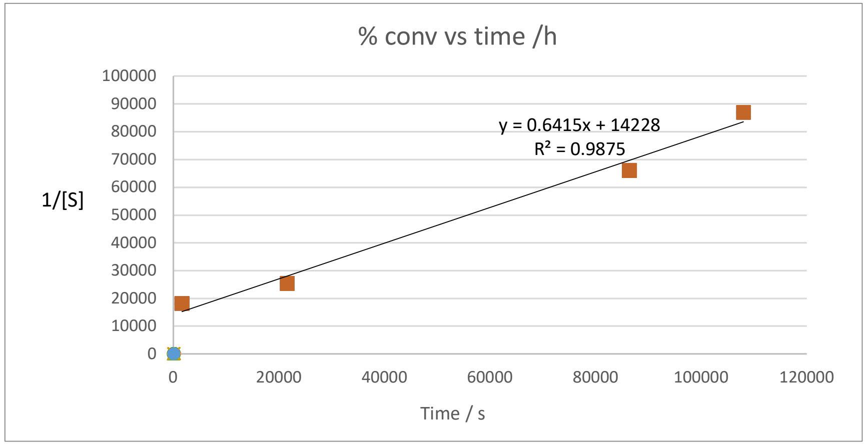

k = 0.64 $mM^{-1}s^{-1}$

Reaction of compound **50** (diamine) with benzylazide in $CDCl_3$.

Compound **50** (3.0 mg, 11 µmol) and BnN_3 (1.5 mg, 11 µmol) in $CDCl_3$ (0.65 mL, [alkyne](initial) = 17 mM.

* [alkyne] is in M/1000 so that gradient of 1/[S] vs time/s is given as $mM^{-1}s^{-1}$ instead of $M^{-1}s^{-1}$

start date/time	end of interval	time elapsed	interval time/s	time/s	time/h	integration of product peak	No. Hs in environment	Integration of SM peak	No. Hs in environment	Conv	[Alkyne]	1/[Alkyne]	conv/%	time/s
20/07/2021 10:25	20/07/2021 11:09	0:0:44:00	2640	2640	0.73	0	1	0.1	4	0.000	1.70E-05	58824	0	2640
20/07/2021 11:09	20/07/2021 15:30	0:4:21:00	15660	15660	4.35	0.03	1	1	2	0.057	1.60E-05	62353	5.7	15660
20/07/2021 15:30	21/07/2021 09:47	0:18:17:00	65820	81480	22.63	0.22	1	1	2	0.306	1.18E-05	84706	30.6	81480
21/07/2021 09:47	21/07/2021 13:48	0:4:01:00	14460	95940	26.65	0.25	1	1	2	0.333	1.13E-05	88235	33.3	95940
21/07/2021 13:48	22/07/2021 09:45	0:19:57:00	71820	167760	46.60	1.08	1	2.39	2	0.475	8.93E-06	111986	47.5	167760
22/07/2021 09:45	23/07/2021 10:47	1:1:02:00	90120	257880	71.63	1.75	1	2	2	0.636	6.18E-06	161765	63.6	257880
23/07/2021 10:47	24/07/2021 12:52	1:2:05:00	93900	351780	97.72	3.04	1	2	2	0.752	4.21E-06	237647	75.2	351780
24/07/2021 12:52	25/07/2021 15:40	1:2:48:00	96480	448260	124.52	2.39	1	1	2	0.827	2.94E-06	340000	82.7	448260
25/07/2021 15:40	26/07/2021 12:52	0:21:12:00	76320	524580	145.72	3.31	1	1	2	0.869	2.23E-06	448235	86.9	524580
26/07/2021 12:52	26/07/2021 16:31	0:3:39:00	13140	537720	149.37	3.76	1	1	2	0.883	2.00E-06	501176	88.3	537720
26/07/2021 16:31	27/07/2021 10:35	0:18:04:00	65040	602760	167.43	4.04	1	1	2	0.890	1.87E-06	534118	89.0	602760

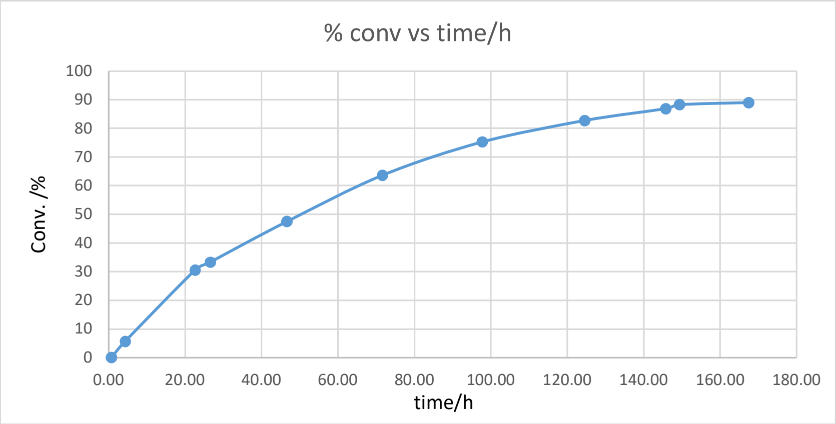

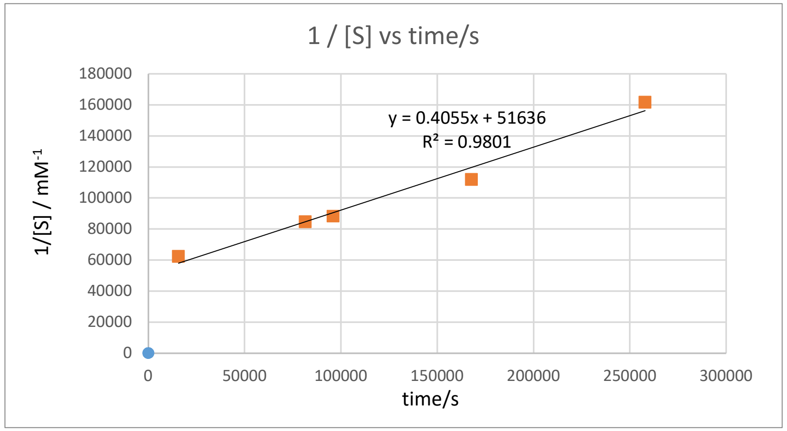

k = 0.40 $mM^{-1}s^{-1}$

Reaction of compound **51** (diNTs) with benzylazide in $CDCl_3$.

Compound **51** (28.8 mg, 50 µmol) and BnN_3 (6.6 mg, 50 µmol) in $CDCl_3$ (0.5 mL, [alkyne](initial) = 100 mM.

* [alkyne] is in M/1000 so that gradient of 1/[S] vs time/s is given as $mM^{-1}\ s^{-1}$ instead of $M^{-1}s^{-1}$

start date/time	end of interval	time elapsed	time interval/s	total time elapsed/s	Time /h	integration of product peak	No. Hs in environment	Integration of SM peak	No. Hs in environment	Conv	[Alkyne]	1/[Alkyne]	Conv /%	Time / s
21/07/2021 13:35	21/07/2021 13:41	0:0:06:00	360.00	360	0.10	0	1	1.00	4	0.000	1.00E-04	10000	0.0	360
21/07/2021 13:41	21/07/2021 14:39	0:0:58:00	3480.00	3480	0.97	0.02	1	4.00	4	0.020	9.80E-05	10200	2.0	3480
21/07/2021 14:39	22/07/2021 09:46	0:19:07:00	68820.00	72300	20.08	0.68	1	4.00	4	0.405	5.95E-05	16800	40.5	72300
22/07/2021 09:46	23/07/2021 10:13	1:0:27:00	88020.00	160320	44.53	1	1	2.51	4	0.614	3.86E-05	25936	61.4	160320
23/07/2021 10:13	24/07/2021 12:51	1:2:38:00	95880.00	256200	71.17	1	1	1.40	4	0.741	2.59E-05	38571	74.1	256200
24/07/2021 12:51	25/07/2021 16:40	1:3:49:00	100140.00	356340	98.98	1.23	1	1.00	4	0.831	1.69E-05	59200	83.1	356340
25/07/2021 16:40	26/07/2021 10:46	0:18:06:00	65160.00	421500	117.08	1.76	1	1.00	4	0.876	1.24E-05	80400	87.6	421500
26/07/2021 10:46	26/07/2021 16:26	0:5:40:00	20400.00	441900	122.75	1	1	0.51	4	0.887	1.13E-05	88431	88.7	441900
26/07/2021 16:26	27/07/2021 10:30	0:18:04:00	65040.00	506940	140.82	3.49	1	1.00	4	0.933	6.68E-06	149600	93.3	506940

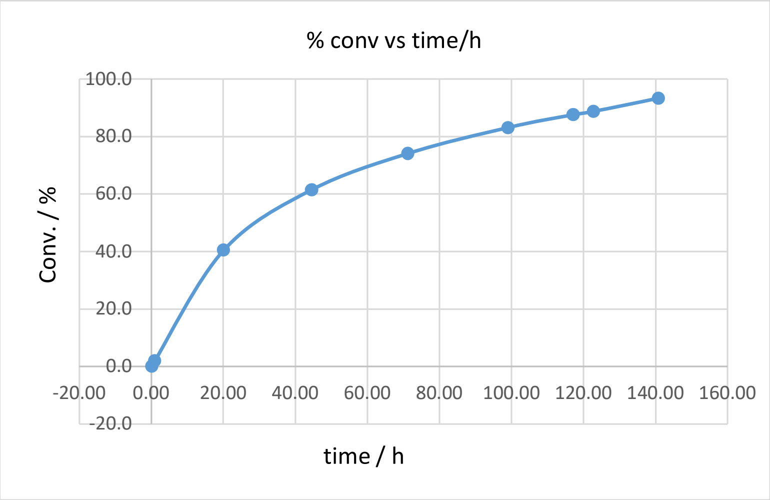

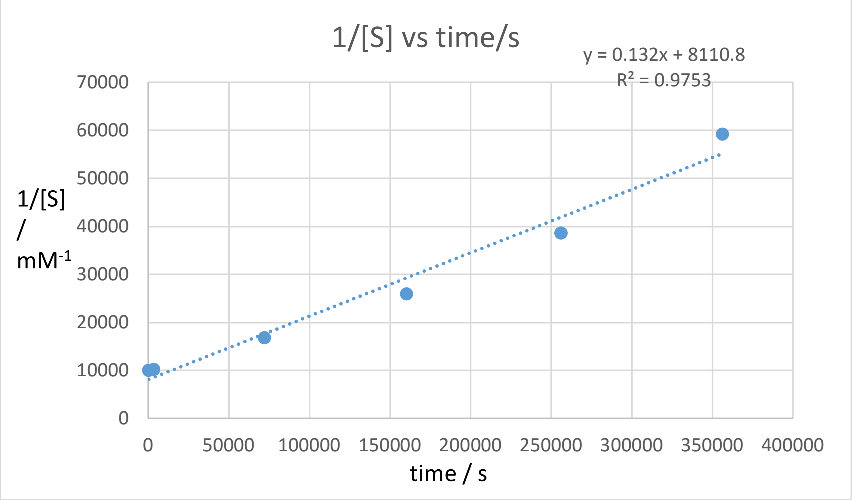

k = 0.13 $mM^{-1}s^{-1}$

Reaction of compound **53** (diMs) with benzylazide in $CDCl_3$.

Compound **53** (10 mg, 24 μmol) and BnN_3 (3.1 mg, 24 μmol) in $CDCl_3$ (0.5 mL, [alkyne](initial) = 48 mM.

time /s	SM peak no Hs	Product integral (2H)	SM peak normalised	P peak normalised	Conv.	Conv/%	1-conv	[Alkyne]	1/[Alkyne]	[Alkyne]t0	[alkyne]	1/[alkyne]
0	4	0.000	1	0.000	0.0000	0.0	1.000	0.0474	21.10	0.0474	0.0000	#DIV/0!
9360	4	0.109	1	0.054	0.0515	5.1	0.949	0.0450	22.24		0.0023	432.28
87300	4	0.810	1	0.405	0.2883	28.8	0.712	0.0337	29.65		0.0097	102.83
162360	4	1.736	1	0.868	0.4646	46.5	0.535	0.0254	39.41		0.0118	84.82
246780	4	3.308	1	1.654	0.6232	62.3	0.377	0.0179	55.99		0.0111	89.85

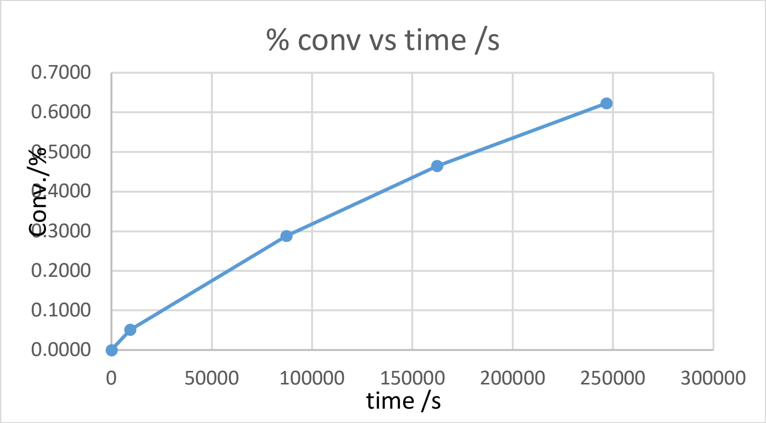

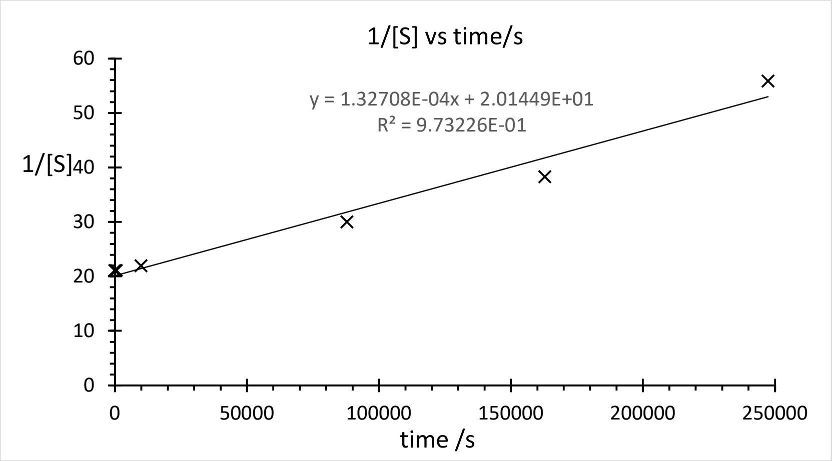

k = 0.13 $mM^{-1}s^{-1}$

Reaction of compound **54** (diTs4C) with benzylazide in $CDCl_3$.

Compound **54** (12.6 mg, 20 μmol) and BnN_3 (2.7 mg, 20 μmol) in $CDCl_3$ (0.5 mL, [alkyne](initial) = 40 mM.

start date/time	end of interval	interval time	interval time/s	time/s	time/h	integration of product peak	No. Hs in environment	Integration of SM peak	No. Hs in environment	Conv	[Alkyne]/ mM	1/[Alkyne]	Conv/%	time/s
02/08/2021 13:52	02/08/2021 13:52	0:0:00:00	0	0	0.000	0	1	2.08	4	0.000	4.00E-05	25000	0.00	0
02/08/2021 13:52	02/08/2021 14:27	0:0:35:00	2100	2100	0.583	1	1	2.08	4	0.658	1.37E-05	7.31E+04	65.79	2100
02/08/2021 14:27	02/08/2021 15:38	0:1:11:00	4260	6360	1.767	1	1	0.44	4	0.901	3.96E-06	2.52E+05	90.09	6360
02/08/2021 15:38	02/08/2021 23:20	0:7:42:00	27720	34080	9.467	1	1	0.05	4	0.988	4.94E-07	2.03E+06	98.77	34080

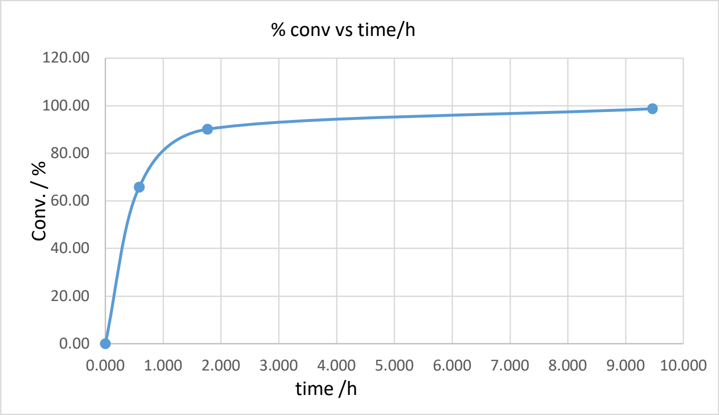

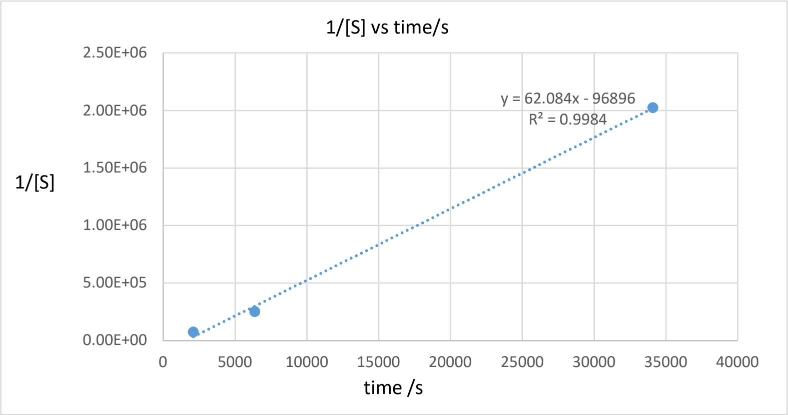

k = 62.1 $mM^{-1}s^{-1}$

Reaction of compound **54** (diTs4C small scale) with benzylazide in $CDCl_3$.

Compound **54** (1.2 mg, 2 μmol) and BnN_3 (0.3 mg, 2 μmol) in $CDCl_3$ (0.5 mL), [alkyne](initial) = 4 mM.

start date/time	end of interval	interval time	interval time/s	time/s	time/h	integration of product peak	No. Hs in environment	Integration of SM peak	No. Hs in environment	Conv	[Alkyne]	1/[Alkyne]	Conv/%	tme/s
06/11/2021 09:03	06/11/2021 09:14	0:0:11:00	660	660	0.183	0.05	1	4	4	0.048	4.00E-03	250	4.76	660
06/11/2021 09:14	06/11/2021 09:44	0:0:30:00	1800	2460	0.683	0.26	1	4	4	0.206	3.17E-03	3.15E+02	20.63	2460
06/11/2021 09:44	06/11/2021 10:14	0:0:30:00	1800	4260	1.183	0.46	1	4	4	0.315	2.74E-03	3.65E+02	31.51	4260
06/11/2021 10:14	06/11/2021 10:44	0:0:30:00	1800	6060	1.683	0.72	1	4	4	0.419	2.33E-03	4.30E+02	41.86	6060
06/11/2021 10:44	06/11/2021 11:14	0:0:30:00	1800	7860	2.183	0.96	1	4	4	0.490	2.04E-03	4.90E+02	48.98	7860
06/11/2021 11:14	06/11/2021 11:44	0:0:30:00	1800	9660	2.683	1.27	1	4	4	0.559	1.76E-03	5.68E+02	55.95	9660
06/11/2021 11:44	06/11/2021 12:14	0:0:30:00	1800	11460	3.183	1.63	1	4	4	0.620	1.52E-03	6.58E+02	61.98	11460
06/11/2021 12:14	06/11/2021 12:44	0:0:30:00	1800	13260	3.683	1.92	1	4	4	0.658	1.37E-03	7.30E+02	65.75	13260
06/11/2021 12:44	06/11/2021 13:14	0:0:30:00	1800	15060	4.183	2.32	1	4	4	0.699	1.20E-03	8.30E+02	69.88	15060
06/11/2021 13:14	06/11/2021 13:44	0:0:30:00	1800	16860	4.683	2.69	1	4	4	0.729	1.08E-03	9.23E+02	72.90	16860
06/11/2021 13:44	06/11/2021 14:14	0:0:30:00	1800	18660	5.183	3.17	1	4	4	0.760	9.59E-04	1.04E+03	76.02	18660
06/11/2021 14:14	06/11/2021 14:44	0:0:30:00	1800	20460	5.683	3.75	1	4	4	0.789	8.42E-04	1.19E+03	78.95	20460
06/11/2021 14:44	06/11/2021 15:14	0:0:30:00	1800	22260	6.183	4.07	1	4	4	0.803	7.89E-04	1.27E+03	80.28	22260
06/11/2021 15:14	06/11/2021 15:44	0:0:30:00	1800	24060	6.683	4.47	1	4	4	0.817	7.31E-04	1.37E+03	81.72	24060
06/11/2021 15:44	06/11/2021 16:14	0:0:30:00	1800	25860	7.183	5.37	1	4	4	0.843	6.28E-04	1.59E+03	84.30	25860
06/11/2021 16:14	06/11/2021 16:44	0:0:30:00	1800	27660	7.683	5.74	1	4	4	0.852	5.93E-04	1.69E+03	85.16	27660
06/11/2021 16:44	06/11/2021 17:14	0:0:30:00	1800	29460	8.183	6.35	1	4	4	0.864	5.44E-04	1.84E+03	86.39	29460
06/11/2021 17:14	06/11/2021 17:44	0:0:30:00	1800	31260	8.683	7.31	1	4	4	0.880	4.81E-04	2.08E+03	87.97	31260
06/11/2021 17:44	06/11/2021 18:14	0:0:30:00	1800	33060	9.183	7.94	1	4	4	0.888	4.47E-04	2.24E+03	88.81	33060
06/11/2021 18:14	06/11/2021 18:44	0:0:30:00	1800	34860	9.683	8.81	1	4	4	0.898	4.08E-04	2.45E+03	89.81	34860

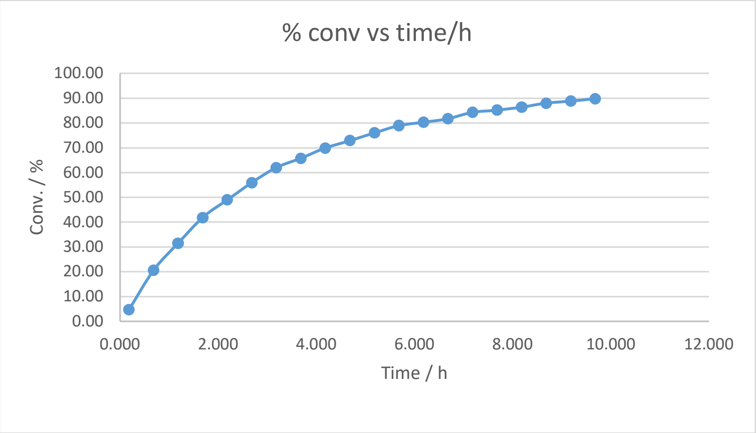

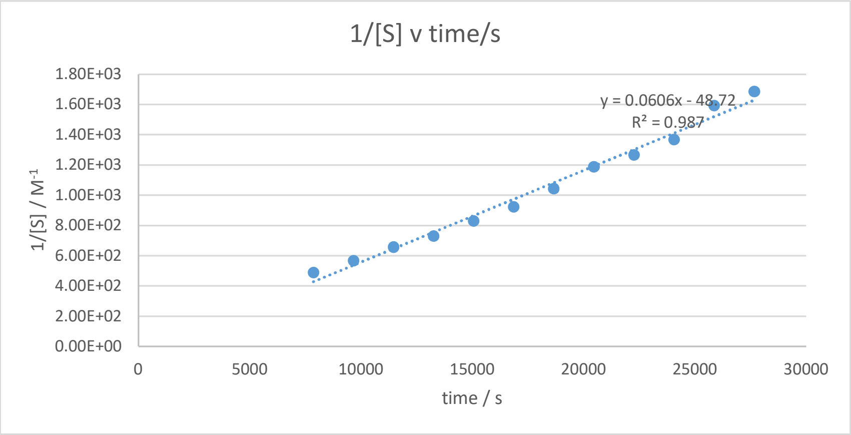

k = 60 $mM^{-1}s^{-1}$

Reaction of compound **56** (diMs4C) with benzylazide in $CDCl_3$.

Compound **56** (5.2 mg, 11 μmol) and BnN_3 (1.5 mg, 11 μmol) in $CDCl_3$ (0.5 mL), [alkyne](initial) = 22 mM.

NMR	Time/s	Alkyne CH2 integral	Alkyne CH2 integral normalised	Product CH2 integral	Product CH2 integral normalised	Conversion	[Alkyne]	1/[Alkyne]	time	Time step	Cumulative time/s	$[Alkyne]_0$/ M
									11:36			0.0218
0	0	100	25	0.00	0.00	0.0000	0.02185	45.8	12:02	0	0	
1	420	100	25	0.61	0.31	0.0121	0.02158	46.3	12:09	420	420	
2	540	100	25	1.17	0.59	0.0230	0.02135	46.8	12:11	120	540	
3	660	100	25	1.67	0.83	0.0322	0.02114	47.3	12:13	120	660	
4	2400	100	25	7.12	3.56	0.1246	0.01913	52.3	12:42	1740	2400	
5	2520	100	25	7.70	3.85	0.1334	0.01893	52.8	12:44	120	2520	
6	4620	100	25	14.19	7.10	0.2211	0.01702	58.8	13:19	2100	4620	
7	4800	100	25	14.72	7.36	0.2275	0.01688	59.2	13:22	180	4800	
8	6000	100	25	18.85	9.43	0.2738	0.01587	63.0	13:42	1200	6000	
9	6120	100	25	19.90	9.95	0.2847	0.01563	64.0	13:44	120	6120	
10	8460	100	25	27.46	13.73	0.3545	0.01410	70.9	14:23	2340	8460	
11	12060	100	25	40.01	20.00	0.4445	0.01214	82.4	15:23	3600	12060	
12	16440	100	25	56.52	28.26	0.5306	0.01026	97.5	16:36	4380	16440	
13	21120	100	25	74.08	37.04	0.5970	0.00880	113.6	17:54	4680	21120	
14	27240	100	25	100.33	50.16	0.6674	0.00727	137.6	19:36	6120	27240	
15	32160	100	25	121.66	60.83	0.7087	0.00636	157.1	20:58	4920	32160	
16	32280	100	25	124.35	62.18	0.7132	0.00627	159.6	21:00	120	32280	
17	41100	100	25	166.40	83.20	0.7690	0.00505	198.1	23:27	8820	41100	
18	79500	100	25	381.29	190.65	0.8841	0.00253	394.8	10:07	38400	79500	
19	93900	100	25	423.25	211.63	0.8943	0.00231	433.2	14:07	14400	93900	

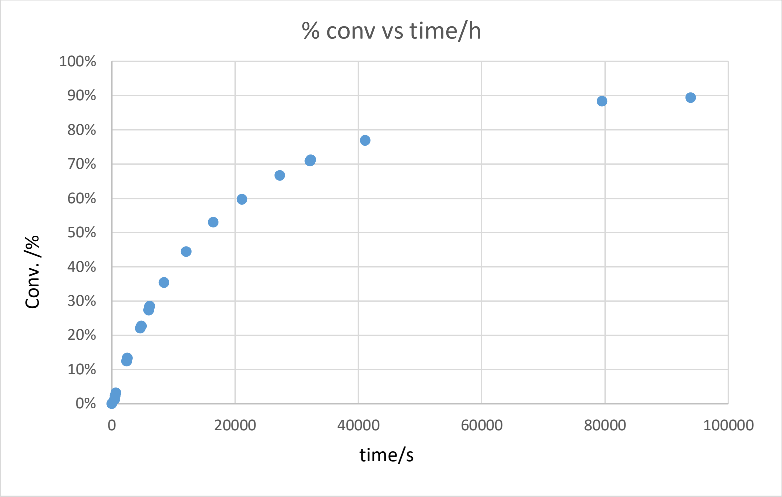

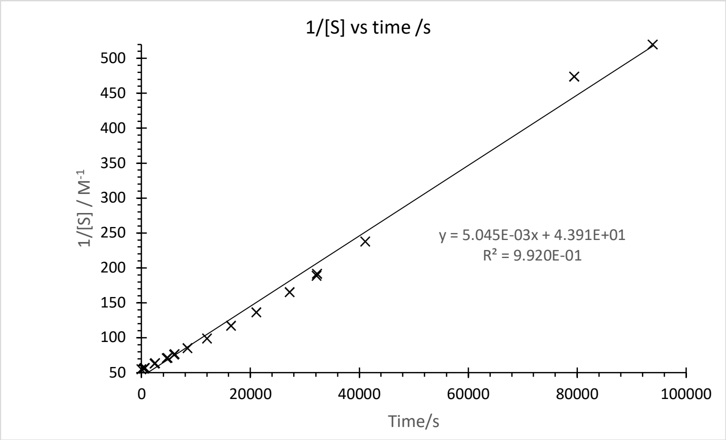

k = 5.0 $mM^{-1}s^{-1}$

Reaction of compound **54** (diTs4C) with benzylazide in d6-DMSO.

Compound **54** (SF759) (3.5 mg, 5.6 μmol) and BnN_3 (0.7 mg, 5.6 μmol) in d6-DMSO (0.5 mL) hence [alkyne](initial) = 11.2 mM.

Azide added 20/22/23 12.00.

Sample	time	SM 1H integral	Product 1H integral	t/min	Conv./%	t/s	[S]/M	1/[S] /mM-1
Alkyne only	11.1	1	0	0	0	0	0.01120	89.3
PhCH2N3 added 12.00	12.02	1	0.02	2	0	120	0.01120	89.3
follow up 1 12:28	12.35	0.59	0.36	35	38	2100	0.00694	144.0
follow up 2 13:35	13.41	0.41	0.51	101	55	6060	0.00504	198.4
follow up 3 14:30	14.54	0.33	0.61	174	65	10440	0.00392	255.1
follow up 4 17:30	17.42	0.25	0.67	342	72	20520	0.00314	318.9
am on 21/11/23	8.24	0.25	0.74	1224	75	73440	0.00280	357.1

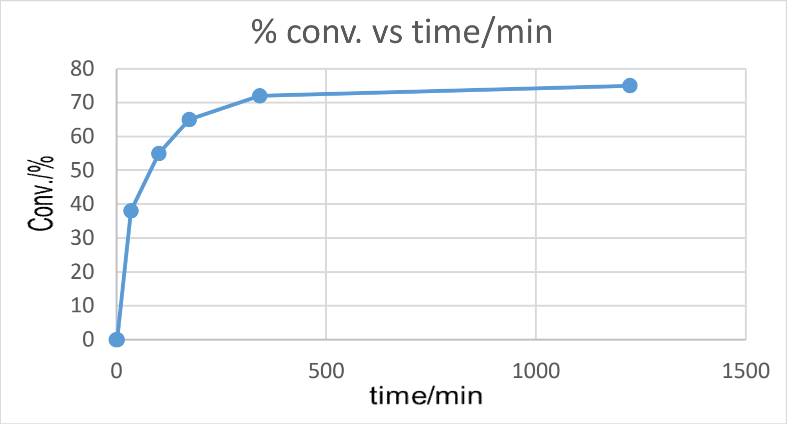

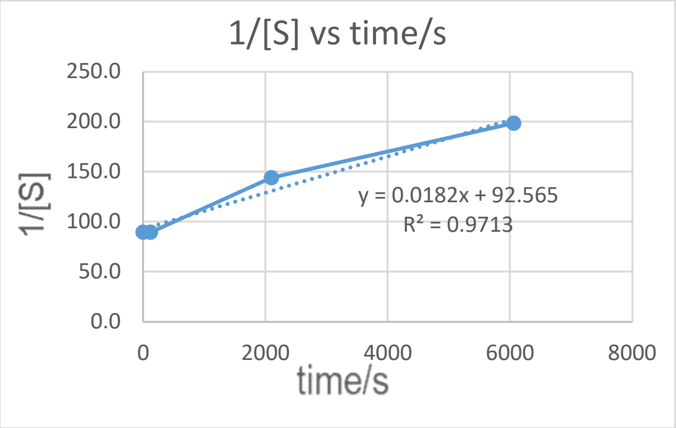

rate = 0.0182 M-1 s-1

18.3 mM-1 s-1

Reaction of compound **56** (diMs4C) with benzylazide in d6-DMSO.

Compound **56** (AS31) (3.1 mg, 6.5 μmol) and BnN_3 (0.86 mg, 6.5 μmol, 9μL) in d6-DMSO (0.5 mL) hence [alkyne](initial) = 13.0 mM.

Azide added at 11.20.

Sample	time	sm 1H integral	product 1H integral	t/min	% product	t/s	[S]/M	1/[S]
alkyne only	11.03	1	0	0	0	0	0.01300	76.9
PhCH2N3 added 11.20	11.3	0.5	0.05	10	9	600	0.01183	84.5
Follow up 1 11:34	11.38	0.5	0.13	18	21	1080	0.01027	97.4
Follow up 2 12:39	13.06	0.5	0.56	106	53	6360	0.00611	163.7
Follow up 3 14:19	14.23	0.5	0.9	183	64	10980	0.00468	213.7
Follow up 4 15:13	15.13	0.5	1.07	233	68	13980	0.00379	264.2
Follow up 5 17:31	17.34	0.5	1.86	391	79	23460	0.00216	463.7
Follow up 6 22:14	22.17	0.5	2.96	657	86	39420	0.00086	1169.0
am 231123	8.45	0.5	9.9	1285	95	77100	0.00023	4273.5

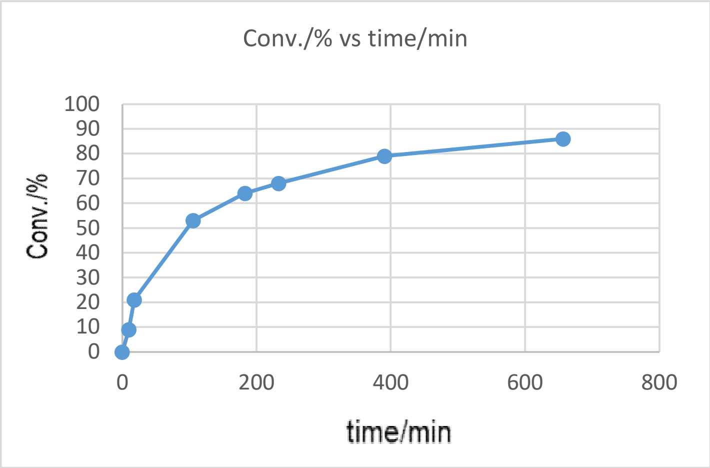

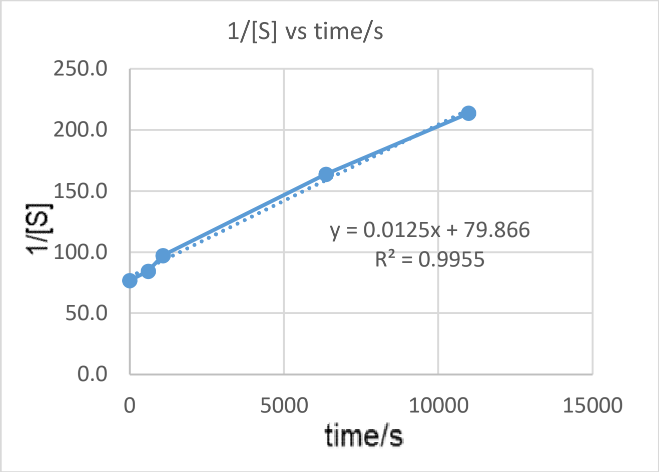

k = 12.5 mM-1 s-1

Reaction of compound **54** (DiTsC4) with phenylazide in $CDCl_3$.

SF759 (3.5 mg, 5.6 μmol) and PhN_3 (11.2 μL of a 0.5 M solution in tBuOMe, 5.6 μmol) in $CDCl_3$ (0.5 mL) [alkyne](initial) = 11.2 mM

The NMR spectra indicated that the PhN3 was in excess, hence a second order rate constant cannot be generated.

		time	SM integral/ H	Product integral/H	time/min	conv/%	t/s	[S]/M	1/[S]
before PhN3	400 10	11.05	1	0					
PhN3 added	300 10	11.57	0.25	0.07	0	22	0	0.01120	89
follow up 1 12:26	400 30	12.31	0.25	0.42	34	63	2040	0.00414	241
follow up 2 13:36	400 60	13.44	0.11	1	107	90	6420	0.00112	893
follow up 3 14:30	400 80	14.51	0.025	1	174	97	10440	0.00034	2976
follow up 4 17:30	400 100	23.32	0.01	1	690	99	41400	0.00011	8929
211123	400 10		0	1	2400	100	144000	0	n/a

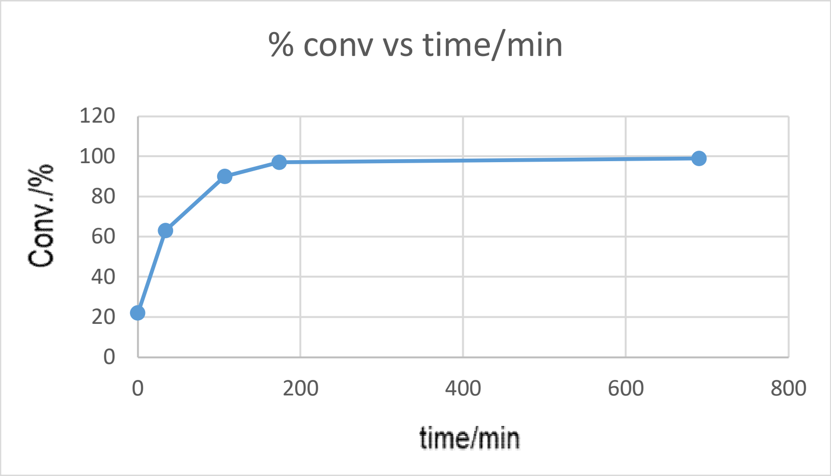

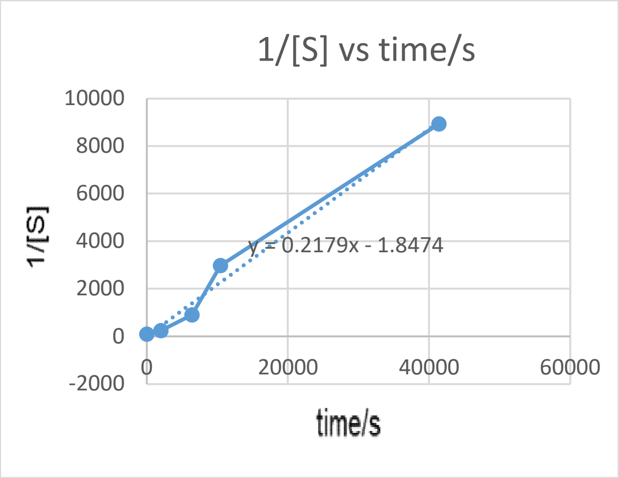

k = 0.22 M-1 s-1 inaccurate due to excess PhN_3

Reaction of compound **56** (DiMsC4) with phenylazide in $CDCl_3$.

AS31 + $CDCl_3$ + PhN_3 PhN3 concentration low at start and more added later.

Compound **56** (AS31) (4.0 mg, 8.4 µmol) and PhN_3 (0.5 M solution in tBuOMe, 0.0084 mmoL, 16.8 µL) in $CDCl_3$ (0.5 mL) hence [alkyne](initial) = 16.8 mM.

21 11 2023	run	time	SM integral/H	Product in	t/min	% product	
before PhN3	400 30	8.48	1	0		0	
PhN3 added	400 50	9.36	1	0	21	0	
09:50	400 70	9.55	0.25	0.01	40	4	
10:55	400 90	11	15.3	1	75	6	
12:23	400 110	12.28	0.25	0.03	191	10	Additional PhN_3 added
13:54	400 130	14.12	0.25	0.06	297	19	
15:51	400 140	16.01	0.25	0.1	406	29	
18:16	400 150	19.45	0.25	0.17	630	40	
22-Nov	400 10	8.28	0.25	0.4	1400	62	

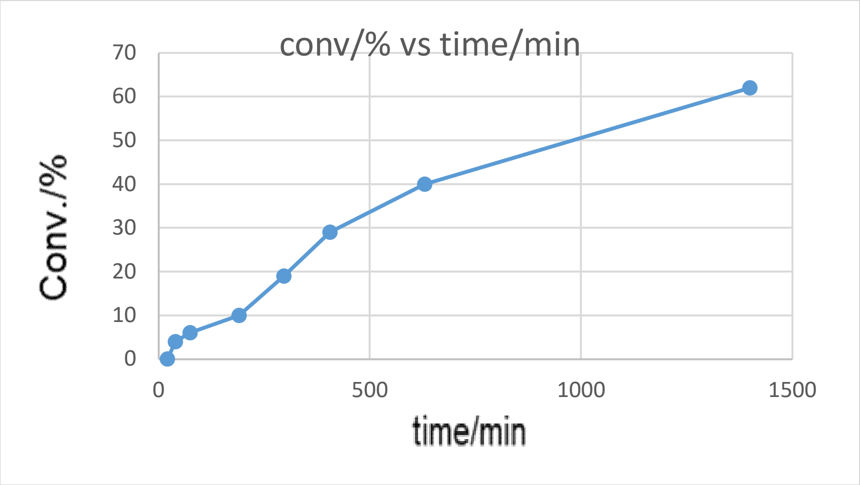

An accurate k value cannot be generated.
